# Genome-wide characterization, evolution, structure, and expression analysis of the F-box genes in *Caenorhabditis*

**DOI:** 10.1186/s12864-021-08189-7

**Published:** 2021-12-11

**Authors:** Ailan Wang, Wei Chen, Shiheng Tao

**Affiliations:** 1 State Key Laboratory of Crop Stress Biology in Arid Areas and College of Life Sciences, Northwest A & F University, Yangling, 712100 Shaanxi China; 2 Bioinformatics Center, Northwest A&F University, Yangling, Shaanxi China; 3 Geneis (Beijing) Co., Beijing, China

**Keywords:** F-box gene family, *Caenorhabditis*, Copy number variation, Tandem duplication, Intron elongation, Sequence divergence

## Abstract

**Background:**

F-box proteins represent a diverse class of adaptor proteins of the ubiquitin-proteasome system (UPS) that play critical roles in the cell cycle, signal transduction, and immune response by removing or modifying cellular regulators. Among closely related organisms of the *Caenorhabditis* genus, remarkable divergence in F-box gene copy numbers was caused by sizeable species-specific expansion and contraction. Although F-box gene number expansion plays a vital role in shaping genomic diversity, little is known about molecular evolutionary mechanisms responsible for substantial differences in gene number of F-box genes and their functional diversification in *Caenorhabditis*. Here, we performed a comprehensive evolution and underlying mechanism analysis of F-box genes in five species of *Caenorhabditis* genus*,* including *C. brenneri*, *C. briggsae*, *C. elegans*, *C. japonica*, and *C. remanei*.

**Results:**

Herein, we identified and characterized 594, 192, 377, 39, 1426 F-box homologs encoding putative F-box proteins in the genome of *C. brenneri*, *C. briggsae*, *C. elegans*, *C. japonica*, and *C. remanei,* respectively. Our work suggested that extensive species-specific tandem duplication followed by a small amount of gene loss was the primary mechanism responsible for F-box gene number divergence in *Caenorhabditis* genus. After F-box gene duplication events occurred, multiple mechanisms have contributed to gene structure divergence, including exon/intron gain/loss, exonization/pseudoexonization, exon/intron boundaries alteration, exon splits, and intron elongation by tandem repeats. Based on high-throughput RNA sequencing data analysis, we proposed that F-box gene functions have diversified by sub-functionalization through highly divergent stage-specific expression patterns in *Caenorhabditis* species.

**Conclusions:**

Massive species-specific tandem duplications and occasional gene loss drove the rapid evolution of the F-box gene family in *Caenorhabditis*, leading to complex gene structural variation and diversified functions affecting growth and development within and among *Caenorhabditis* species*.* In summary, our findings outline the evolution of F-box genes in the *Caenorhabditis* genome and lay the foundation for future functional studies.

**Supplementary Information:**

The online version contains supplementary material available at 10.1186/s12864-021-08189-7.

## Background

The formation of novel genes plays an essential role in biological evolution, such as morphological innovations and adaptation to environmental changes. Organisms can acquire novel genes through various molecular processes. For instance, genomic rearrangements, retroposition, horizontal gene transfer, and duplication-divergence of existing genes are responsible for novel gene birth [1]. The novel genes derived from different evolution mechanisms have distinct molecular signatures and are not equally active in all genomes. Among all of these evolutionary mechanisms for generating novel genes, gene duplication is a significant contributor that facilitates organisms to adapt to dynamically changing environments [2, 3].

Multiple possible evolutionary fates have been proposed for duplicated genes [4, 5]. The most likely fate of a duplicated gene is pseudogenization (i.e., unexpressed or functionless). Given increased gene dosage is beneficial, two gene copies will preserve the original gene function [6], the evolutionary process of which is also referred to as concerted evolution [7]. Another evolutionary fate is sub-functionalization, in which each daughter gene adopts partial original functions of their parental gene [2]. One of the most critical outcomes of gene duplication is neofunctionalization, with one copy undergoing adaptive changes and another maintaining ancestral function [3, 8, 9]. Each of these processes can retain duplicate genes in different conditions [10–13].

In *Caenorhabditis* species, gene duplication has been a vital evolutionary force for generating genetic diversity among F-box proteins [14]. F-box proteins are a class of substrate adaptor proteins that function in SKP1–CUL1–F-box protein (SCF)-mediated ubiquitination protein degradation pathway [15]. The number of F-box genes varies dramatically among closely related species or subspecies [15]. F-box genes are the largest and fastest evolving gene family in plants [16–19]. For instance, the number of F-box Kelch genes (FBKs) tremendously varies among *Arabidopsis thaliana*, *Oryza sativa*, *Poulus trichocarpa*, and *Vitis vinifera* [17]. Their species-specific metabolism might require a large number of F-box proteins, such as responses to various hormones [20], the circadian clock and photomorphogenesis [21, 22], flower development [23], and defense responses [24]. However, F-box genes are small in most investigated animals and relatively conserved among closely related species. For instance, the F-box gene number varies from 66 to 81 in *Euarchontoglires* [25] and only 42–47 in 12 extant *Drosophila* species [26].

In contrast, F-box genes massively expanded in the *Caenorhabditis* genus, and the number of F-box genes is even more than 1 thousand [14]. However, few studies have considered how these numerous F-box genes are generated in the genomes of *Caenorhabditis* species and why they were preserved after duplicated. To illustrate these intriguing undocumented scientific problems, we investigated mechanisms responsible for F-box gene number divergence in *Caenorhabditis* and their gene structural and functional diversification.

## Results

### Prediction of F-box genes and their protein domain architectures

The F-box domain is ∼40 amino acids long near the N terminus of E3 ubiquitin ligase, and a well-known function is acting as a Cullin1 adapter for ubiquitin-mediated proteolysis [27, 28]. Comprehensive genomic characterization defined F-box domain-encoding genes in five *Caenorhabditis* species (*C. brenneri*, *C. briggsae*, *C. elegans*, *C. japonica*, and *C. remanei*) and one outgroup (*P. pacificus*), based on an approach combining software HMMER, ScanProsite, PSI-BLAST, and InterProScan. The pairwise comparison of F-box HMM logos showed the high similarity between the F-box proteins identified in each species and the known F-box proteins confirming our approach (Fig. S2). The identified F-box domain-containing protein sequences in FASTA format are found in supplemental datasets S1, S2, S3, S4, S5, S6, which are *C. brenneri*, *C. briggsae*, *C. elegans*, *C. japonica*, *C. remanei* and *P. pacificus* F-box proteins, respectively. The identified F-box genes varied considerably among the five species, from 39 members in *C. japonica* to 1426 members in *C. remanei* (Table 1). Hence, *C. japonica* has the minimum number of F-box genes, and even *P. pacificus* has more F-box genes, reaching 97. For five *Caenorhabditis* species, the size of the F-box gene number is not proportional to the total number of genes in corresponding genomes, suggesting changes in the importance of the F-box gene family in *Caenorhabditis.* Intriguingly, the 1426 F-box genes in the *C. remanei* genome account for ~ 4.5% of total coding potential. In contrast, the proportion of F-box genes identified in the *C. japonica* genome has dropped to 0.13%.

**Table 1:** The number of F-box protein-coding genes identified in six species of nematodes

Species	Hmmer	psscan	Hmmer & psscan	psiblast	F-box genes	Genome genes	Percent
*C. brenneri*	559	471	437	1	594	30,667	1.93%
*C. briggsae*	181	150	139	0	192	21,936	0.88%
*C. elegans*	356	302	281	0	377	20,532	1.84%
*C. japonica*	35	23	19	0	39	29,964	0.13%
*C. remanei*	1377	1252	1203	1	1426	31,444	4.54%
*P. pacificus*	95	79	77	0	97	29,644	0.33%

**Fig. 1:**
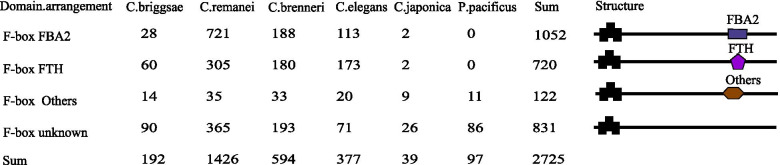
Schematics of domain architectures of main types of F-box proteins from six *Caenorhabditis* species. F-box gene family member representatives with different N terminal functional domains are shown. The C terminal black convex box represents the F-box domain that binds Cullins, and the N terminal blue rectangle and purple pentagon represent F-box associated region 2 (FBA2) and FTH respectively that bind substrate. C terminal known functional domains except FBA2 and FTH were referred to as ‘Others’ that were observed only a few F-box proteins. The numbers represent the count of a particular type of domain in the corresponding species

In five *Caenorhabditis* species, most of the F-box genes with an identified C-terminus functional domain fall into two broad subfamilies: ~ 1052 contain an FBA2 domain, and ~ 720 contain an FTH domain (Fig. 1). In these two family members, the N-terminal domain of the F box is followed by a more divergent region consisting of approximately 300 amino acids domain, called FBA2 or FTH. In contrast to these two types of F-box genes identified in each of five *Caenorhabditis* species, they have no orthologs in *P. pacificus*. The striking contrast result illustrates that F-box-FBA2 and F-box-FTH genes are *Caenorhabditis*-specific and might be generated after the lineage-sorting divergence between *P. pacificus* and the *Caenorhabditis* genus. Among the remaining 856 F-box genes identified in five *Caenorhabditis* species, 111 members include known C-terminal domains such as WD-40, LRR-6, PbH1, and others (data not shown), all of which present in only one or a few F-box proteins. In addition, the remaining 745 F-box genes have no known C-terminal domains that have been characterized.

### Identification of paralogs and orthologs of F-box genes

In the ENSEMBL database, the homology relationships of genes have been inferred and annotated based on sequence similarity, phylogenetic tree, and chromosomal locations. Paralogs and orthologs of F-box genes in five *Caenorhabditis* species were downloaded from the ENSEMBL database using BioMart, respectively. F-box genes are unequally divided into paralogous groups of different sizes (Fig. S3). As shown in Fig. S3, F-box domains were lost from many members of each F-box gene paralogous group. Furthermore, the F-box domains were lost from F-box genes unequally among paralogous groups, accounting for ~ 5% to ~ 98% of the total number of genes in paragroups. Of 2725 identified F-box genes, 1519 members were unequally divided into 270 orthologous groups with different sizes. A total of 1053 genes without F-box domain-encoding regions were considered as orthologs of these F-box genes (Fig. S4). Many orthologs of F-box genes from one species are missing in another *Caenorhabditis* species, presumably by deletion.

Protein sequences from each paragroup and orthogroup were aligned, respectively. We found several mechanisms potentially responsible for the loss of F-box domains that were present in their homologs: (1) multiple point mutations occurred in F-box domain-encoding region; (2) long DNA fragments were inserted into F-box domain regions (3) the whole F-box domain-encoding DNA fragments were deleted from the extant gene (data not shown).

**Fig. 2:**
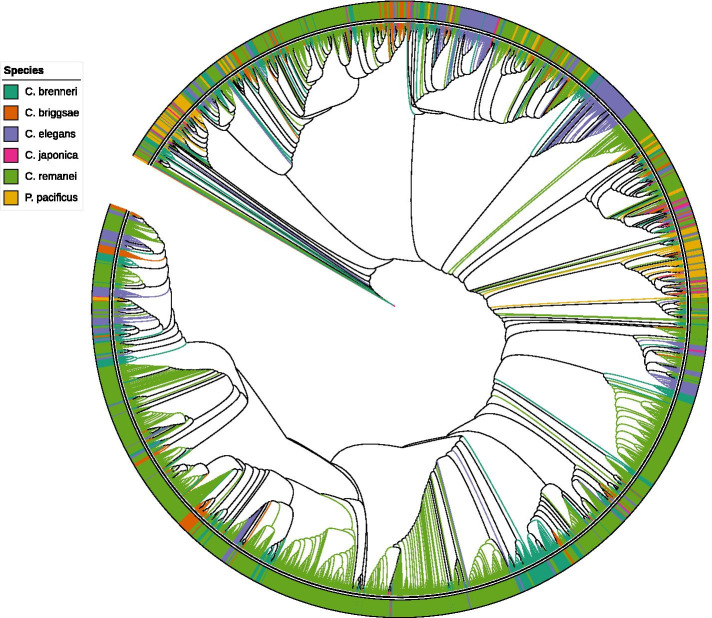
Phylogenetic relationships of F-box proteins from *C. brenneri*, *C. briggsae*, *C. elegans*, *C. japonica*, *C. remanei*, and *P. pacificus* being color-coded light sea green, chocolate, purple, pink, lime-green, and orange, respectively. F-box domain sequences were aligned using MUSCLE. A simplified version of the phylogenetic tree was generated by maximum likelihood implemented in RAxML. The complete phylogeny with statistical support is shown in Fig. S6

A maximum-likelihood phylogenetic tree was constructed based on F-box domain sequences from 2725 proteins (Fig. 2). According to evolutionary stability, the F-box gene family includes two types of genes. One class with clear, conserved orthologs with bootstrap support in the five *Caenorhabditis* species, and a second type without a clear one-to-one orthologous group undergoing rapid birth-death evolution. For simplicity, we refer to the former members as “stable” genes and the latter as “unstable” genes based on their number of evolutionary conservations across five *Caenorhabditis* species. The constructed phylogenetic tree has large species-specific clades and only seven sets of stable orthologous groups, each of which has a single member in each species. The complete phylogeny with statistical support is shown in Fig. S5. We speculate that one-to-one orthologous F-box genes may target endogenous proteins for ubiquitin-mediated degradation as part of conserved normal development or physiology, which change little with time. In striking contrast to stable genes, the unstable F-box genes have continued to evolve rapidly by species-specific and birth-death evolution. It seems reasonable to propose that rapidly evolutionary F-box genes may recognize foreign proteins as part of the nematode innate immune system. Furthermore, exogenous pathogenic virus and bacterial protein are plausible targets, which drive an arms race between pathogens and nematode innate immune system. The phylogenetic tree of the paralogous genes from each species was reconstructed (Fig. S6).

### F-box gene number divergence in *Caenorhabditis* and underlying mechanisms

**Fig. 3:**
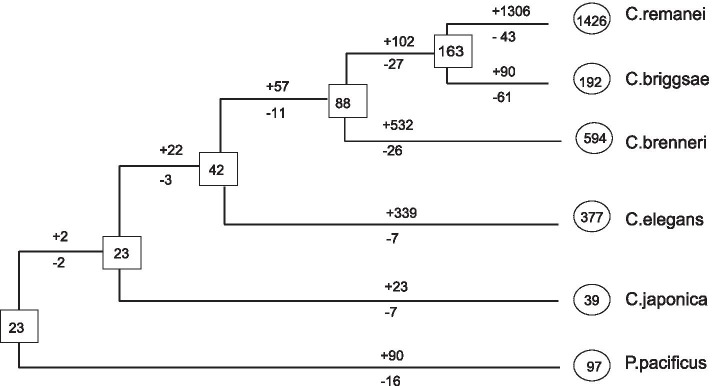
Evolutionary change of the number of F-box genes in the six nematode species. The numbers in circles and rectangles represent the numbers of genes in extant and ancestral species, respectively. The numbers with plus and minus signs indicate the numbers of genes gains and losses, respectively, for each branch

During the long-term process of *Caenorhabditis* evolution, the evolutionary dynamic of F-box genes was investigated by reconciling gene tree and species tree using the maximum parsimony method. A total of 2473 gains and 144 losses events were inferred to have occurred in the F-box gene family in the *Caenorhabditis* lineage (Fig. 3). Linage-specific gene gains and losses remarkably revealed high evolutionary dynamics of F-box genes in the *Caenorhabditis* genus, with only 23 putative F-box genes inferred in ancestral species and as many as 1426 members in *C. remanei*. The number of F-box genes strikingly diverged, particularly after the *Caenorhabditis* species split, from only 39 members in *C. japonica* to the most prominent F-box gene family with 1426 members in *C. remanei*. Unveiling the mechanisms responsible for gene duplications origination and functional divergence would illuminate these F-box genes’ biological function.

The putative F-box genes identified from each *Caenorhabditis* species were mapped to corresponding chromosomes or contigs when chromosome-level genome assembly with high quality was not available. The F-box gene locus on the genome is not accidental, and with most members residing on particular chromosomes or arms. For instance, the vast majority of the F-box genes from *C. elegans* are concentrated and clustered on Chromosomes II, III, and V (Fig. S7a). Similarly, most F-box genes from *C. briggsae* are overrepresented on Chromosomes III and V (Fig. S7b). The number of F-box genes arising by tandem duplication was estimated according to the intergenic distance measured by the number of genes residing in that region. At least 53%, 43, 52, and 74% of F-box genes were inferred from tandem duplications in *C. brenneri*, *C. briggsae*, *C. elegans,* and *C. remanei* genome, respectively (Table 2).

**Table 2:** The number of F-box protein-coding genes arisen through tandem duplications

	F-box genes	Single genes	Tandem duplication	Percent
*C. brenneri*	594	78	315	53%
*C. briggsae*	192	64	82	43%
*C. elegans*	377	67	197	52%
*C. remanei*	1296	96	962	74%

### Gene structural divergence between F-box gene sibling pairs

**Fig. 4:**
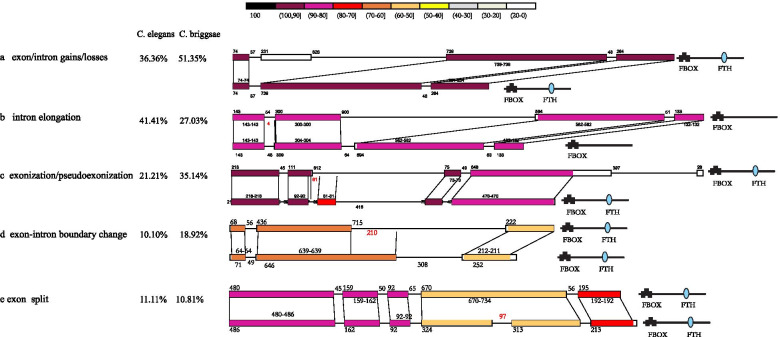
The exon-intron structure of five pairs of representative sibling paralogs and the domain organization of their proteins. The schematic diagram showed five types of underlying mechanisms responsible for structure divergence of F-box gene paralogs: exon/intron gain/loss (**a**), intron region substantial elongation (**b**), exonization/pseudoexonization (**c**), exon-intron boundary change (**d**), and exon split (**e**). The frequency of genes that underwent the corresponding mechanism was shown for F-box paralogs from *C. elegans* and *C. briggsae*. The color in the schematic diagram represented sequence similarity, and the numbers above or below indicate the DNA sequence length in corresponding regions

We compared the gene structures of the closely related F-box gene siblings from *C. elegans* and *C. briggsae* due to their high-quality genome sequences. Gene structural and sequence identical comparisons across 136 sibling pairs from *C. elegans* and *C. briggsae* revealed five distinct mechanisms involved in the divergence of these F-box gene paralogs: 1) exon/intron gains/losses; 2) sequence exonization/pseudoexonization; 3) alteration in exon/intron boundaries; 4) splitting one exon into two; 5) introns elongate/shorten more than twice length of ancestral ones. These five mechanisms that have occurred in five representative sibling pairs are shown in Fig. 4. Comparisons across 99 sibling pairs from *C. elegans* are schematically shown in Fig. S8, with their divergent mechanisms summarized more precisely in Supplementary file 1. Among 99 *C. elegans* sibling pairs, 41 pairs have diverged by introns elongating more than twice the length of the homologous intron. Furthermore, evolutionary events of intron elongation substantially have occurred more than once in some sibling pairs. Subsequently, the second frequent divergent mechanism associated with the F-box gene divergent in *C. elegans* is exon/intro gains and losses. The divergent events caused by the other three mechanisms have occurred in 21, 10, 11 pairs of 99 *C. elegans* F-box gene sibling pairs, respectively. Similarly, comparisons across 37 F-box gene sibling pairs from *C. briggsae* are schematically in Fig. S9, with their divergent mechanisms are summarized more precisely in Supplementary file 1. The F-box gene paralogs divergence patterns in *C. briggsae* are similar to those seen previously in *C. elegans*. The first two frequent patterns of F-box paralogs divergent in *C. briggsae* are exon/intron gains/losses that occurred in half of the compared sibling pairs, followed by intron elongation.

**Fig. 5:**
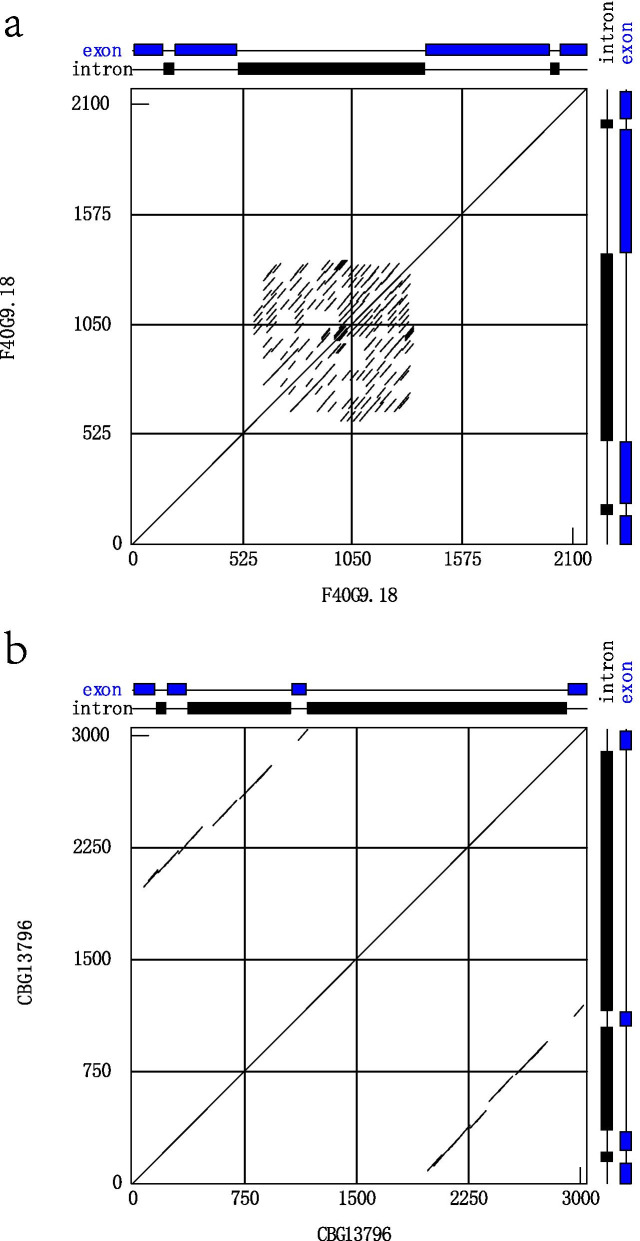
Dot-matrix comparisons of the F-box genes F40G9.18 and CBG13796. A black box denotes intron regions. **a** DNA dot plot analysis of F40G9.18 with itself using window 30, percent match 60. The solid blue lines denote homologous regions with aligning similarities more significant than 60%. **b** DNA dot plot analysis of CBG13796 with itself using window 30, percent match 60. The solid blue lines denote homologous regions with aligning similarities more significant than 60%

The overwhelming pattern of intron elongation substantially in F-box genes from *C. elegans C. briggsae* is intriguing. However, studies associated with the evolution of intron elongation are scarce. Therefore, we investigated the underlying mechanism for intron sequence elongation in F-box genes from *C. elegans* and *C. briggsae*. DotPlot was used for sequence alignment of one sequence with itself for 51 *C. elegans* and *C. briggsae* F-box genes in which the intron sequence elongated substantially. Generally, short-sequence DNA repeats were found in all 51 genes, particularly in elongated intron sequence regions. The results of dot matrix analysis of two representative genes, F40G9.18 and CBG13796, are illustrated in Fig. 5. The closely related paralogs F40G9.18 and F40G9.9 have the same number of exons and introns and > 80% identical coding region. Nevertheless, they substantially diverged at the second intron length, with 900 bp long in the former versus only 64 bp long later (Fig. S8). Remarkably, direct repeats of ~ 50 bp DNA sequence are concentrated on the 500–1400 bp region of F40G9.18, where the intron 2 resides (Fig. 5a). The CBG13796 and CBG13789 are paralogs from *C. briggsae*, with apparently divergent gene structures (Fig. S9). The CBG13796 has an intron 2 of 1743 bp long with multiple ~ 50 bp repeats (Fig. 5b), absent in the paralogs CBG13789. We speculate that the striking divergent gene structure of paralogs CBG13796 and CBG13789 might be caused by the elongation intron. Therefore it is reasonable to conclude that short-sequence DNA repeats result in substantial intron elongation, so they provide the raw materials of evolution for establishing divergent exon-intron structure whereby novel functional gene origination.

### Functional divergence of F-box gene duplicates

**Fig. 6:**
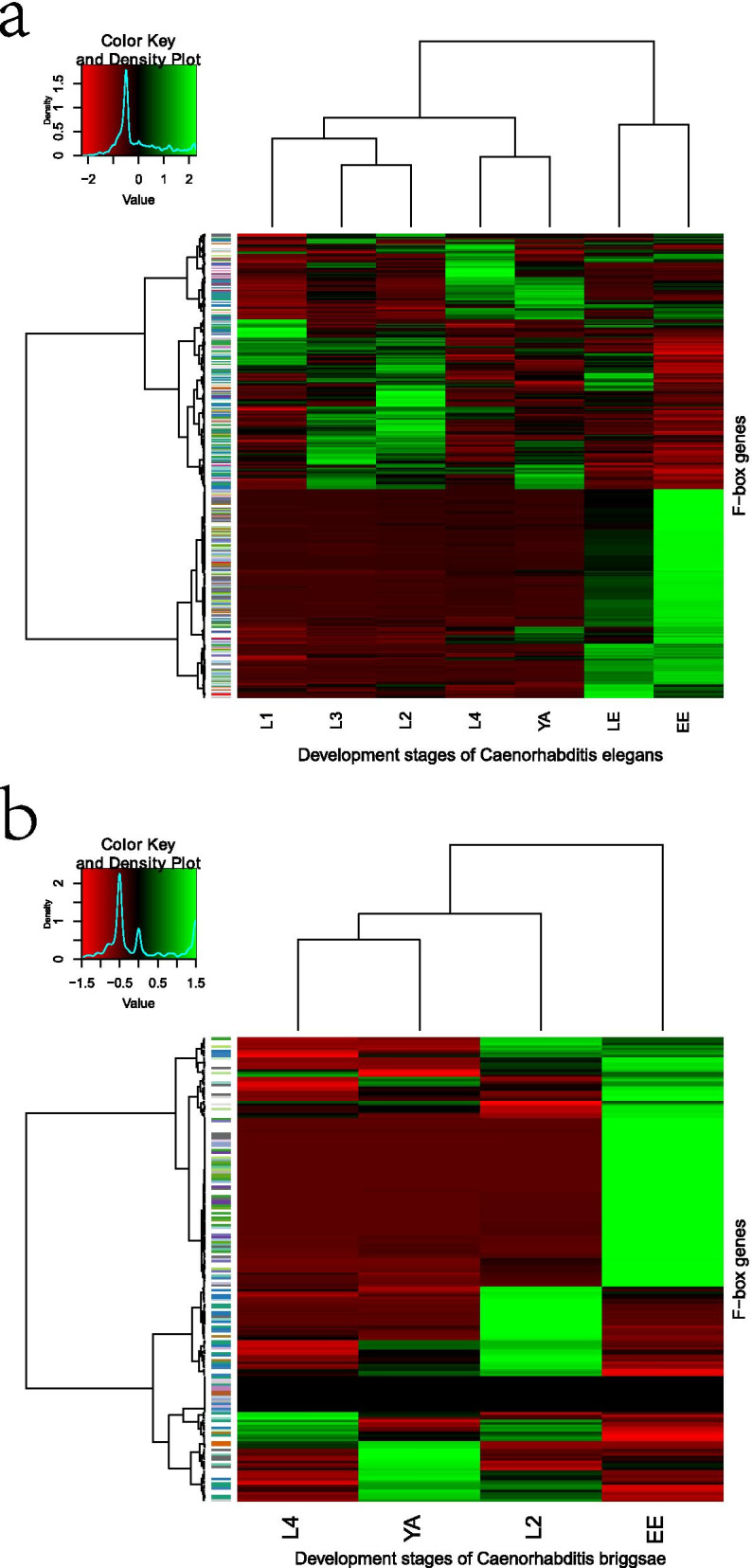
Expression profiles of F-box protein-coding genes differentially expressed during nematode developmental stages. The relative transcript abundances of F-box genes were determined by RNA-seq assay and visualized as a heatmap. The color scale (representing Z-Score of expression signal values) is shown at the top. Similar expression patterns clustered the developmental stages and paralogs. a. Expression profiles of 377 F-box protein-coding genes in *C. elegans* at different developmental stages. The X-axis indicates different developmental stages, including Embryo (EE), L1 Larvae early (LE), L1 Larvae (L1), L2 Larvae (L2), L3 Larvae (L3), L4 Larvae (L4), and Young adult (YA). b. Expression profiles of 192 F-box protein-coding genes in *C. briggsae* at different developmental stages. The X-axis indicates different developmental stages, including Embryo (EE), L2 Larvae (L2), L4 Larvae (L4), and Young adult (YA)

**Fig. 7:**
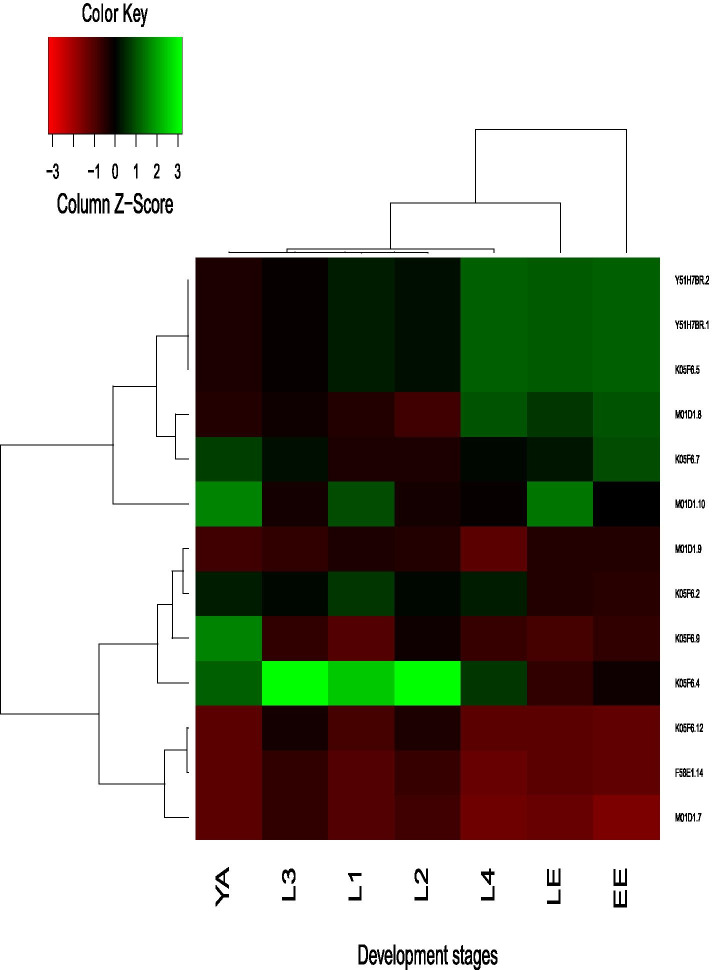
Expression profiles of one representative F-box gene paragroup at seven different development stages *C. elegans*. The color scale (representing Z-Score of expression signal values) is shown at the top. The X-axis indicates different developmental stages, including Embryo (EE), L1 Larvae early (LE), L1 Larvae (L1), L2 Larvae (L2), L3 Larvae (L3), L4 Larvae (L4), and young adult (YA). The Y-axis represents 12 paralogs from the same paragroup of *C. elegans.* The paralogs clustered together indicate conserved similar expression patterns, while scattered paralogs across the tree indicate diverged discrepant expression patterns

One possible mechanism for the functional divergence of duplicated genes is via differential temporal- or spatial-specific expression patterns during evolution [4, 5]. For *C. elegans*, we retrieved gene transcripts for F-box genes expressing at seven developmental stages, including Embryo (EE), L1 Larvae early (LE), L1 Larvae (L1), L2 Larvae (L2), L3 Larvae (L3), L4 Larvae (L4), and Young adult (YA). The Gene expression pattern of F-box genes was compared among seven developmental stages in *C. elegans.* Most F-box genes show a stage-specific expression pattern. Some members have an exceptionally high expression at the embryonic stage, while others have particular high expression at the Larval stage (Fig. 6a). Based on the K-means Cluster method, all members within 42 paralogous groups clustered into eight groups, and none of the paragroups grouped into the same cluster. We observed divergent expression patterns for members of the same paralogous group (Fig. 7). Three paralogs at the bottom of Fig. 7 represent consistent patterns with low expression in each developmental stage, whereas three paralogs at the top show highly expressed in L4, LE, and EE development stages. The remaining seven paralogs, in contrast, have remarkably differential stage-specific expression patterns. Gene expression patterns of 99 closely related sibling pairs were further investigated, and 48 have diverged with differential stage-specific expression patterns.

Similarly, of 192 putative F-box genes identified in *C. briggsae*, gene transcripts are obtained for 177 members expressing at one or more than one stage of four developmental stages, including Embryo (EE), L2 Larvae (L2), L4 Larvae (L4), and Young adult (YA). The Gene expression pattern of these 177 F-box genes was compared among four developmental stages of *C. briggsae.* Divergent expression patterns of F-box genes in *C. briggsae* are comparable with those seen previously in *C. elegans*. The majority of F-box genes show a stage-specific expression pattern (Fig. 6b). Like in *C. elegans*, all 29 F-box gene paragroups from *C. briggsae* also diverged in expression patterns. Furthermore, 22 pairs of 37 close related siblings have differential stage-specific expression patterns. Therefore, it seems reasonable to conclude that these F-box genes have been sub-functionalized via stage-specific gene expression in *C. elegans* and *C. briggsae*.

### Selection pressure on F-box genes

In order to investigate the potential contribution of evolutionary restrictions to sequence differences between paralogs, we estimated the mutation rates and selection patterns of 95 and 37 pairs of F-box paralogs of *C. elegans* and *C. briggsae*, respectively. The pairwise comparison results showed that all dN/dS ratios except one (*Y56A3A.10* vs. *Y56A3A.14*) were smaller than 1 (Supplementary File 1), suggesting that the F-box gene paralogs were subjected to purifying selection. Since the overall strong purification selection may obscure positive selection detection on some regions, we performed dN/dS ratio sliding window analysis in pairwise sequence comparisons. The window size was set to 45 codons, with an offset of nine codons between successive windows. The window size roughly correlated with the size of some structural domains of the F-box proteins. To correct the multiple-testing problem using sliding window analysis, we choose a trial-and-error approach against high false positives [29, 30]. Positive selection was supported only if dN/dS ratio > 1.5 and purifying selection was indicated by dN/dS ratio < 0.67 in sliding windows of 45 codons [29]. The sliding window analysis of dN/dS revealed significant diversified selection features throughout the coding region of F-box genes (Fig. S10 and S11). Although most coding regions were constrained to less than 0.67, some sliding windows analyzed showed dN/dS ratios greater than 1.5 (Supplementary File 1). Most of the dN/dS ratios of the N-terminal region encoding for the first 50 amino acids were less than 1.0 (Fig. S10 and S11), suggesting that the N-terminal F-box domains were under strong purifying selection. Although the entire gene, especially the N-terminal, was subjected to purifying selection, positive selection has occurred in some regions. There were 73 pairs out of 95 analyzed paralogs of *C. elegans*, and 25 pairs out of 37 paralogs of *C. briggsae* showed large peaks with dN/dS value greater than 1.5 at C-terminus, indicating that substrate-targeting domains have undergone positive selection (Fig. S10 and S11). In summary, in contrast to N-terminal F-box domain encoding regions, C-terminal regions have been proposed under less selective pressure.

## Discussion

### F-box gene identifying approach in *Caenorhabditis*

In the present study, the Hidden Markov model, regular expression, and in combination with InterProScan were used to predict F-box protein-encoding genes in Caenorhabditis genus genomes comprehensively. Those highly diverged proteins in the F-box domain region could not be predicted as F-box proteins, although they might still retain F-box protein function. However, more likely, those F-box paralogs that lost F-box domains have evolved into novel functional genes. Although the prediction approach was challenging to avoid false-negative prediction, it was widely applied in numerous studies [31–33]. In addition, the identification of F-box genes in humans using our approach is highly reliable [25]. Notably, the duplicates of identified F-box genes have diverged substantially at corresponding F-box domain regions, contributing to their functional divergence. However, this conjecture should be further confirmed by experimental evidence in the future.

### A significant expansion of F-box genes within *Caenorhabditis* genomes

Although the differentiation time of *Caenorhabditis* species is far longer than that of *Euarchontoglires* species, the variability of the F-box gene in *Caenorhabditis* species is more significant than that of *Euarchontoglires* species [25, 34–36]. Many F-box gene duplicates rapidly diverged at the F-box domain region, such as long sequence fragment insertion/deletion and numerous short sequence repeats in intron regions. Once duplicates emerge, redundant copies may undergo relaxed selection pressure, and mutations in sequences provide raw materials for the evolution of novel function elements [4]. Some members of the F-box gene family were not conserved among *Caenorhabditis*, as each F-box gene in a species does not always have an ortholog in another species. The corresponding ancestral F-box gene may have diverged at the F-box domain region, contributing to the evolution of new traits. The number of F-box genes in *Caenorhabditis* species was substantially more than that of other animals [15] and even more than plants [17]. Thus, based on lineage-specific F-box genes’ colossal expansion and contraction, we proposed that F-box genes in *Caenorhabditis* species show remarkably plastic evolution at the level of gene gains and gene loss.

G protein-coupled receptors (GPCRs) form the largest superfamily of cell surface receptors in *Caenorhabditis* species [37]; *C. elegans* genome encodes approximately 1300 GPCRs genes, most of which were identified in related species *C. briggsae* and *C. remanei* [37]*.* GPCRs in *Caenorhabditis* species include 19 prominent families, some of which were species-specific expanded primarily in *C. elegans and C. remanei* [37]*.* Many studies in *C. elegans* demonstrated the crucial role of GPCRs in innate immunity via their signaling in many physiological processes and for detecting a variety of environmental signals, including bacterial secondary metabolites [38, 39]. Therefore, we conclude that the evolutionary dynamics of GPCRs are comparable with that of the F-box gene family in *Caenorhabditis* species. Furthermore, we speculate that the F-box gene family and GPCRs may function as regulators of innate immunity and are involved in the same physiological process.

### Rapid sequence and expression divergence of F-box genes in *Caenorhabditis*

The present study investigated the gene structure and expression divergence mechanisms of closely related F-box gene paralogs in *C. briggsae* and *C. elegans*. In such short twenty million years of evolutionary history since the speciation of *Caenorhabditis* genus [40], the number of F-box genes massively gain and loss in certain species of *Caenorhabditis* genus. For instance, *C. elegans* requires F-box protein fog-2 [41] that regulates the translation of tra-2 mRNAs during hermaphrodite development [42]. However, *C. briggsae* lacks fog-2 [43] and instead uses a novel F-box protein *she-1* created by recent gene duplication and acts upstream of tra-2 as fog-2 does in *C. elegans* [44]. Thus, both species recruited F-box genes produced by recent duplication events into the sex-determination pathway to control hermaphrodite development, but they use distinct paralogs. This result implies not only the number of F-box genes massive gain and loss in particular species of *Caenorhabditis* genus, but also F-box gene duplicates rapidly diverged at expression. In addition, a stage-specific expression pattern of closely related F-box paralogs was widely observed during the physical development of *C. briggsae* and *C. elegans*, indicating that the function of F-box paralogs may have been sub-function. We speculated that the rapid evolution of F-box genes in Caenorhabditis species was driven by the requirement of adaptation to living environment change.

F-box genes displayed significant gene number variation, structural and functional, and expression pattern divergence, implying that these genes play an essential function in the environmental adaptation and reproduction process [14]. A study showed that the SCF complex responds to microsporidiosis and virus-mediated ubiquitin [45]. The target of the immune proteasome was ubiquitinated by E3 ubiquitin ligase, although no evidence shows which Culling and adaptor protein was involved in this process. Thomas conjectured that the ancestor system of Culling degradation of exogenous proteins is also the ancestor of MHC I [14]. The exogenous and endogenous cullin adaptor proteins might be identified via evolutionary studies if the conjecture was correct.

## Conclusions

This study analyzed the underlying mechanisms of F-box gene sequences divergence, gene expression pattern, and gene number gains/losses in five *Caenorhabditis* species. We identified 594, 192, 377, 39, 1426 F-box homologs in the genome of *C. brenneri*, *C. briggsae*, *C. elegans*, *C. japonica*, and *C. remanei,* respectively. In particular, we found that tandem duplications have played an essential role in the enormous expansion of the F-box gene family. There are many mechanisms identified for F-box gene structural divergence. Moreover, analyses of their expression profiles provide functional information for members of the F-box gene family in *C. elegans* and *C. briggsae* at different development stages. Importantly, our results shed light on the evolution pattern of F-box genes in *Caenorhabditis* species, which will provide a valuable resource for understanding the biological roles of individual F-box genes*.*

## Methods

### Data retrieval

The proteomic sequences of five *Caenorhabditis* species (*C. brenneri*, *C. briggsae*, *C. elegans*, *C. japonica*, *C. remanei*) and one outgroup species (*P. pacificus*) were downloaded from the ENSEMBL Genome Browser. The Hidden Markov model and Prosite file of F-box domain were downloaded from PFAM (http://pfam.xfam.org/family/f-box#tabview=tab6) [46] and PROSITE respectively (ftp://ftp.expasy.org/databases/prosite/) [47]. Transcriptome sequencing data of different developmental phases of *C. elegans* and *C. briggsae* was downloaded from modENCODE (http://www.modencode.org/) [48].

### Genome-wide prediction of F-box genes in five species of *Caenorhabditis*

Hmmersearch program implemented in HMMER software [49] was used to search for F-box domain-containing proteins in the proteome sequences of five *Caenorhabditis* species and.

*P. pacificus*. We also used regular expression implemented in local script multi-thread ps_scan.pl, a parallel computing Perl program modified from ps_scan.pl downloaded from PROSITE [47] to predict F-box proteins. Finally, to comprehensively predict F-box proteins that diverged largely at the F-box domain, the above-identified F-box proteins were used as a PSI-BLAST (e-value = 1e-30) search query against proteome sequences. All of the identified putative F-box proteins were then scanned for F-box domains using modified multi-thread iprscan_lwd.pl downloaded from InterProScan [50]. The longest protein isoform per gene was retained as the final F-box protein dataset. F-box domain sequences were aligned separately for each species. The HMM profiles were built using ‘hmmbuild’ from the HMMER package, followed by pairwise alignment of the built profile HMMs with F-box profile from PFAM database using LogoMat-P [51]. A schematic overview of the whole pipeline is shown in Fig. S1.

### Identification of homology relationship between F-box genes

The paralogs of each F-box gene were downloaded from ENSEMBL using Biomart. Genes that were paralogous to each other were considered as a paralogous group (paragroup). The F-box gene orthologs in five *Caenorhabditis* species were downloaded from ENSEMBL using Biomart. F-box genes that were orthologous to each other were considered as an orthologous group (orthogroup). The pipeline for prediction of gene orthology/paralogy relationships in ENSEMBL include the following basic steps: 1) Load a representative translation of each gene from all species used in Ensembl; 2) Run an HMM search on the TreeFam HMM library to classify the sequences into their families; 3) Cluster the genes that did not have any match into additional families: run NCBI Blast+1 (refined with SmithWaterman) on every orphaned gene against every other (both self and non-self species); 4) Large families that would be too complex to analyze are broken down with QuickTree7 to limit them to 1500 genes; 5) For each cluster (family), build multiple sequence alignments based on the protein sequences using either a combination of multiple aligners; 6) For each aligned cluster, build a phylogenetic tree using TreeBeST 5 using the CDS back-translation of the protein multiple alignments from the original DNA sequences. 7) infer pairwise gene relations of orthology and paralogy types from each gene tree.

Some genes identified from the above homologous search approach were not predicted as F-box domain-encoding genes. We used sequences alignment to study the possible mechanisms responsible for the absence of F-box domains from those F-box homologs.

### Reconstructing the phylogenetic tree of the F-box gene family

The phylogenetic history of the F-box gene family across and within the five *Caenorhabditis* species and *P. pacificus* were reconstructed using a maximum likelihood approach. First, amino acid sequences of F-box domain regions were extracted from the protein sequences. Second, Multiple sequence alignments (MSA) of the extracted amino acid sequences across or within each of the five *Caenorhabditis* species were generated using MUSCLE 3.52 [52], followed by removal gap columns using Gappyout implemented in trimAl software [53]. Third, the resulting alignments were used for gene tree inference by RAxML [54] using a PROTGAMMAVT model of evolution. Statistical support was obtained from 100 bootstrap replicates in RAxML. The online tool iTol [55] was used to display, manipulate, and annotate phylogenetic trees.

### F-box gene number variation and underlying mechanisms

In *Caenorhabditis*, a large number of F-box genes are conserved only at the F-box domain region. Therefore, a whole F-box gene sequence is inappropriate for constructing gene trees to infer gene number variation. A gene tree was constructed using F-box domain region sequences for each orthologous group in the present study. Next, we combined the gene tree with the species tree [56, 57] to infer gene number variation using NOTUNG [58]. Finally, we inferred the total variation for all F-box genes based on the inference mentioned earlier.

The DNA sequences of *C. elegans* and *C. briggsae* have been assembled into whole chromosomes. The R package RIdeogram was used to show gene density distribution on Chromosomes for F-box genes from *C. elegans* and *C. briggsae,* respectively [59]. Two genes were considered tandem duplications, given that there were no more than twenty genes between them [33]. For species with no assembled chromosomes, we treated a Contig as a chromosome, resulting in underestimates of tandem duplicates.

### Divergence of the gene structure of F-box paralogs

A phylogenetic tree was constructed for each identified F-box gene paralogous group. The closest two paralogs were compared for their difference in gene structure. Because of transcriptome sequencing data available for *C. elegans* and *C. briggsae*, the divergence mechanisms of F-box gene paralogs in the two species were studied. Each exon sequence was aligned with the sequence of the sibling using lfasta program [60]. Next, the similarity between the two compared sequences was shown in the graph, and the customized Perl scripts completed the whole process. A total of 99 and 37 siblings were aligned well in *C. elegans* and *C. briggsae,* respectively. The gene structural divergence mechanisms of these paralogs were then investigated.

DNA dot matrix analysis was performed on F-box gene sequences with itself using DNAMAN program version 6.0 (Lynnon Corporation, Pointe-Claire, Quebec, Canada) to find short tandem repeats. Dotplot was conducted with the following options: window size 30, minimum identity 60%.

### Functional divergence of duplicated F-box genes

F-box genes are a vastly expanded gene family, implying that these duplicates may have diverged in function. Thus, we studied the mechanism responsible for functional divergence of these identified F-box gene paralogs. Transcriptome profiles based on RNA-seq technology for *C. elegans* and *C. briggsae* were downloaded from modENCODE. Genome sequences and GTF files for *C. elegans* and *C. briggsae* were downloaded from the ENSEMBL database for RNA-seq data analysis.

Index files for the two genomes were generated using Bowtie2 [61]. RNA-seq reads were aligned with respective genomes using Tophat software [62], followed by assembling with Cufflinks [63]. Finally, differential expression analyses were performed using Cuffdiff [1]. We referred to program flow in literature [64]. Heatmap ideographs of gene expression differences were drawn with R package gplot from Bioconductor [65]. Development phase-specific expression of F-box paralogous group were calculated using mean deviation approach in R software.

### Detecting the selection pressure on F-box genes

In order to detect the selection pressure on the F-box genes, codons were extracted for each amino acid that was aligned between closely related paralogs using protein alignment as a guide, excluding regions containing gaps by using trimAl [53]. The synonymous substitution (dS), nonsynonymous substitution (dN), their ratio dN/dS, and the sliding window of duplicated genes were calculated using the DnaSP v5.0 program [66], following Nei and Gojobori method with the Jukes and Cantor correction [67, 68]. Sliding window options: window length = 45 bp; step size = 9 bp.

## Supplementary Information

**Additional file 1: Figure S1.** A workflow to identify F-box genes for the six nematode species. First, F-box profile HMM (PF00646) and PROSITE motif (PS50181) were used as a pattern to search for F-box proteins in the proteome sequences by Hmmersearch and ps_san.pl program, respectively. Second, the putative F-box proteins were used as a PSI-BLAST (e-value = 1e-30) search query against proteome sequences. Third, all of the putative F-box proteins were scanned for the F-box domain using modified multi-thread iprscan_lwd.pl program; Finally, pairwise alignment comparisons of the built profile HMM for each species with identified F-box profile HMM were visualized manually.

**Additional file 2: Figure S2.** Pairwise alignments of HMM Logos of F-box proteins. The overall height of the letter stacks represents the relative entropy of the distribution of the emission probabilities within some state relative to the background distribution given for the complete profile. The relative height of a letter corresponds to its emission probability from a state’s distribution. The column width denotes the relative contribution of the position to the overall protein family. Insert states are drawn in red. The aligned states in each HMM are framed and connected by a line. The numbers above and below each Logo show state positions in the HMM. a. Alignments of *C. elegans*-specific F-box HMM with the HMM of PF00646 from the PFAM database. b. Alignments of *C. briggsae*-specific F-box HMM with the HMM of PF00646 from the PFAM database. c. Alignments of *C. brenneri*-specific F-box HMM with the HMM of PF00646 from the PFAM database. d. Alignments of *C. remanei*-specific F-box HMM with the HMM of PF00646 from the PFAM database. e. Alignments of *C. japonica*-specific F-box HMM with the HMM of PF00646 from the PFAM database. f. Alignments of *P. pacificus*-specific F-box HMM with the HMM of PF00646 from the PFAM database.

**Additional file 3: Figure S3.** The number of genes with and without F-box domains in each F-box Paragroup in six *Caenorhabditis* species. The Y-axis is the number of paralogous genes, while the X-axis represents the sequence number. The blue and gray boxes indicate the FBOX and Non-FBOX genes, respectively. Non-FBOX genes deleted the F-box domain in each paragroup were identified as putative paralogs of F-BOX genes by the ENSEMBL database.

**Additional file 4: Figure S4.** The number of genes with and without F-box domains in each F-box orthogroup. The Y-axis is the number of orthologous genes in each orthogroup, while the X-axis represents the sequence number. The blue and gray boxes indicate the FBOX and Non-FBOX genes, respectively. Non-FBOX genes deleted the F-box domain and were identified as a putative orthologous counterpart in six *Caenorhabditis* species by the ENSEMBL database.

**Additional file 5: Figure S5.** The entirely phylogenetic relationships of F-box proteins from *C. brenneri*, *C. briggsae*, *C. elegans*, *C. japonica*, and *C. remanei* and *P. pacificus* being color-coded light sea green, chocolate, purple, pink, lime-green, and orange, respectively. F-box domain sequences were aligned using MUSCLE. The topology was generated by maximum likelihood analysis using RAxML. Statistical support was obtained by 100-bootstrap RAxML replicates, and schematic triangles denote bootstrap values greater than 50.

**Additional file 6: Figure S6.** Phylogenetic tree of F-box domain sequences from each of the six species. The topology was generated by maximum likelihood analysis using RAxML. 100-bootstrap RAxML replicates obtained statistical support, and schematic triangles indicate bootstrap values greater than 50. Different colored boxes indicate f-box genes from different clades.

**Additional file 7: Figure S7.** Chromosomal distribution of genome-wide identified F-box genes. Chromosome numbers are denoted at the bottom of each bar, and its relative length indicates chromosome size. Gene density is indicated according to the heatmap in the legend at a 1-Mb window scale. a. The gene density of identified F-box genes across the entire genome of *C. elegans.* Of 377 identified F-box genes, 269 were distributed in 21 gene clusters with five or more F-box genes. Notably, one gene cluster located on Chromosome II consists of 42 F-box genes, and the other one is located on Chromosome III, which includes 32 F-box genes. b. The gene density of identified F-box genes across the entire genome of *C. briggsae.* ‘un’ indicates an unknown chromosome. Of 192 identified F-box genes, 76 were distributed in 7 gene clusters with five or more F-box genes. Of note, two neighboring gene clusters on Chromosome V consist of 21 and 18 F-box genes.

**Additional file 8: Figure S8.** Evolutionary diverged Exon-intron structure of representative sibling paralogs in *C. elegans.* Ninety-nine sibling paralogs were compared for their exon-intron structure divergence. The color scale shown at the top of the schematic diagram represents the sequence similarity of the aligned homologous region. The numbers above and below each exon/intron denote the nucleotide length of alignments.

**Additional file 9: Figure S9.** Evolutionary diverged Exon-intron structure of representative sibling paralogs in *C. briggsae.* Thirty-seven sibling paralogs were compared for their exon-intron structure divergence. The color scale shown at the top of the schematic diagram represents the sequence similarity of the aligned homologous region) is shown at the top. The numbers above and below each exon/intron show the nucleotide length of alignments.

**Additional file 10: Figure S10.** Sliding-window plots of dS, dN, and dN/dS in pairwise comparisons of 95 closely related paralogs of F-box genes from *C. elegans*. The window size is 45 codons, and the offset between windows is nine codons. The solid line represents plots of dN/dS, the short-dotted line indicates plots of dS, and the long-dotted line indicates plots of dN.

**Additional file 11: Figure S11.** Sliding-window plots of dS, dN, and dN/dS in pairwise comparisons of 37 closely related paralogs of F-box genes from *C. briggsae*. The window size is 45 codons, and the offset between windows is nine codons. The solid line represents plots of dN/dS, the short-dotted line indicates plots of dS, and the long-dotted line indicates plots of dN.

**Additional file 12 **Supplementary Datasets (S1-S6) provide the identified F-box protein sequences for *C. brenneri*, *C. briggsae*, *C. elegans*, *C. japonica*, *C. remanei,* and *P. pacificus*, respectively.

**Additional file 13: Supplementary File 1.** shows the analysis results of gene structural and functional diversification and selection pressure of closely related paralogs from *C.elegans* and *C.briggsae,* respectively.

## Data Availability

The proteomic and cDNA sequences for six species (*C. brenneri*, *C. briggsae*, *C. elegans*, *C. japonica*, *C. remanei*, and *P. pacificus*) and the GTF file for *C. brenneri* and *C. briggsae* were available in EnsembleMetazoa database with release version 51 (https://metazoa.ensembl.org/info/data/ftp/index.html). Expression data for *C. elegans* was downloaded from modENCODE database (http://data.modencode.org/cgi-bin/findFiles.cgi?download=6532,3974,3975,4493,4547,4548,3977,3978,4544,4579,4580,3882,4006,4007,4527,4529,4574,4038,4039,4530,4575,4041,4532,3879,4044,4045,4055,4173,4534,4535,4577,4578,4594). Expression data for *C. briggsae* was downloaded from the modENCODE database (http://data.modencode.org/cgi-bin/findFiles.cgi?download=6528,6529,6530,6532). All other data generated during this study are included in this article and its Additional files. The Hidden Markov model of the F-box domain was downloaded from the PFAM database (http://pfam.xfam.org/family/f-box#tabview=tab6). The Prosite file of the F-box domain was downloaded from the PROSITE database (ftp://ftp.expasy.org/databases/prosite/).

## Abbreviations

UPS: Ubiquitin-proteasome system
*C. brenneri*: *Caenorhabditis* brenneri
*C. briggsae*: *Caenorhabditis briggsae*
*C. elegans*: *Caenorhabditis elegans*
*C. japonica*: *Caenorhabditis* japonica
*C. remanei*: *Caenorhabditis* remanei
SCF: SKP1–CUL1–F-box protein
FBKs: F-box Kelch genes
*P. pacificus*: Pristionchus pacificus
paragroup: Paralogous group
orthogroup: Orthologous group
