# Supplementary figures and images for: Genome-wide characterization, evolution, structure, and expression analysis of the F-box genes in Caenorhabditis
Source: BMC Genomics. 2021 Dec 11;22:889. doi: 10.1186/s12864-021-08189-7 (PMC8665587; doi:10.1186/s12864-021-08189-7)

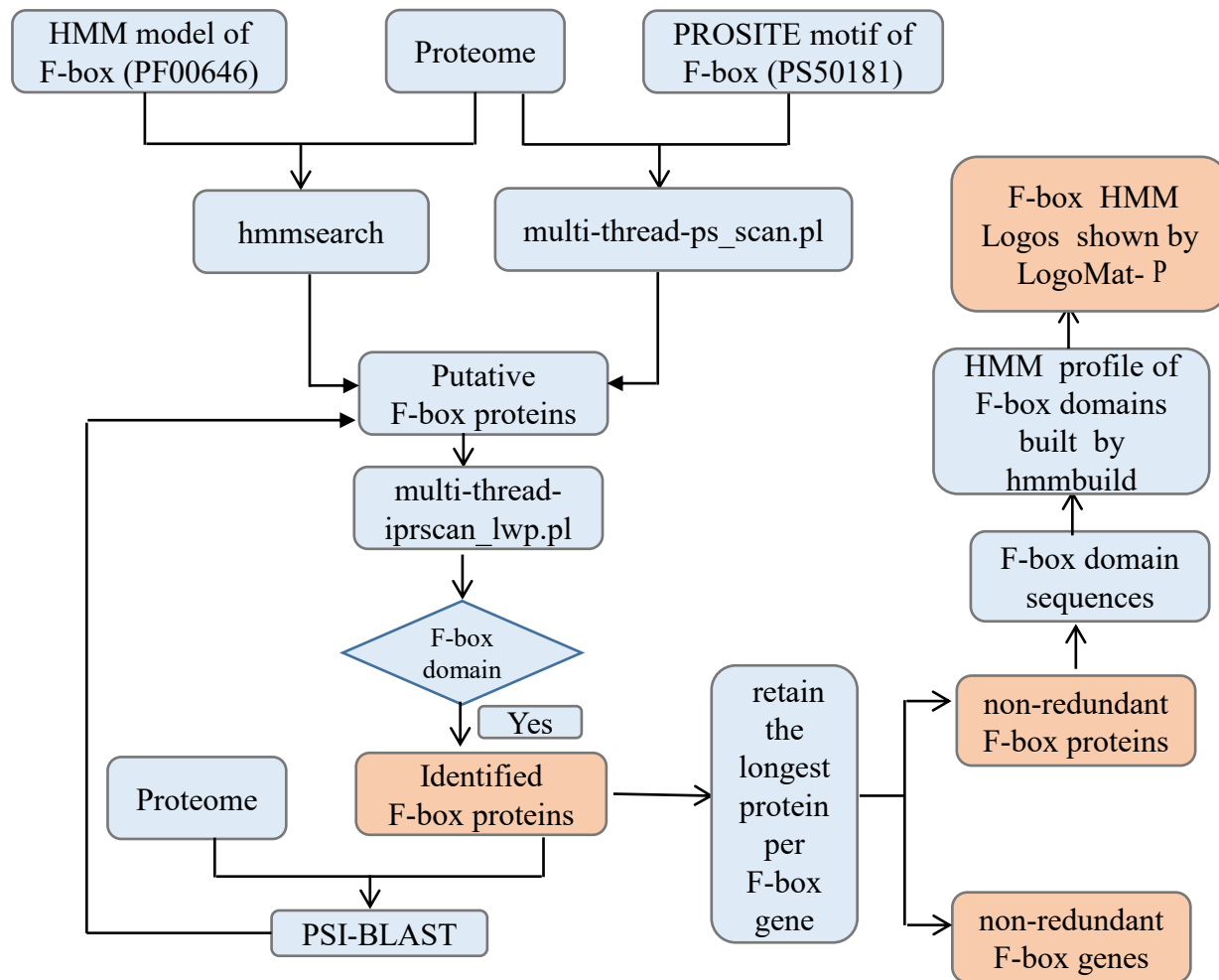

Supplement: Supplementary file 1 — Additional file 1: Figure S1. A workflow to identify F-box genes for the six nematode species. First, F-box profile HMM (PF00646) and PROSITE motif (PS50181) were used as a pattern to search for F-box proteins in the proteome sequences by Hmmersearch and ps_san.pl program, respectively. Second, the putative F-box proteins were used as a PSI-BLAST (e-value = 1e-30) search query against proteome sequences. Third, all of the putative F-box proteins were scanned for the F-box domain using modified multi-thread iprscan_lwd.pl program; Finally, pairwise alignment comparisons of the built profile HMM for each species with identified F-box profile HMM were visualized manually. [file 12864_2021_8189_MOESM1_ESM.pdf]

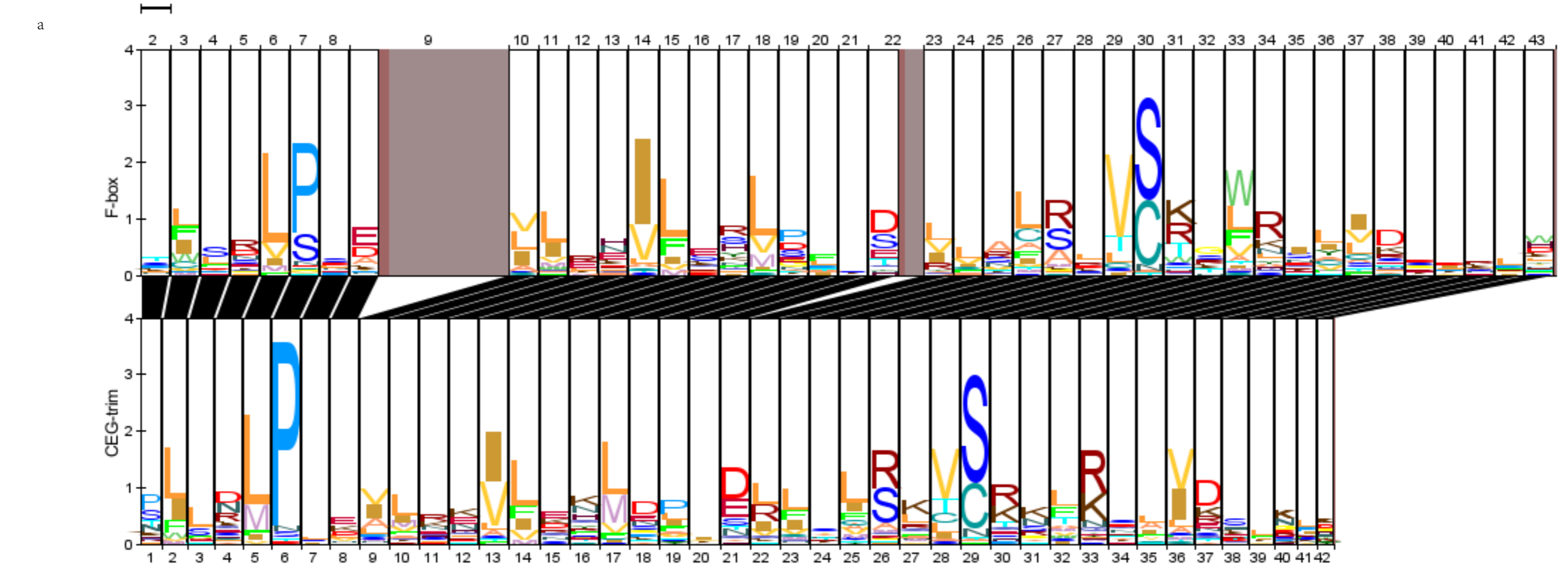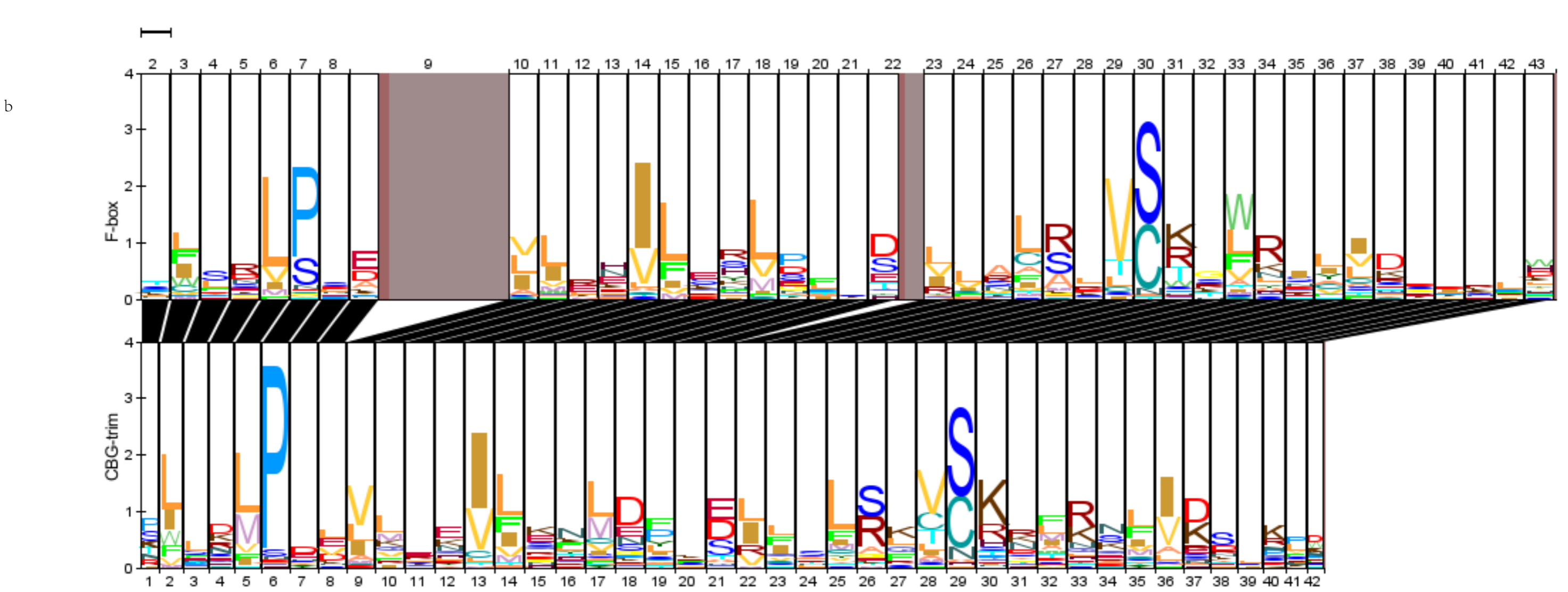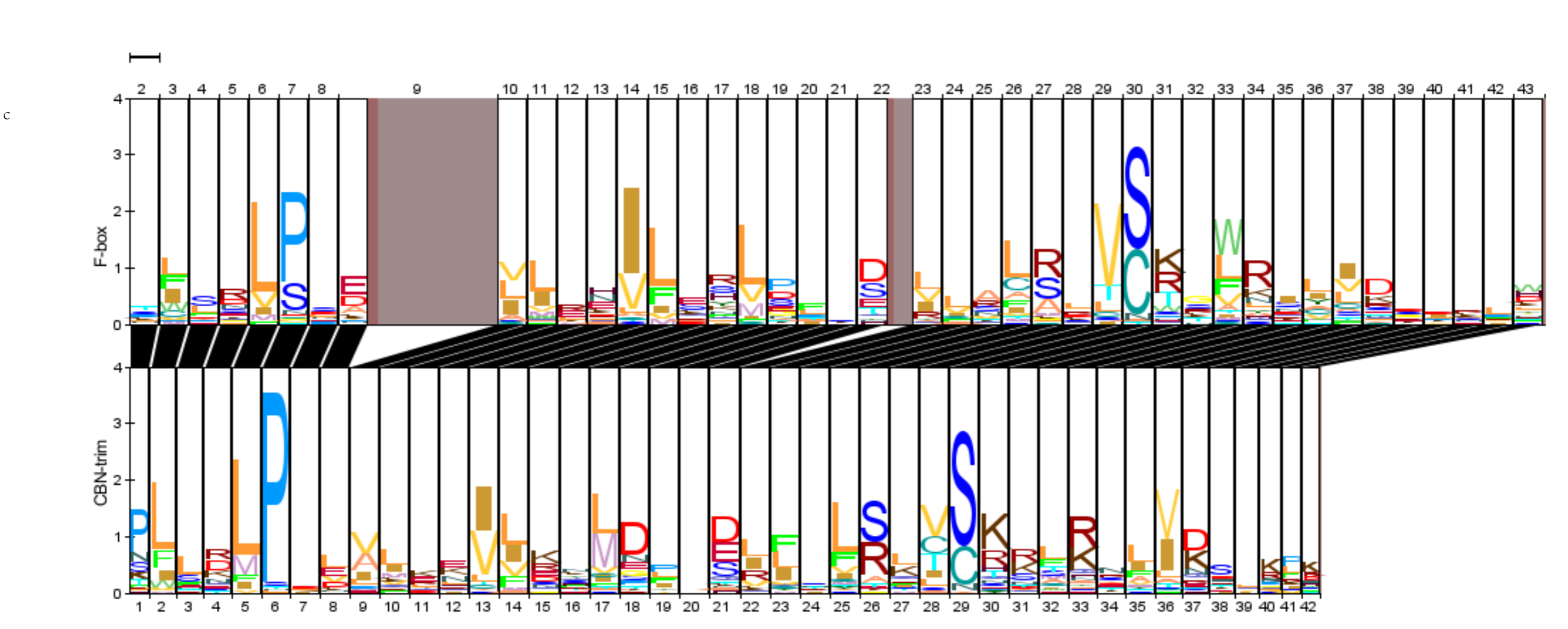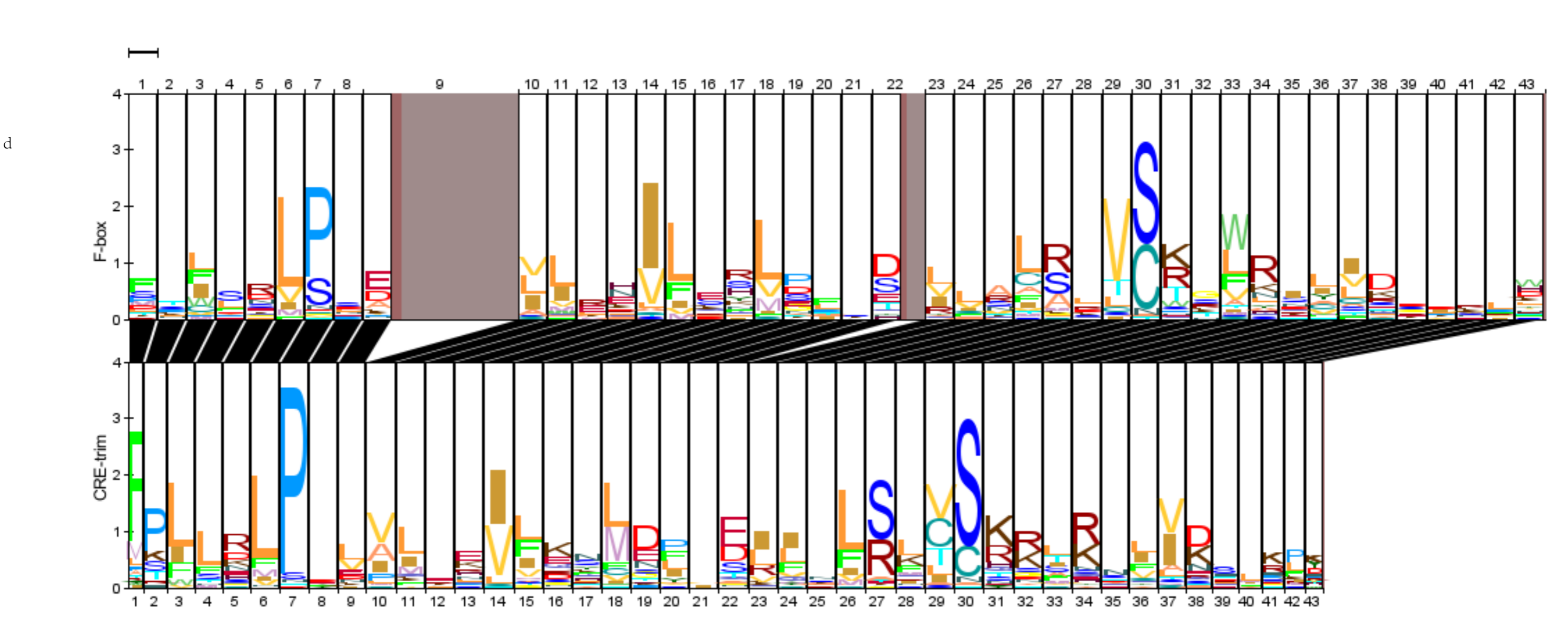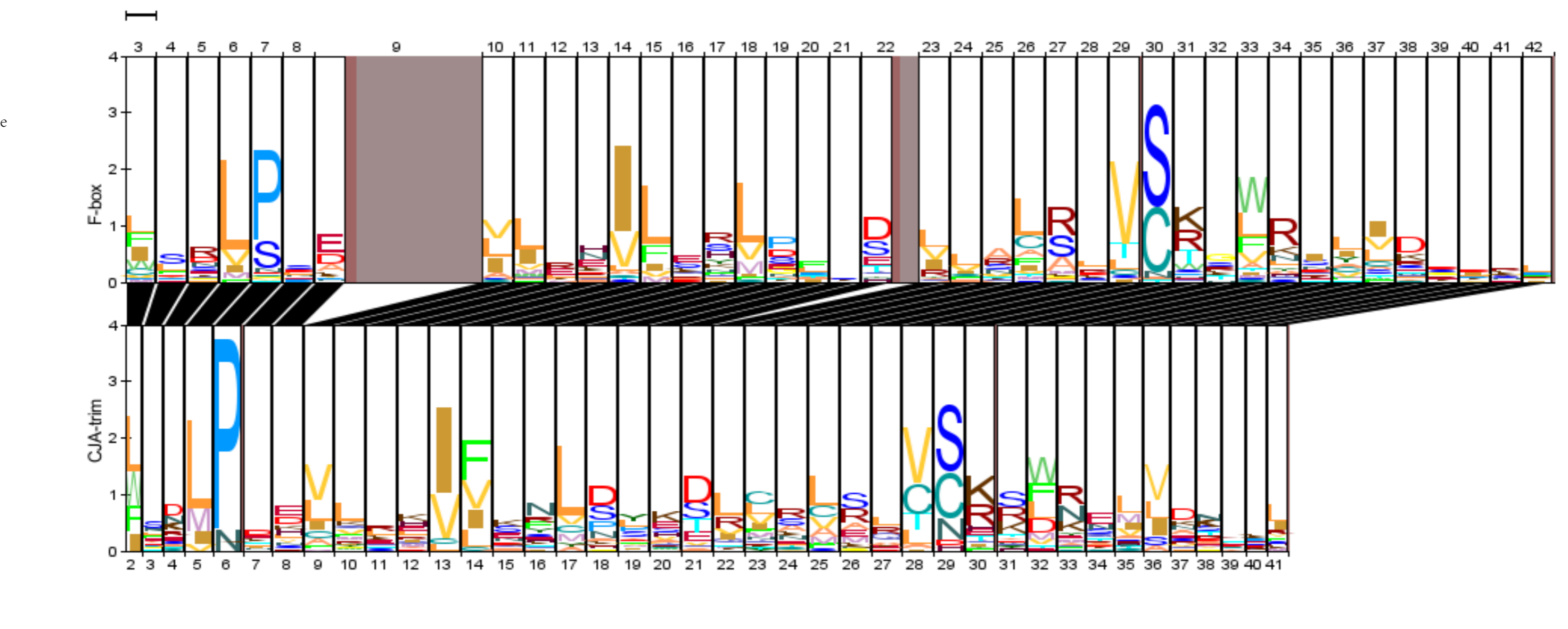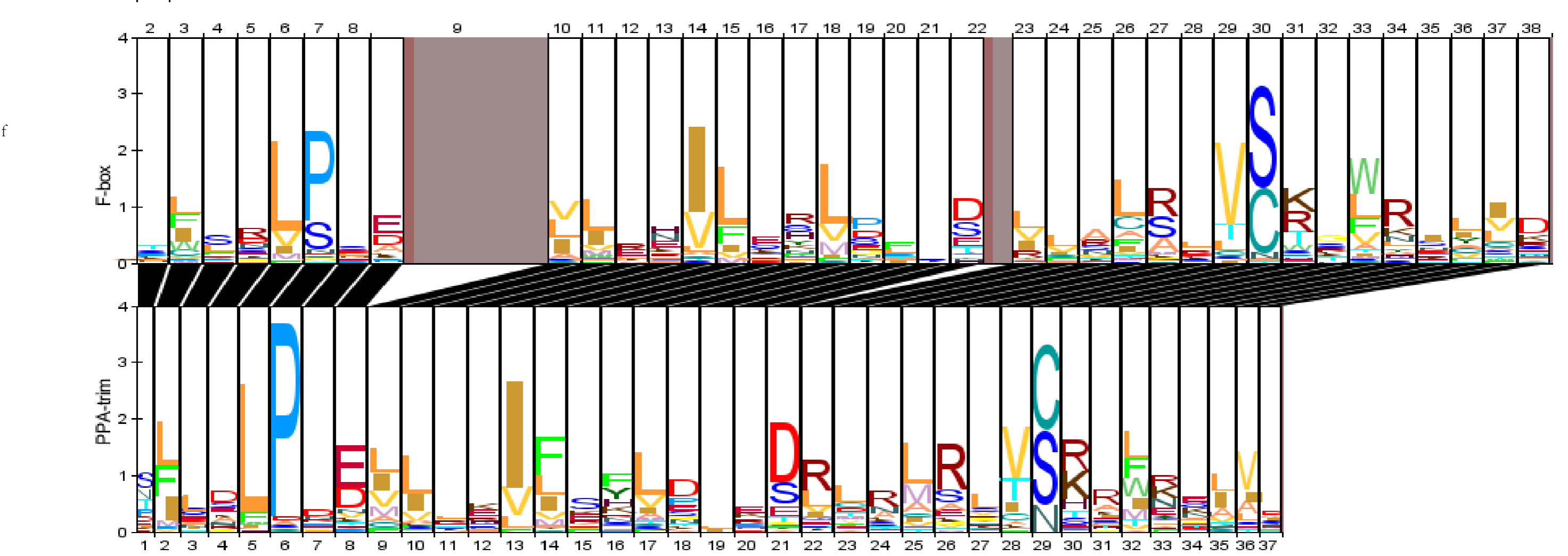

Supplement: Supplementary file 2 — Additional file 2: Figure S2. Pairwise alignments of HMM Logos of F-box proteins. The overall height of the letter stacks represents the relative entropy of the distribution of the emission probabilities within some state relative to the background distribution given for the complete profile. The relative height of a letter corresponds to its emission probability from a state’s distribution. The column width denotes the relative contribution of the position to the overall protein family. Insert states are drawn in red. The aligned states in each HMM are framed and connected by a line. The numbers above and below each Logo show state positions in the HMM. a. Alignments of C. elegans-specific F-box HMM with the HMM of PF00646 from the PFAM database. b. Alignments of C. briggsae-specific F-box HMM with the HMM of PF00646 from the PFAM database. c. Alignments of C. brenneri-specific F-box HMM with the HMM of PF00646 from the PFAM database. d. Alignments of C. remanei-specific F-box HMM with the HMM of PF00646 from the PFAM database. e. Alignments of C. japonica-specific F-box HMM with the HMM of PF00646 from the PFAM database. f. Alignments of P. pacificus-specific F-box HMM with the HMM of PF00646 from the PFAM database. [file 12864_2021_8189_MOESM2_ESM.pdf]

### Pristionchus pacificus

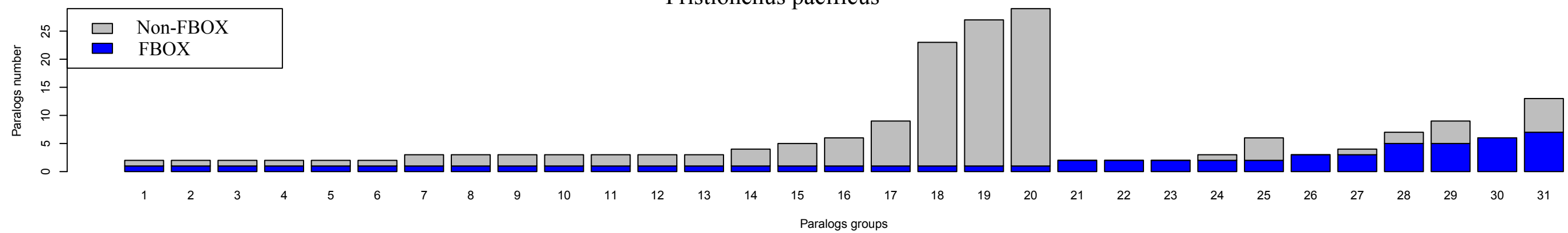

### C.japonica

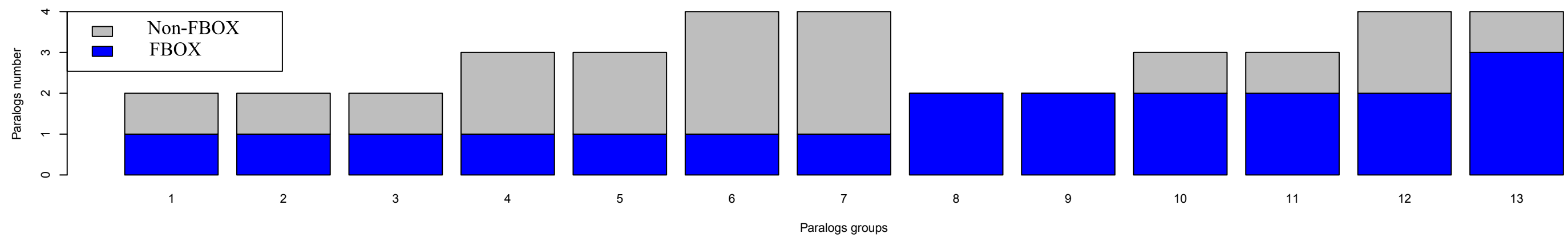

### C.elegans

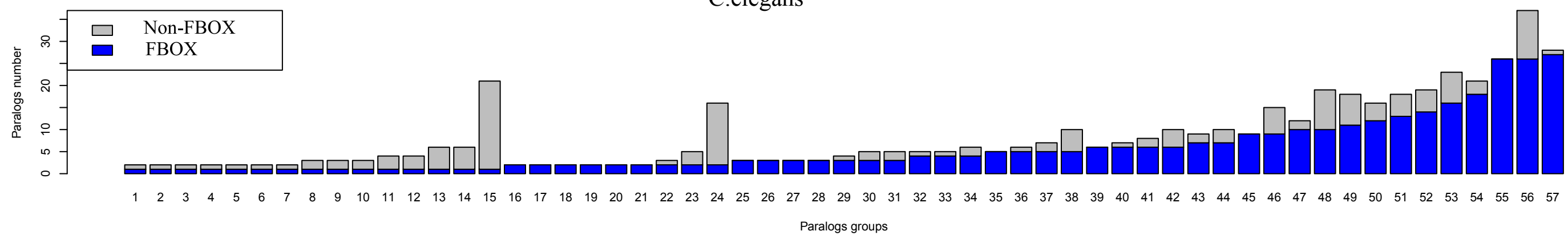

### C.brenneri

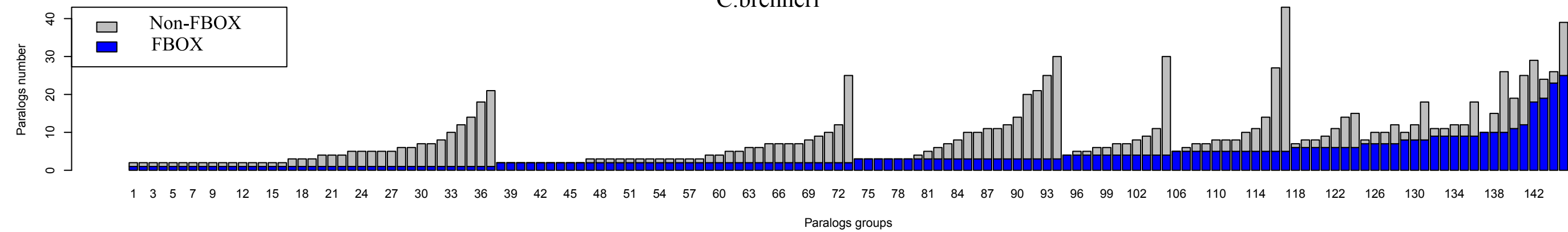

### C.remanei

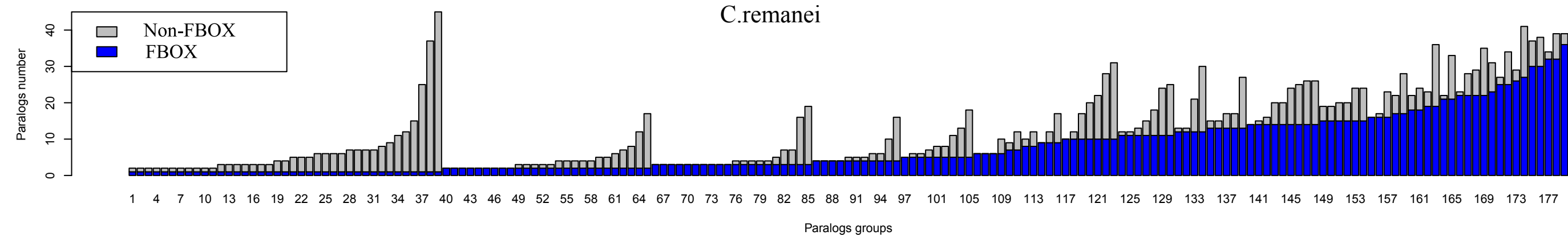

### C.briggsae

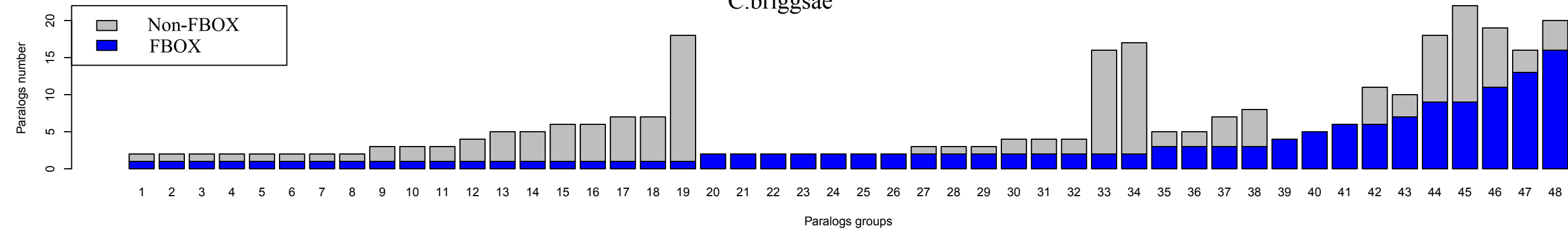

Supplement: Supplementary file 3 — Additional file 3: Figure S3. The number of genes with and without F-box domains in each F-box Paragroup in six Caenorhabditis species. The Y-axis is the number of paralogous genes, while the X-axis represents the sequence number. The blue and gray boxes indicate the FBOX and Non-FBOX genes, respectively. Non-FBOX genes deleted the F-box domain in each paragroup were identified as putative paralogs of F-BOX genes by the ENSEMBL database. [file 12864_2021_8189_MOESM3_ESM.pdf]

F-box and Non\_Fbox genes in Orthogroups

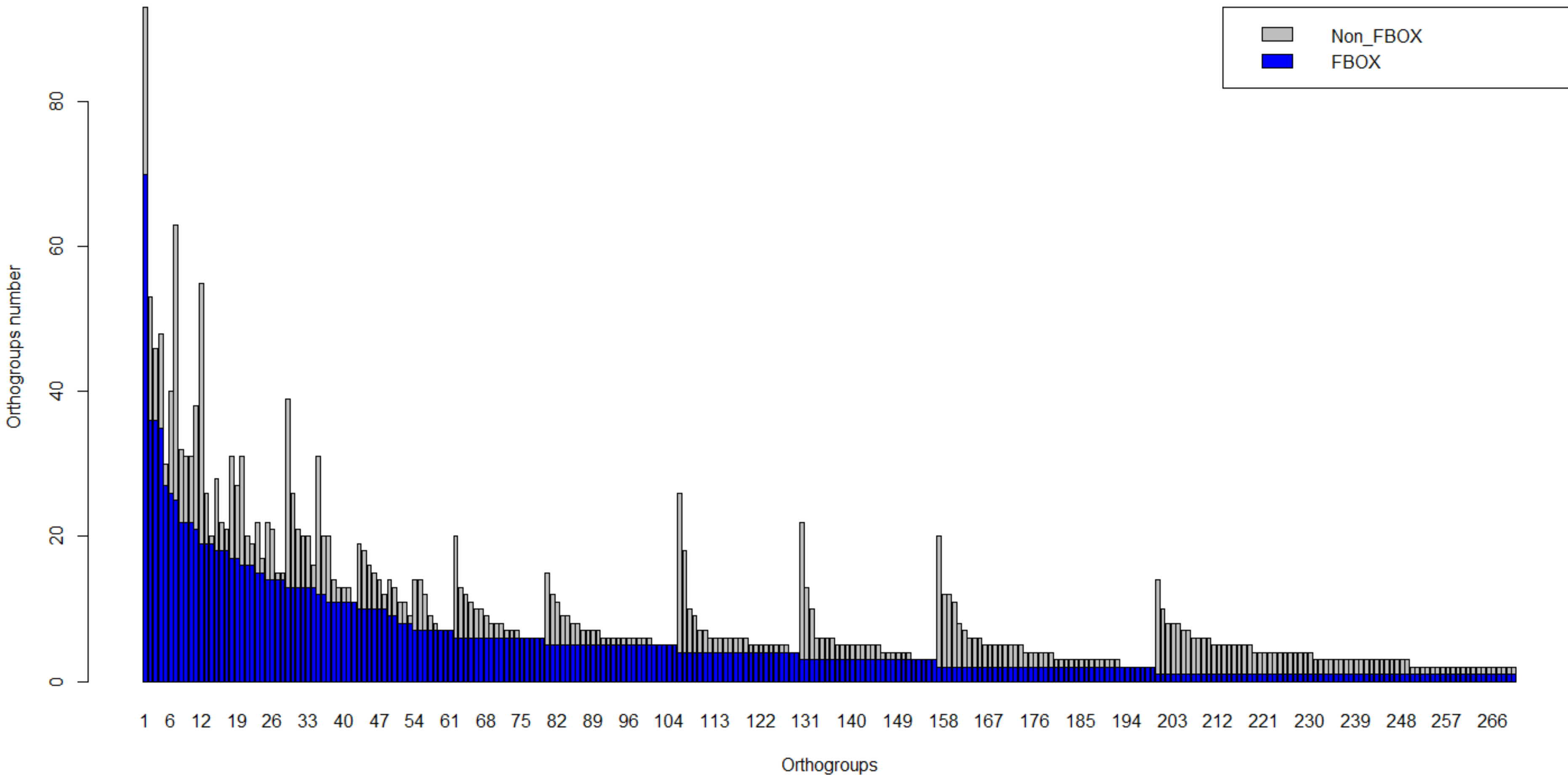

Supplement: Supplementary file 4 — Additional file 4: Figure S4. The number of genes with and without F-box domains in each F-box orthogroup. The Y-axis is the number of orthologous genes in each orthogroup, while the X-axis represents the sequence number. The blue and gray boxes indicate the FBOX and Non-FBOX genes, respectively. Non-FBOX genes deleted the F-box domain and were identified as a putative orthologous counterpart in six Caenorhabditis species by the ENSEMBL database. [file 12864_2021_8189_MOESM4_ESM.pdf]

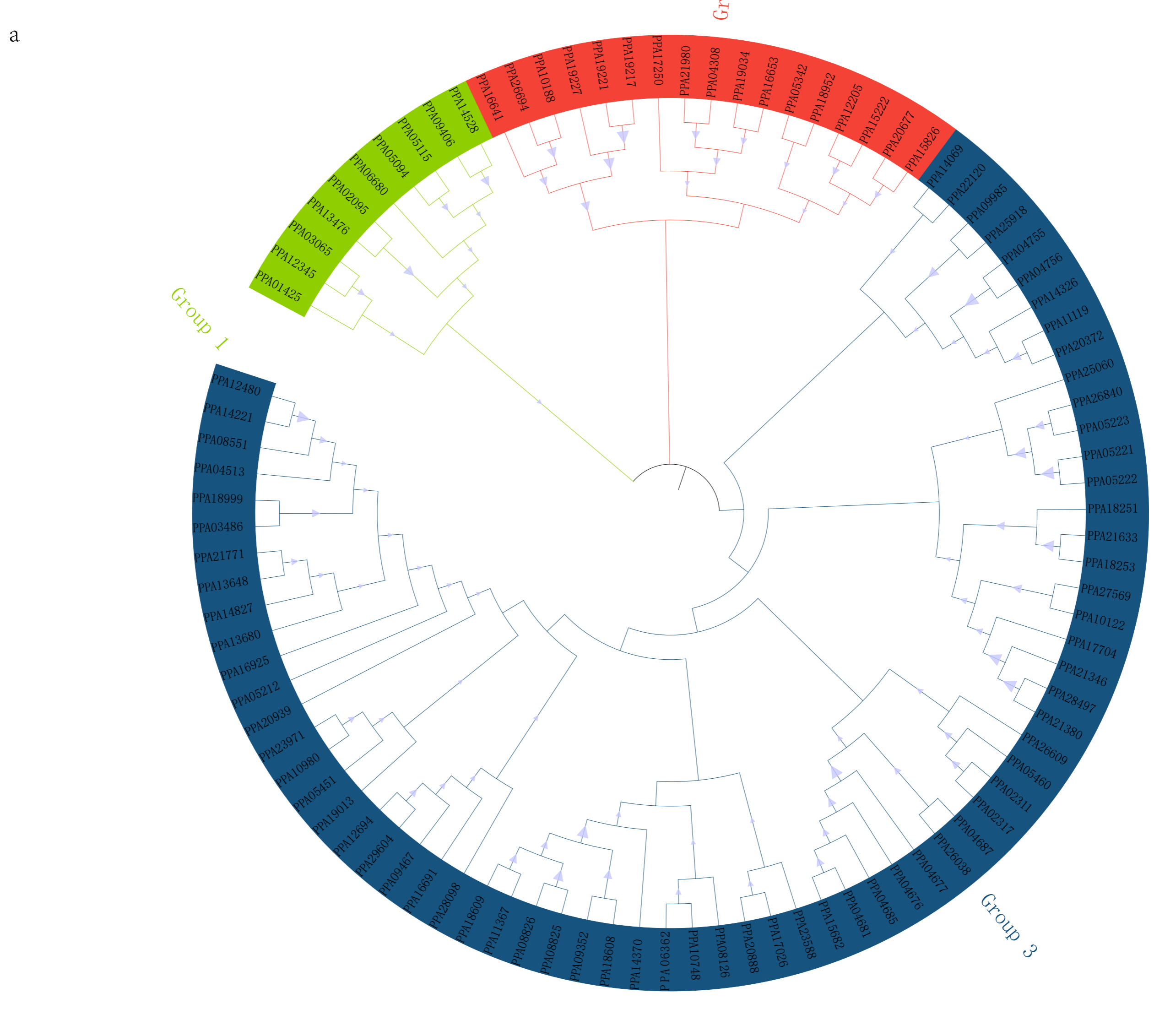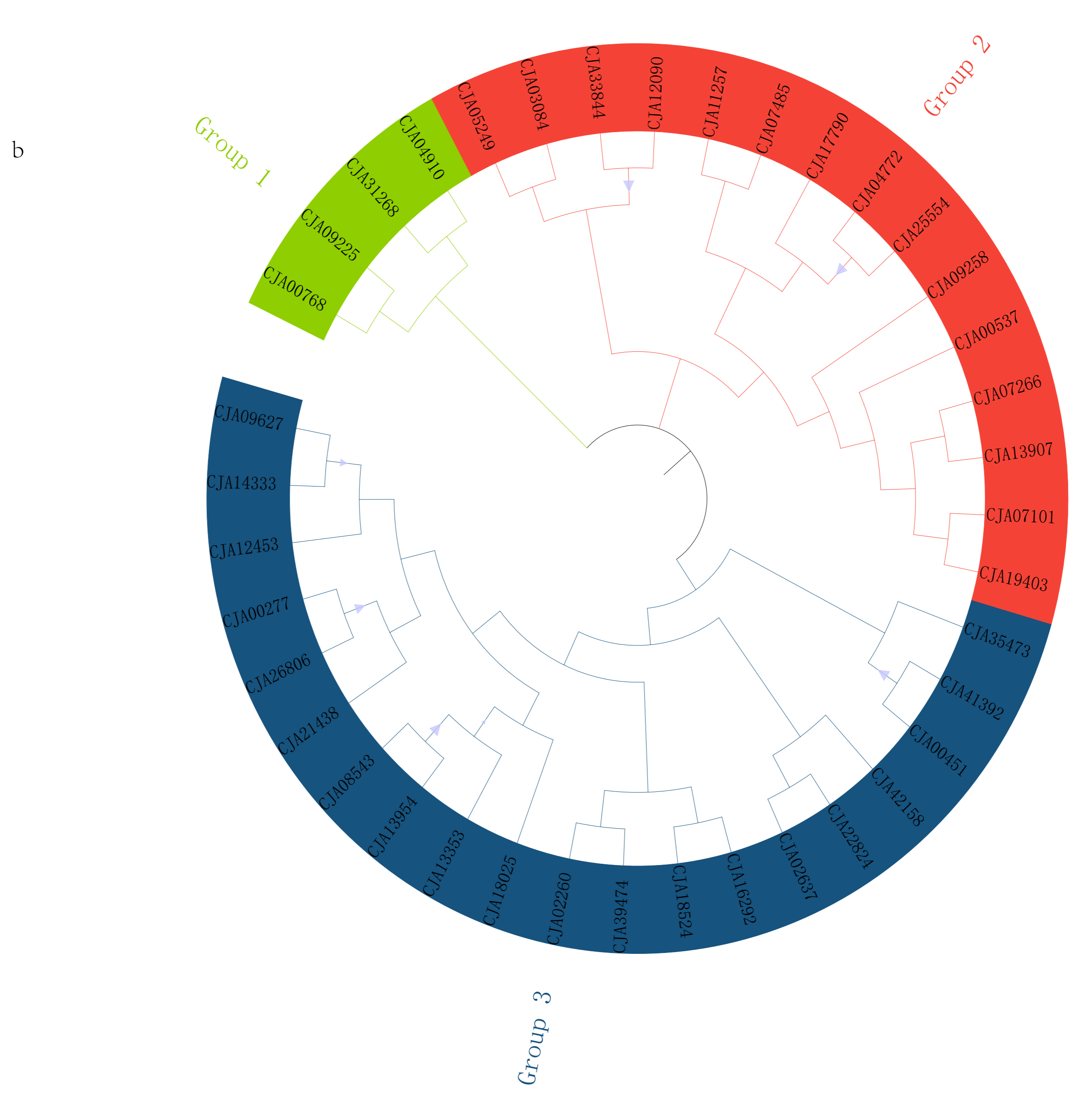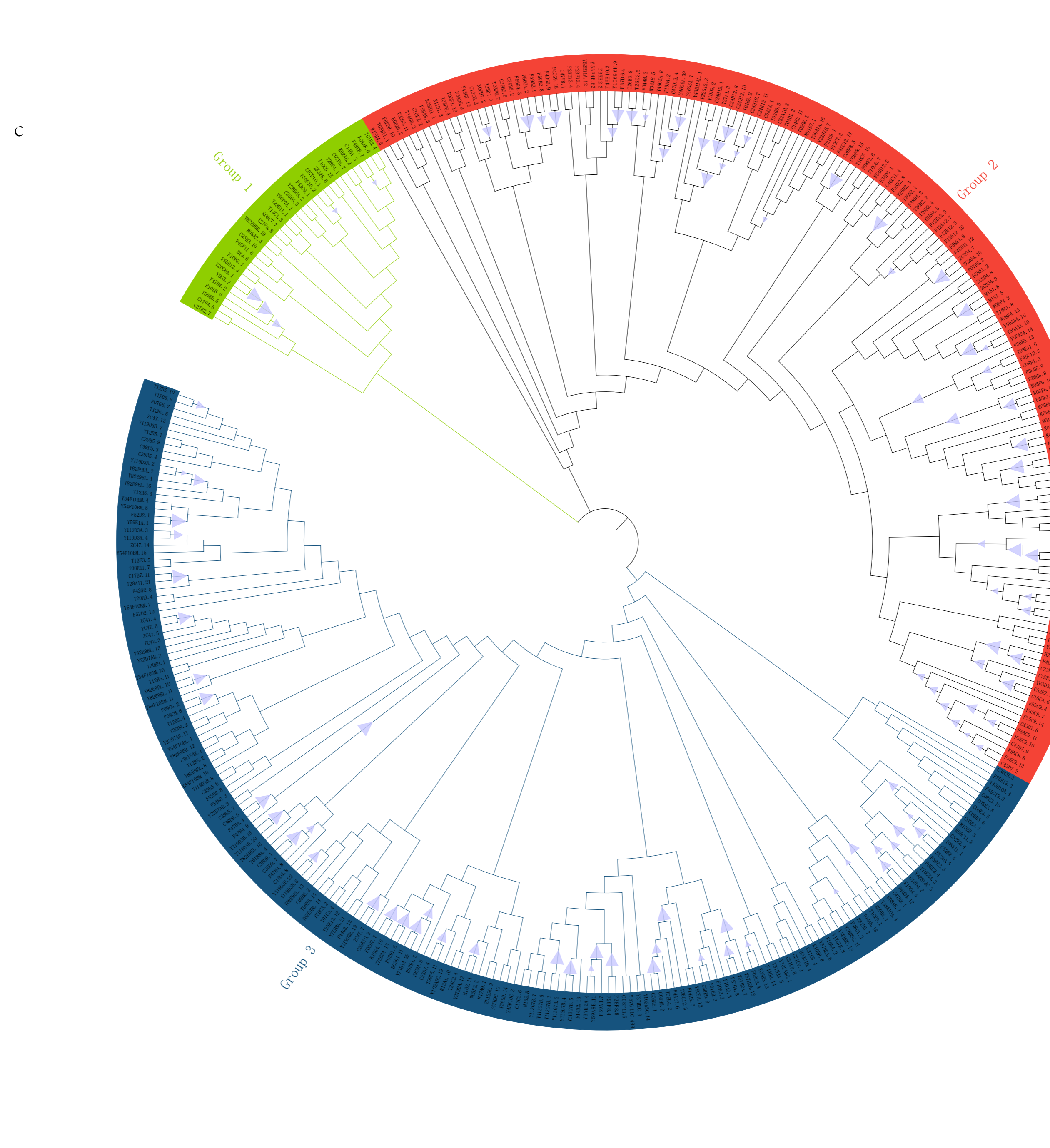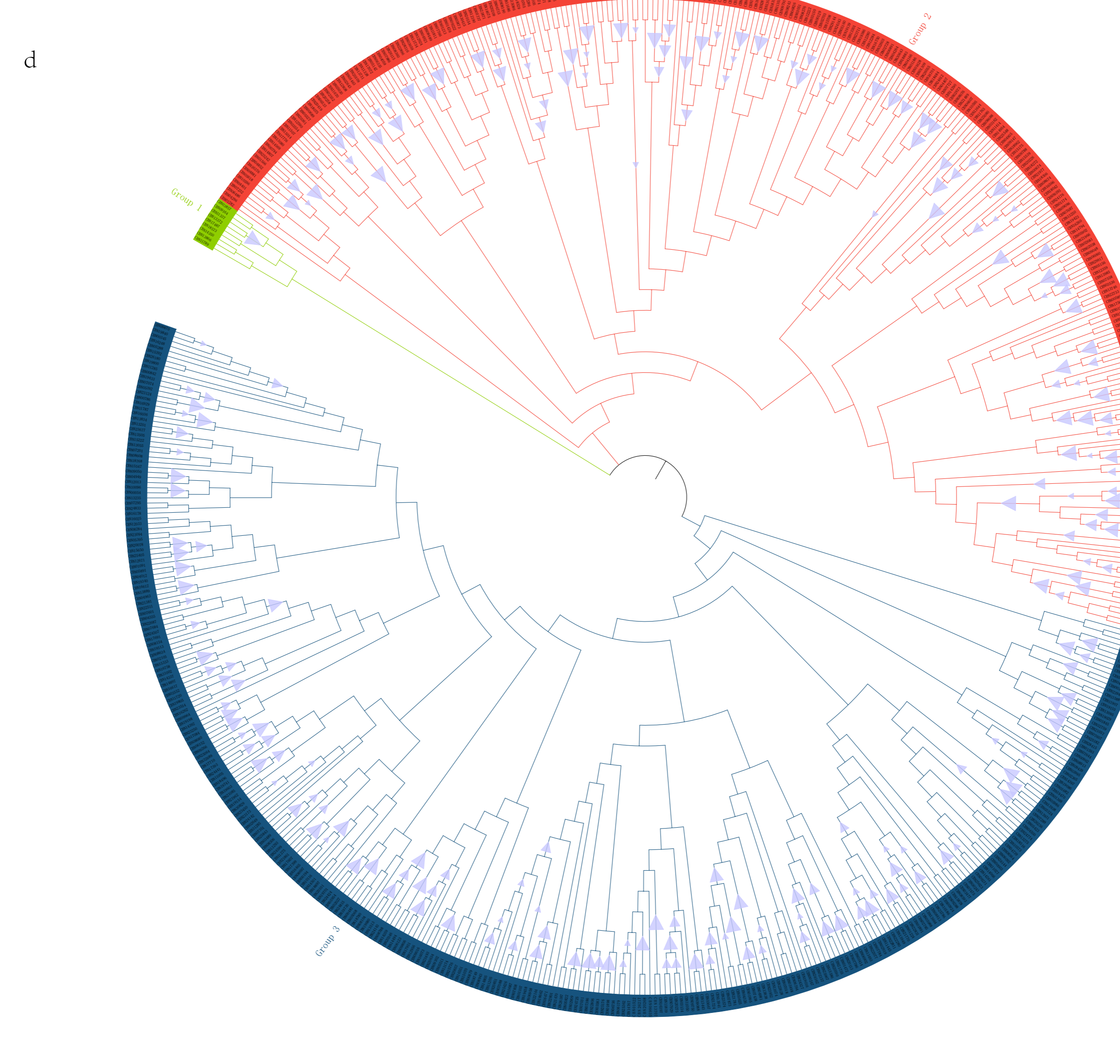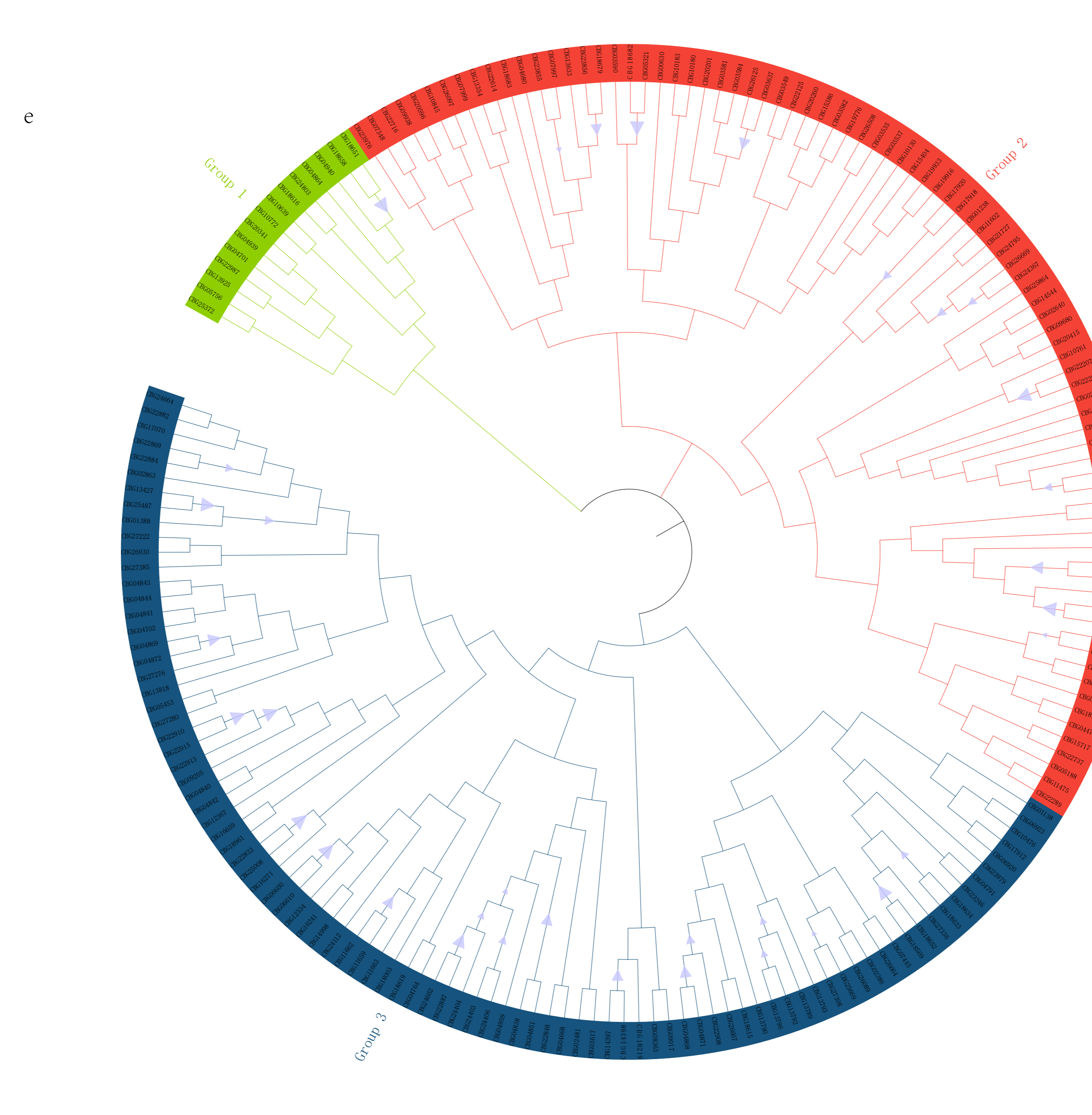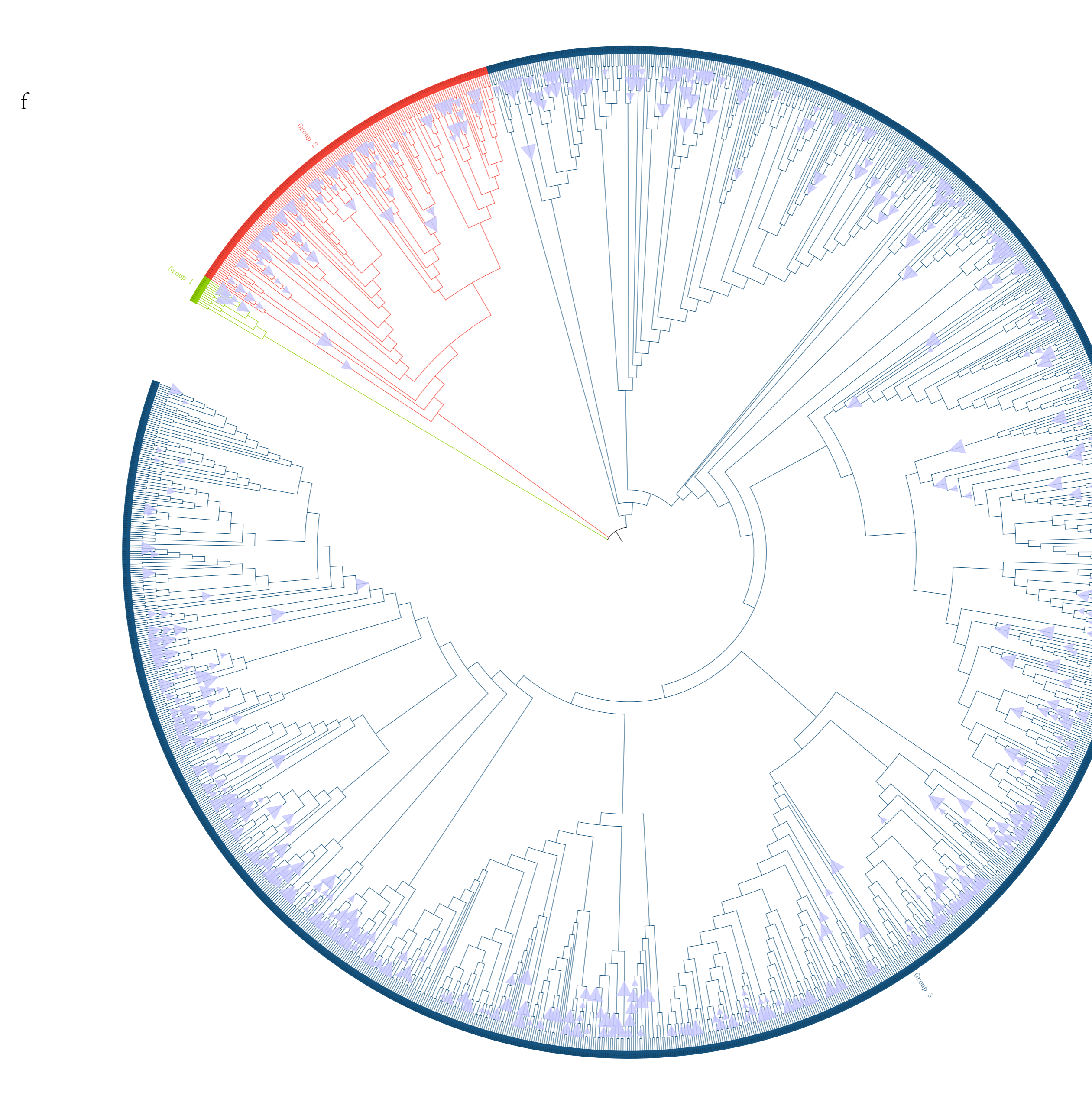

Supplement: Supplementary file 6 — Additional file 6: Figure S6. Phylogenetic tree of F-box domain sequences from each of the six species. The topology was generated by maximum likelihood analysis using RAxML. 100-bootstrap RAxML replicates obtained statistical support, and schematic triangles indicate bootstrap values greater than 50. Different colored boxes indicate f-box genes from different clades. [file 12864_2021_8189_MOESM6_ESM.pdf]

a

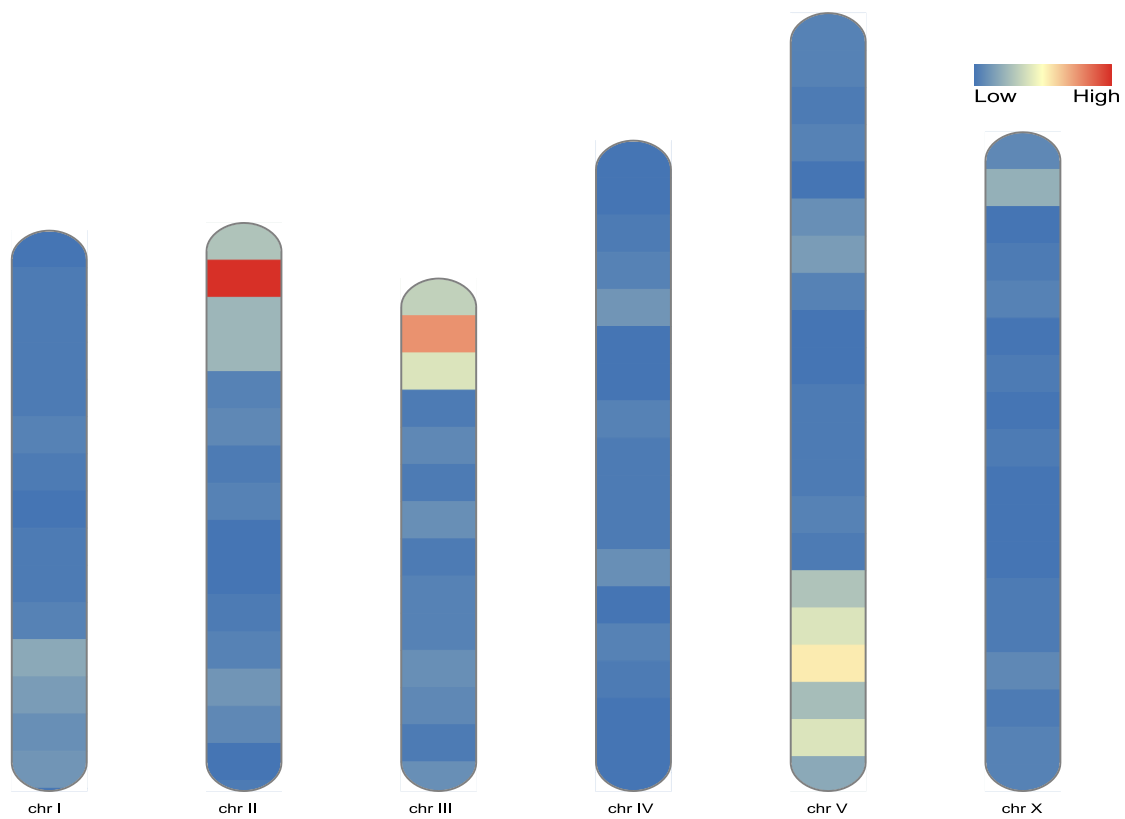

b

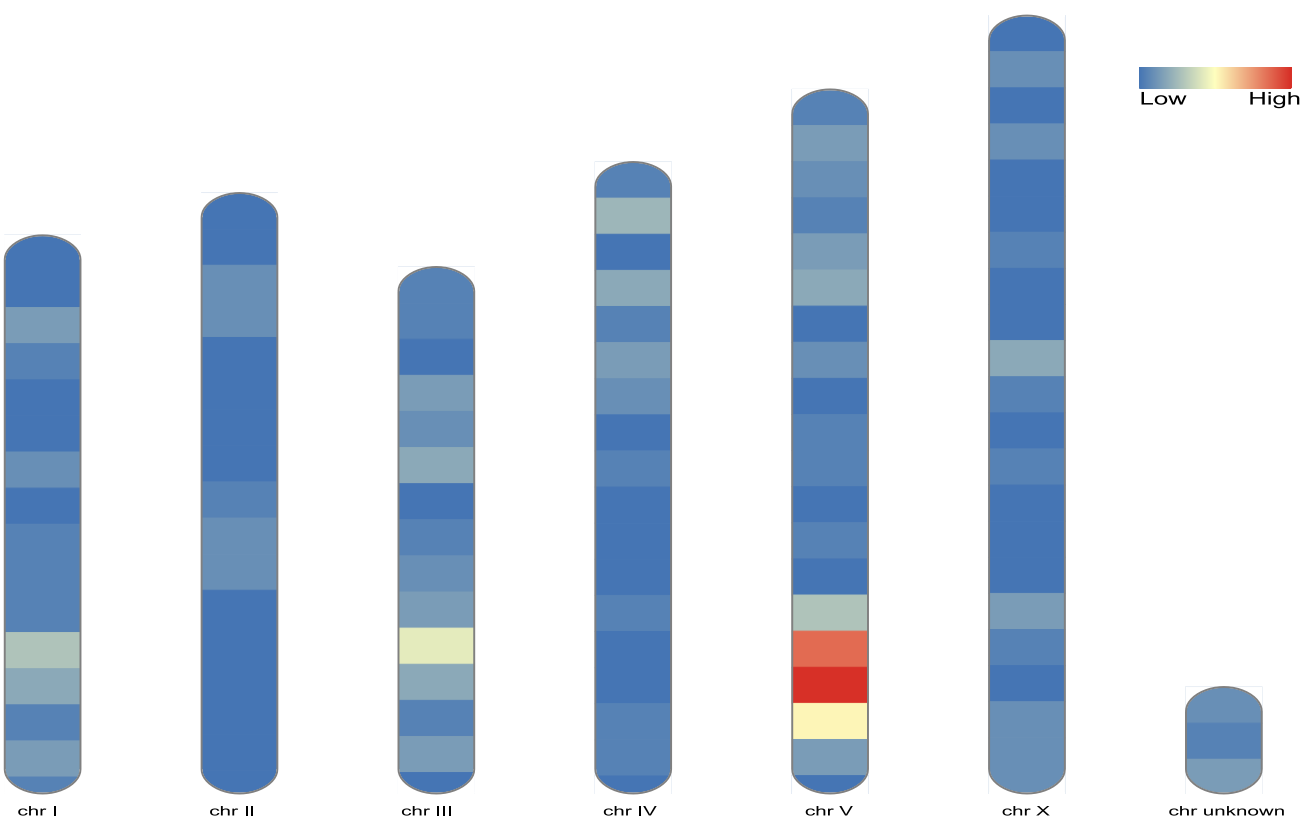

Supplement: Supplementary file 7 — Additional file 7: Figure S7. Chromosomal distribution of genome-wide identified F-box genes. Chromosome numbers are denoted at the bottom of each bar, and its relative length indicates chromosome size. Gene density is indicated according to the heatmap in the legend at a 1-Mb window scale. a. The gene density of identified F-box genes across the entire genome of C. elegans. Of 377 identified F-box genes, 269 were distributed in 21 gene clusters with five or more F-box genes. Notably, one gene cluster located on Chromosome II consists of 42 F-box genes, and the other one is located on Chromosome III, which includes 32 F-box genes. b. The gene density of identified F-box genes across the entire genome of C. briggsae. ‘un’ indicates an unknown chromosome. Of 192 identified F-box genes, 76 were distributed in 7 gene clusters with five or more F-box genes. Of note, two neighboring gene clusters on Chromosome V consist of 21 and 18 F-box genes. [file 12864_2021_8189_MOESM7_ESM.pdf]

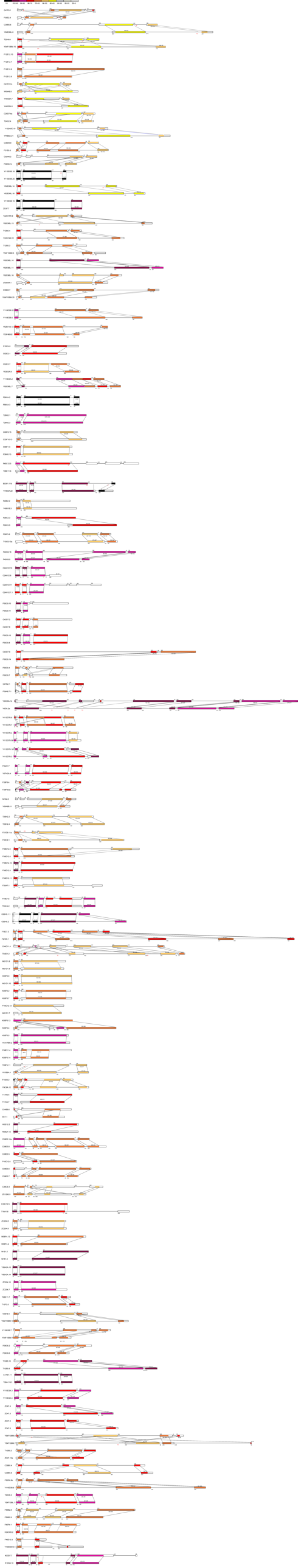

Supplement: Supplementary file 8 — Additional file 8: Figure S8. Evolutionary diverged Exon-intron structure of representative sibling paralogs in C. elegans. Ninety-nine sibling paralogs were compared for their exon-intron structure divergence. The color scale shown at the top of the schematic diagram represents the sequence similarity of the aligned homologous region. The numbers above and below each exon/intron denote the nucleotide length of alignments. [file 12864_2021_8189_MOESM8_ESM.pdf]

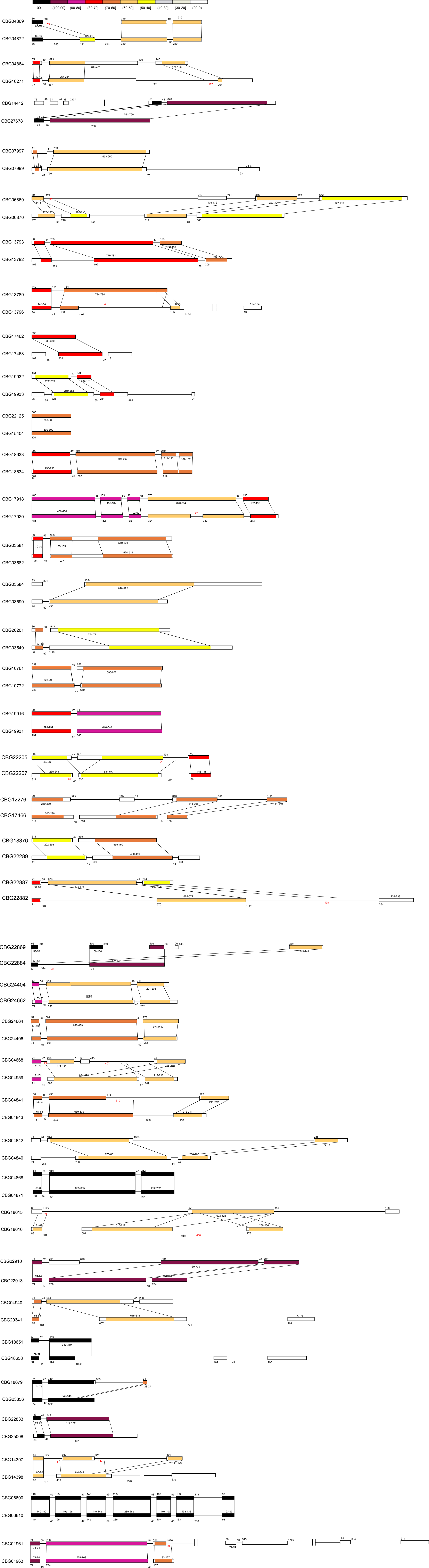

Supplement: Supplementary file 9 — Additional file 9: Figure S9. Evolutionary diverged Exon-intron structure of representative sibling paralogs in C. briggsae. Thirty-seven sibling paralogs were compared for their exon-intron structure divergence. The color scale shown at the top of the schematic diagram represents the sequence similarity of the aligned homologous region) is shown at the top. The numbers above and below each exon/intron show the nucleotide length of alignments. [file 12864_2021_8189_MOESM9_ESM.pdf]
