# Supplementary material for: Genome-wide characterization, evolution, structure, and expression analysis of the F-box genes in Caenorhabditis
Source: BMC Genomics. 2021 Dec 11;22:889. doi: 10.1186/s12864-021-08189-7 (PMC8665587; doi:10.1186/s12864-021-08189-7)
Supplement: Supplementary file 10 — Additional file 10: Figure S10. Sliding-window plots of dS, dN, and dN/dS in pairwise comparisons of 95 closely related paralogs of F-box genes from C. elegans. The window size is 45 codons, and the offset between windows is nine codons. The solid line represents plots of dN/dS, the short-dotted line indicates plots of dS, and the long-dotted line indicates plots of dN. [file 12864_2021_8189_MOESM10_ESM.pdf]

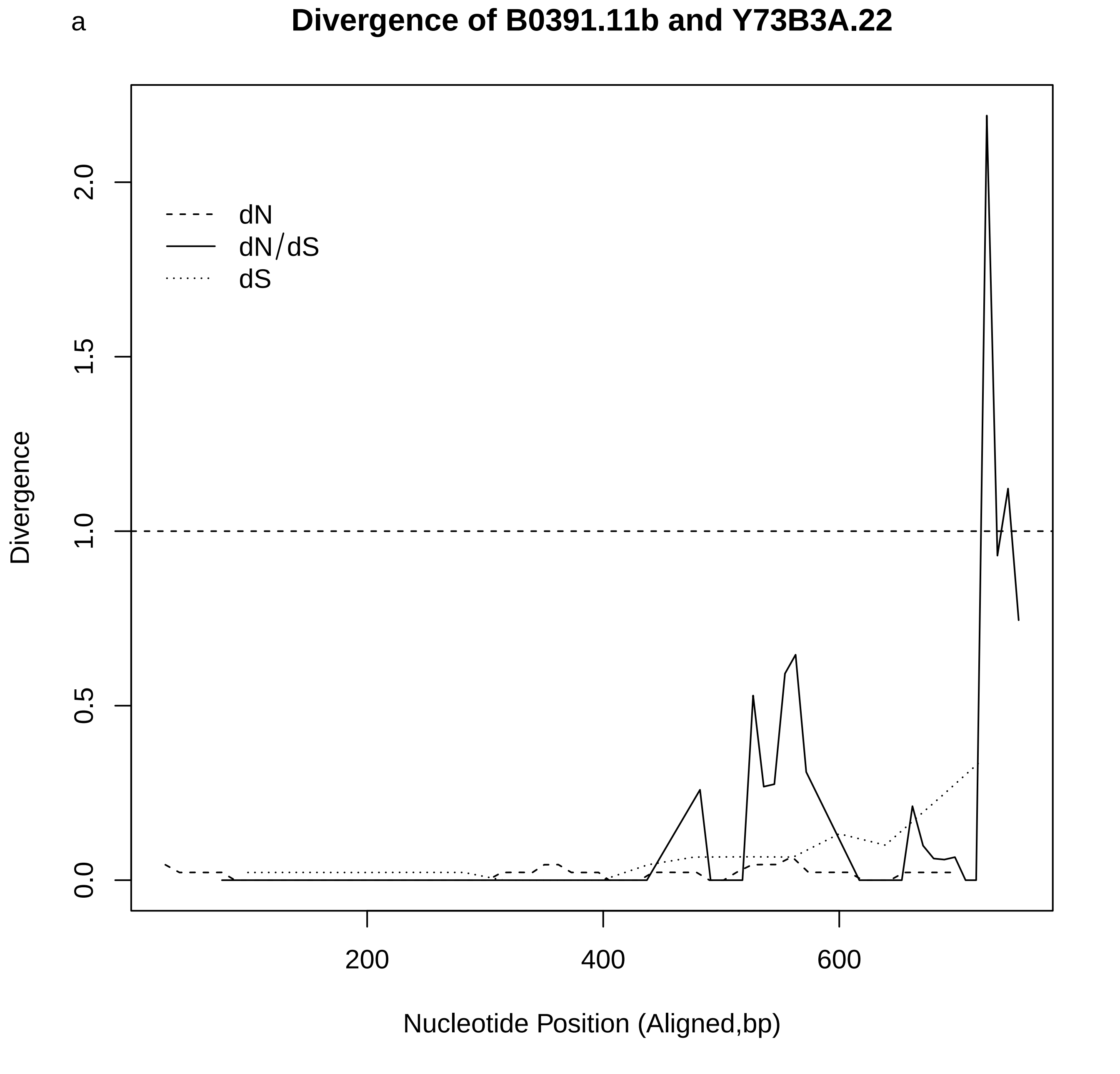
a
Divergence of B0391.11b and Y73B3A.22
dN
dN/dS
dS
Divergence
0.0
0.5
1.0
1.5
2.0
200
400
600
Nucleotide Position (Aligned,bp)

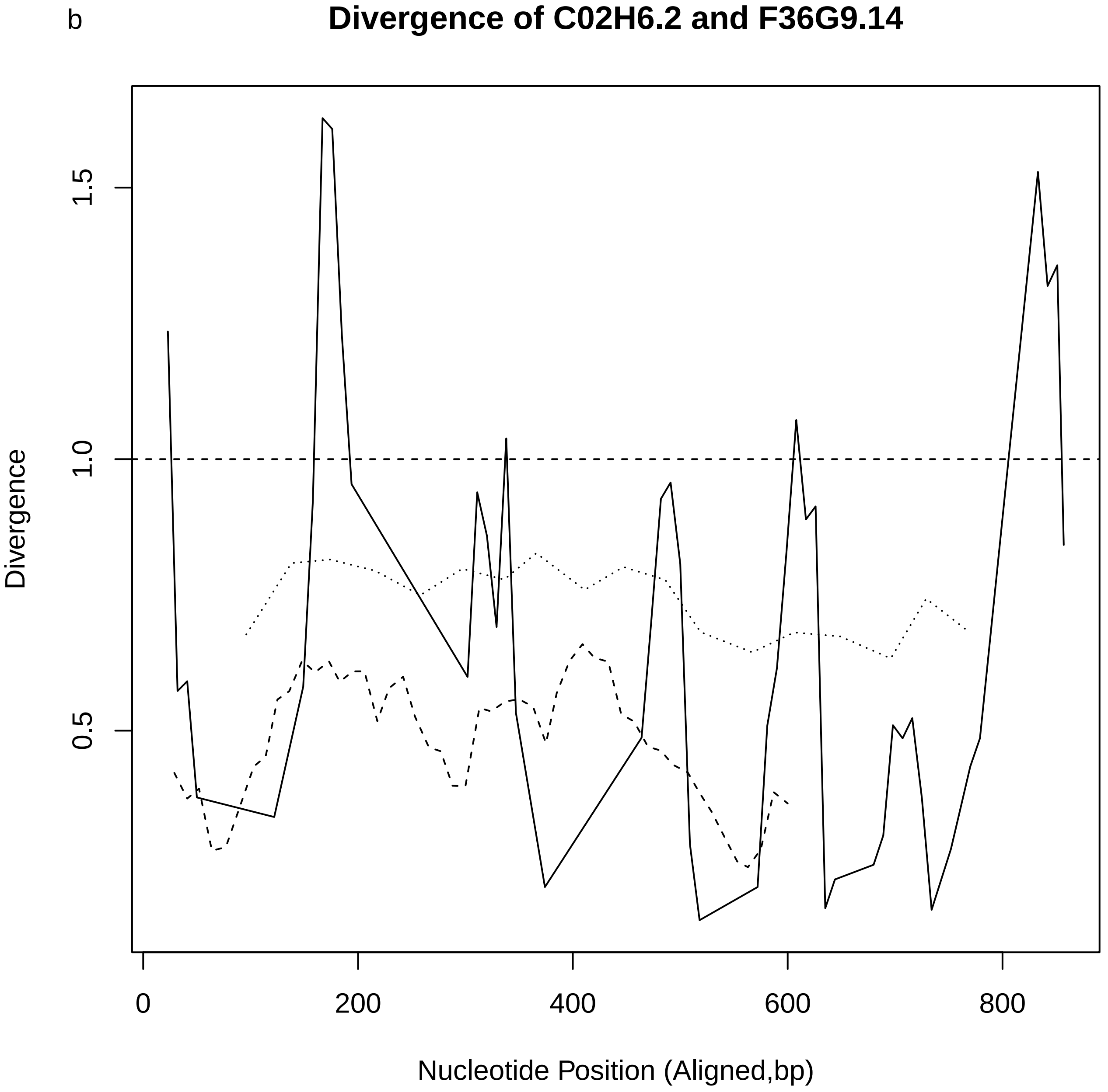
b
Divergence of C02H6.2 and F36G9.14
Divergence
1.5
1.0
0.5
0
200
400
600
800
Nucleotide Position (Aligned,bp)

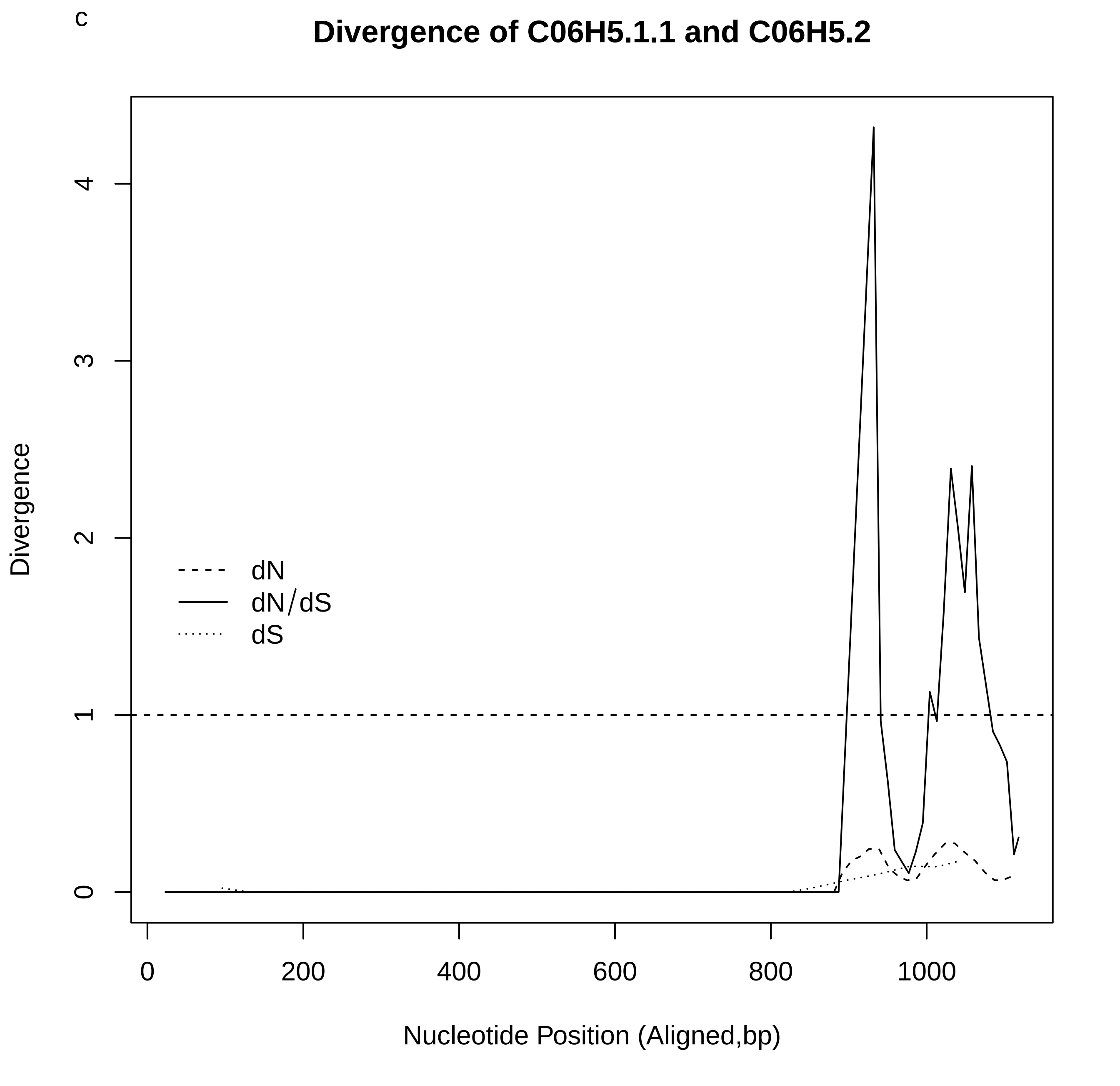
c
Divergence of C06H5.1.1 and C06H5.2
Divergence
Nucleotide Position (Aligned,bp)
dN
dN/dS
dS
0
1
2
3
4
0
200
400
600
800
1000

d

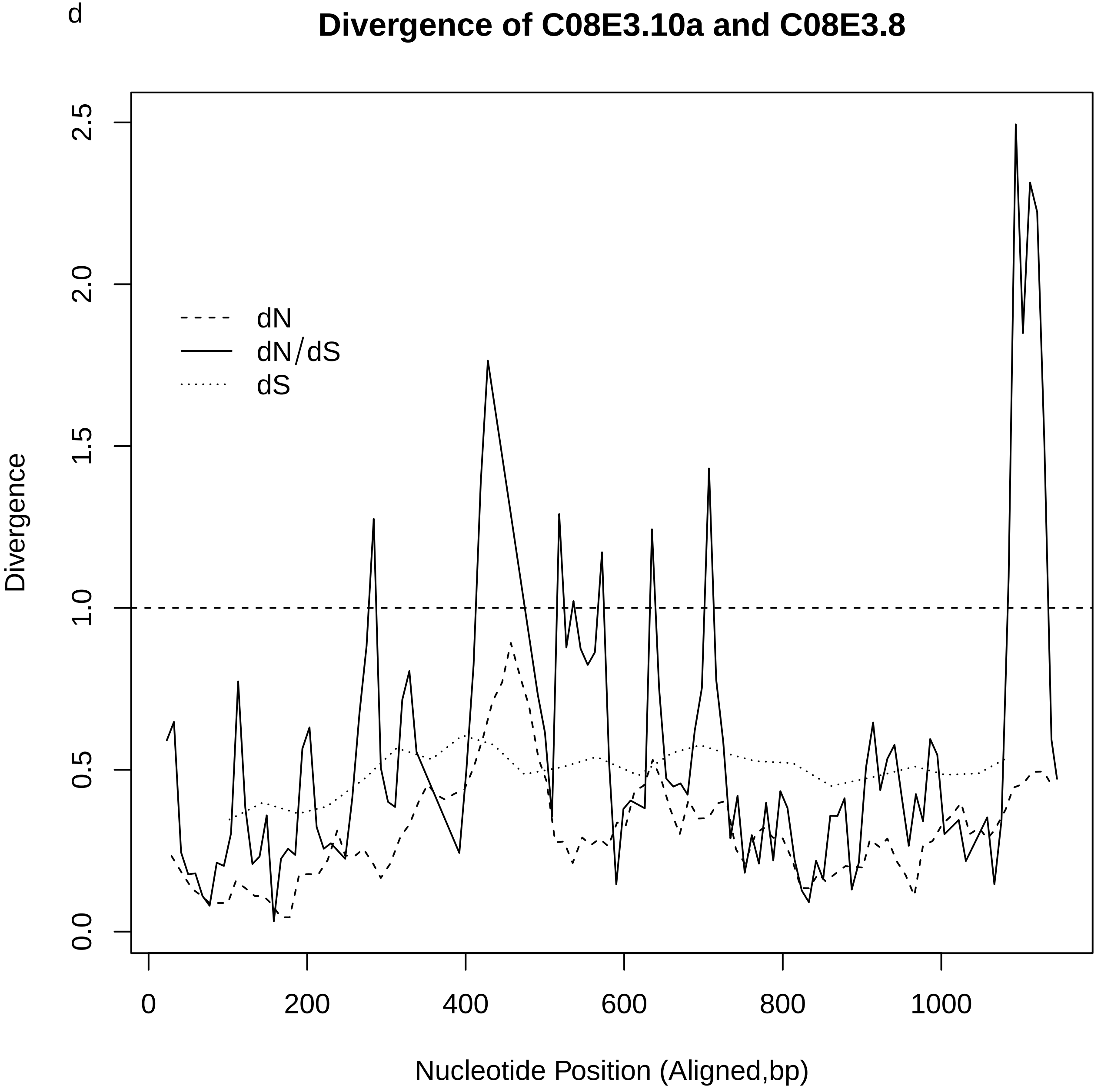
Divergence of C08E3.10a and C08E3.8
dN
dN/dS
dS
Divergence
0.0
0.5
1.0
1.5
2.0
2.5
0
200
400
600
800
1000
Nucleotide Position (Aligned,bp)

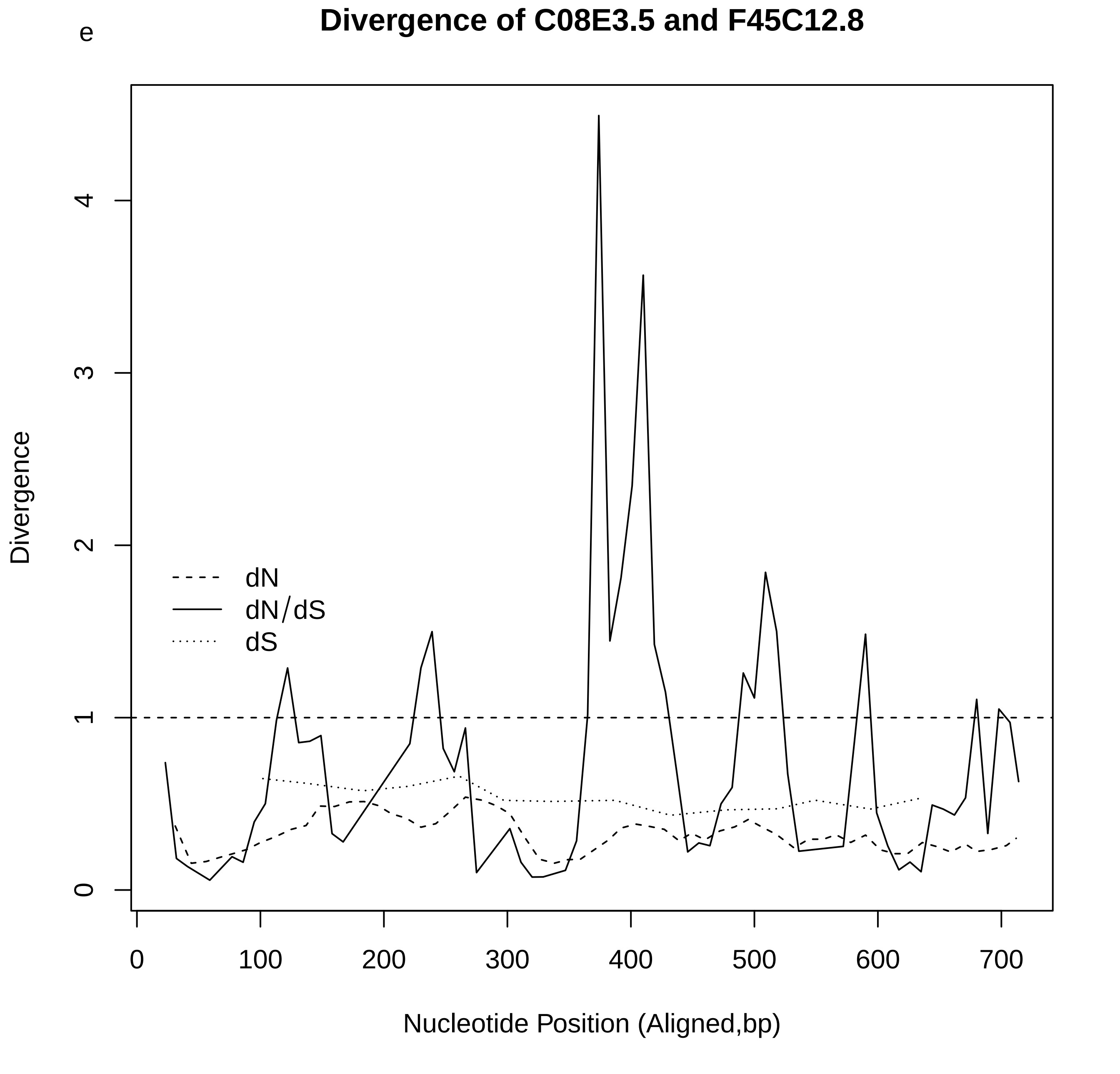

e
Divergence of C08E3.5 and F45C12.8
Divergence
0
1
2
3
4
dN
dN/dS
dS
0
100
200
300
400
500
600
700
Nucleotide Position (Aligned,bp)

f

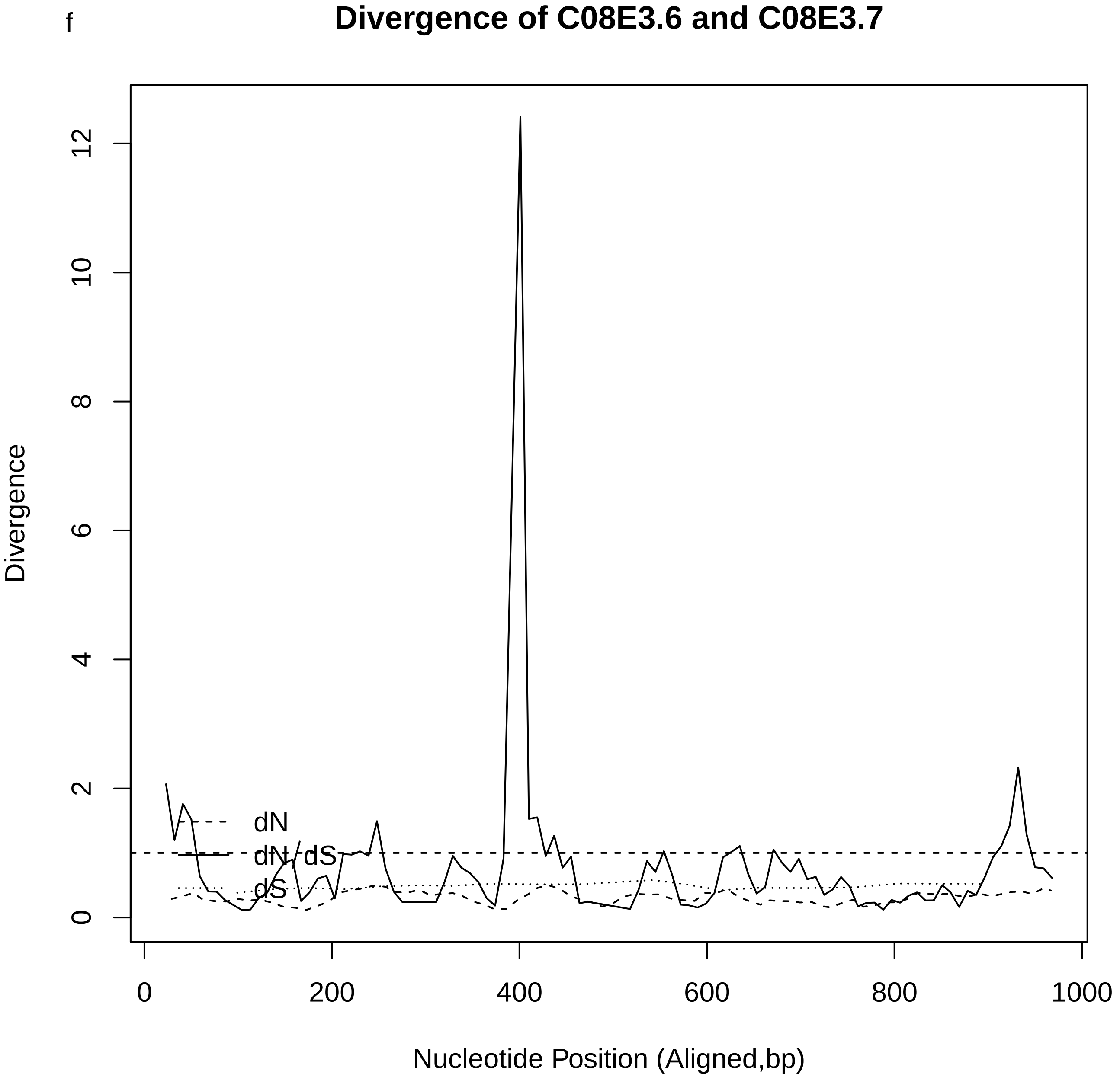

Divergence of C08E3.6 and C08E3.7
Divergence
0
2
4
6
8
10
12
dN
dN/dS
dS
0
200
400
600
800
1000
Nucleotide Position (Aligned,bp)

g

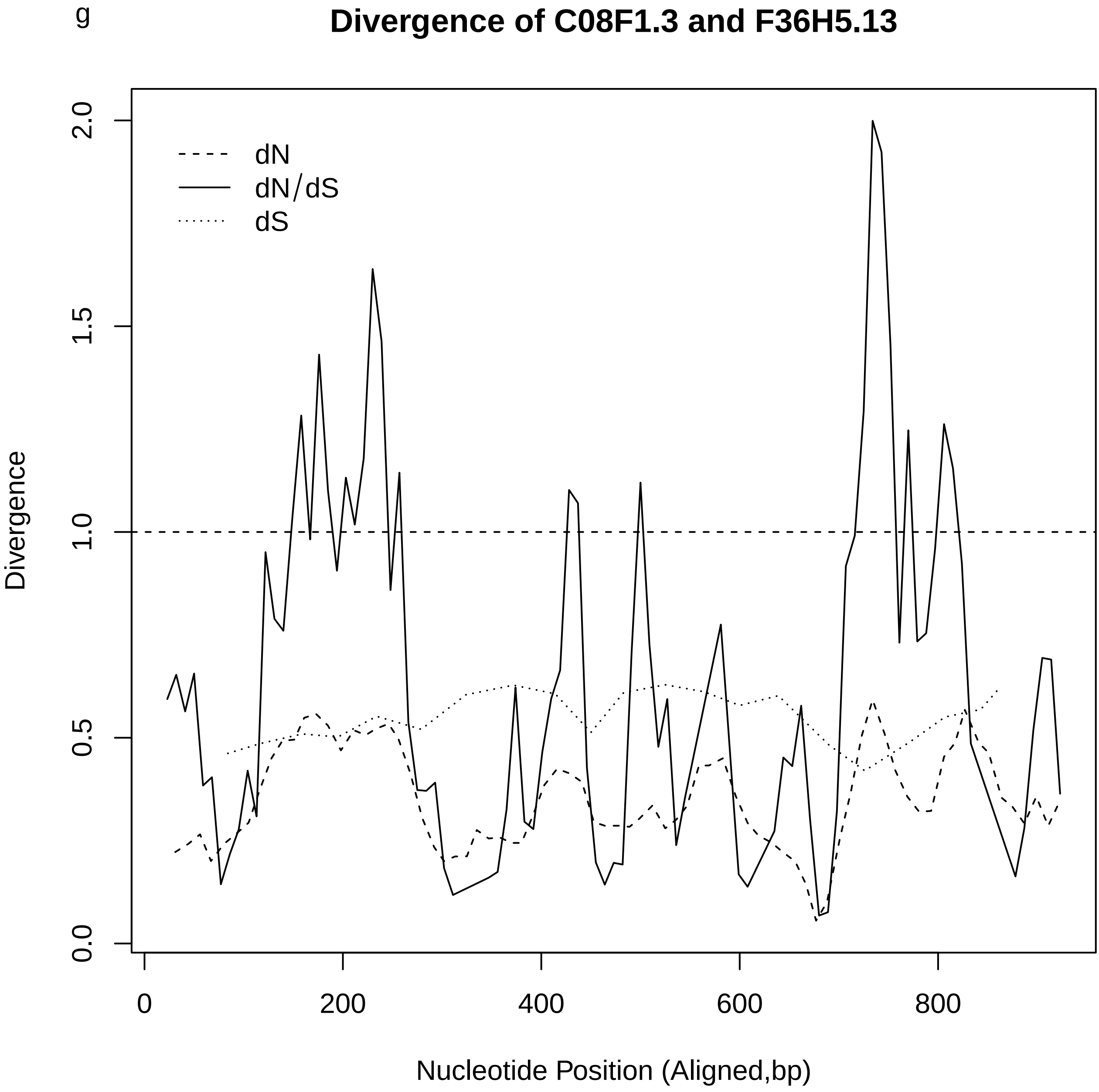
Divergence of C08F1.3 and F36H5.13
dN
dN/dS
dS
Divergence
0.0
0.5
1.0
1.5
2.0
0
200
400
600
800
Nucleotide Position (Aligned,bp)

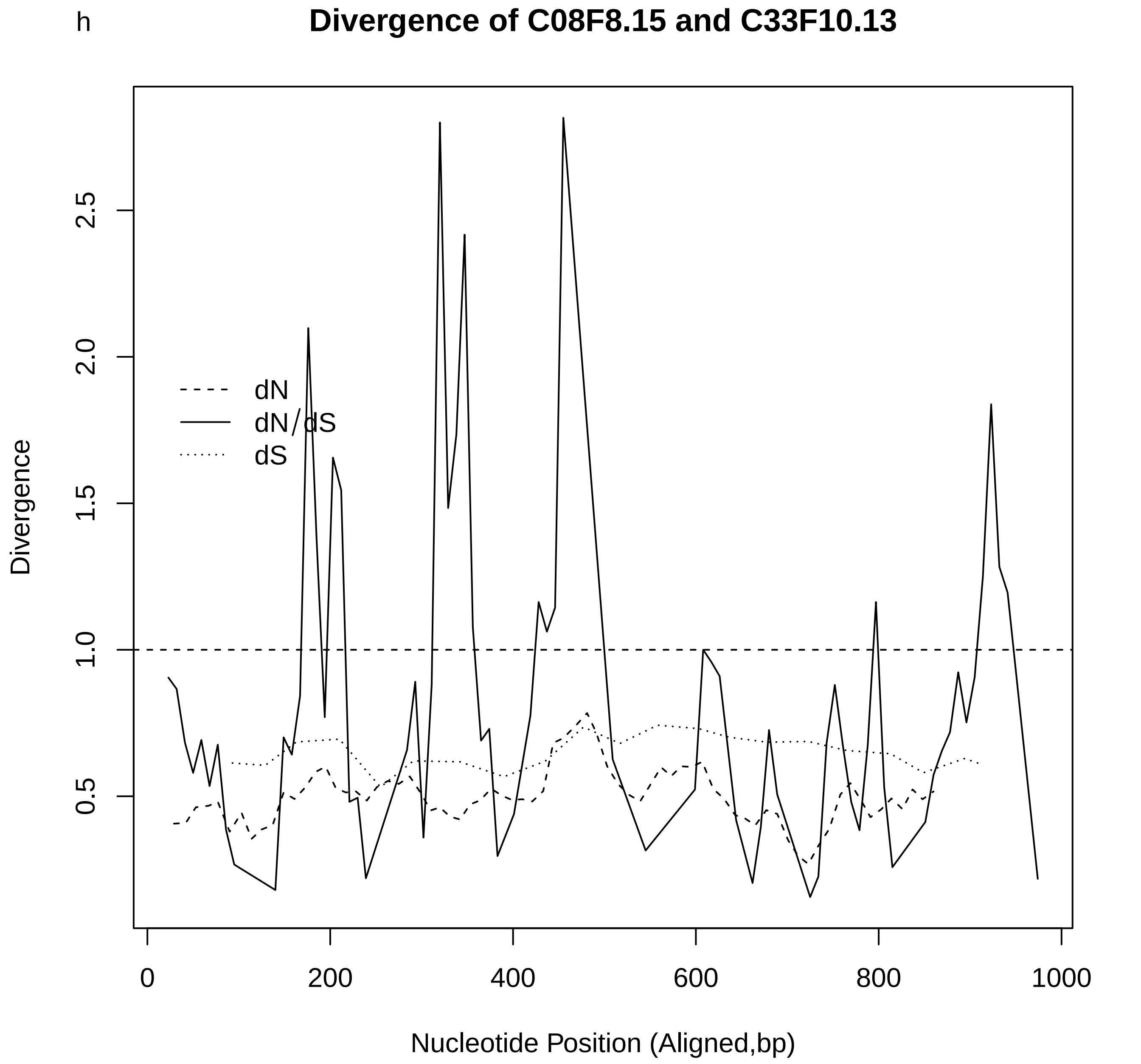
h
Divergence of C08F8.15 and C33F10.13
Divergence
Nucleotide Position (Aligned,bp)
dN
dN/dS
dS
0.5
1.0
1.5
2.0
2.5
0
200
400
600
800
1000

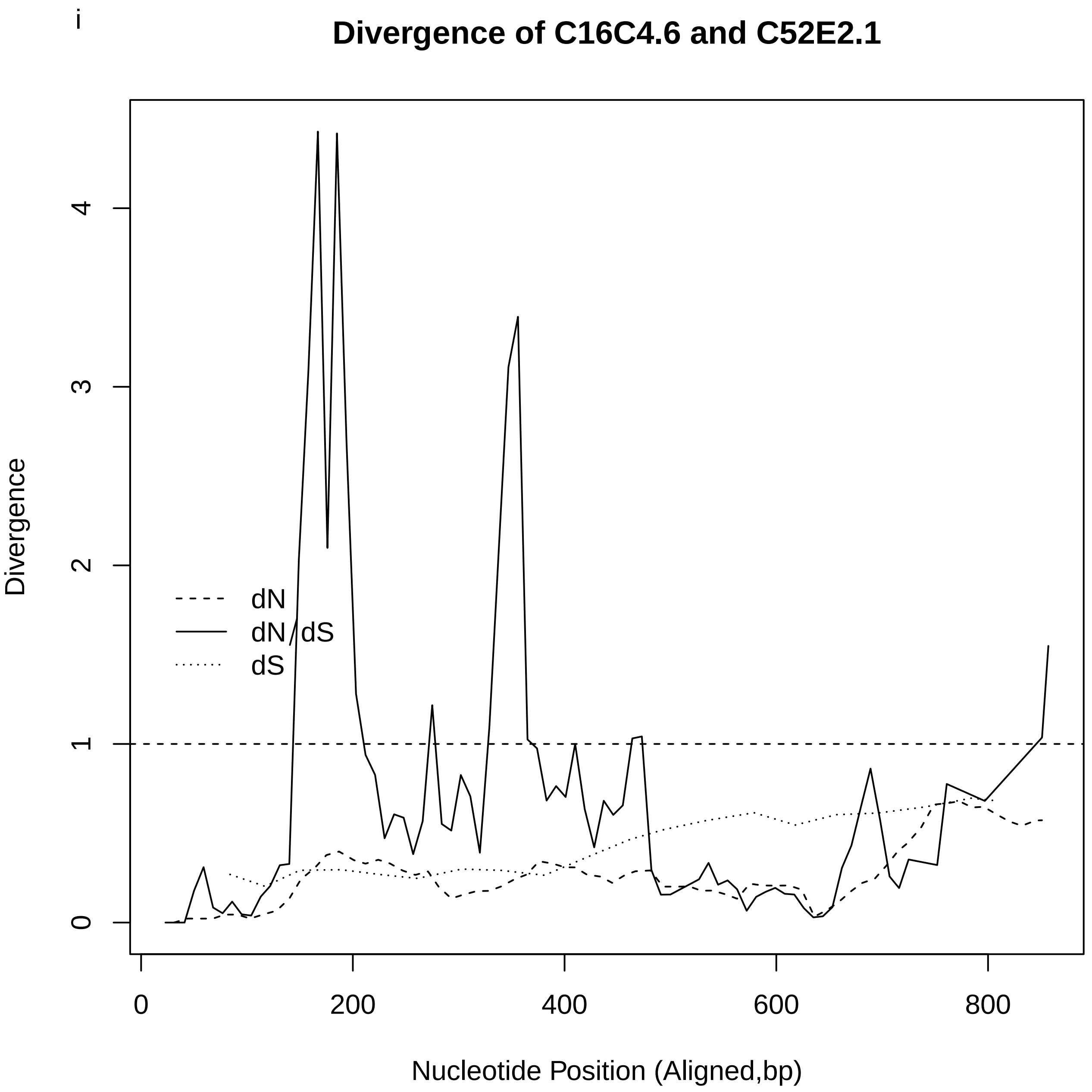
i
Divergence of C16C4.6 and C52E2.1
Divergence
Nucleotide Position (Aligned,bp)
dN
dN/dS
dS
0
1
2
3
4
0
200
400
600
800

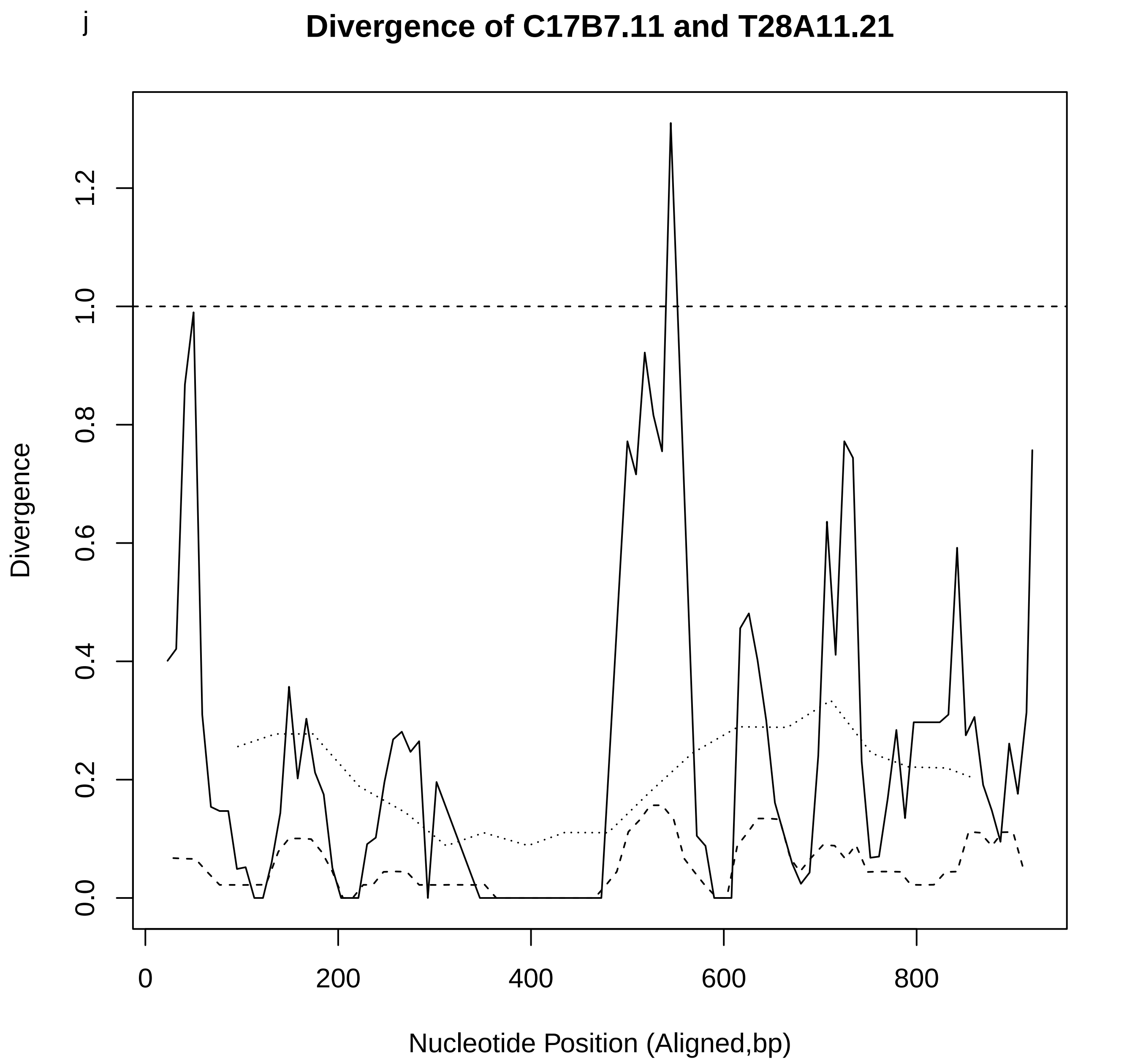
j
Divergence of C17B7.11 and T28A11.21
Divergence
0.0
0.2
0.4
0.6
0.8
1.0
1.2
0
200
400
600
800
Nucleotide Position (Aligned,bp)

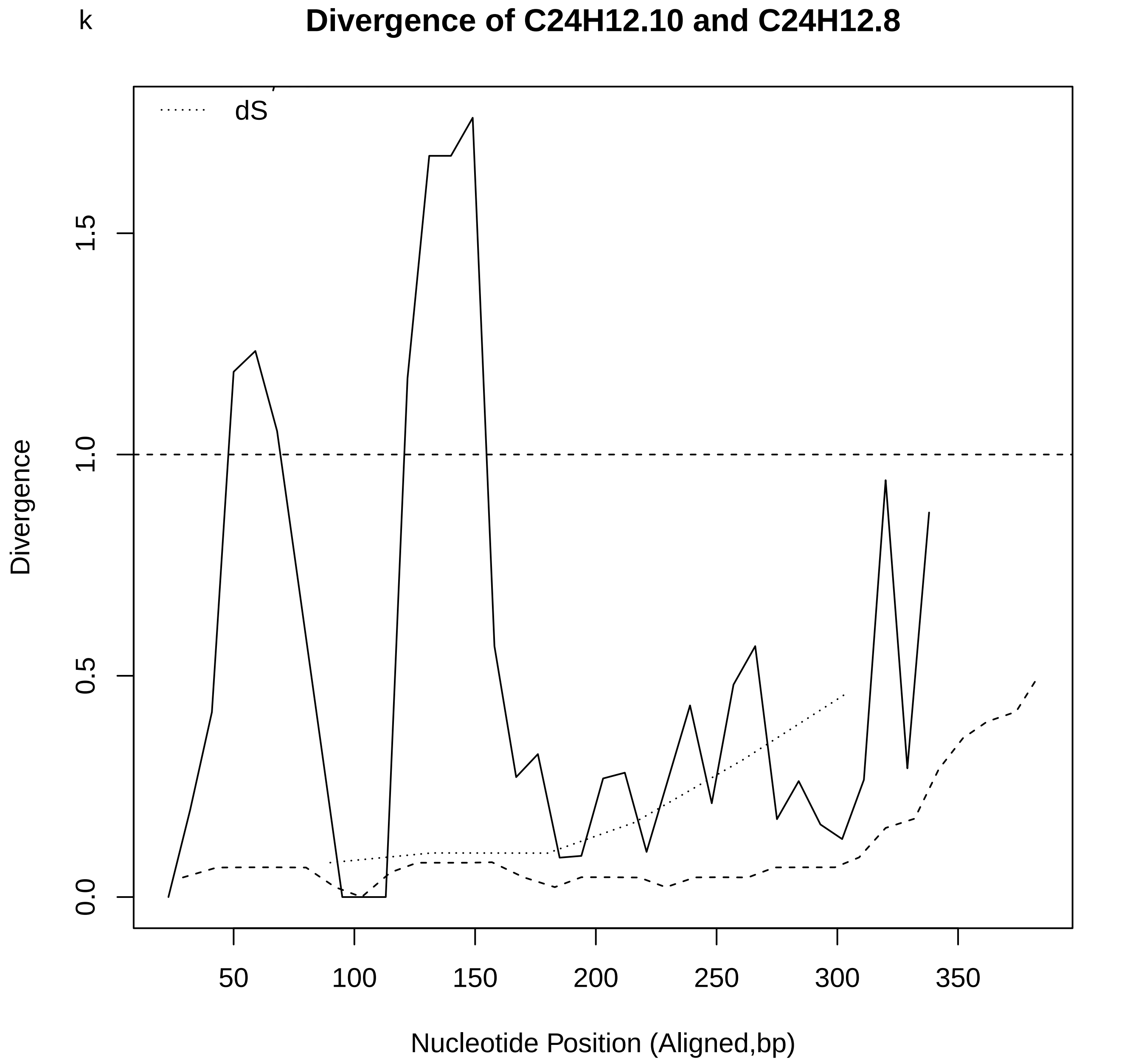

k
Divergence of C24H12.10 and C24H12.8
dS
Divergence
0.0
0.5
1.0
1.5
50
100
150
200
250
300
350
Nucleotide Position (Aligned,bp)

I

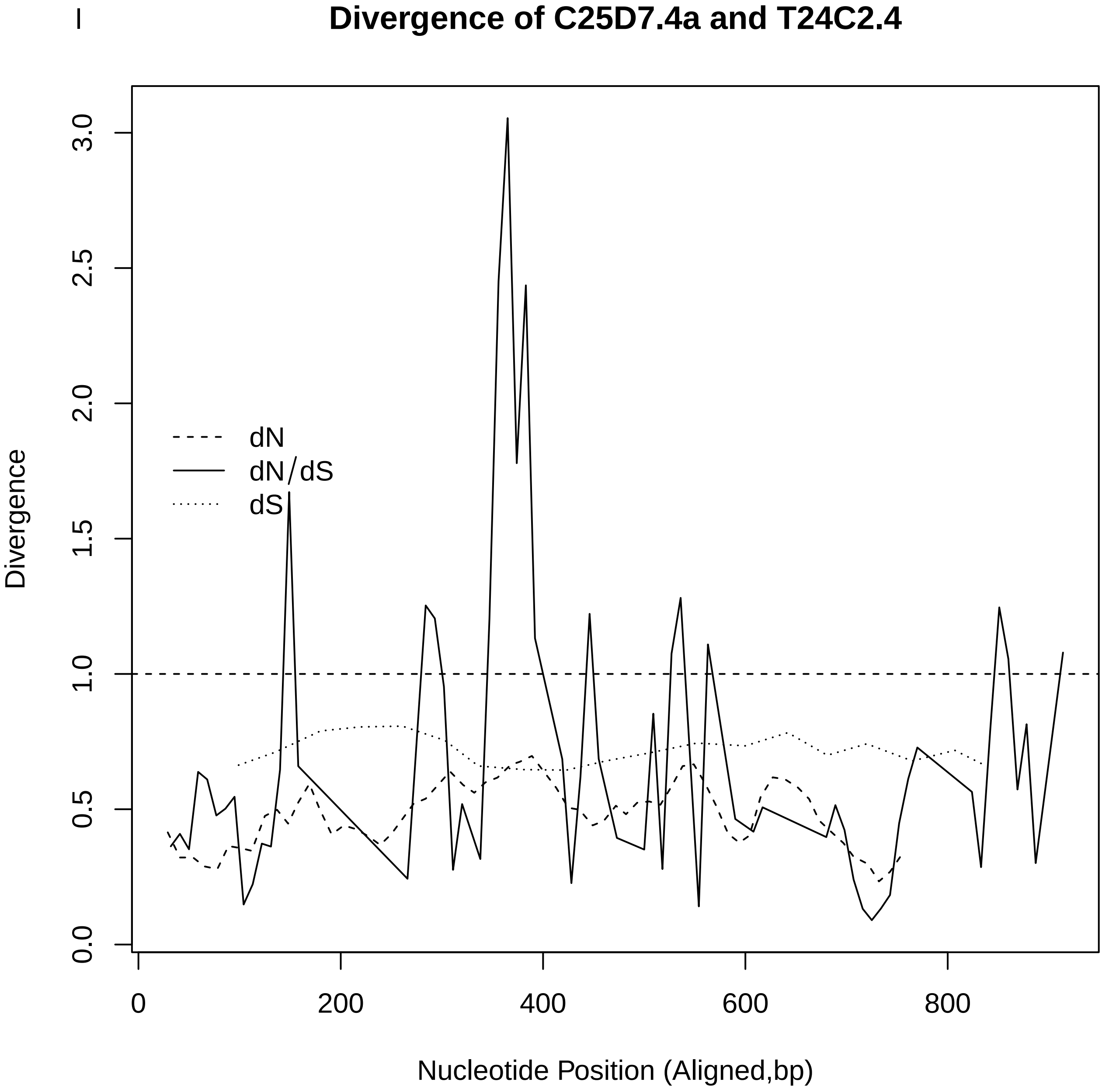

Divergence of C25D7.4a and T24C2.4
Divergence
3.0
2.5
2.0
1.5
1.0
0.5
0.0
dN
dN/dS
dS
0
200
400
600
800
Nucleotide Position (Aligned,bp)

m

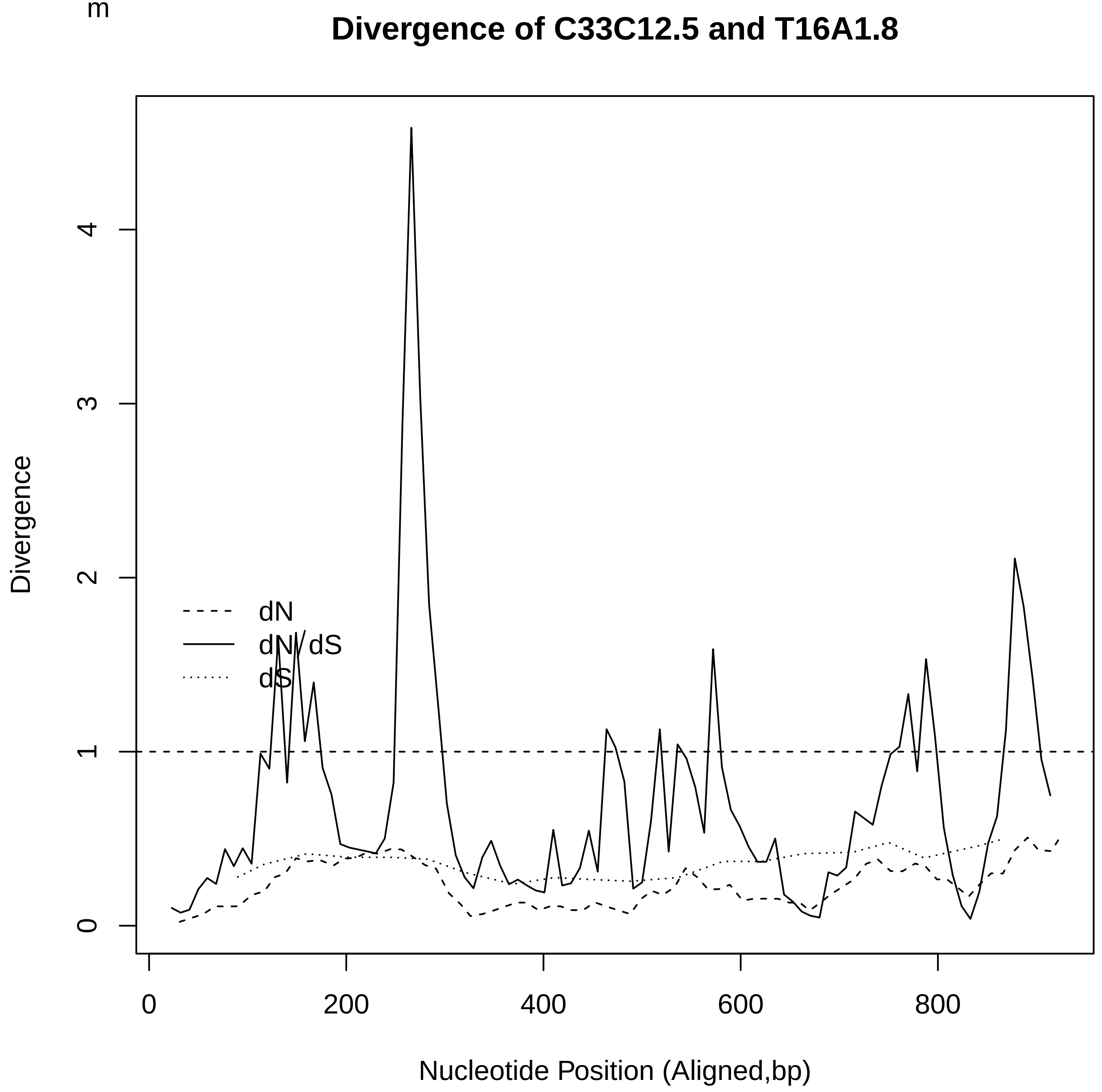
Divergence of C33C12.5 and T16A1.8
Divergence
Nucleotide Position (Aligned,bp)
dN
dN/dS
dS
0
1
2
3
4
0
200
400
600
800

n

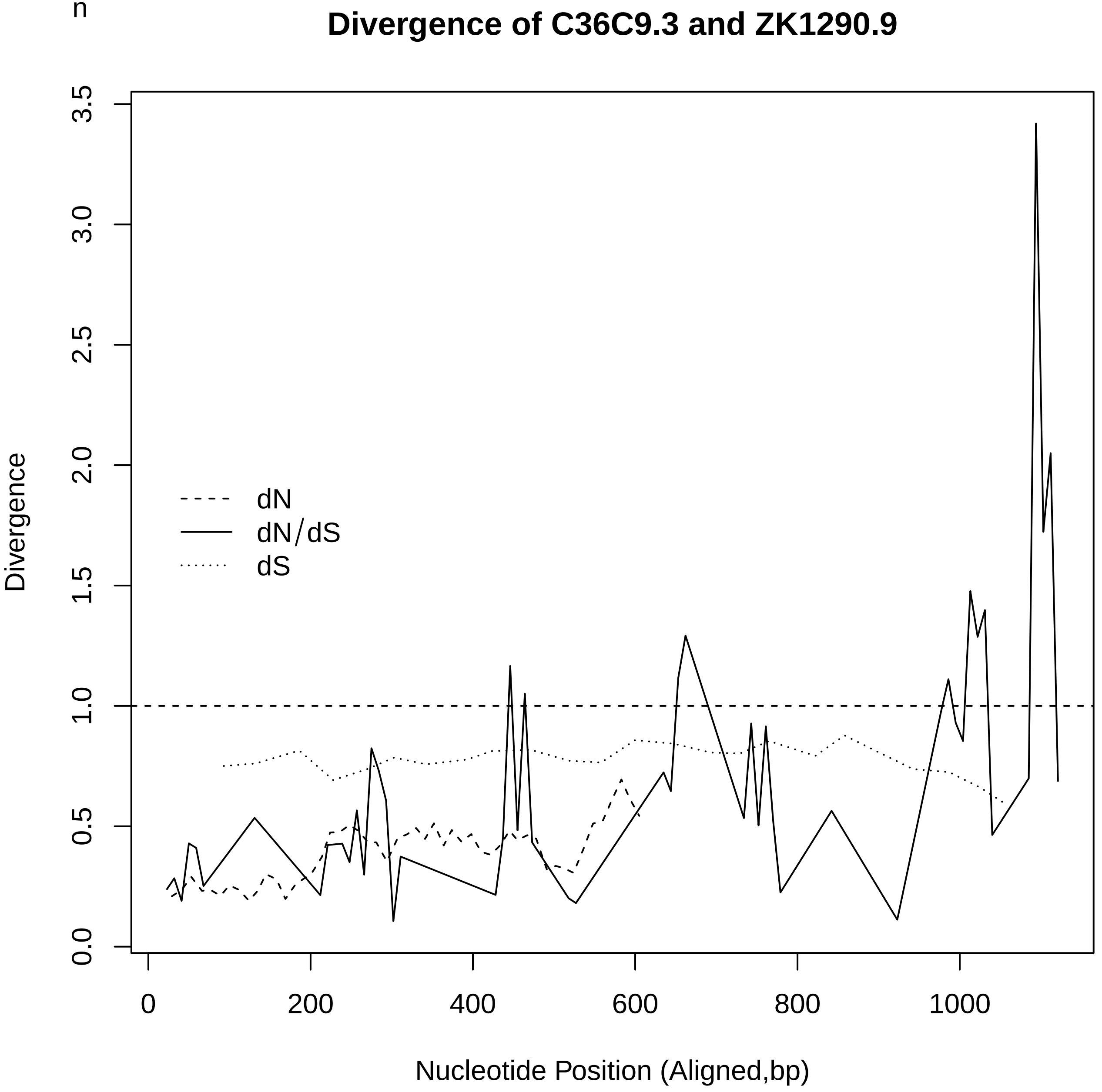
Divergence of C36C9.3 and ZK1290.9
Divergence
Nucleotide Position (Aligned,bp)
dN
dN/dS
dS
0.0
0.5
1.0
1.5
2.0
2.5
3.0
3.5
0
200
400
600
800
1000

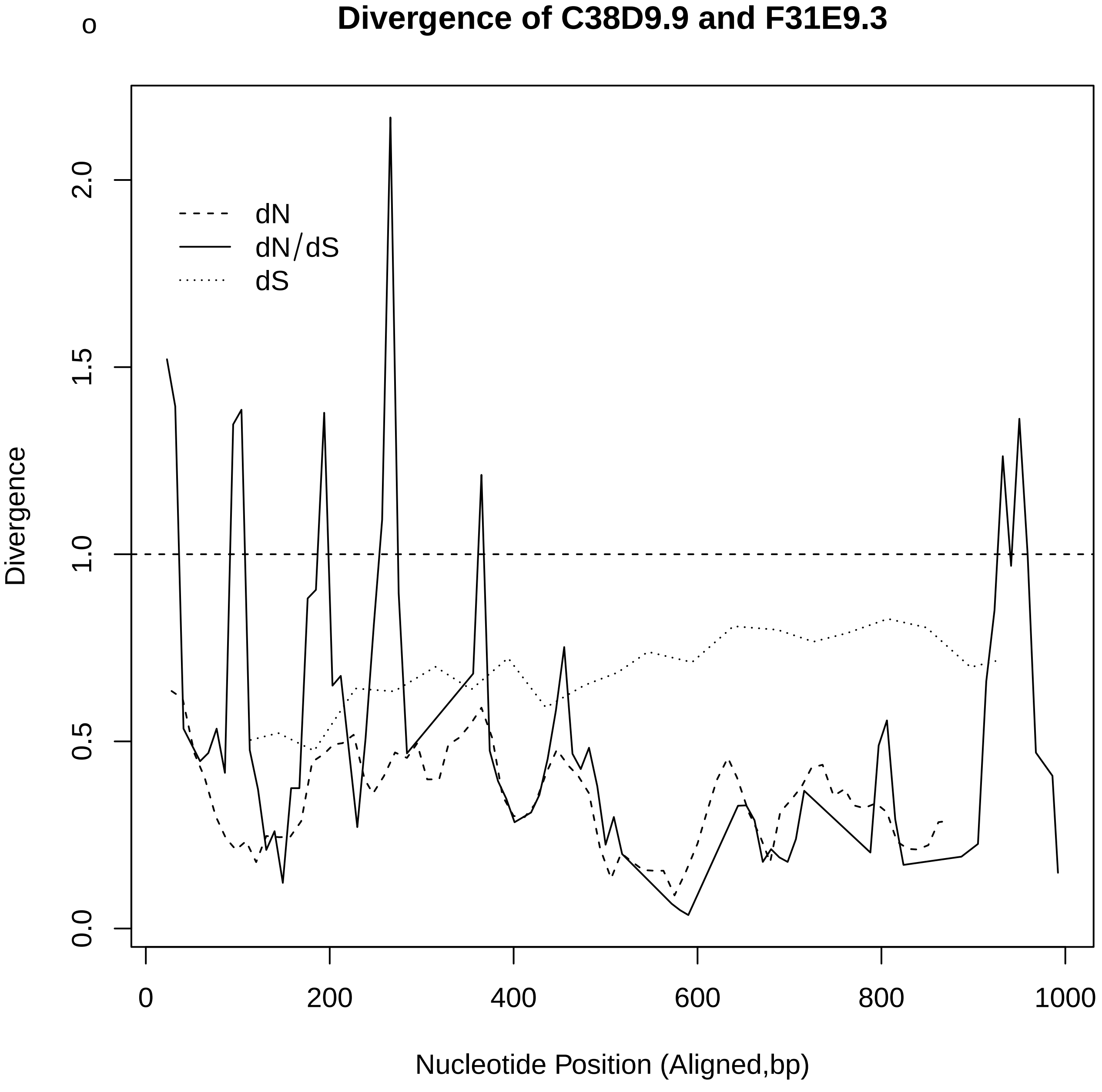
Divergence of C38D9.9 and F31E9.3
dN
dN/dS
dS
Divergence
0.0
0.5
1.0
1.5
2.0
0
200
400
600
800
1000
Nucleotide Position (Aligned,bp)

p

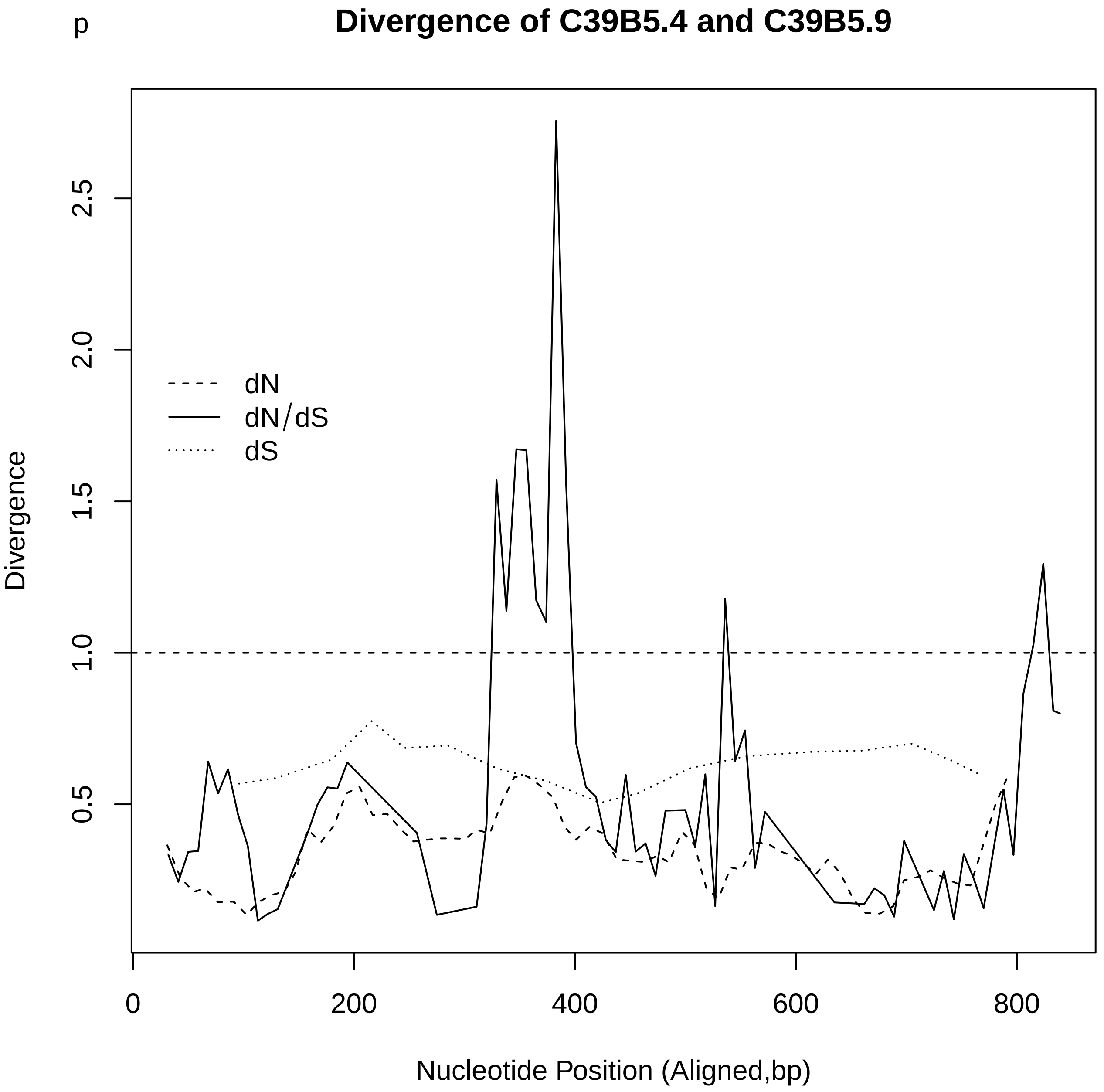
Divergence of C39B5.4 and C39B5.9
dN
dN/dS
dS
Divergence
2.5
2.0
1.5
1.0
0.5
0
200
400
600
800
Nucleotide Position (Aligned,bp)

q

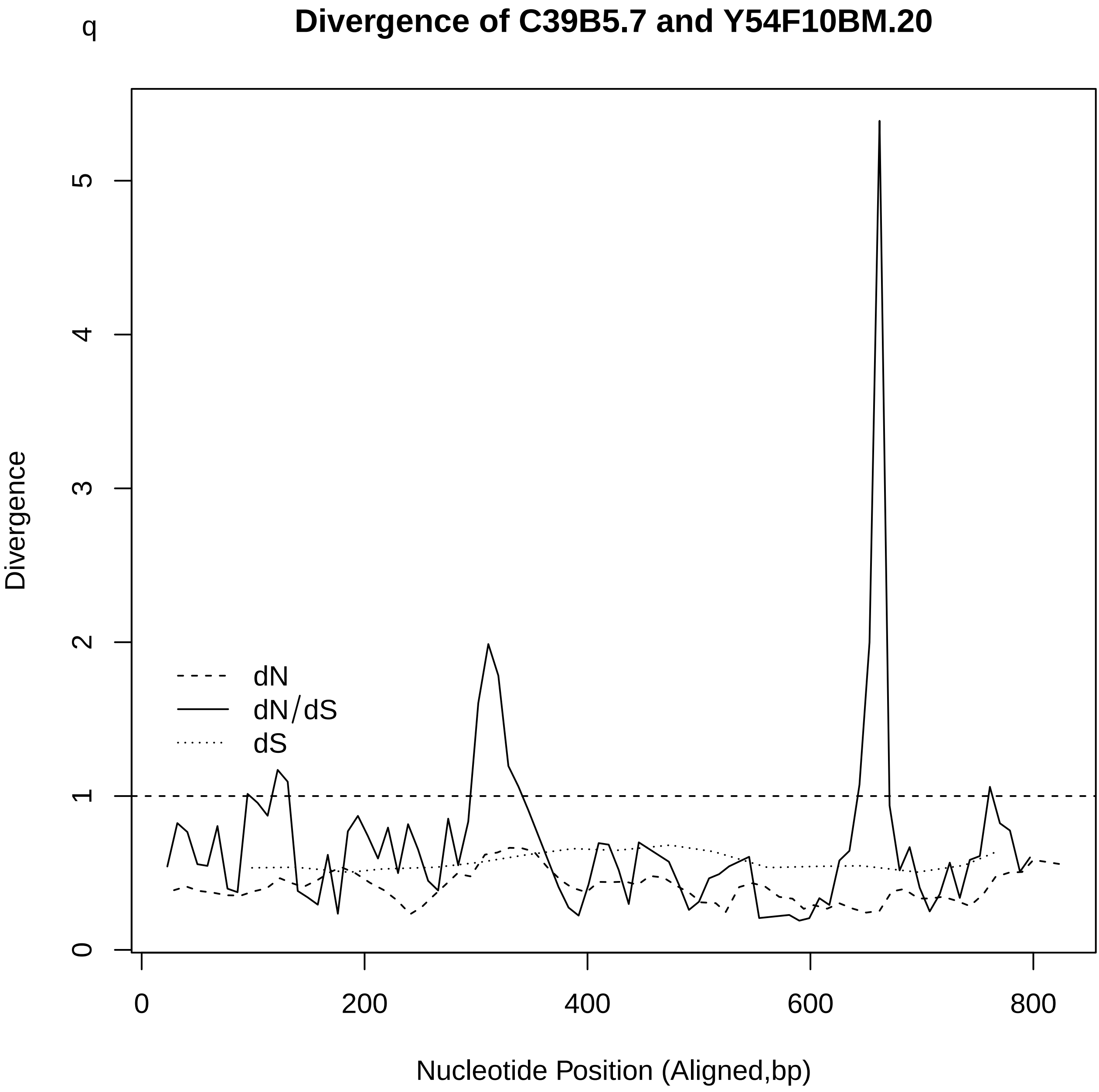

Divergence of C39B5.7 and Y54F10BM.20
Divergence
Nucleotide Position (Aligned,bp)
dN
dN/dS
dS
0
1
2
3
4
5
0
200
400
600
800

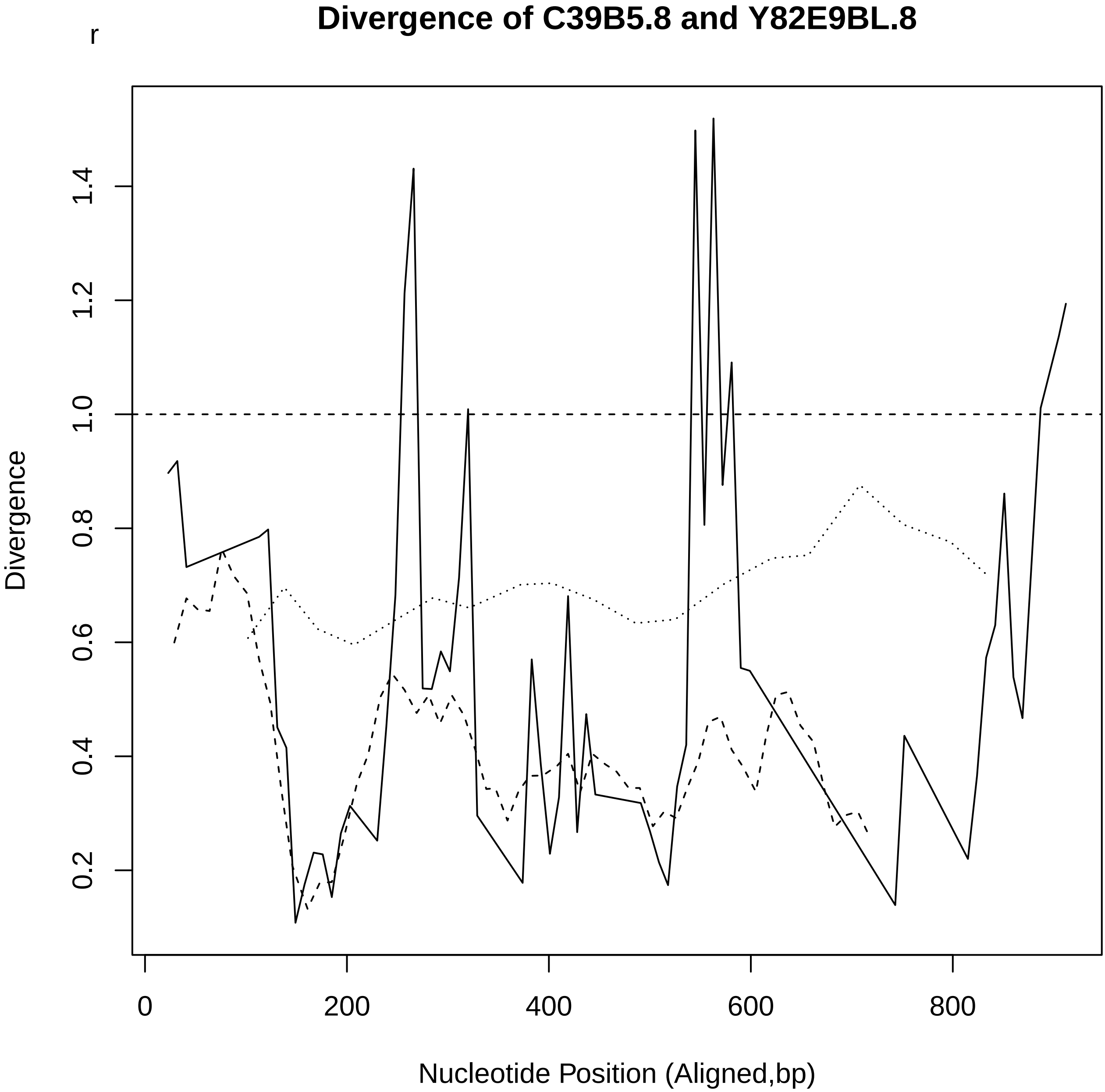
Divergence of C39B5.8 and Y82E9BL.8
r
Divergence
0.2
0.4
0.6
0.8
1.0
1.2
1.4
0
200
400
600
800
Nucleotide Position (Aligned,bp)

s

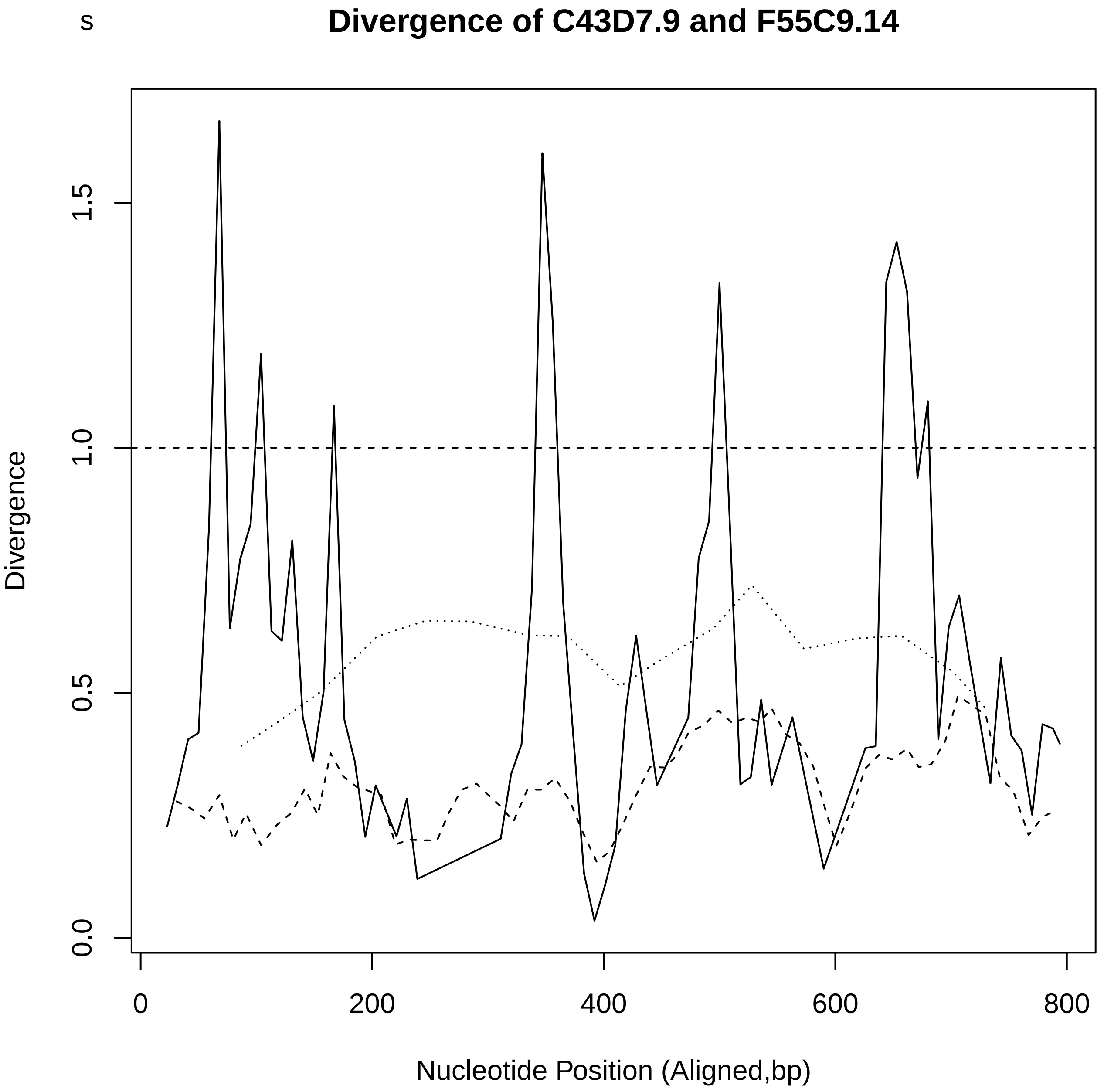

Divergence of C43D7.9 and F55C9.14
Divergence
0.0
0.5
1.0
1.5
0
200
400
600
800
Nucleotide Position (Aligned,bp)

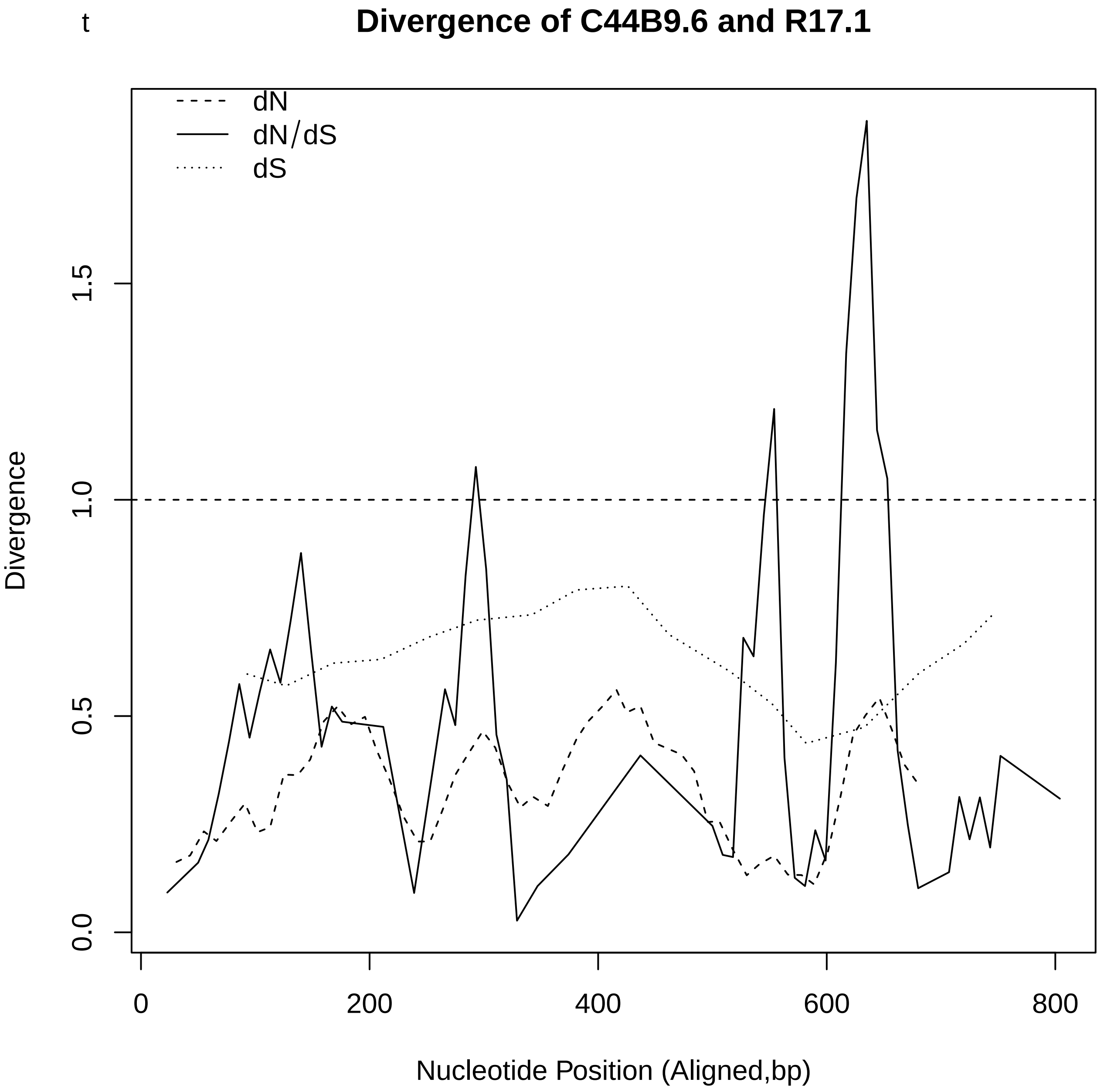
t
Divergence of C44B9.6 and R17.1
dN
dN/dS
dS
Divergence
0.0
0.5
1.0
1.5
0
200
400
600
800
Nucleotide Position (Aligned,bp)

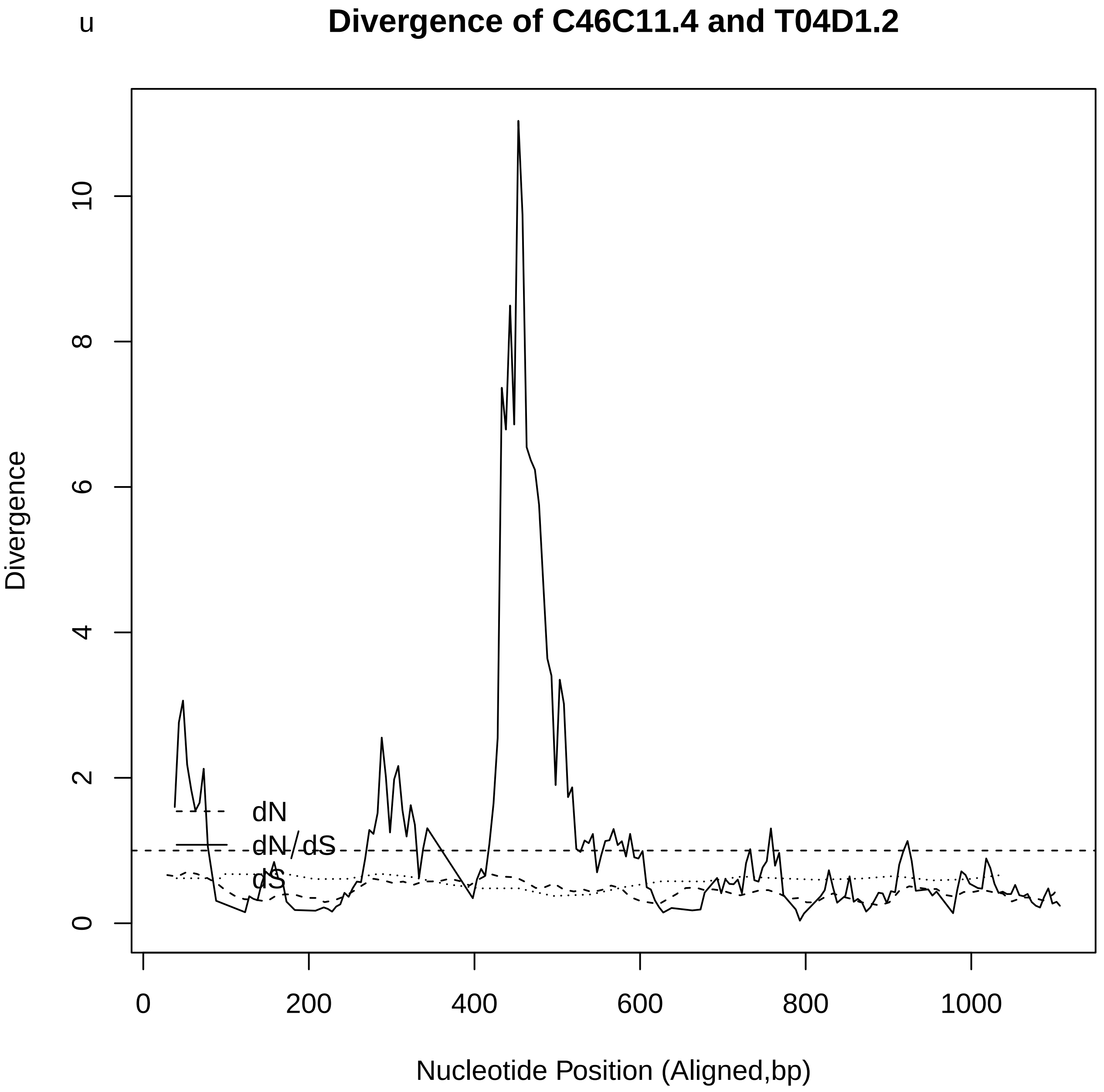
u
Divergence of C46C11.4 and T04D1.2
Divergence
0
2
4
6
8
10
0
200
400
600
800
1000
Nucleotide Position (Aligned,bp)
dN
dN/dS
dS

v

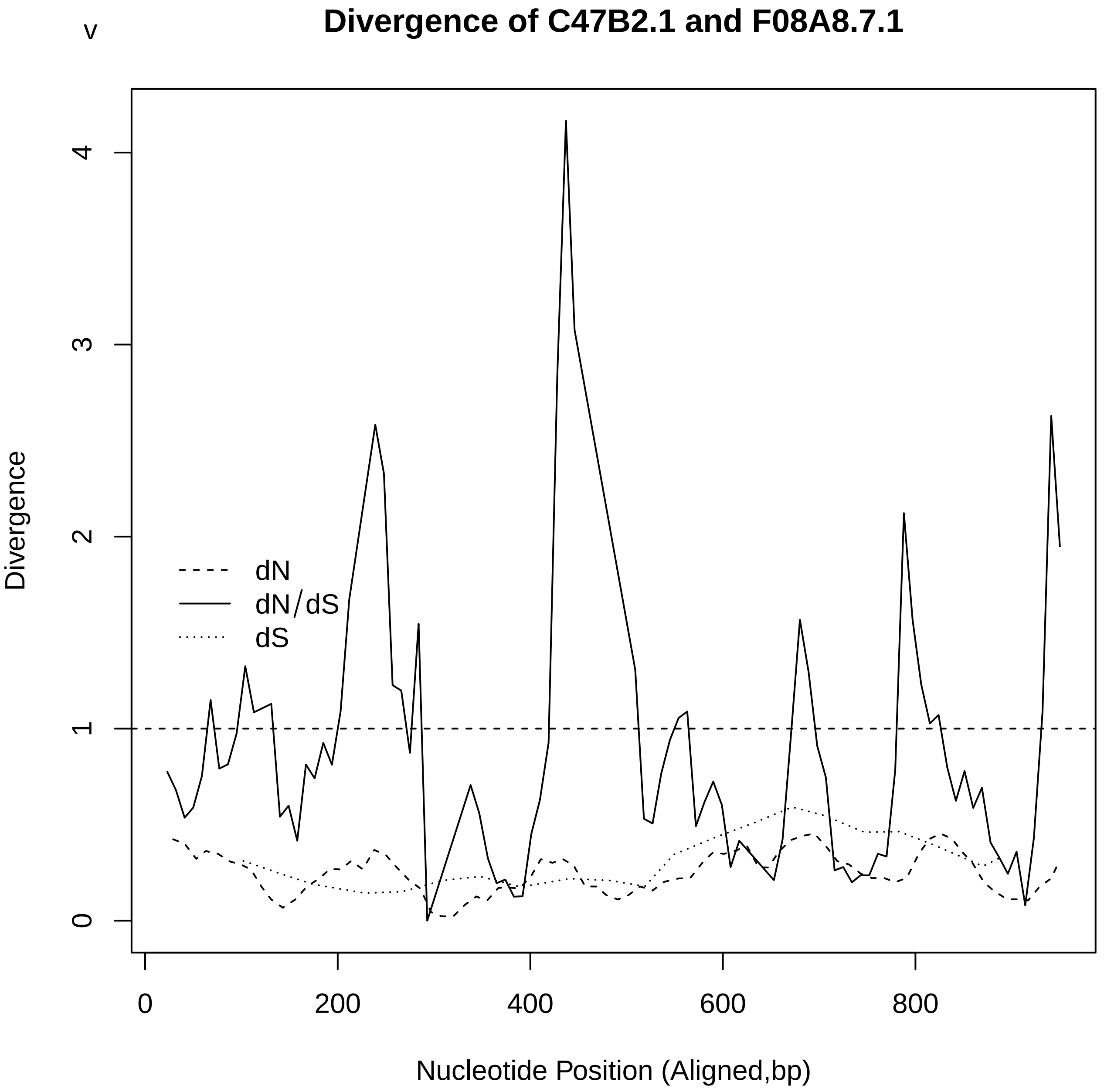

Divergence of C47B2.1 and F08A8.7.1
Divergence
Nucleotide Position (Aligned,bp)
0
1
2
3
4
0
200
400
600
800
dN
dN/dS
dS

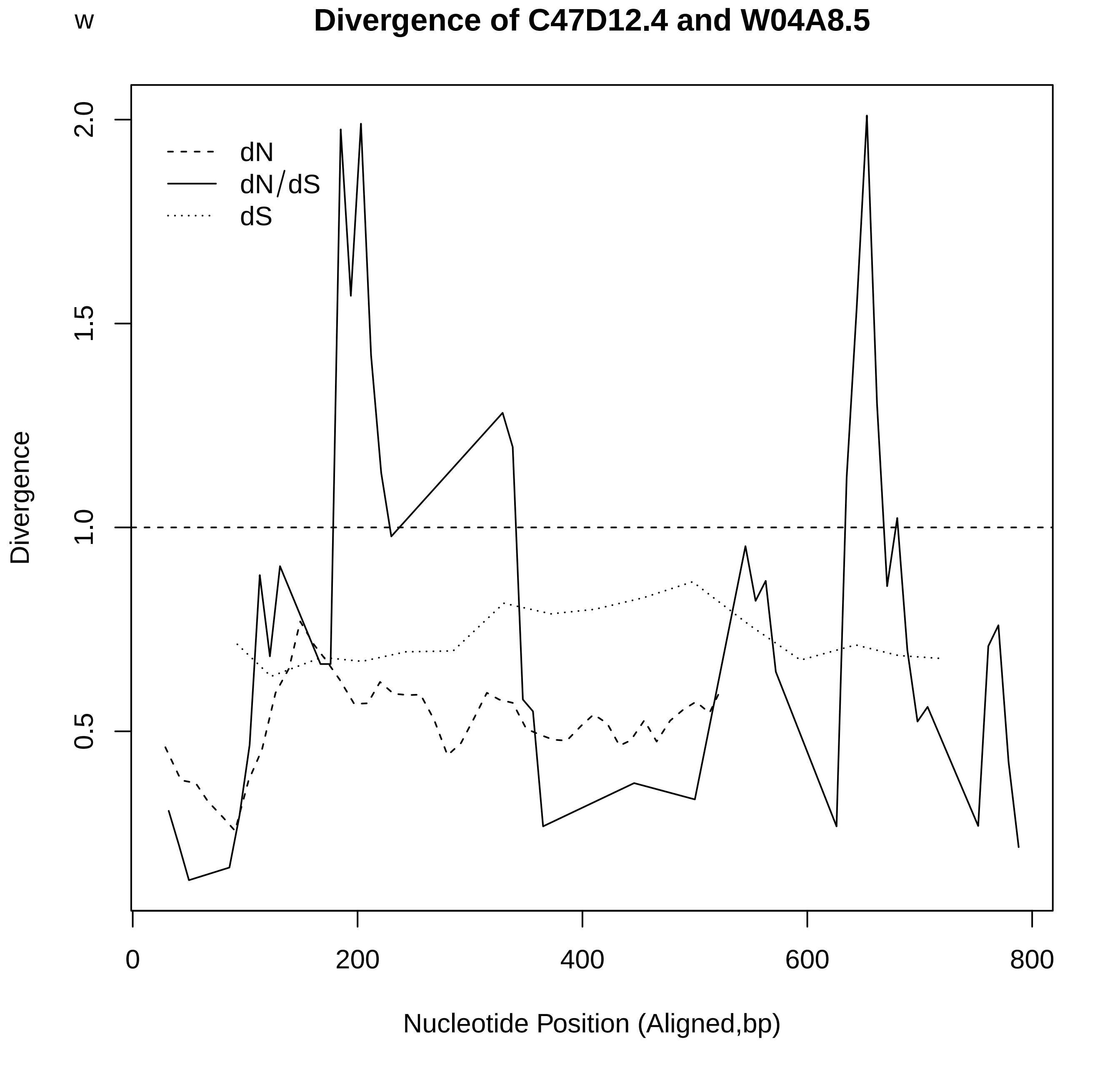

w
Divergence of C47D12.4 and W04A8.5
dN
dN/dS
dS
Divergence
2.0
1.5
1.0
0.5
0
200
400
600
800
Nucleotide Position (Aligned,bp)

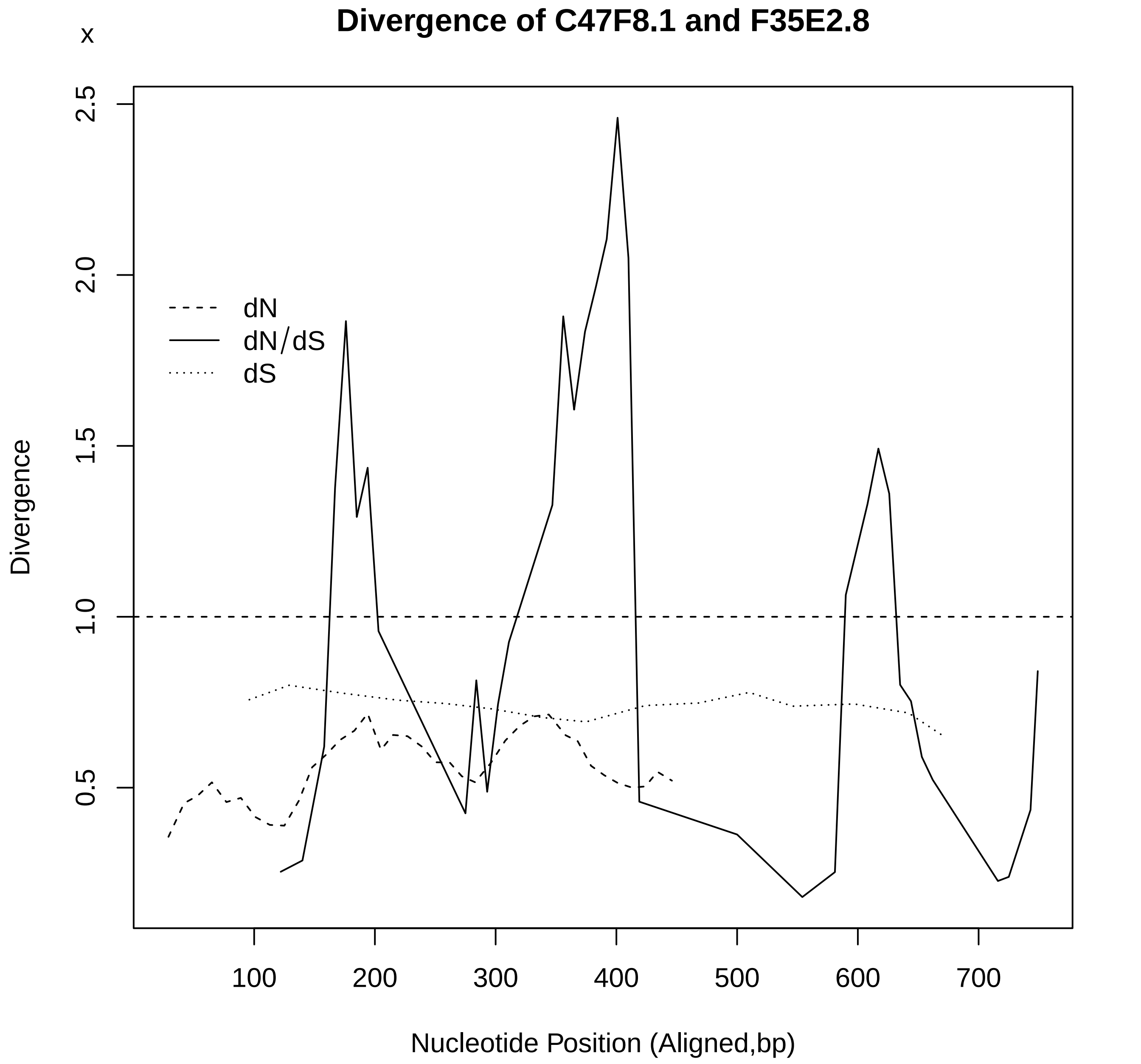
x
Divergence of C47F8.1 and F35E2.8
dN
dN/dS
dS
Divergence
0.5
1.0
1.5
2.0
2.5
100
200
300
400
500
600
700
Nucleotide Position (Aligned,bp)

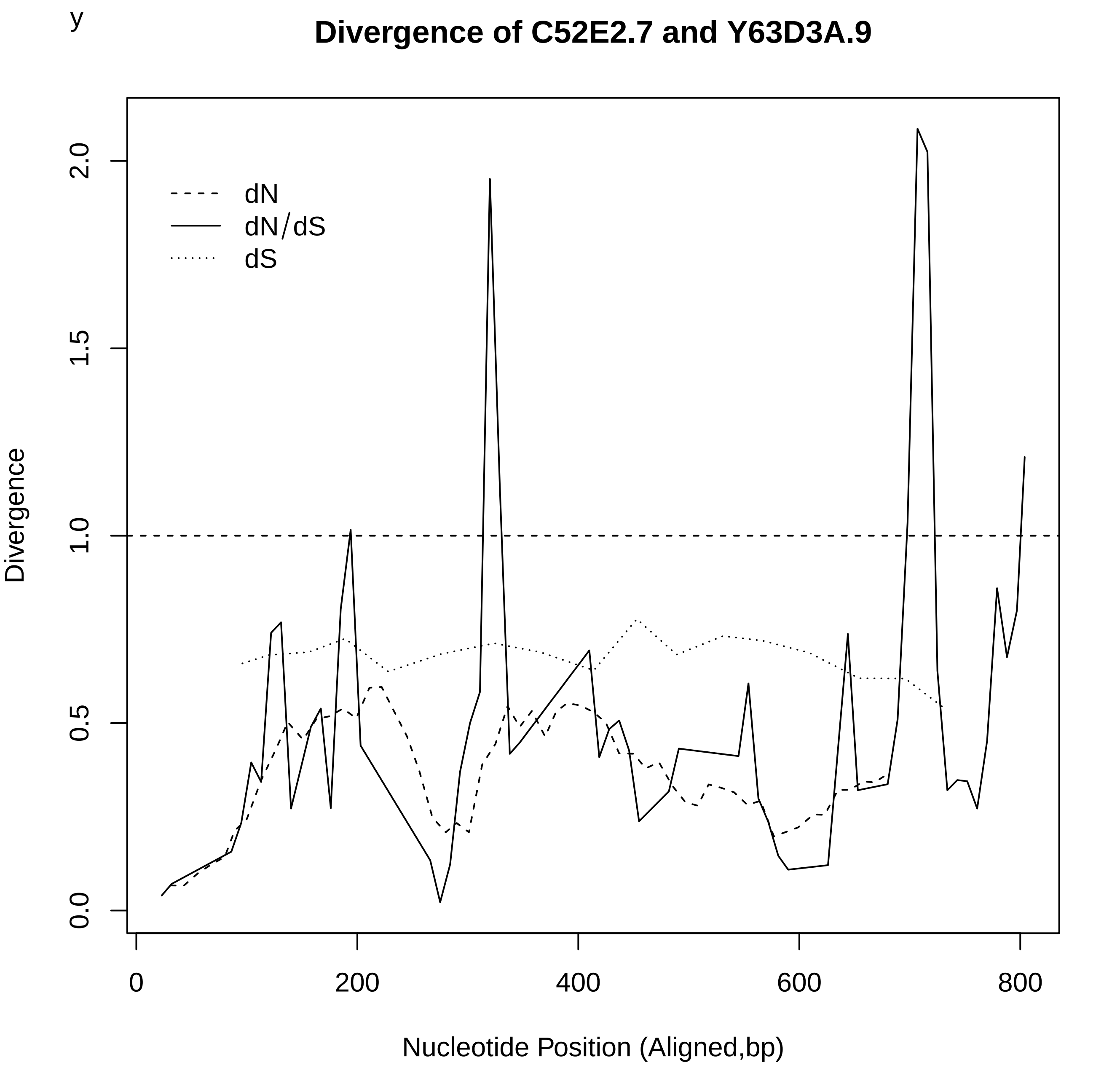

y
Divergence of C52E2.7 and Y63D3A.9
dN
dN/dS
dS
Divergence
0.0
0.5
1.0
1.5
2.0
0
200
400
600
800
Nucleotide Position (Aligned,bp)

z

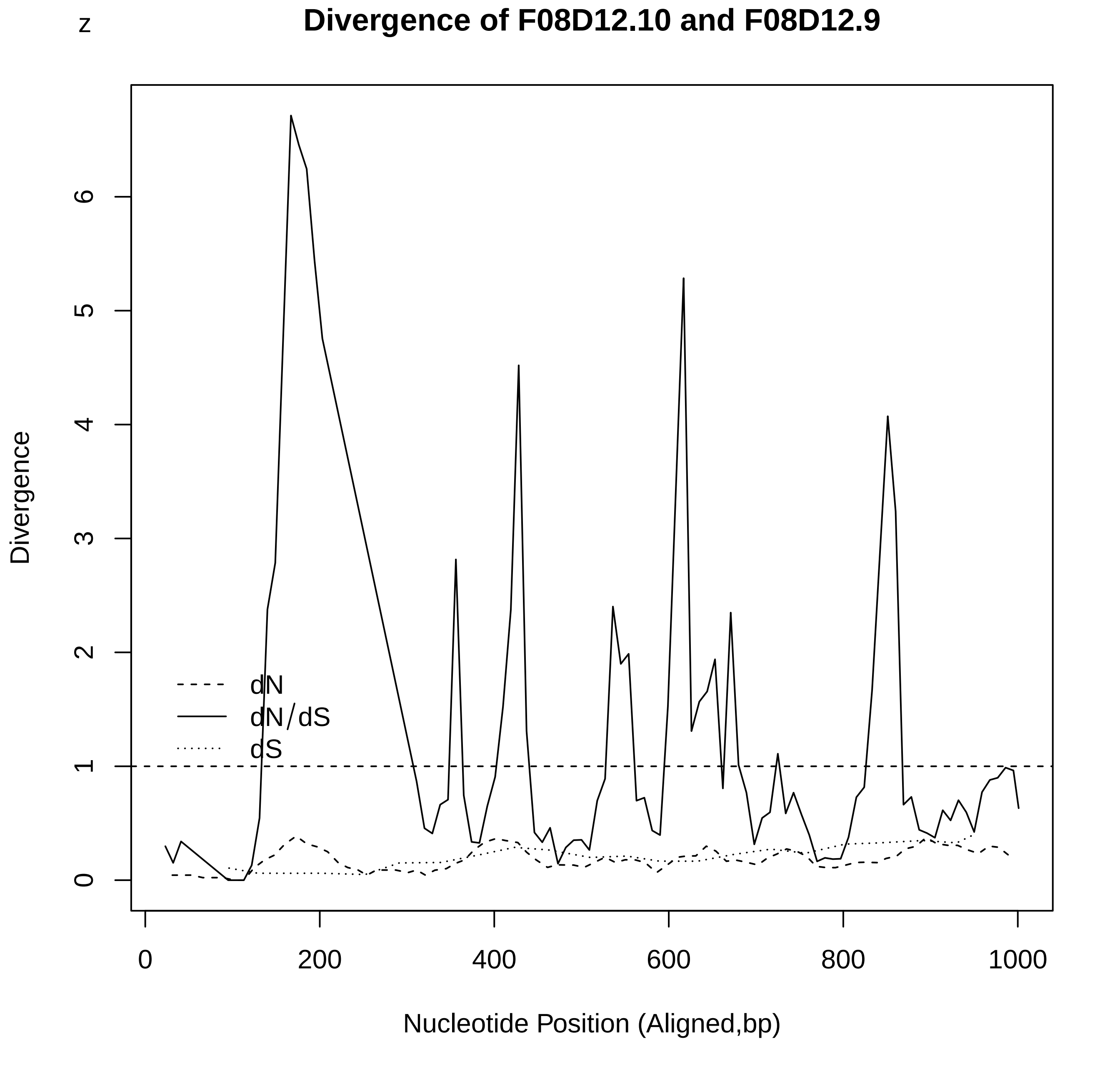
Divergence of F08D12.10 and F08D12.9
Divergence
0
1
2
3
4
5
6
dN
dN/dS
dS
0
200
400
600
800
1000
Nucleotide Position (Aligned,bp)

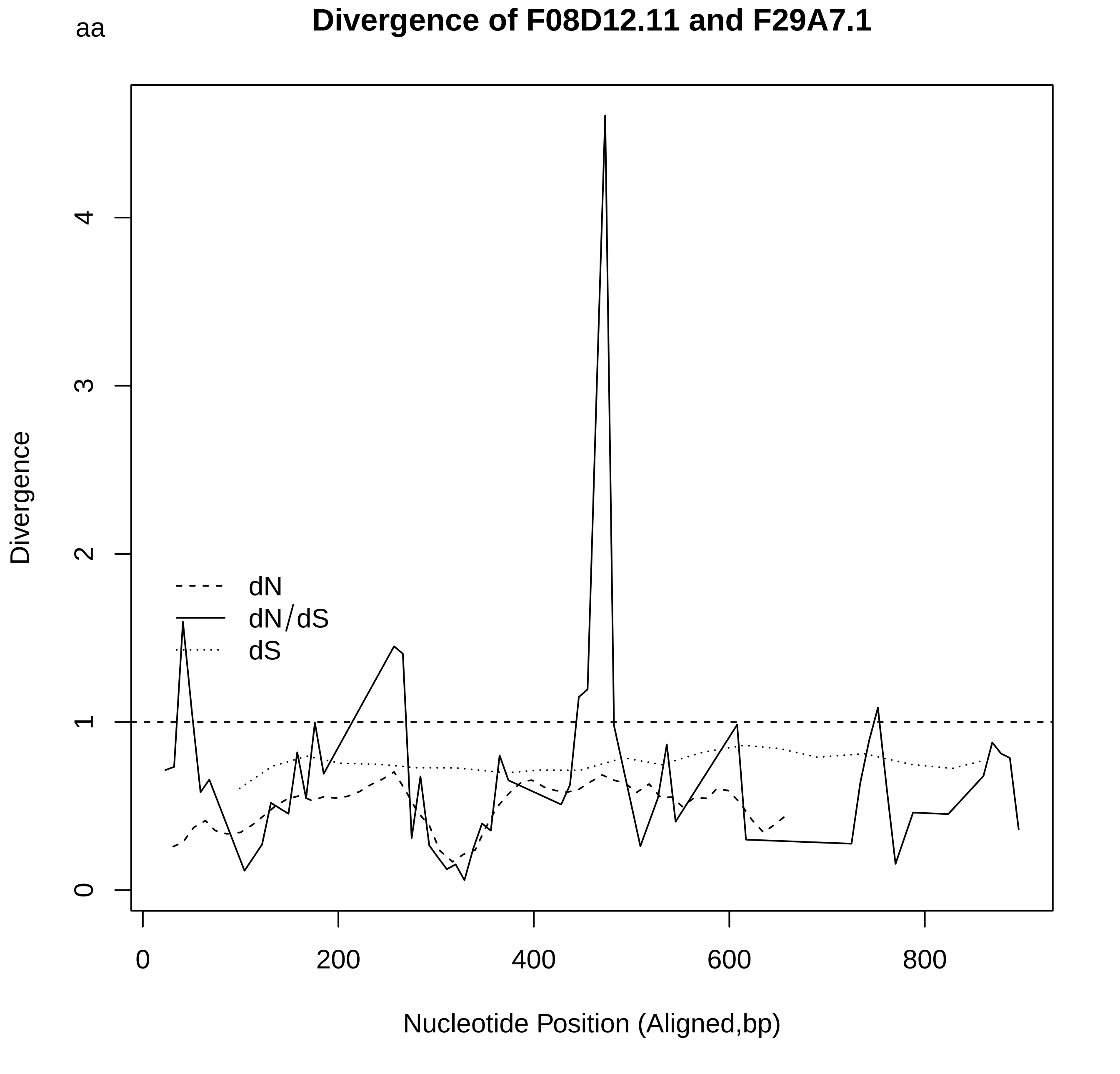
aa
Divergence of F08D12.11 and F29A7.1
Divergence
Nucleotide Position (Aligned,bp)
dN
dN/dS
dS
0
1
2
3
4
0
200
400
600
800

ab

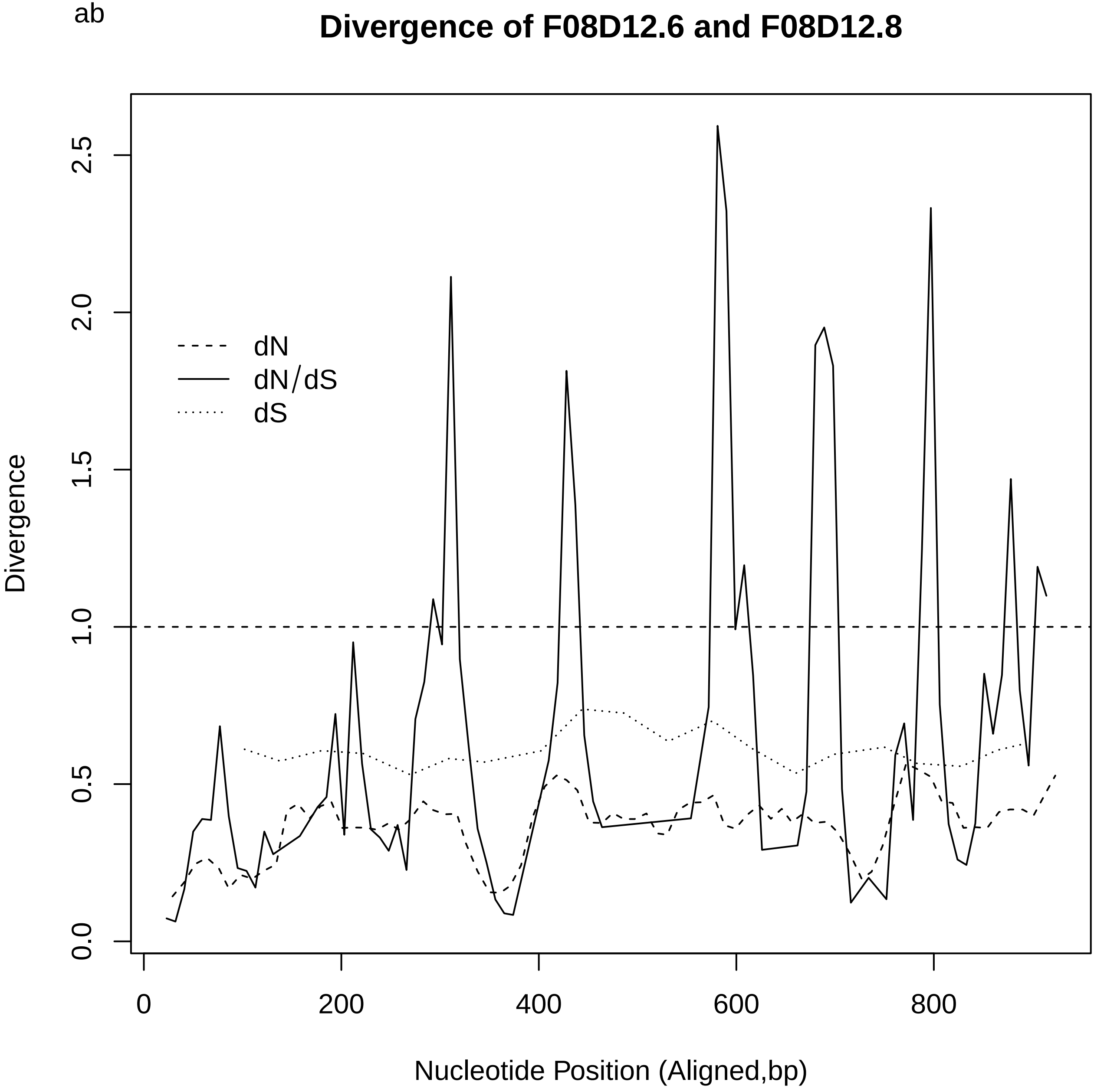
Divergence of F08D12.6 and F08D12.8
dN
dN/dS
dS
Divergence
0.0
0.5
1.0
1.5
2.0
2.5
0
200
400
600
800
Nucleotide Position (Aligned,bp)

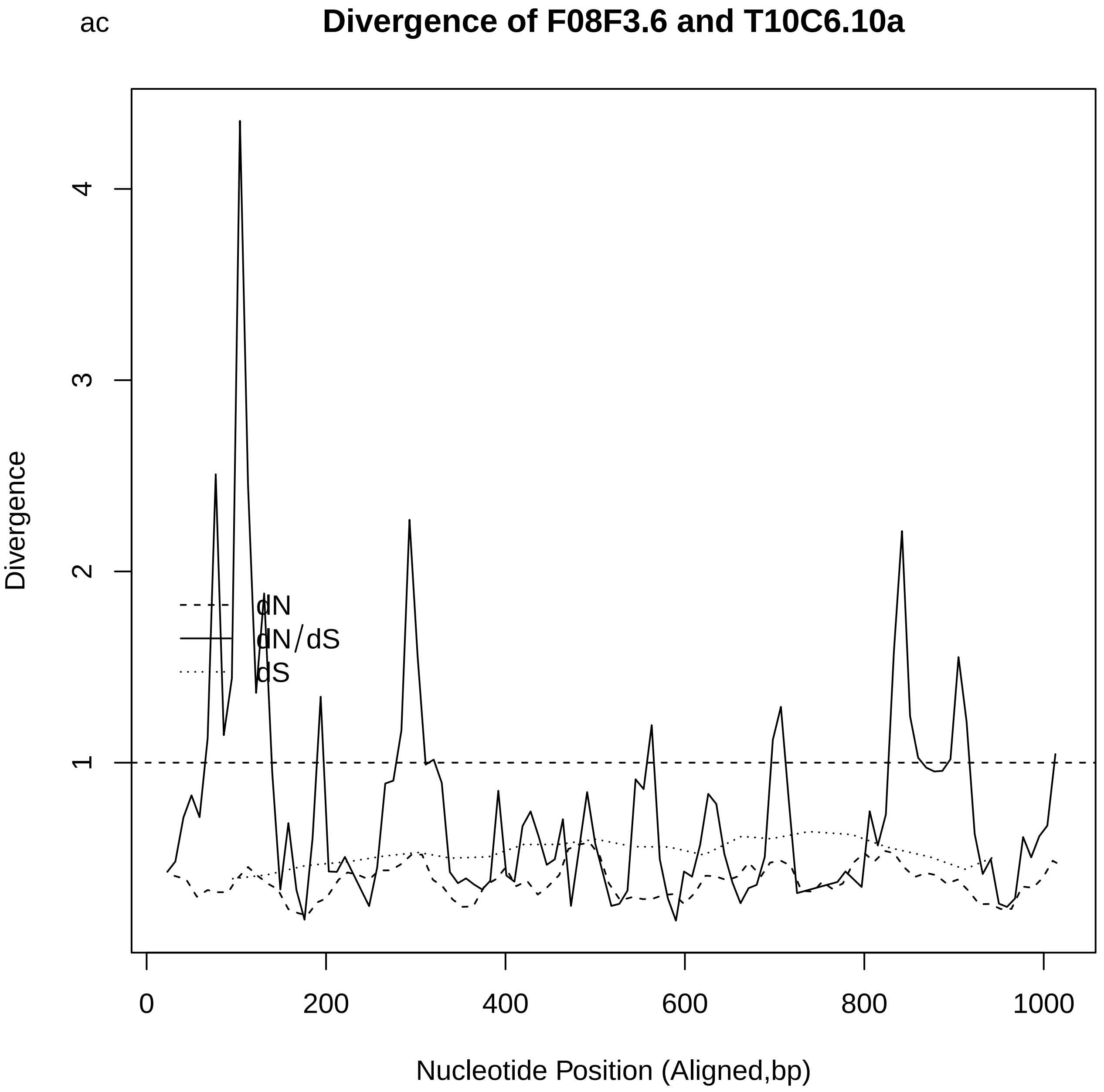
ac
Divergence of F08F3.6 and T10C6.10a
Divergence
1
2
3
4
dN
dN/dS
dS
0
200
400
600
800
1000
Nucleotide Position (Aligned,bp)

ad

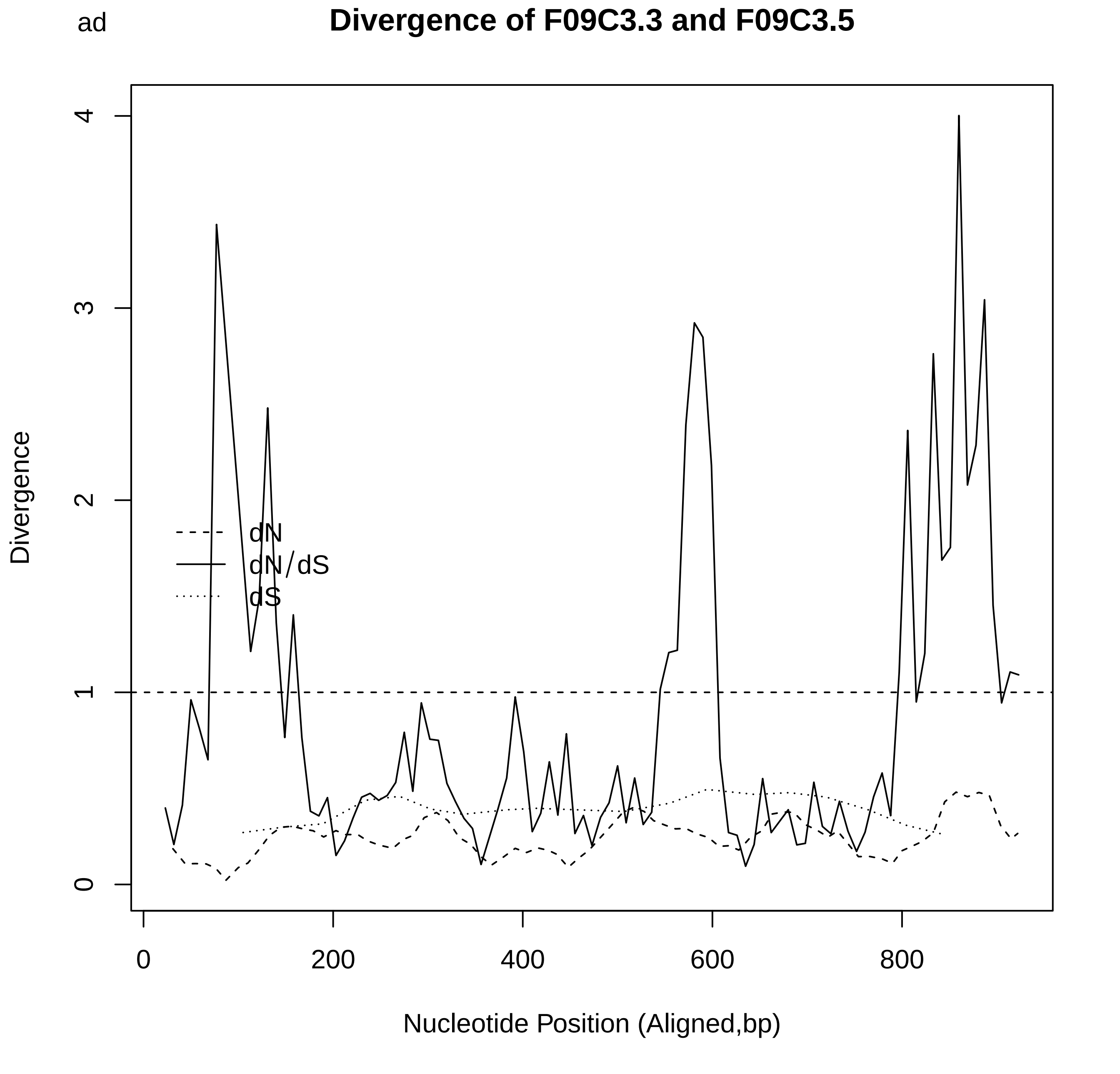

Divergence of F09C3.3 and F09C3.5
Divergence
Nucleotide Position (Aligned,bp)
dN
dN/dS
dS
0
1
2
3
4
0
200
400
600
800

ae

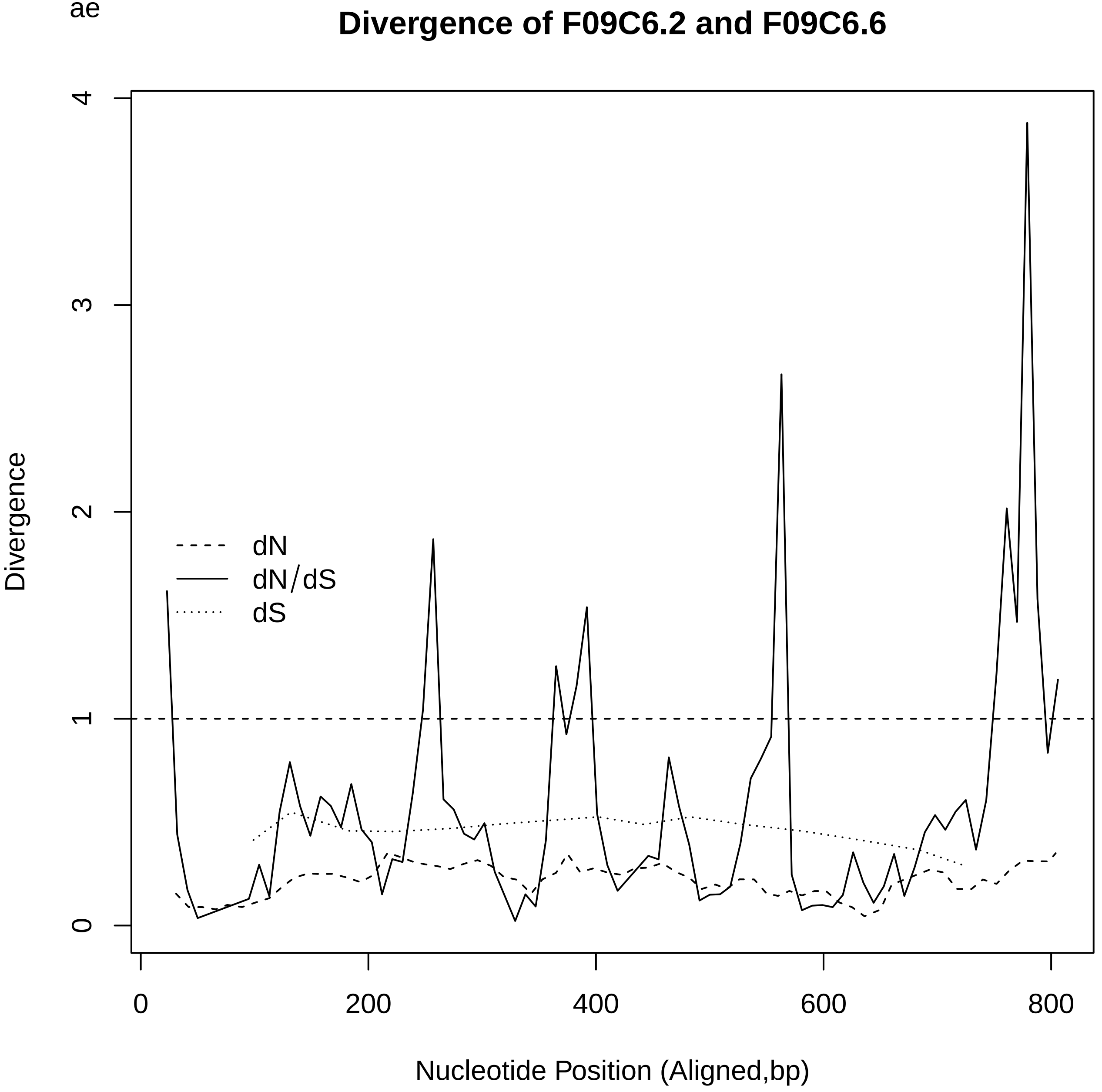
Divergence of F09C6.2 and F09C6.6
Divergence
Nucleotide Position (Aligned,bp)
dN
dN/dS
dS
0
1
2
3
4
0
200
400
600
800

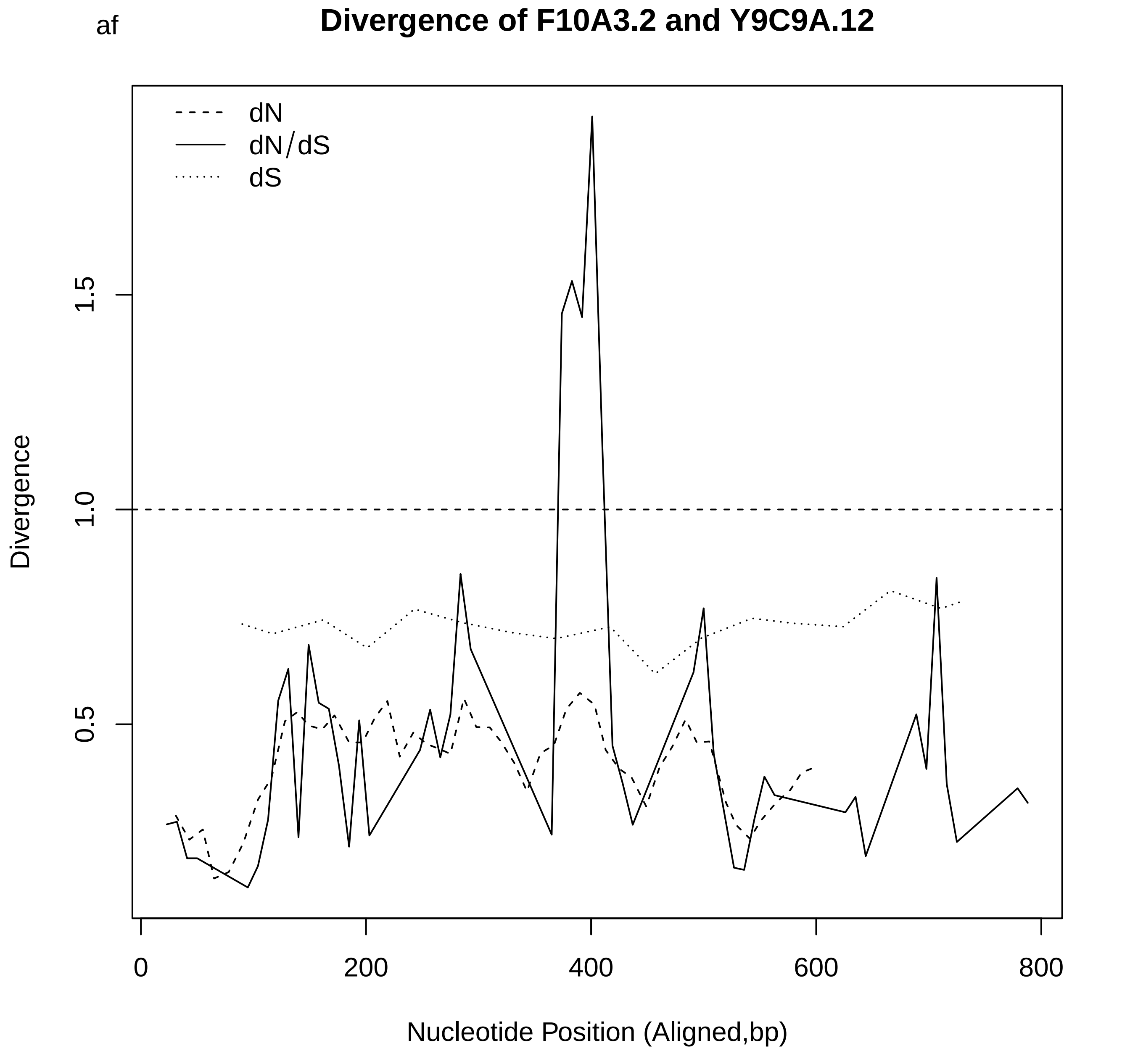

af
Divergence of F10A3.2 and Y9C9A.12
dN
dN/dS
dS
Divergence
0.5
1.0
1.5
0
200
400
600
800
Nucleotide Position (Aligned,bp)

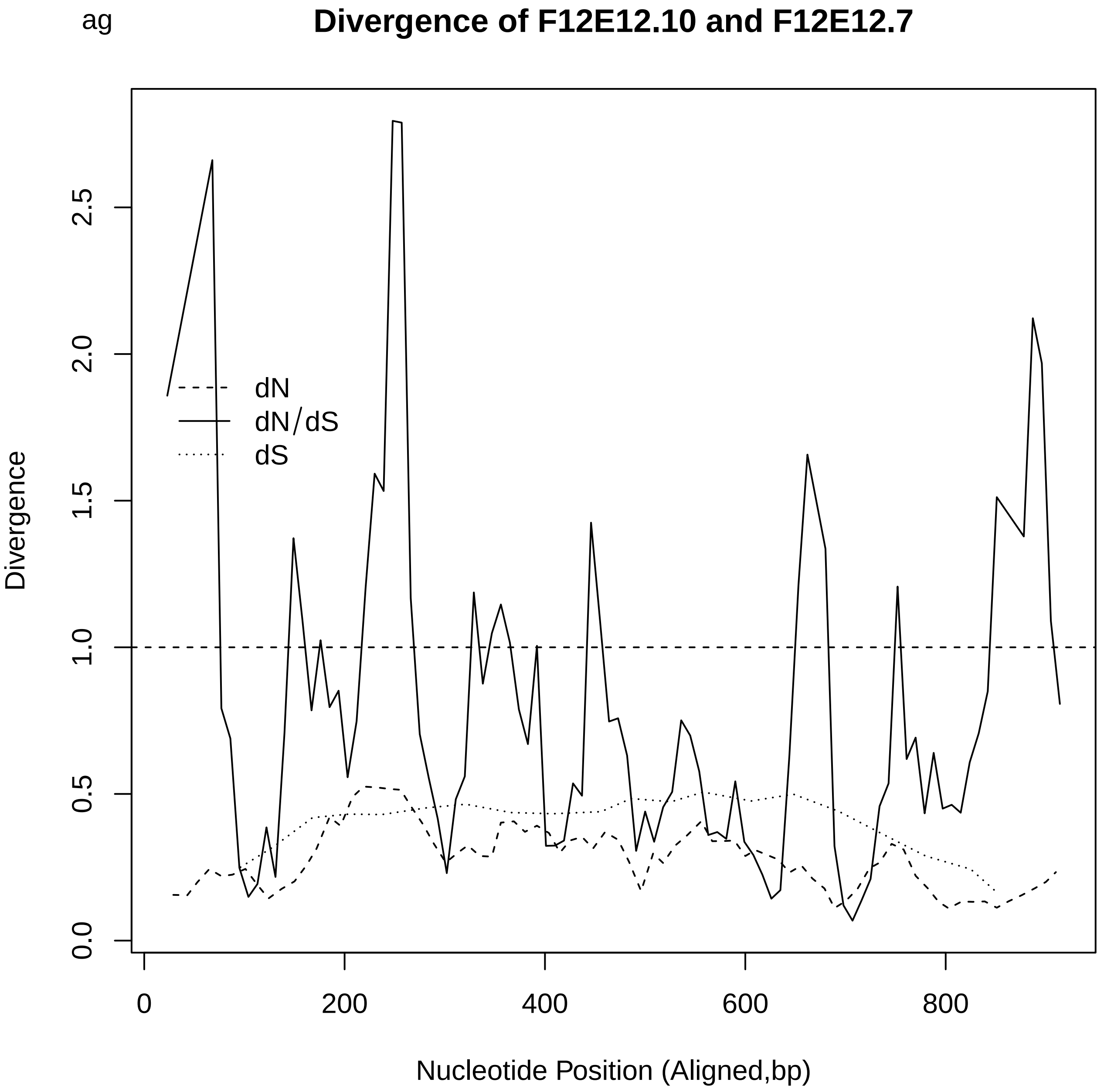
ag
Divergence of F12E12.10 and F12E12.7
dN
dN/dS
dS
Divergence
0.0
0.5
1.0
1.5
2.0
2.5
0
200
400
600
800
Nucleotide Position (Aligned,bp)

ah

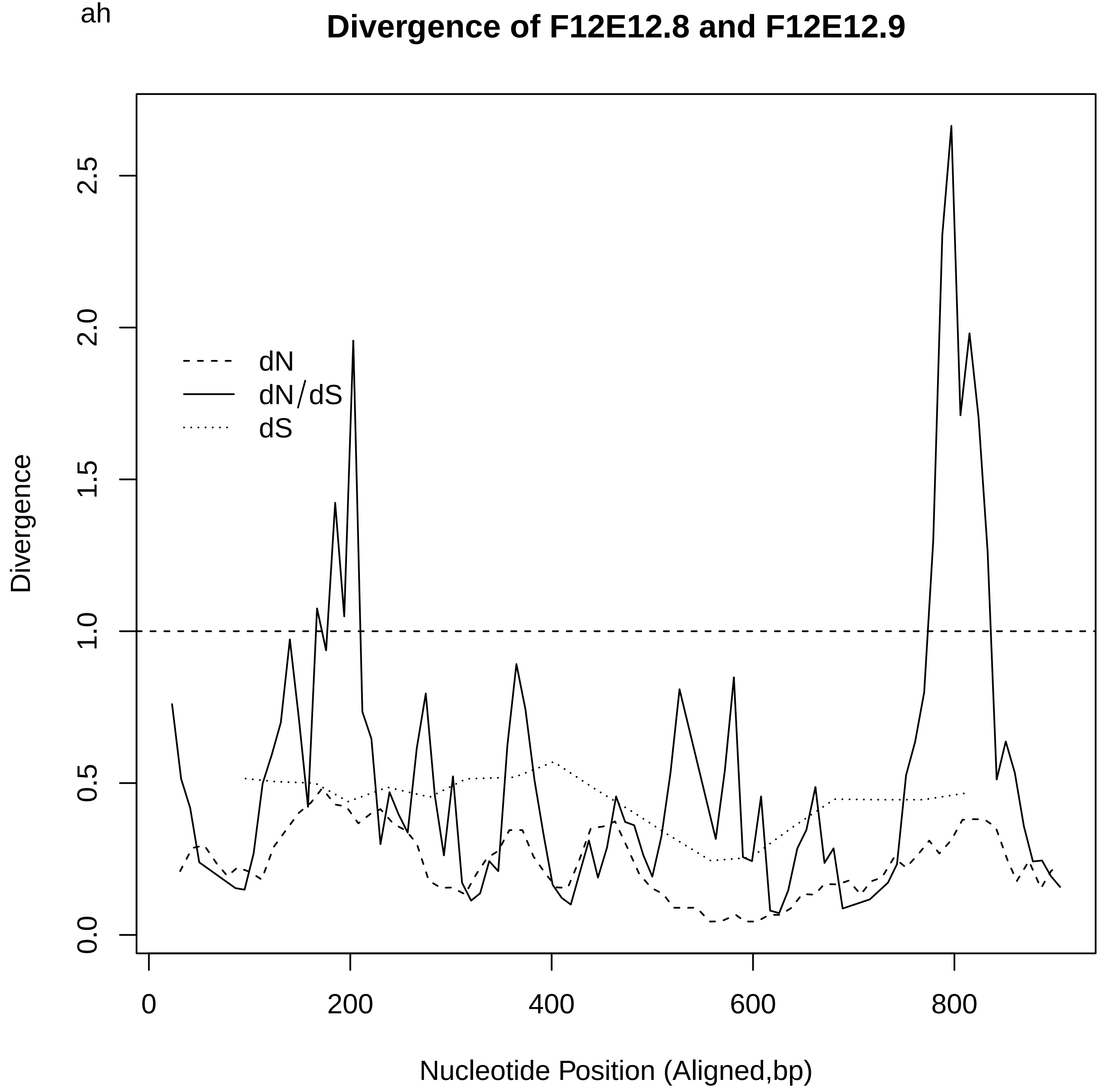

Divergence of F12E12.8 and F12E12.9
dN
dN/dS
dS
Divergence
0.0
0.5
1.0
1.5
2.0
2.5
0
200
400
600
800
Nucleotide Position (Aligned,bp)

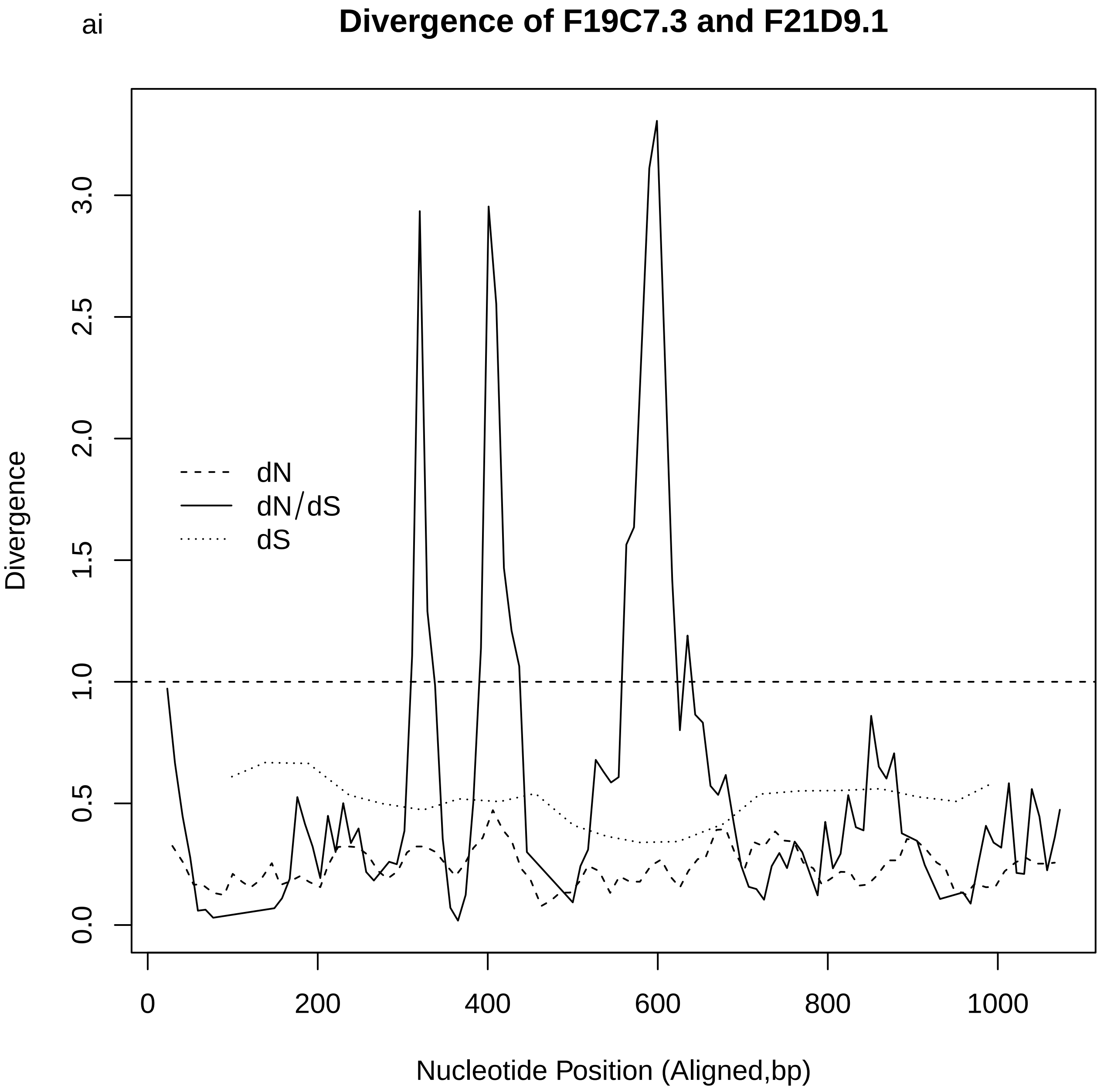
ai
Divergence of F19C7.3 and F21D9.1
Divergence
Nucleotide Position (Aligned,bp)
dN
dN/dS
dS
0.0
0.5
1.0
1.5
2.0
2.5
3.0
0
200
400
600
800
1000

aj

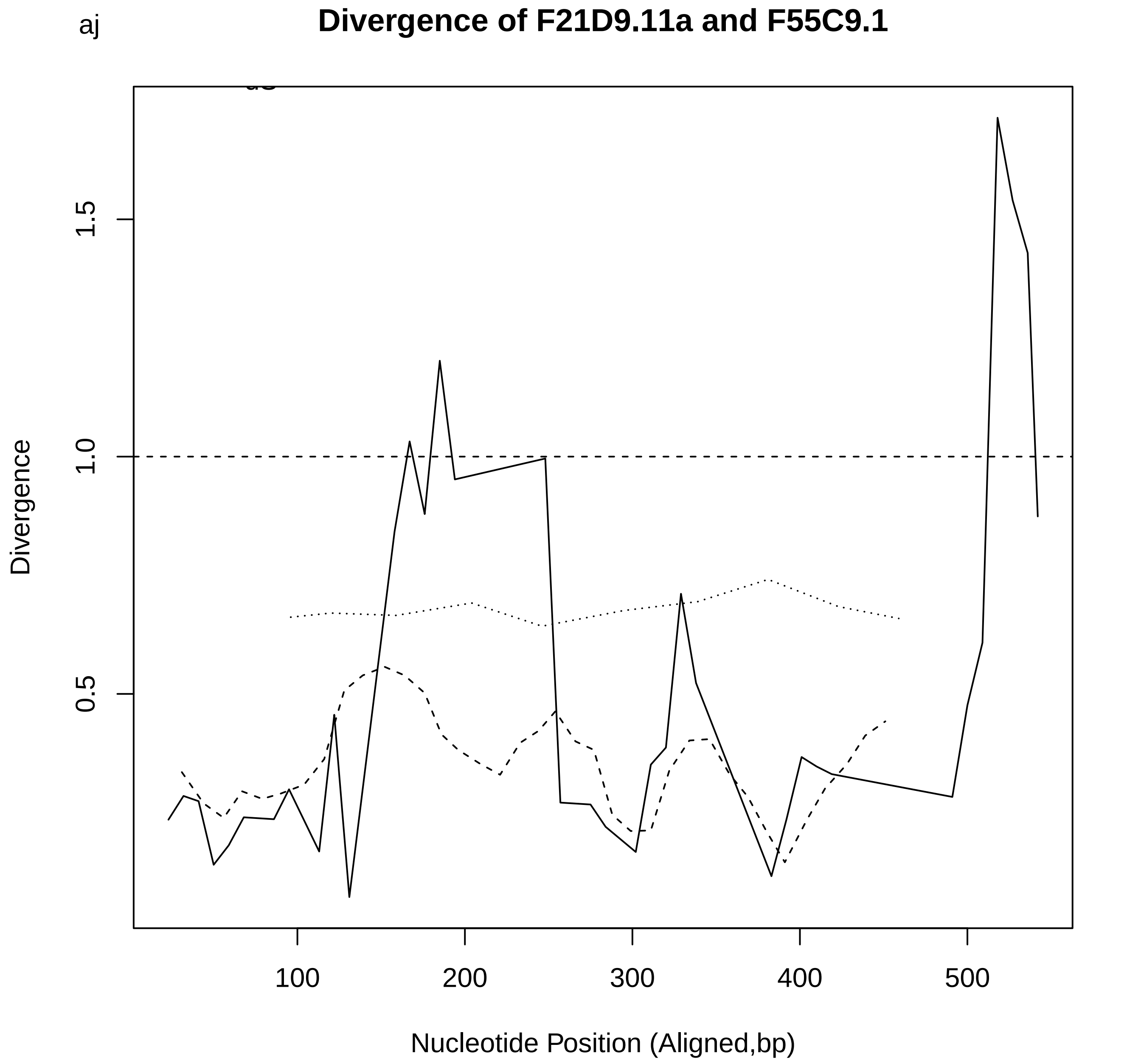
Divergence of F21D9.11a and F55C9.1
Divergence
1.5
1.0
0.5
100
200
300
400
500
Nucleotide Position (Aligned,bp)

ak

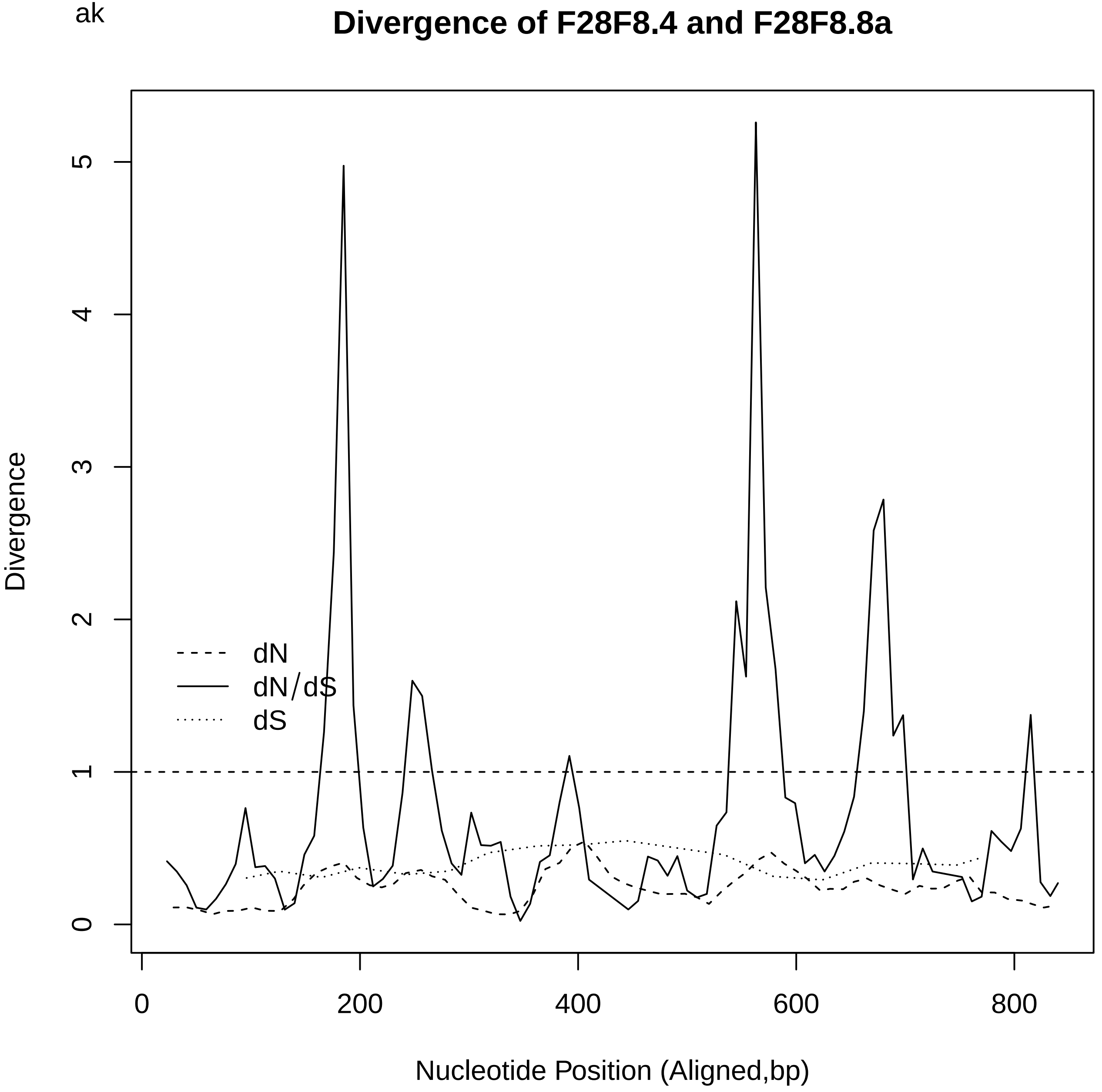

Divergence of F28F8.4 and F28F8.8a
Divergence
Nucleotide Position (Aligned,bp)
dN
dN/dS
dS
0
1
2
3
4
5
0
200
400
600
800

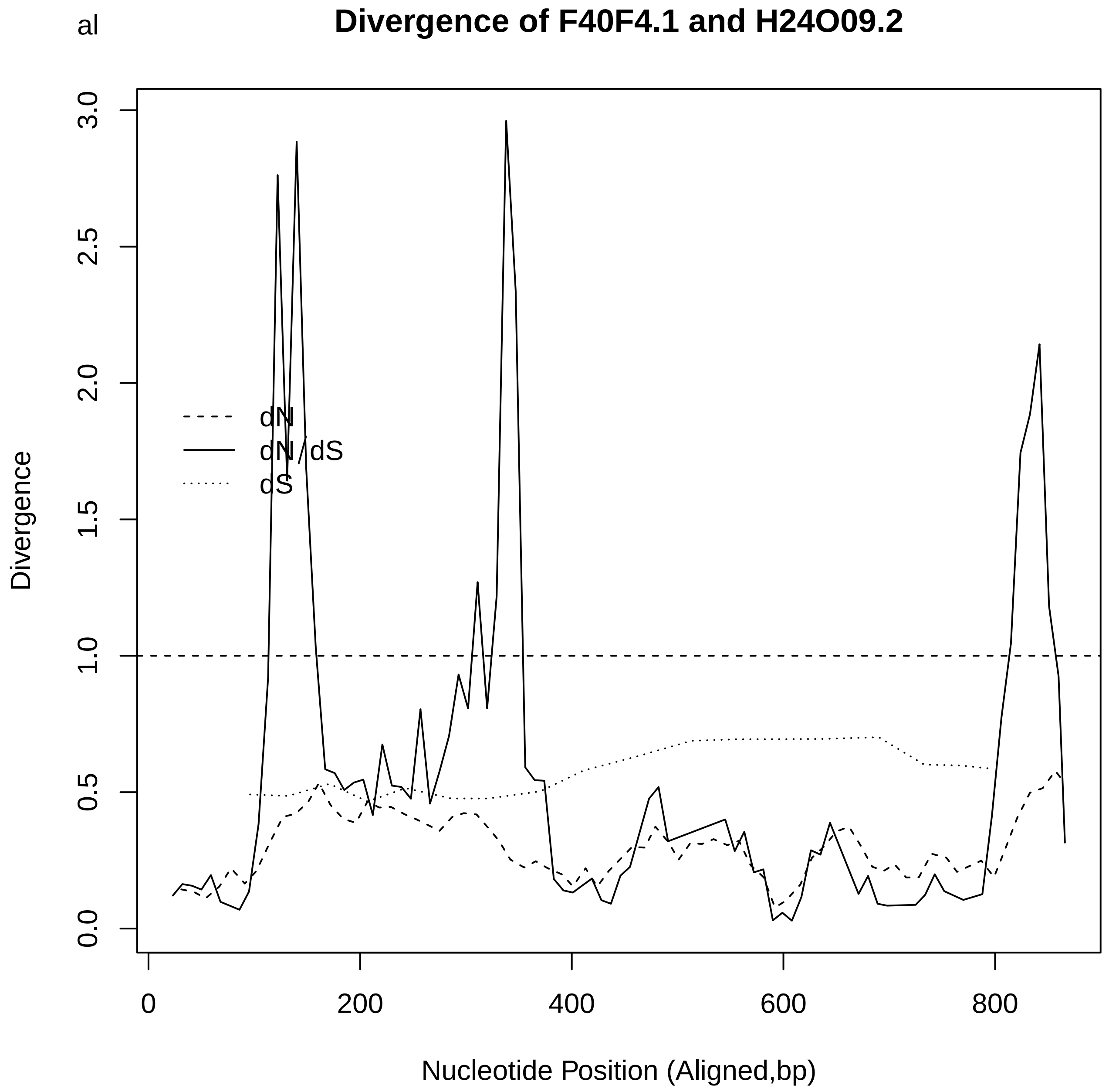
al
Divergence of F40F4.1 and H24O09.2
Divergence
Nucleotide Position (Aligned,bp)
dN
dN/dS
dS
0.0
0.5
1.0
1.5
2.0
2.5
3.0
0
200
400
600
800

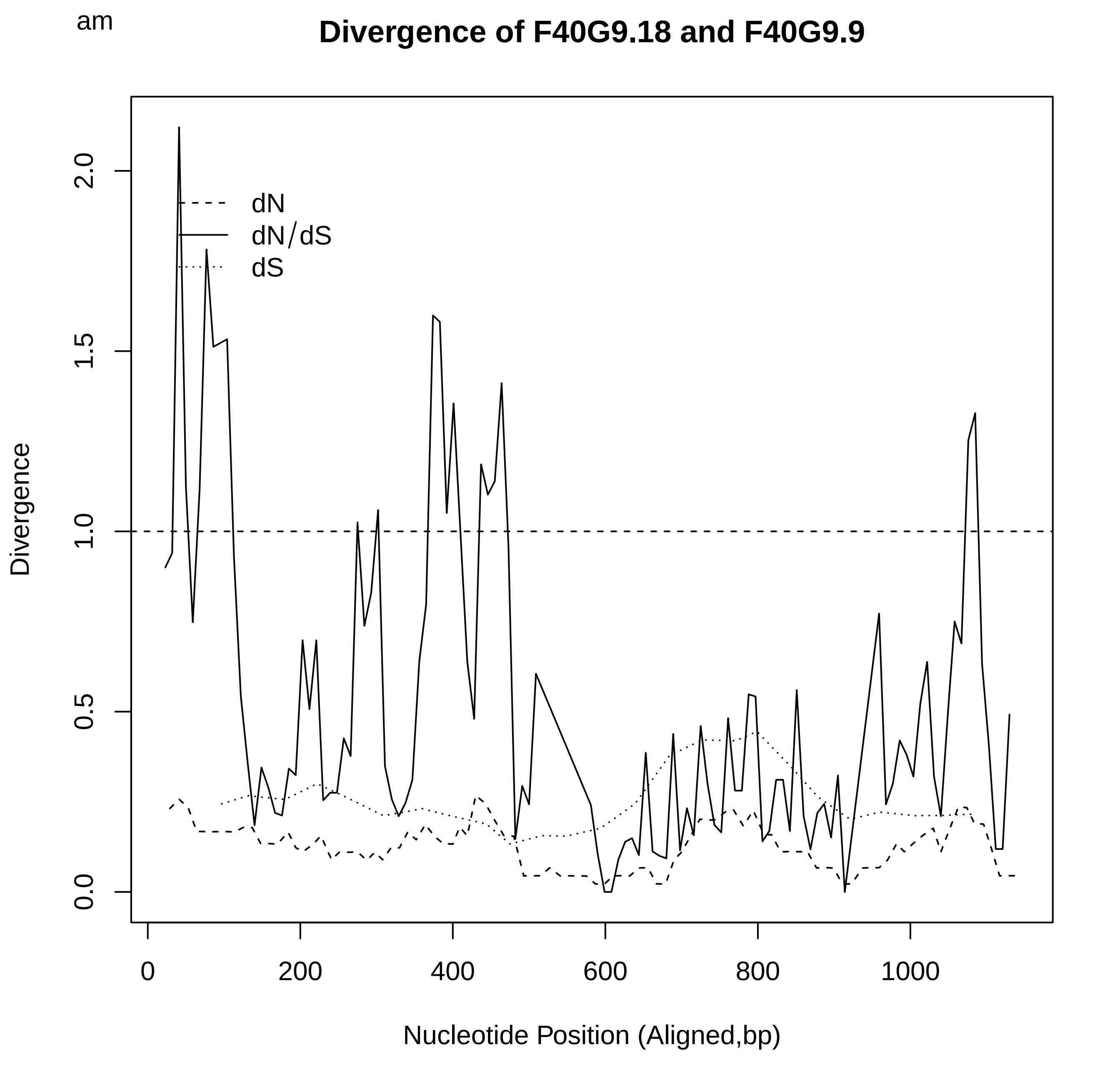
am
Divergence of F40G9.18 and F40G9.9
dN
dN/dS
dS
Divergence
0.0
0.5
1.0
1.5
2.0
0
200
400
600
800
1000
Nucleotide Position (Aligned,bp)

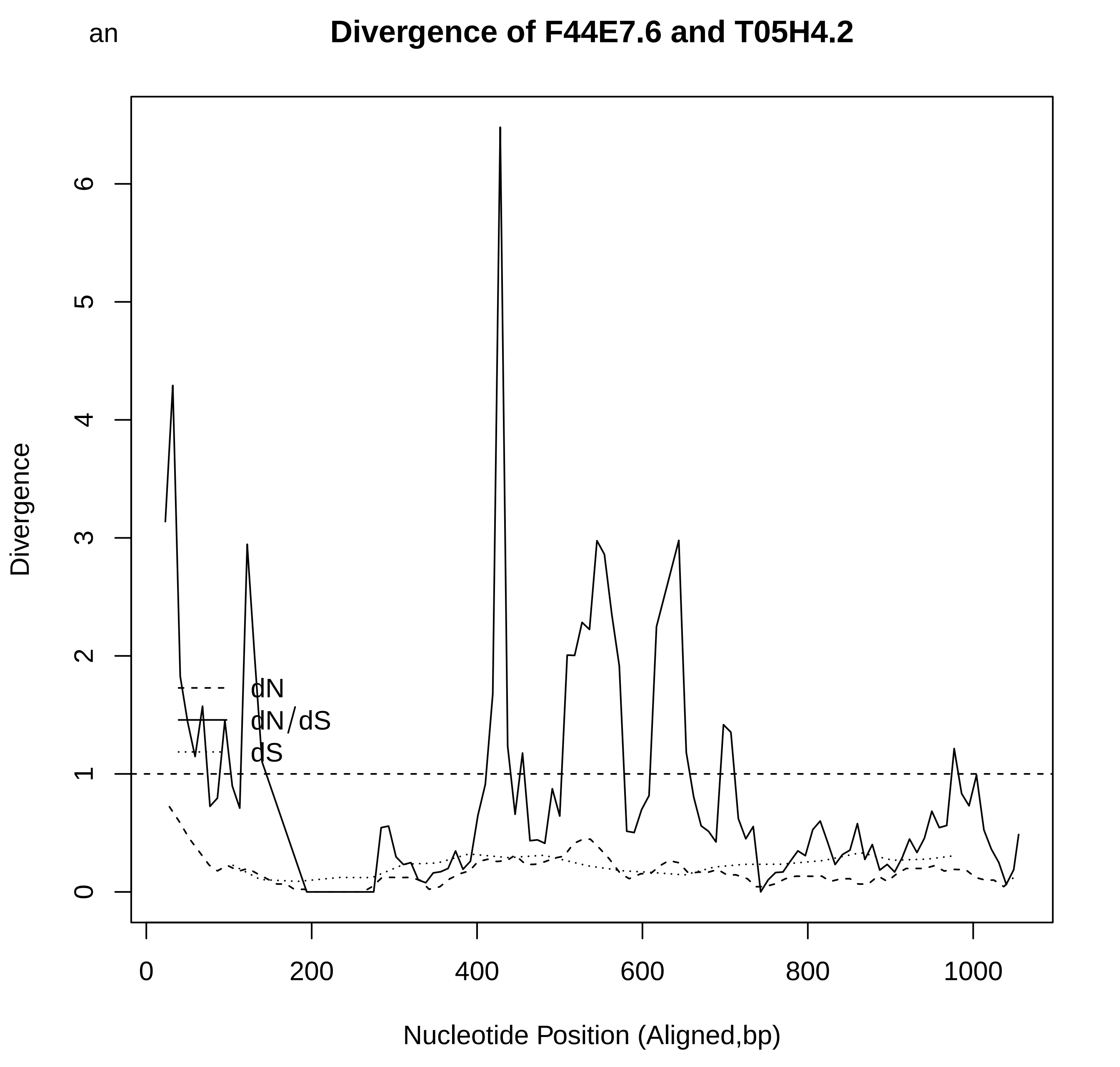
an
Divergence of F44E7.6 and T05H4.2
Divergence
0
1
2
3
4
5
6
dN
dN/dS
dS
0
200
400
600
800
1000
Nucleotide Position (Aligned,bp)

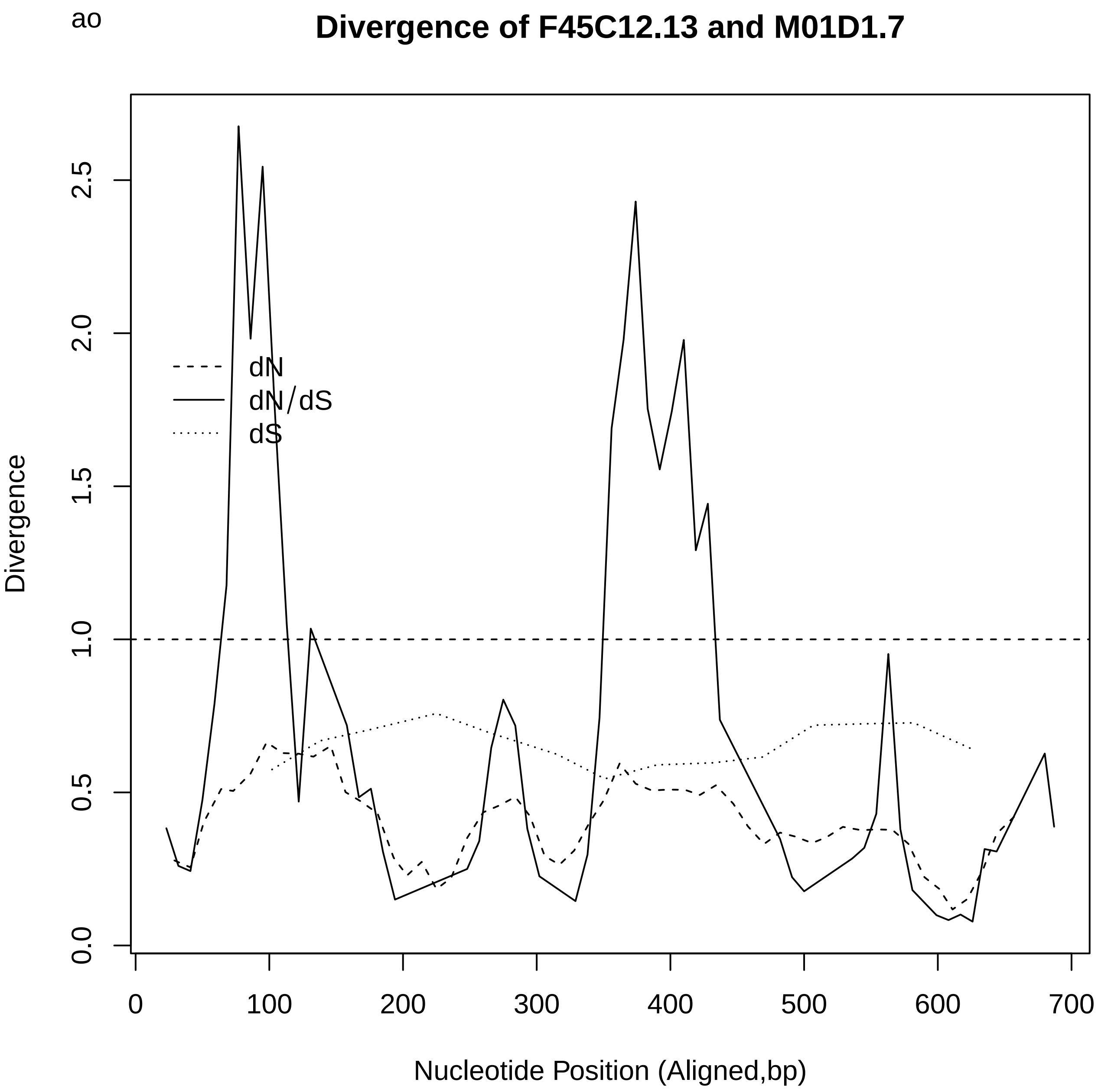
ao
Divergence of F45C12.13 and M01D1.7
Divergence
Nucleotide Position (Aligned,bp)
dN
dN/dS
dS
0.0
0.5
1.0
1.5
2.0
2.5
0
100
200
300
400
500
600
700

ap

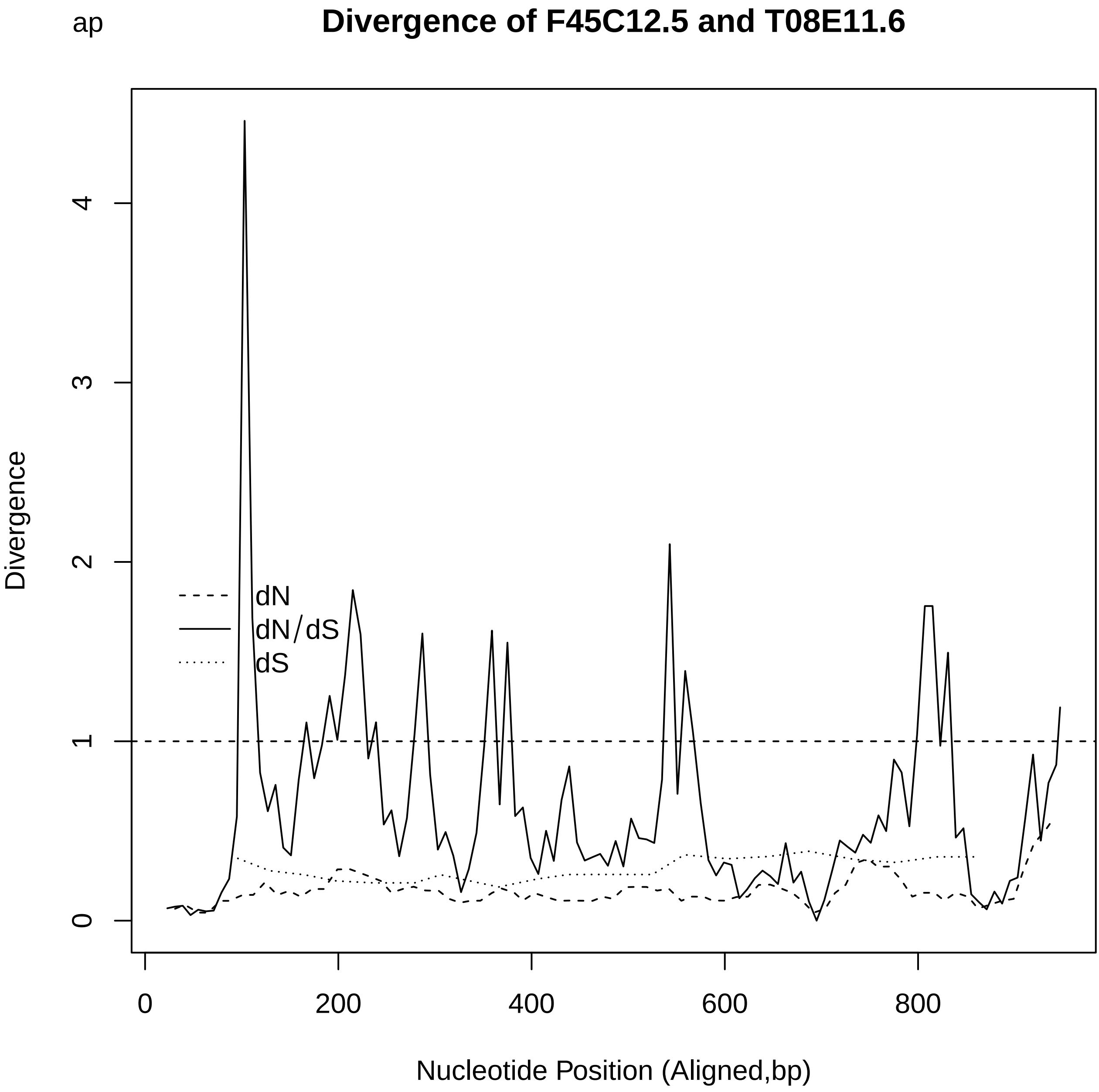

Divergence of F45C12.5 and T08E11.6
Divergence
0
1
2
3
4
dN
dN/dS
dS
0
200
400
600
800
Nucleotide Position (Aligned,bp)

aq

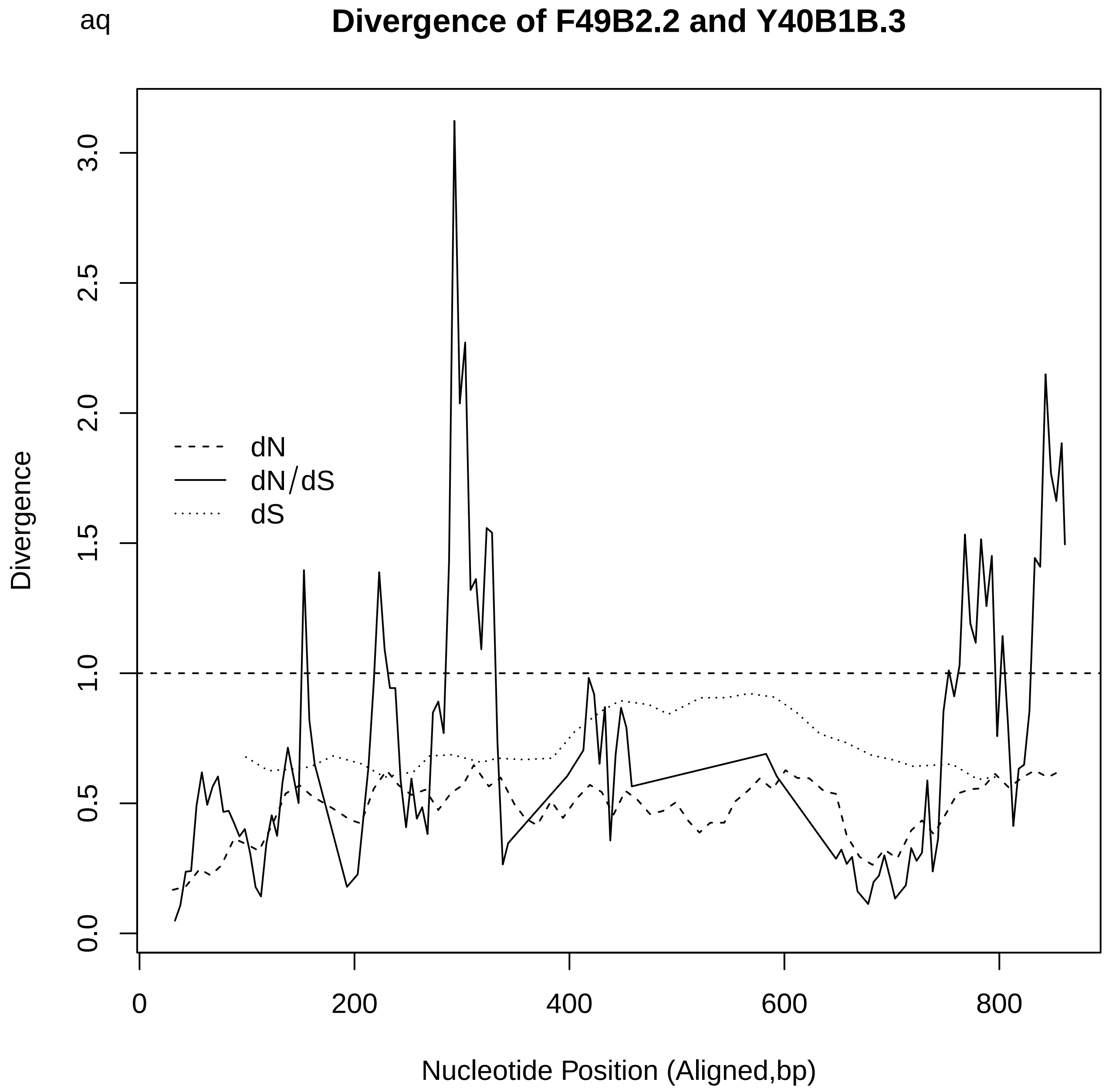
Divergence of F49B2.2 and Y40B1B.3
Divergence
Nucleotide Position (Aligned,bp)
dN
dN/dS
dS
0.0
0.5
1.0
1.5
2.0
2.5
3.0
0
200
400
600
800

ar

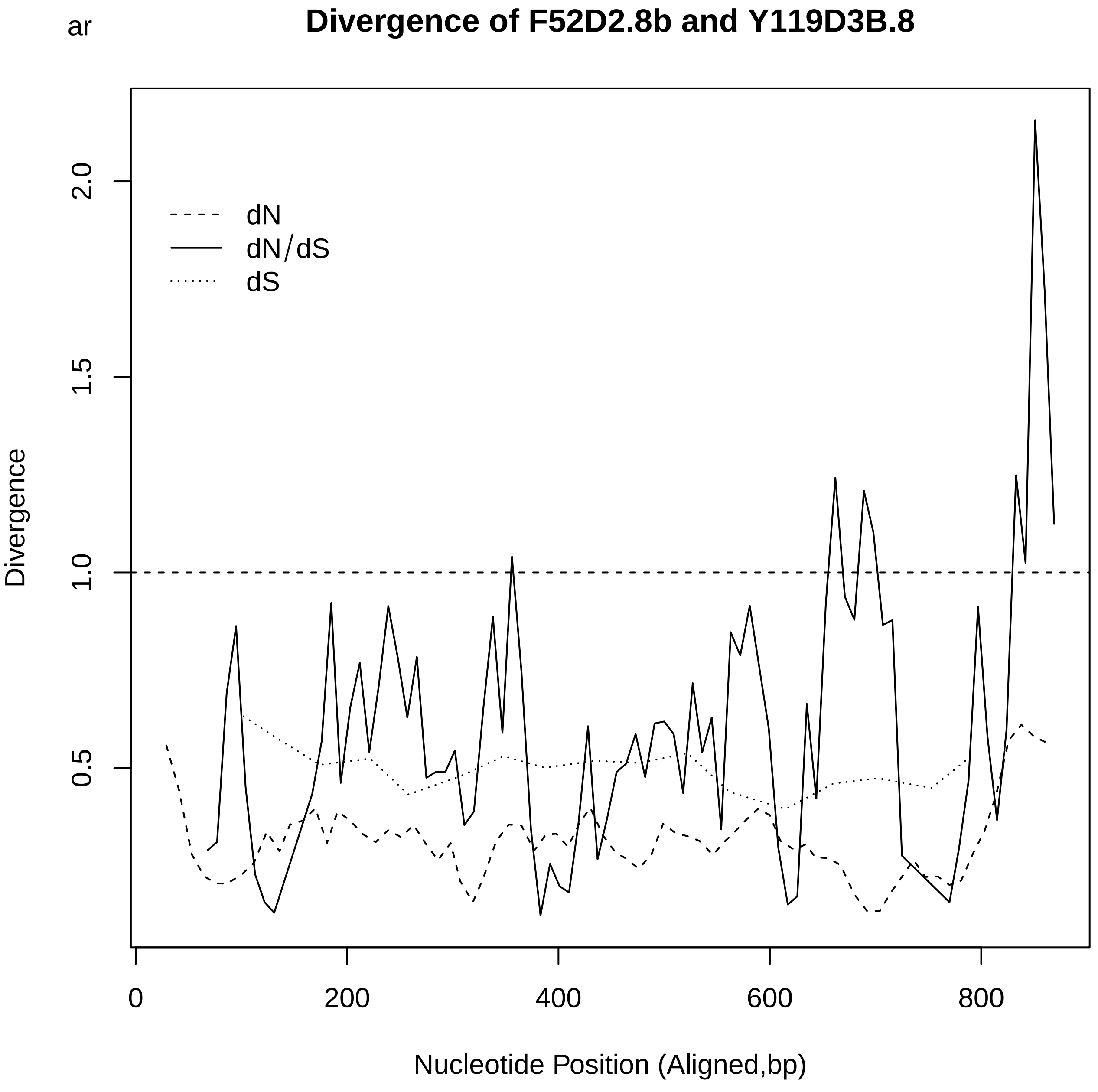

Divergence of F52D2.8b and Y119D3B.8
dN
dN/dS
dS
Divergence
0.5
1.0
1.5
2.0
0
200
400
600
800
Nucleotide Position (Aligned,bp)

as

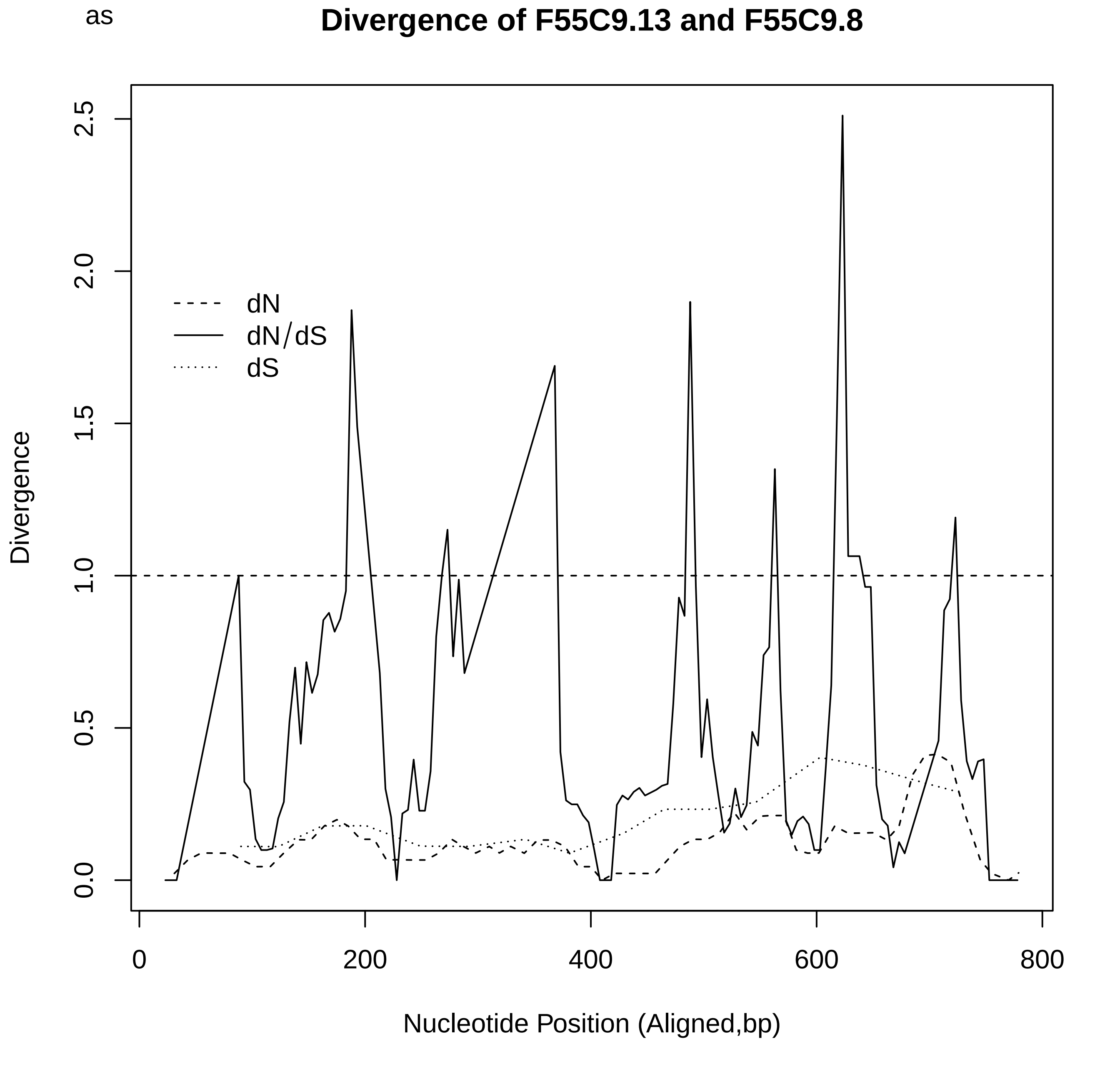

Divergence of F55C9.13 and F55C9.8
Divergence
Nucleotide Position (Aligned,bp)
dN
dN/dS
dS
0.0
0.5
1.0
1.5
2.0
2.5
0
200
400
600
800

at

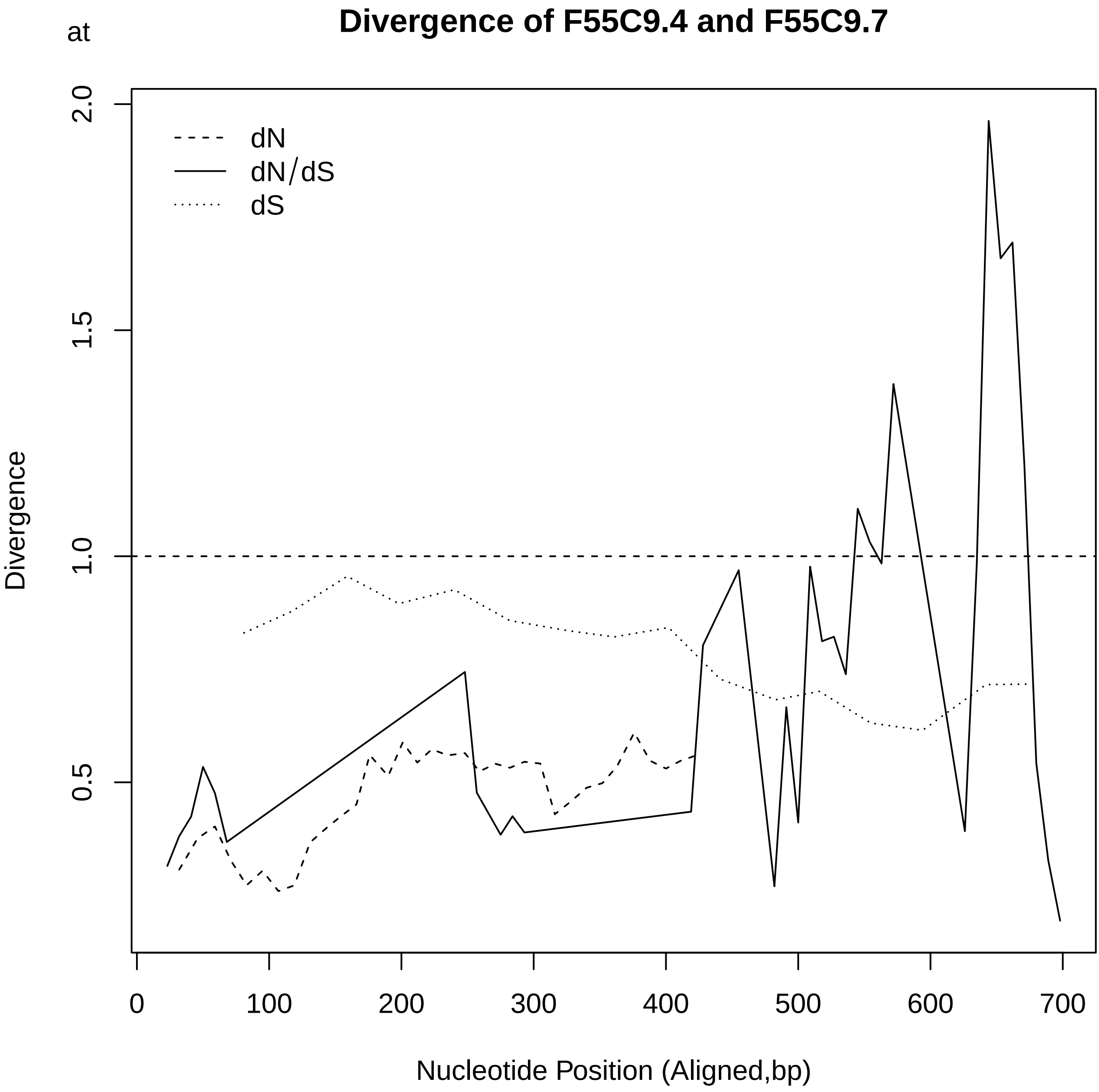

Divergence of F55C9.4 and F55C9.7
dN
dN/dS
dS
Divergence
2.0
1.5
1.0
0.5
0
100
200
300
400
500
600
700
Nucleotide Position (Aligned,bp)

au

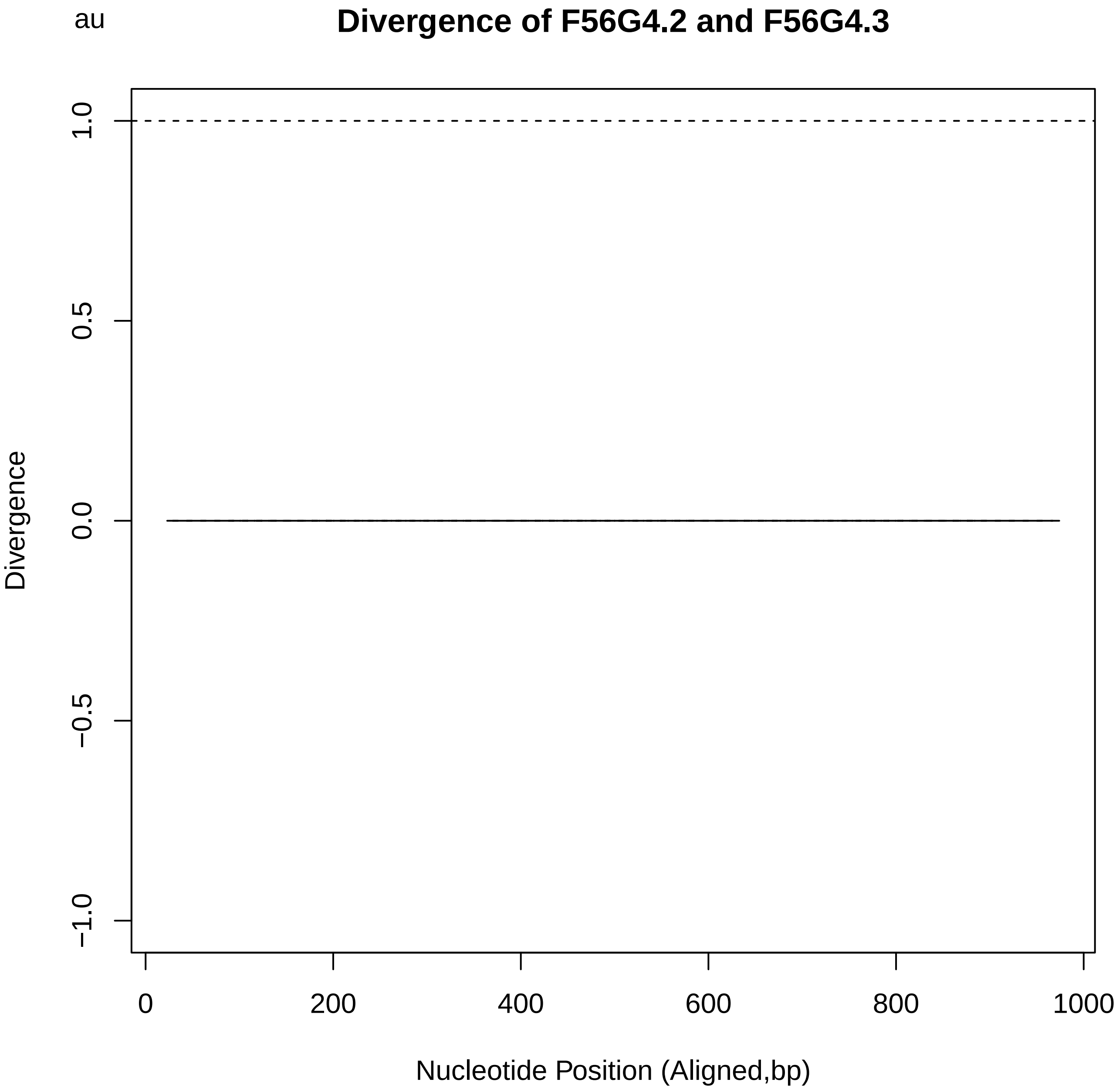

Divergence of F56G4.2 and F56G4.3
Divergence
1.0
0.5
0.0
−0.5
−1.0
0
200
400
600
800
1000
Nucleotide Position (Aligned,bp)

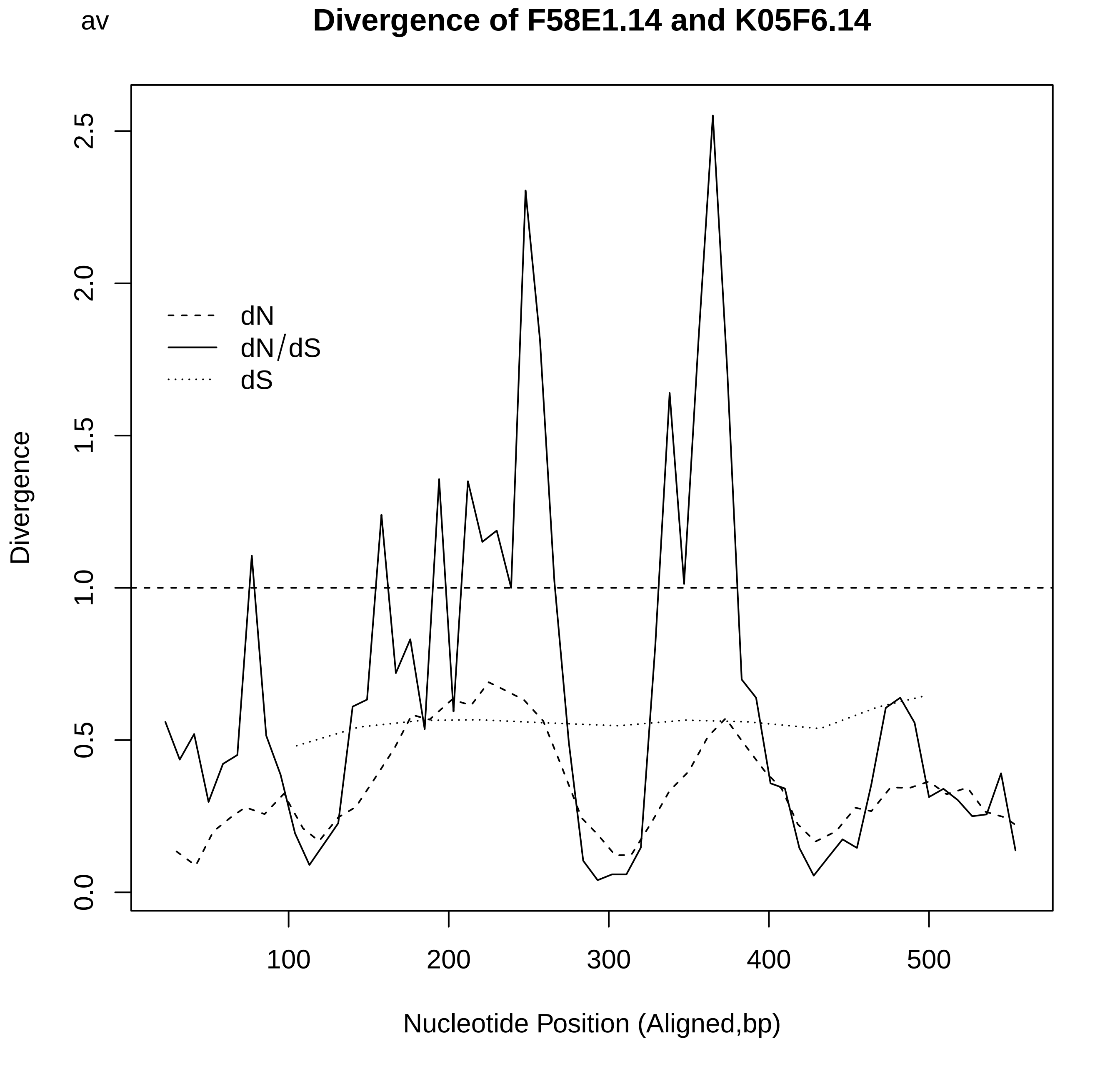
av
Divergence of F58E1.14 and K05F6.14
dN
dN/dS
dS
Divergence
0.0
0.5
1.0
1.5
2.0
2.5
100
200
300
400
500
Nucleotide Position (Aligned,bp)

aw

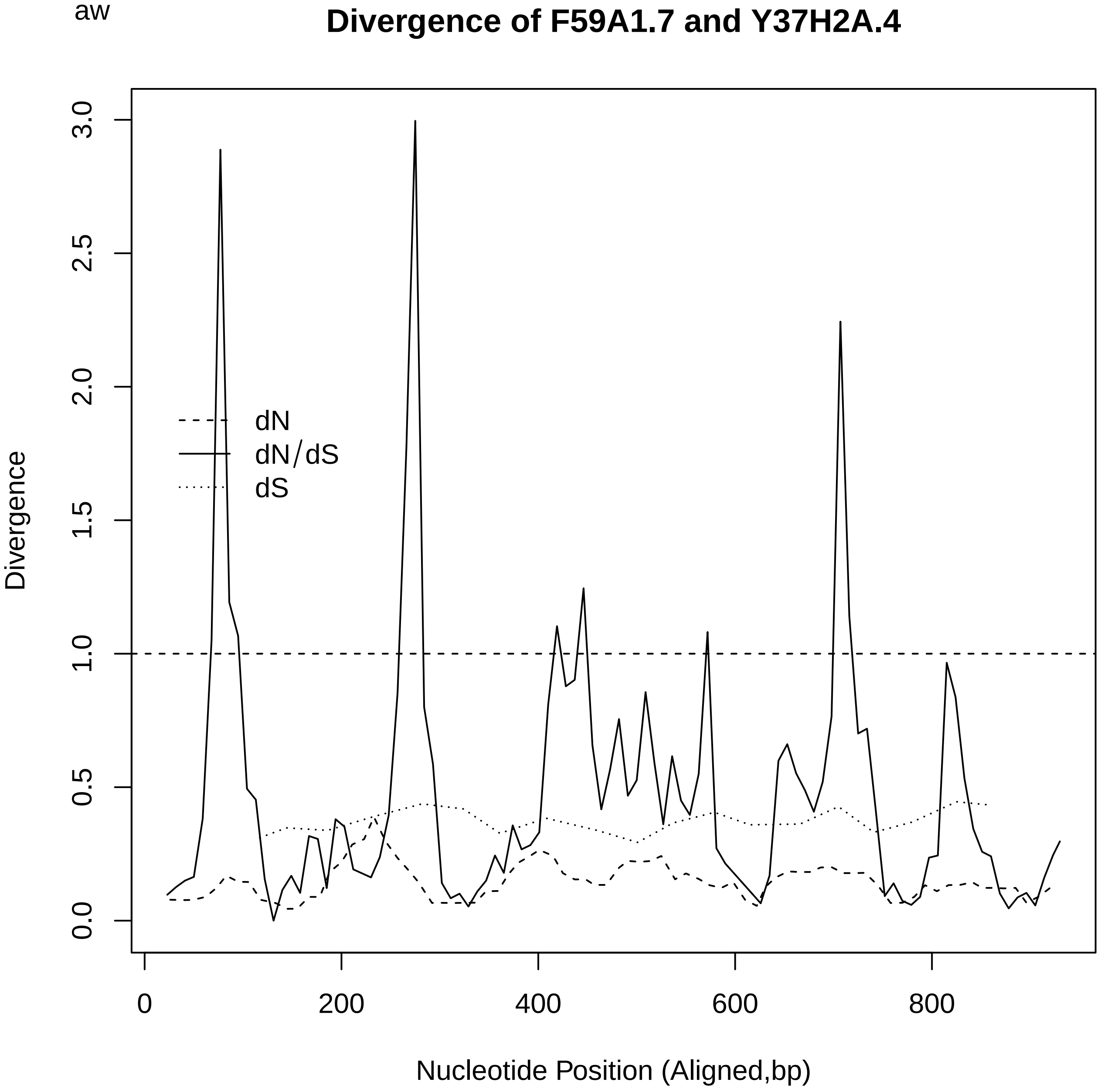

Divergence of F59A1.7 and Y37H2A.4
Divergence
Nucleotide Position (Aligned,bp)
dN
dN/dS
dS
0.0
0.5
1.0
1.5
2.0
2.5
3.0
0
200
400
600
800

ax

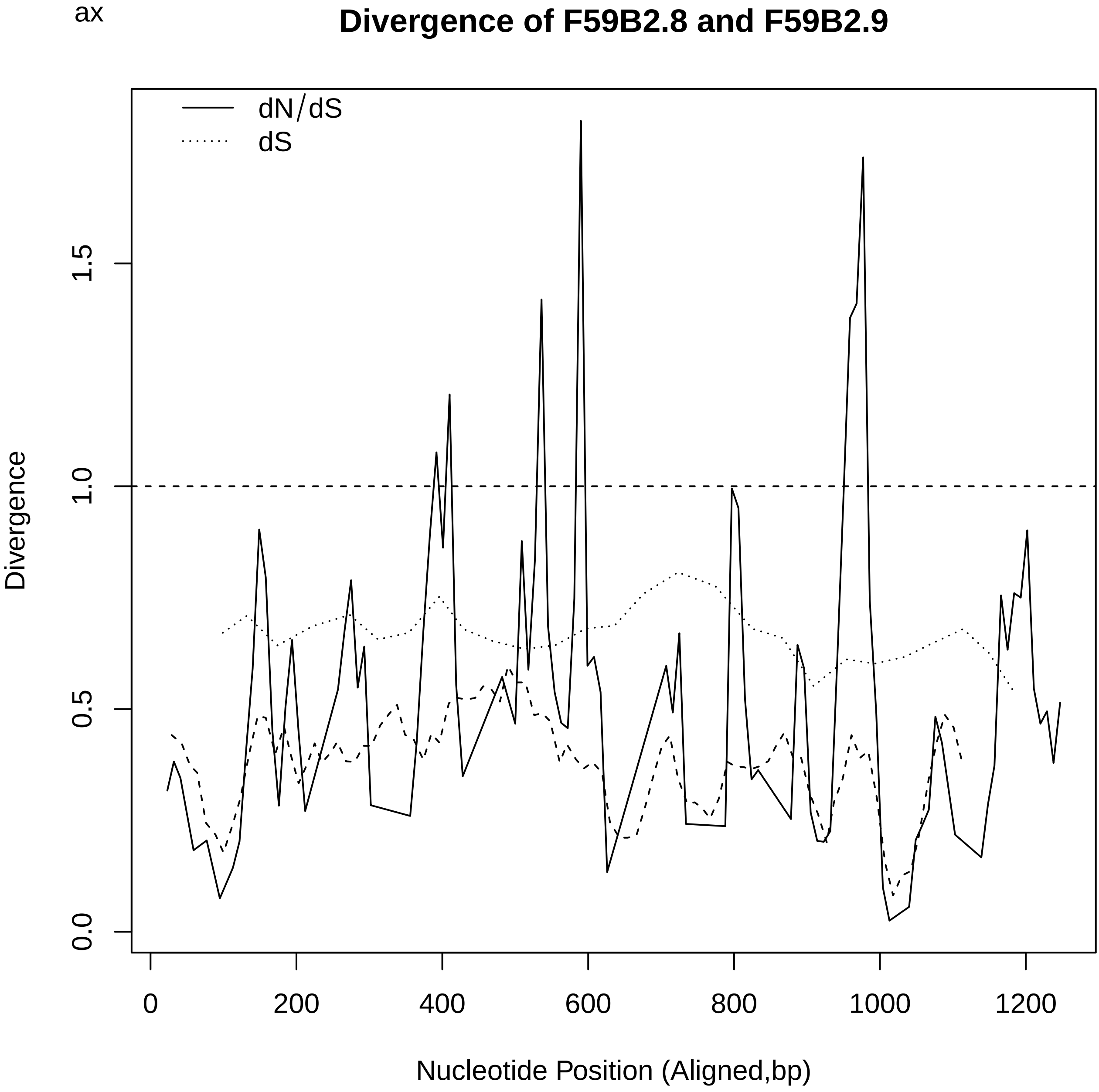
Divergence of F59B2.8 and F59B2.9
dN/dS
dS
Divergence
0.0
0.5
1.0
1.5
0
200
400
600
800
1000
1200
Nucleotide Position (Aligned,bp)

ay

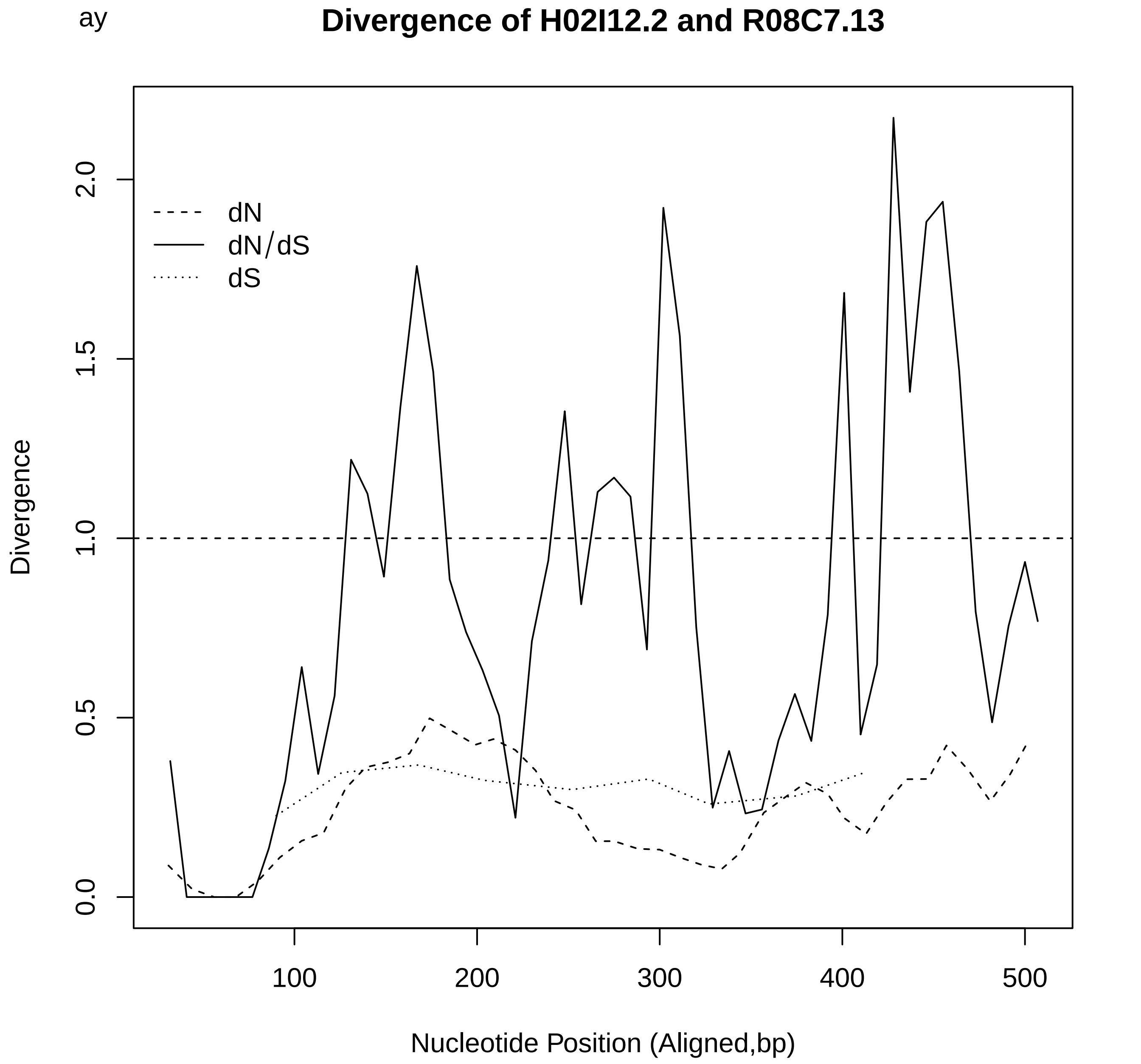

Divergence of H02I12.2 and R08C7.13
dN
dN/dS
dS
Divergence
0.0
0.5
1.0
1.5
2.0
100
200
300
400
500
Nucleotide Position (Aligned,bp)

az

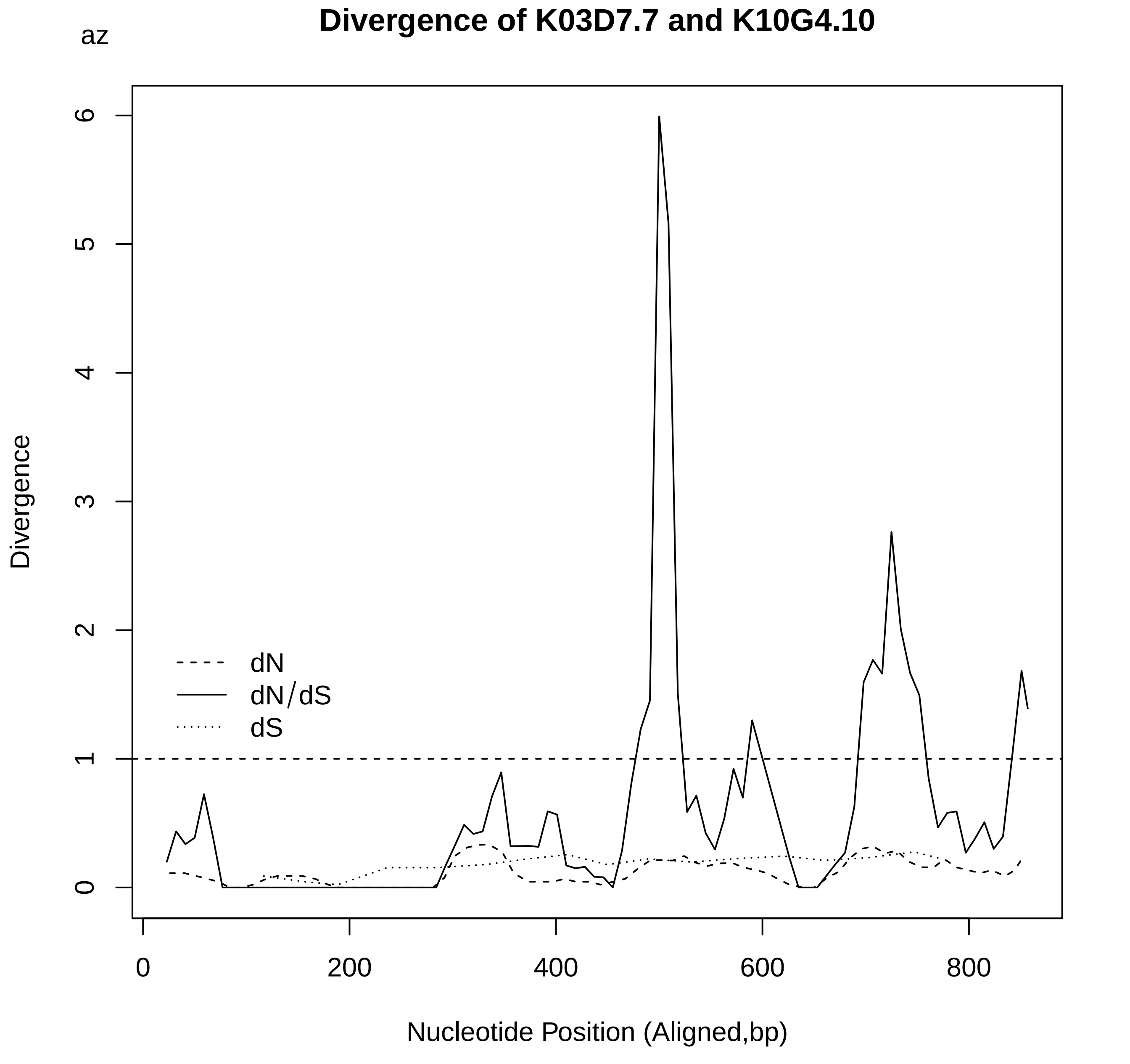

Divergence of K03D7.7 and K10G4.10
Divergence
Nucleotide Position (Aligned,bp)
dN
dN/dS
dS
0
1
2
3
4
5
6
0
200
400
600
800

ba

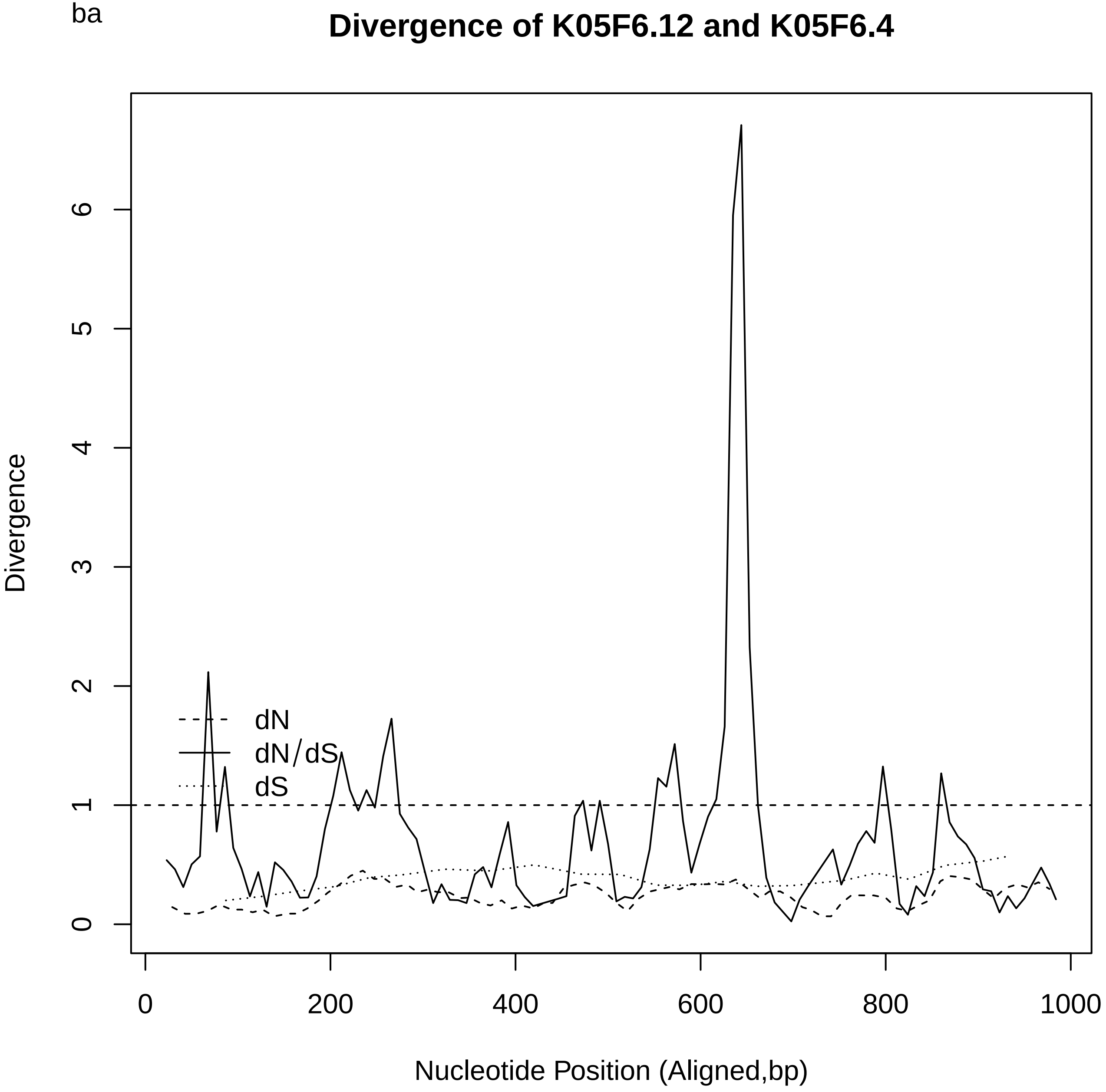
Divergence of K05F6.12 and K05F6.4
Divergence
0
1
2
3
4
5
6
dN
dN/dS
dS
0
200
400
600
800
1000
Nucleotide Position (Aligned,bp)

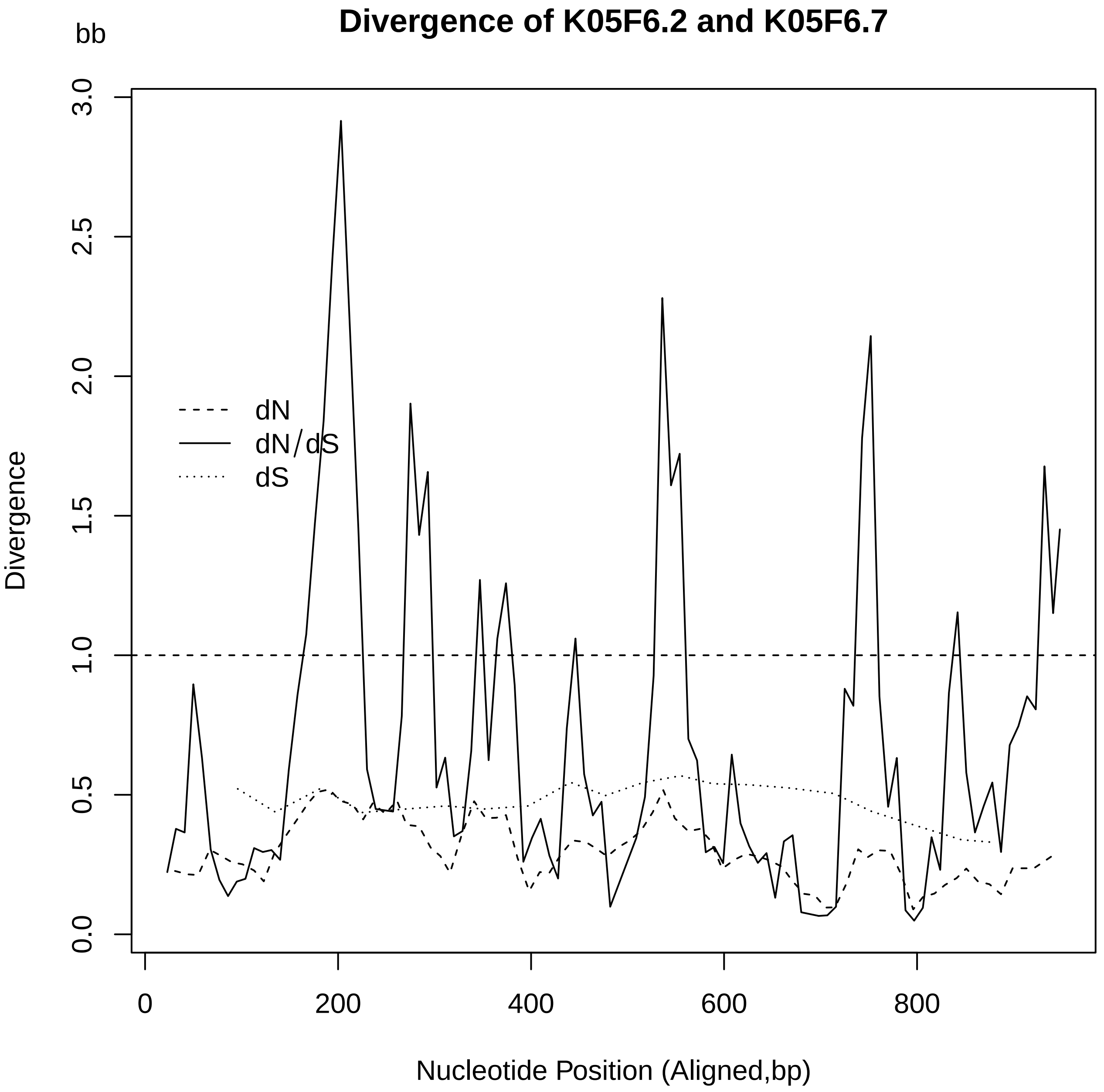
Divergence of K05F6.2 and K05F6.7
bb
Divergence
Nucleotide Position (Aligned,bp)
dN
dN/dS
dS
0.0
0.5
1.0
1.5
2.0
2.5
3.0
0
200
400
600
800

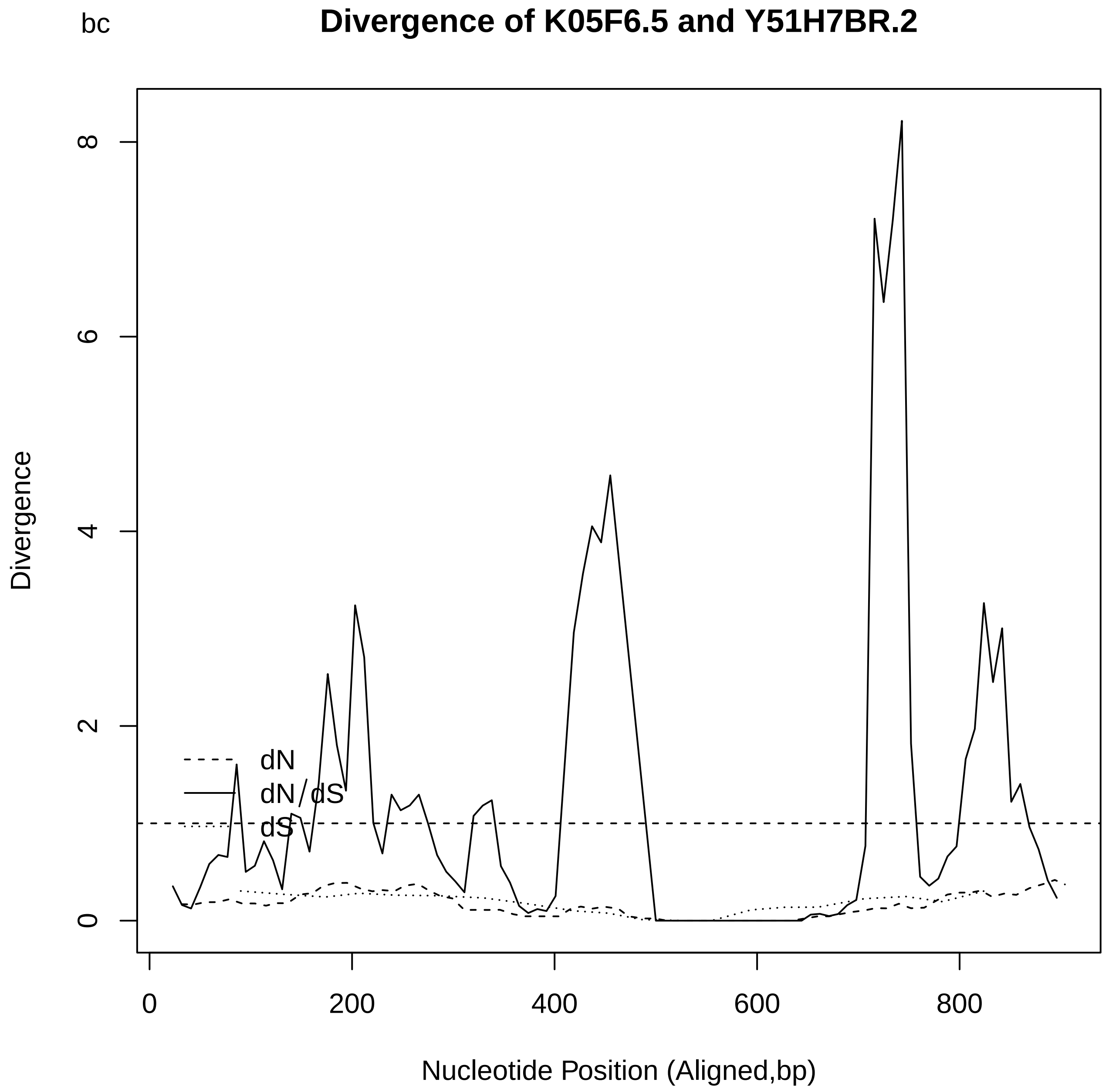
bc
Divergence of K05F6.5 and Y51H7BR.2
Divergence
Nucleotide Position (Aligned,bp)
0
2
4
6
8
0
200
400
600
800
dN
dN/dS
dS

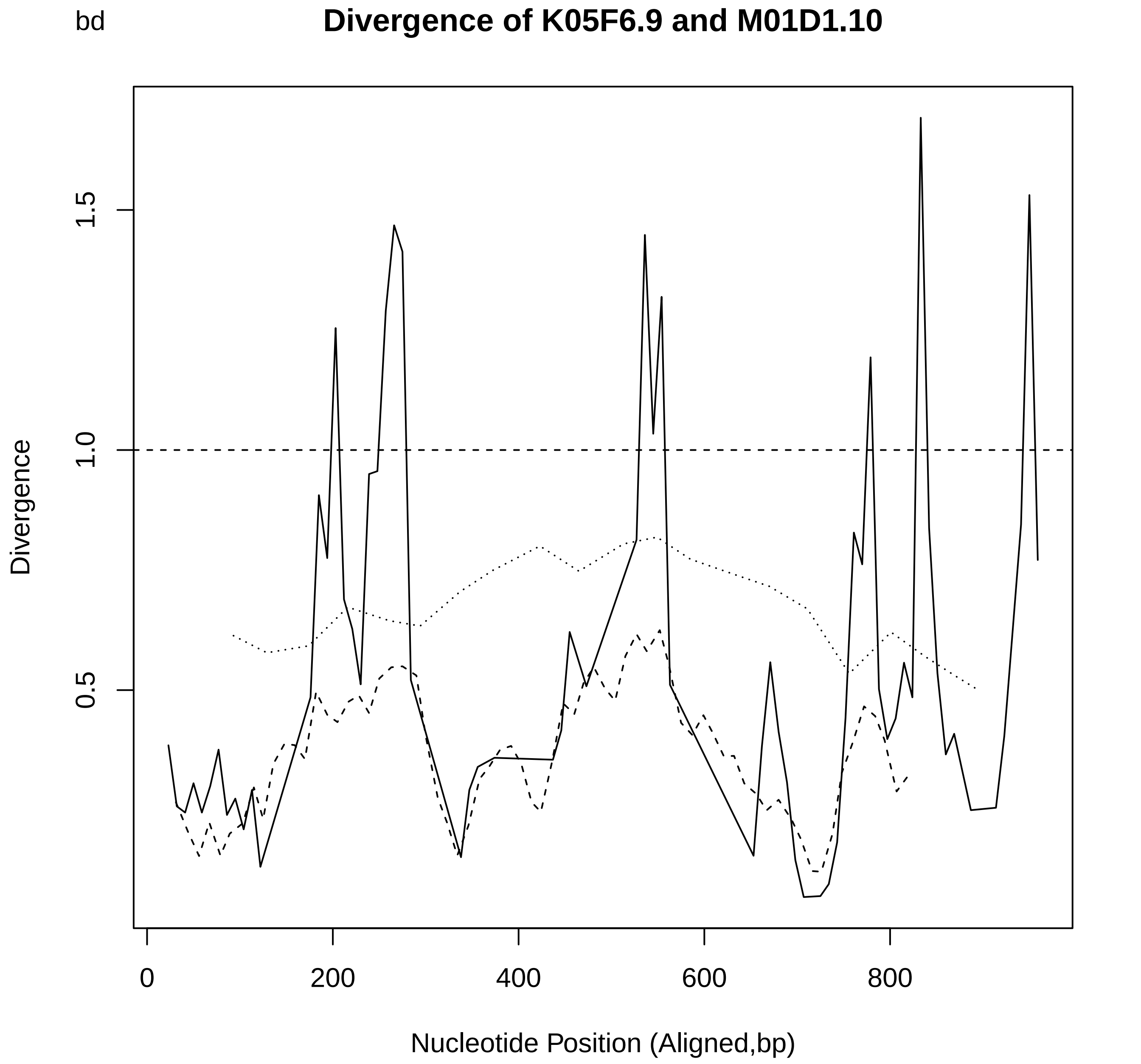
bd
Divergence of K05F6.9 and M01D1.10
Divergence
0.5
1.0
1.5
0
200
400
600
800
Nucleotide Position (Aligned,bp)

be

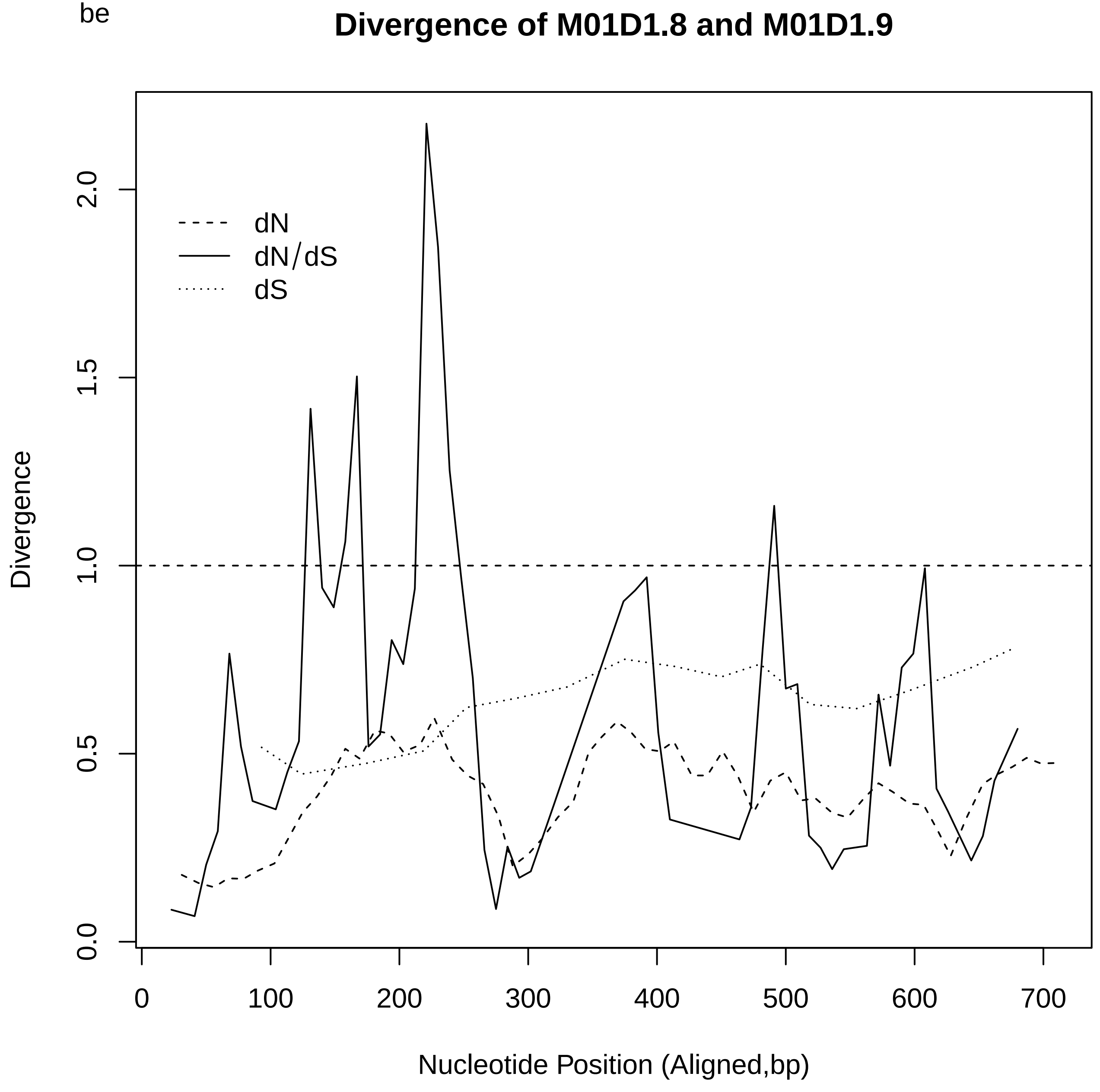
Divergence of M01D1.8 and M01D1.9
dN
dN/dS
dS
Divergence
0.0
0.5
1.0
1.5
2.0
0
100
200
300
400
500
600
700
Nucleotide Position (Aligned,bp)

bf

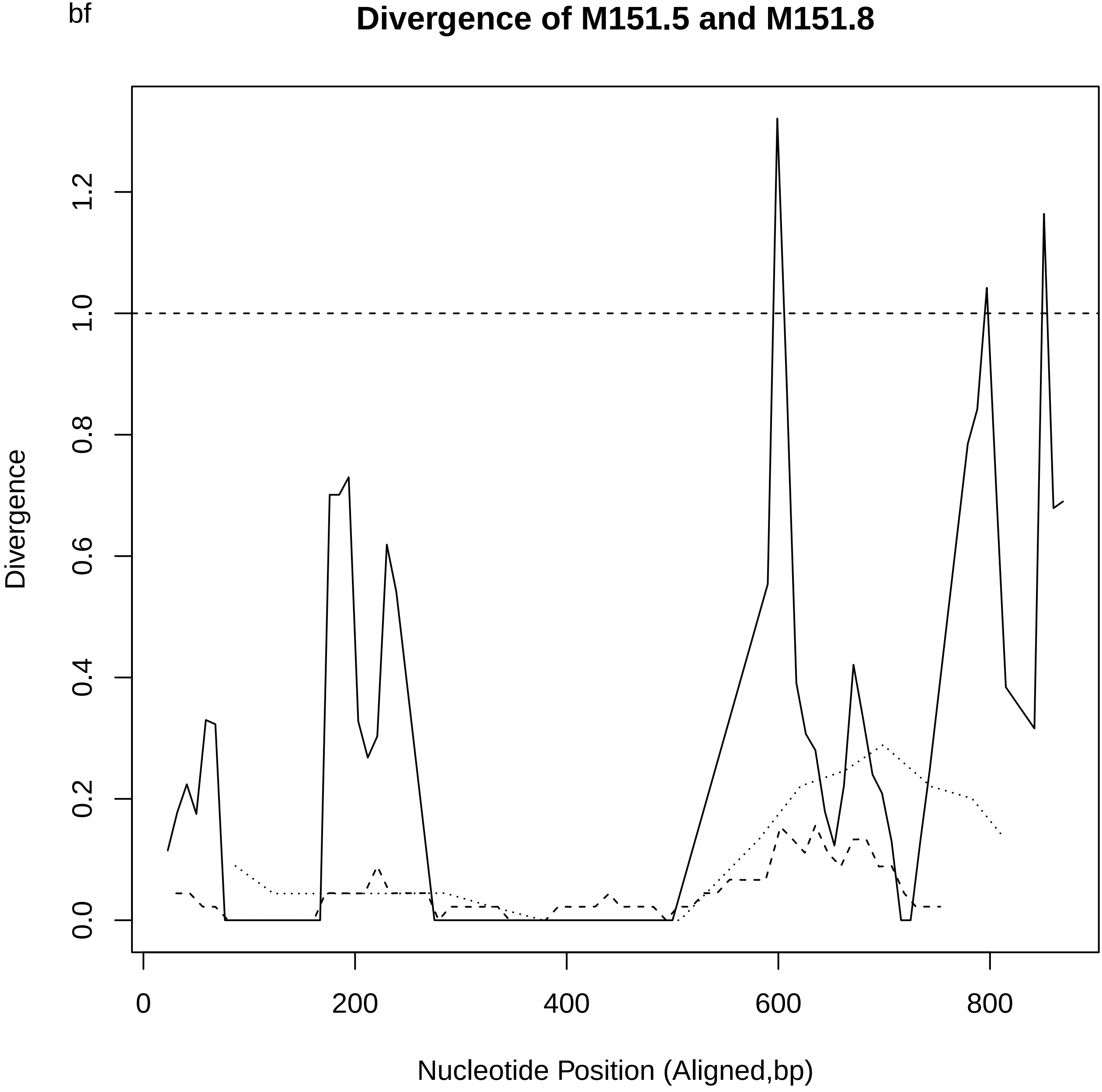

Divergence of M151.5 and M151.8
Divergence
0.0
0.2
0.4
0.6
0.8
1.0
1.2
0
200
400
600
800
Nucleotide Position (Aligned,bp)

bg

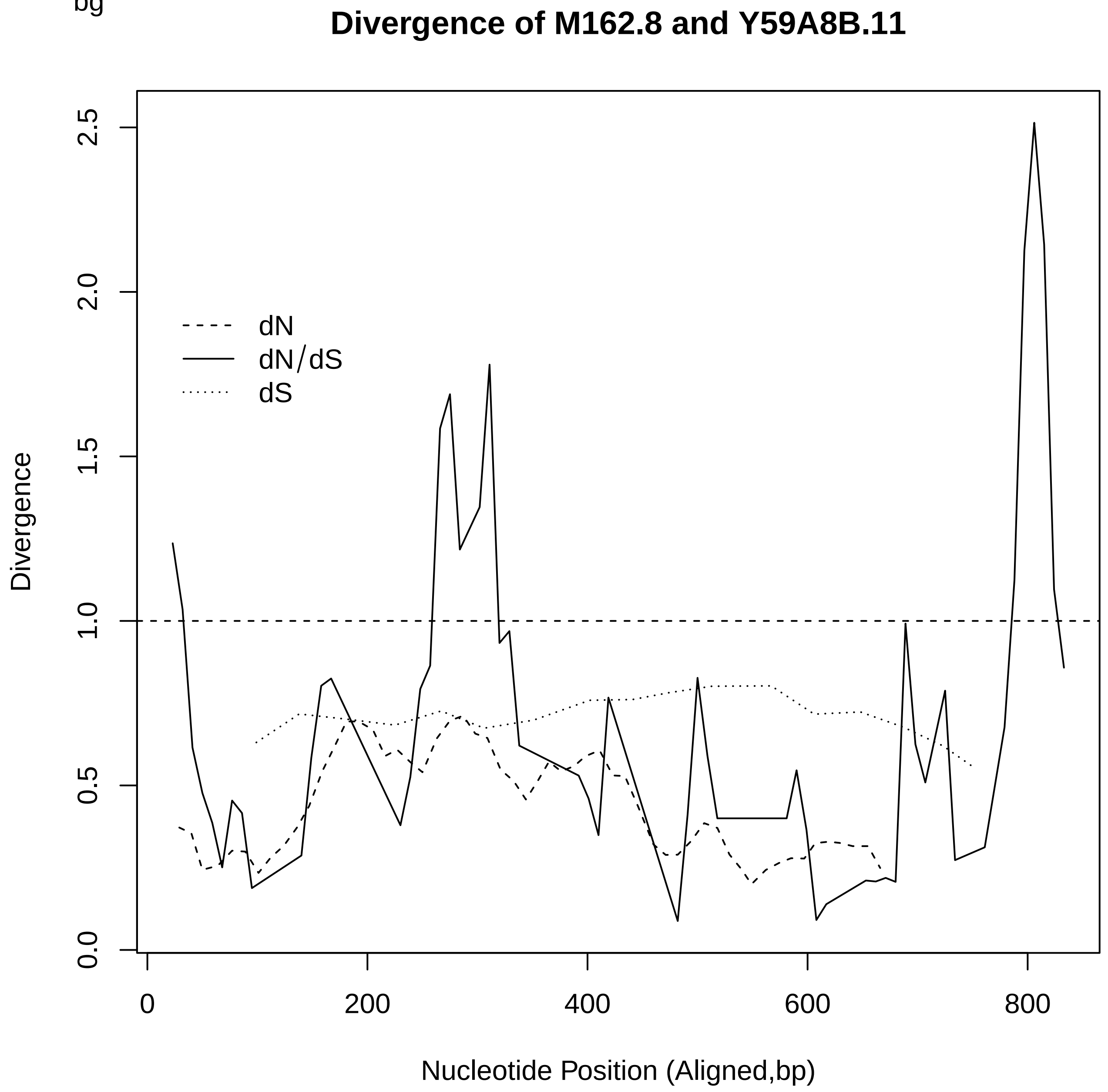
Divergence of M162.8 and Y59A8B.11
dN
dN/dS
dS
Divergence
0.0
0.5
1.0
1.5
2.0
2.5
0
200
400
600
800
Nucleotide Position (Aligned,bp)

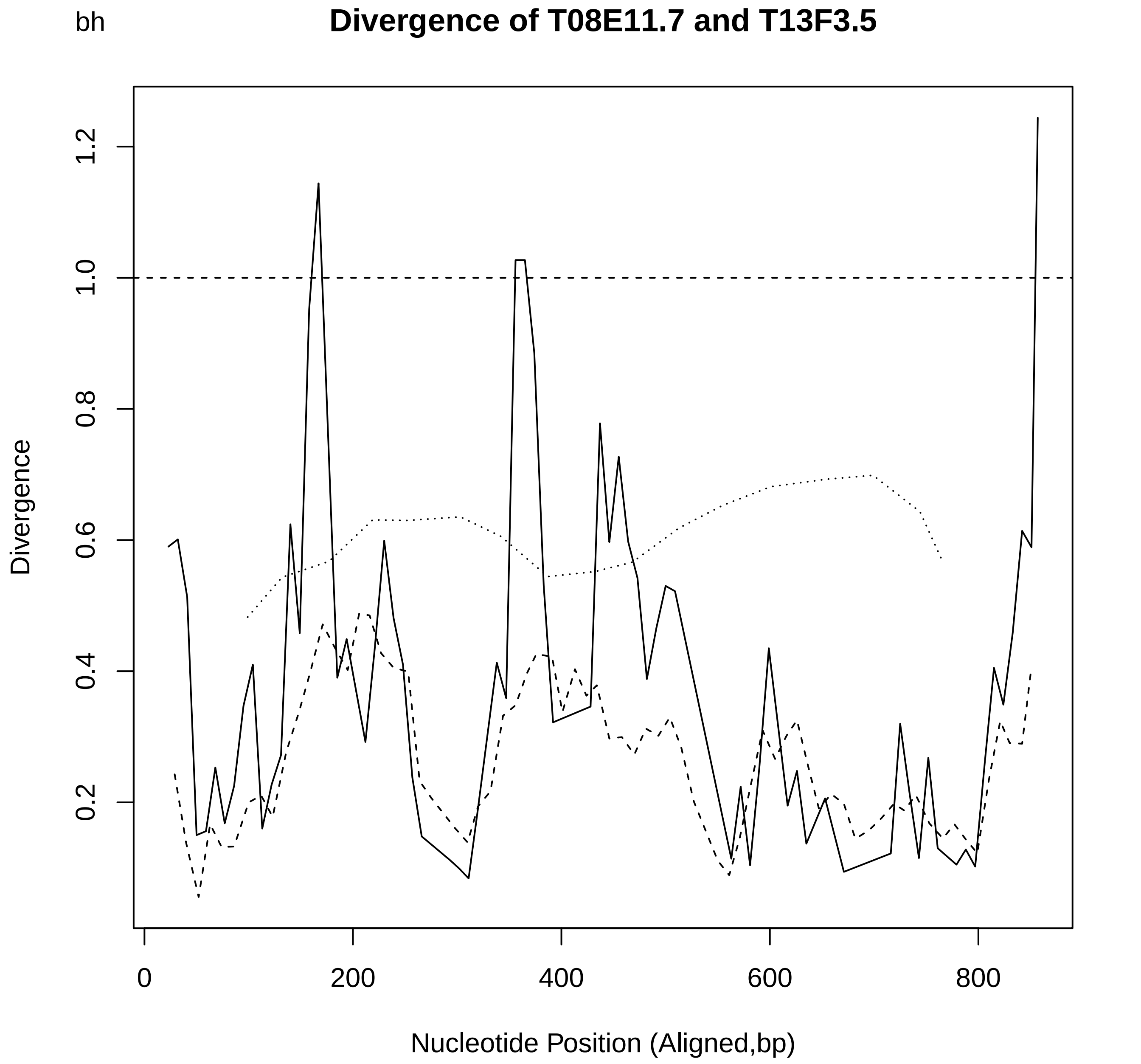

bh
Divergence of T08E11.7 and T13F3.5
Divergence
Nucleotide Position (Aligned,bp)
0
200
400
600
800
0.2
0.4
0.6
0.8
1.0
1.2

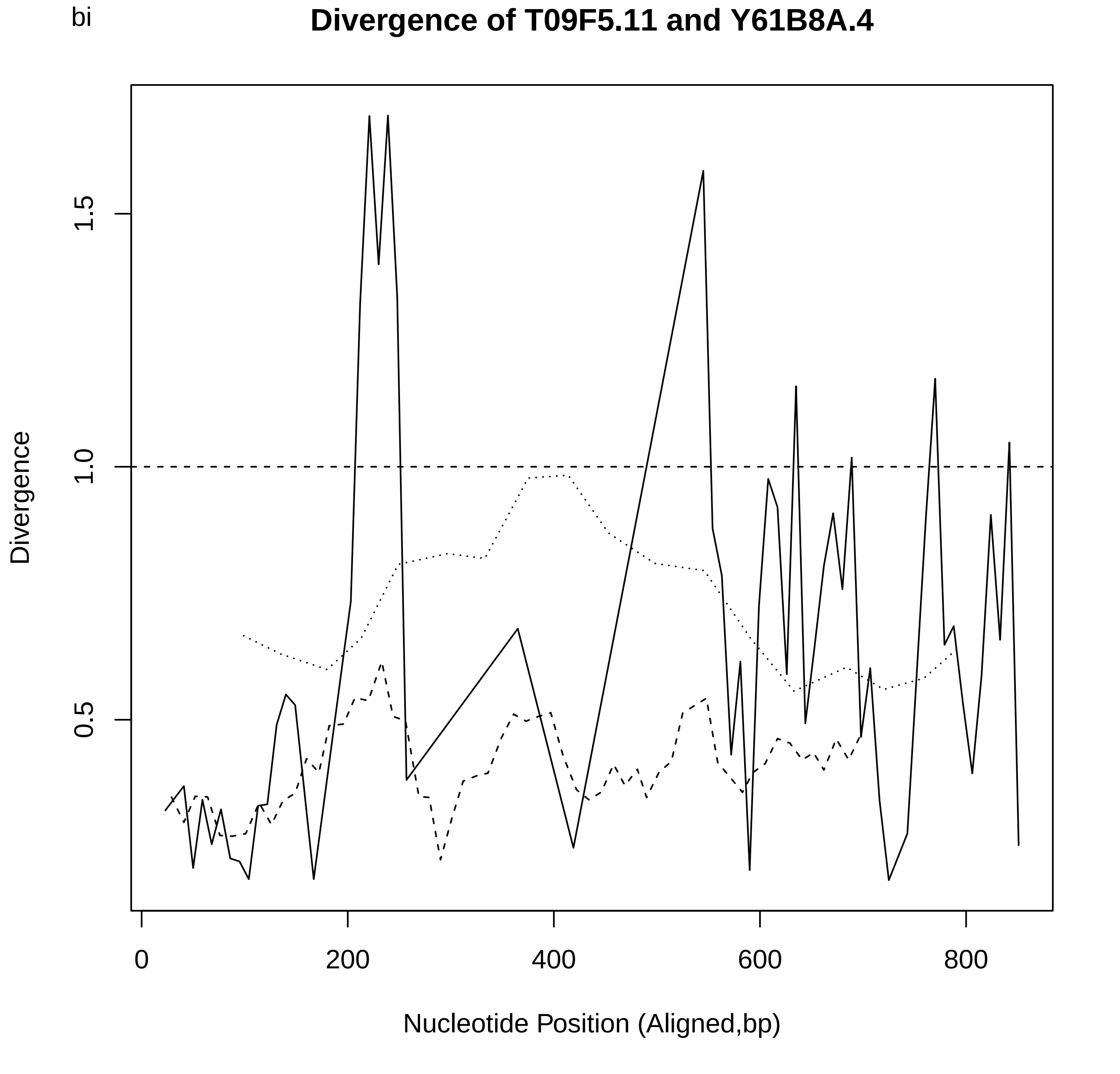
bi
Divergence of T09F5.11 and Y61B8A.4
Divergence
1.5
1.0
0.5
0
200
400
600
800
Nucleotide Position (Aligned,bp)

bj

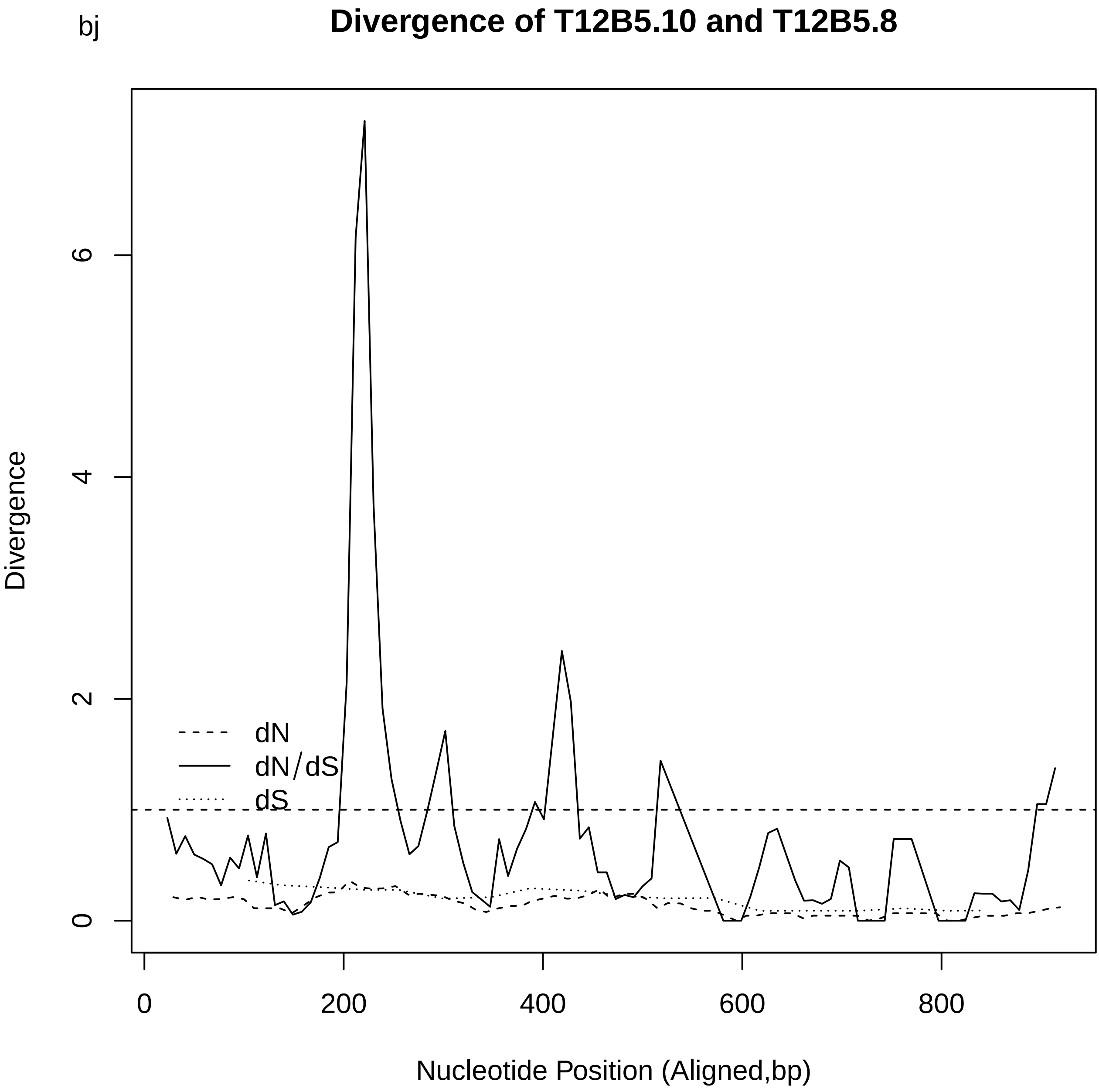

Divergence of T12B5.10 and T12B5.8
Divergence
Nucleotide Position (Aligned,bp)
dN
dN/dS
dS
0
2
4
6
0
200
400
600
800

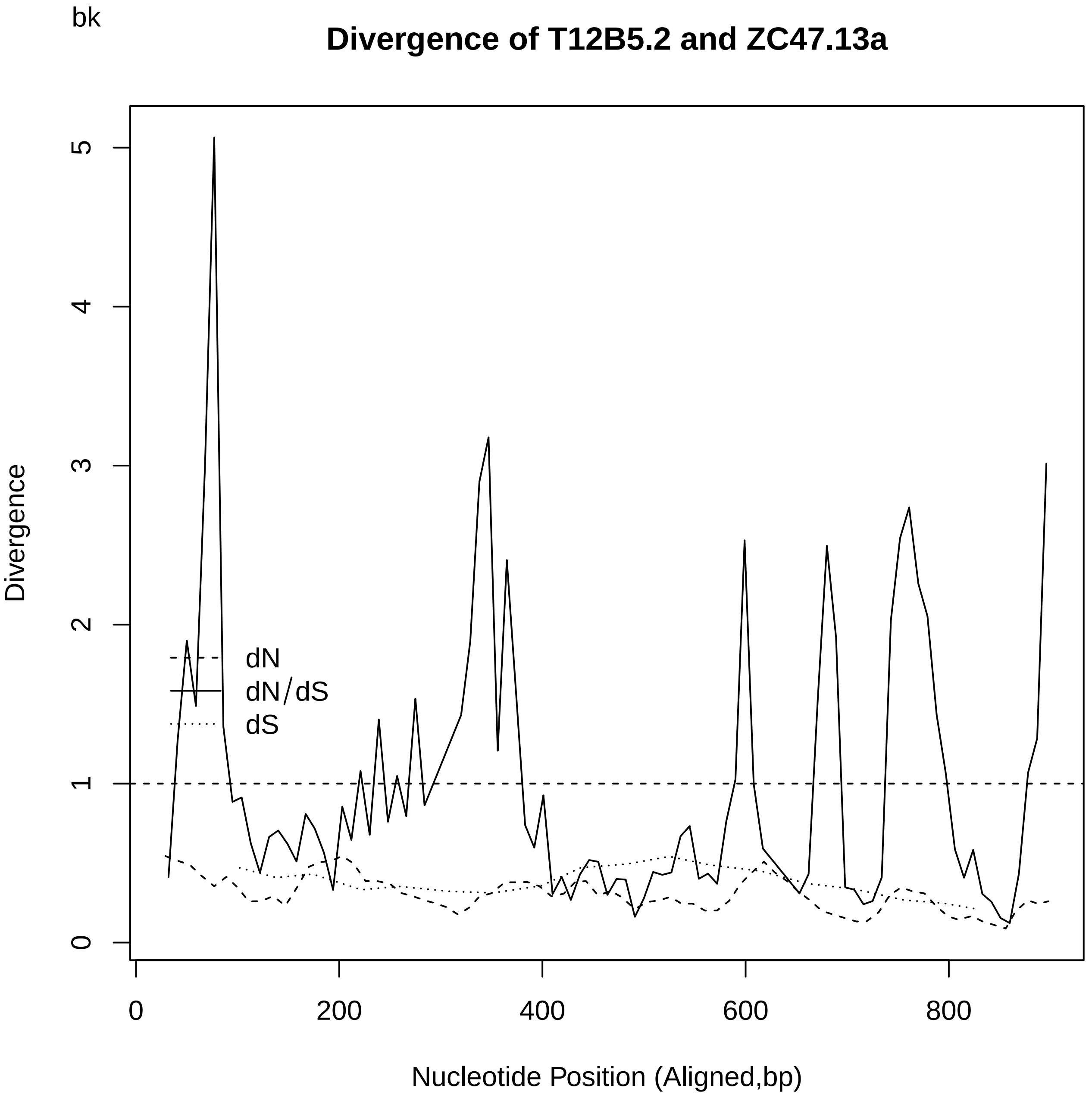
bk
Divergence of T12B5.2 and ZC47.13a
Divergence
Nucleotide Position (Aligned,bp)
dN
dN/dS
dS
0
1
2
3
4
5
0
200
400
600
800

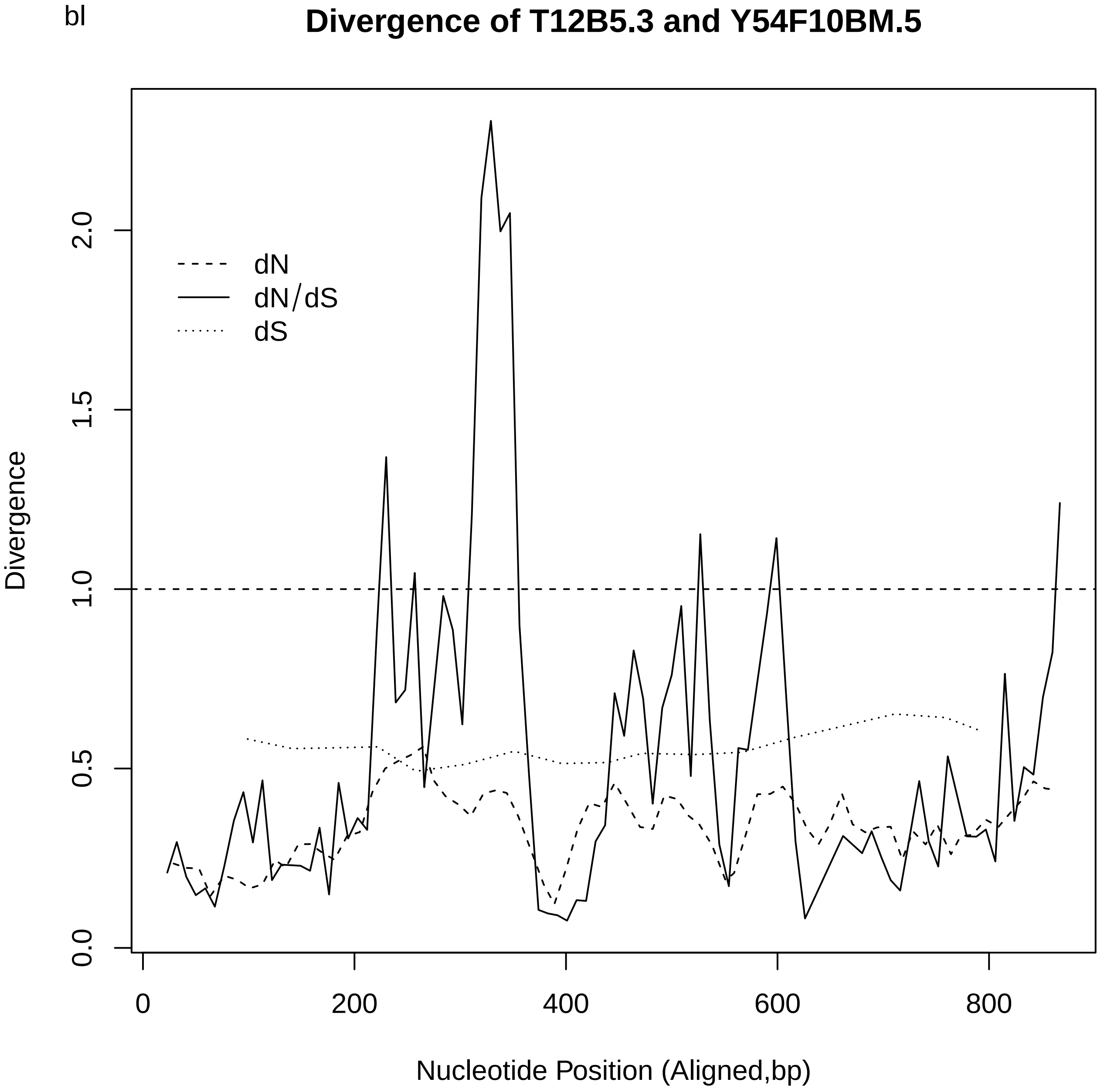
bl
Divergence of T12B5.3 and Y54F10BM.5
dN
dN/dS
dS
Divergence
0.0
0.5
1.0
1.5
2.0
0
200
400
600
800
Nucleotide Position (Aligned,bp)

bm

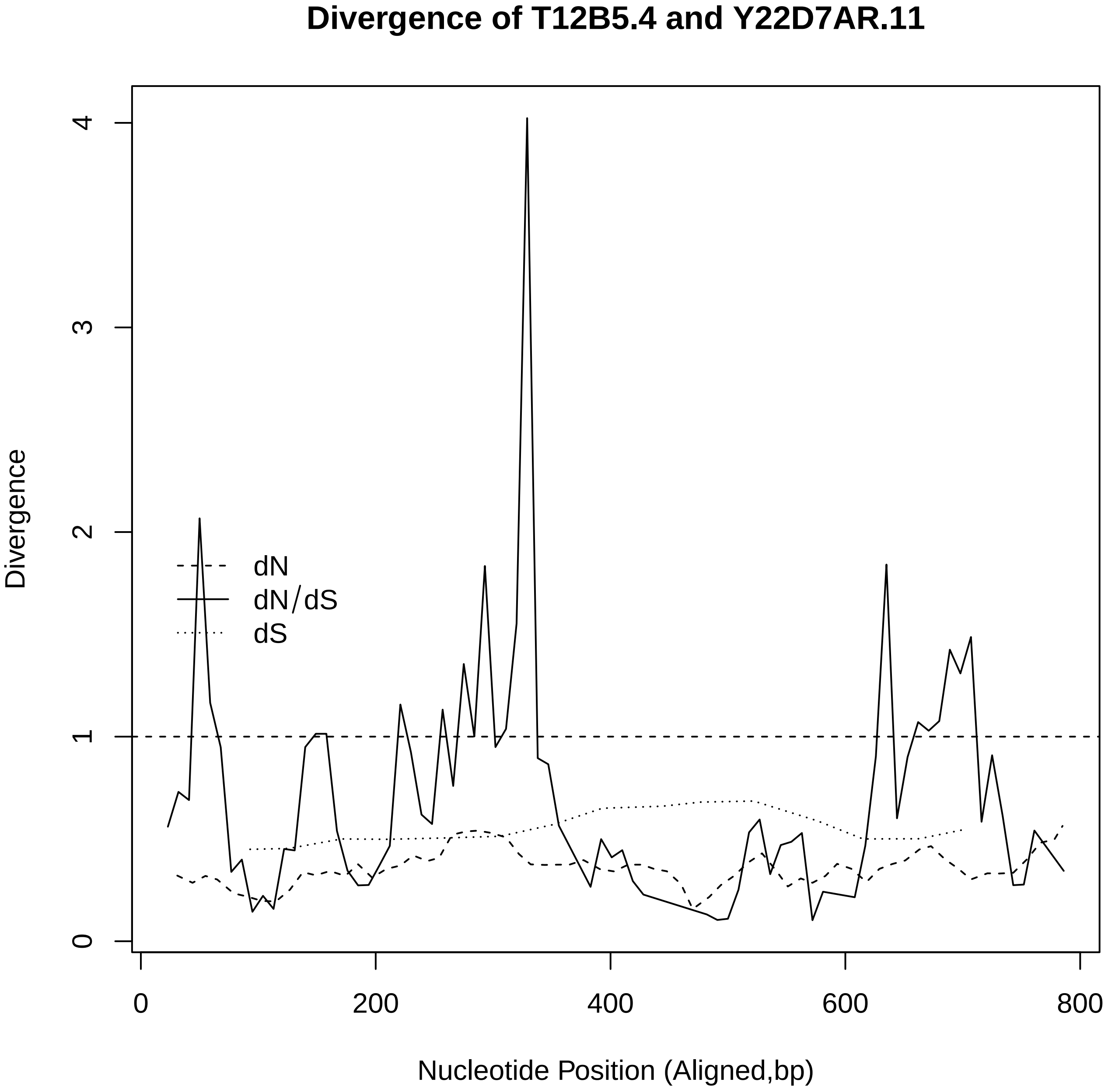

Divergence of T12B5.4 and Y22D7AR.11
Divergence
0
1
2
3
4
dN
dN/dS
dS
0
200
400
600
800
Nucleotide Position (Aligned,bp)

bn

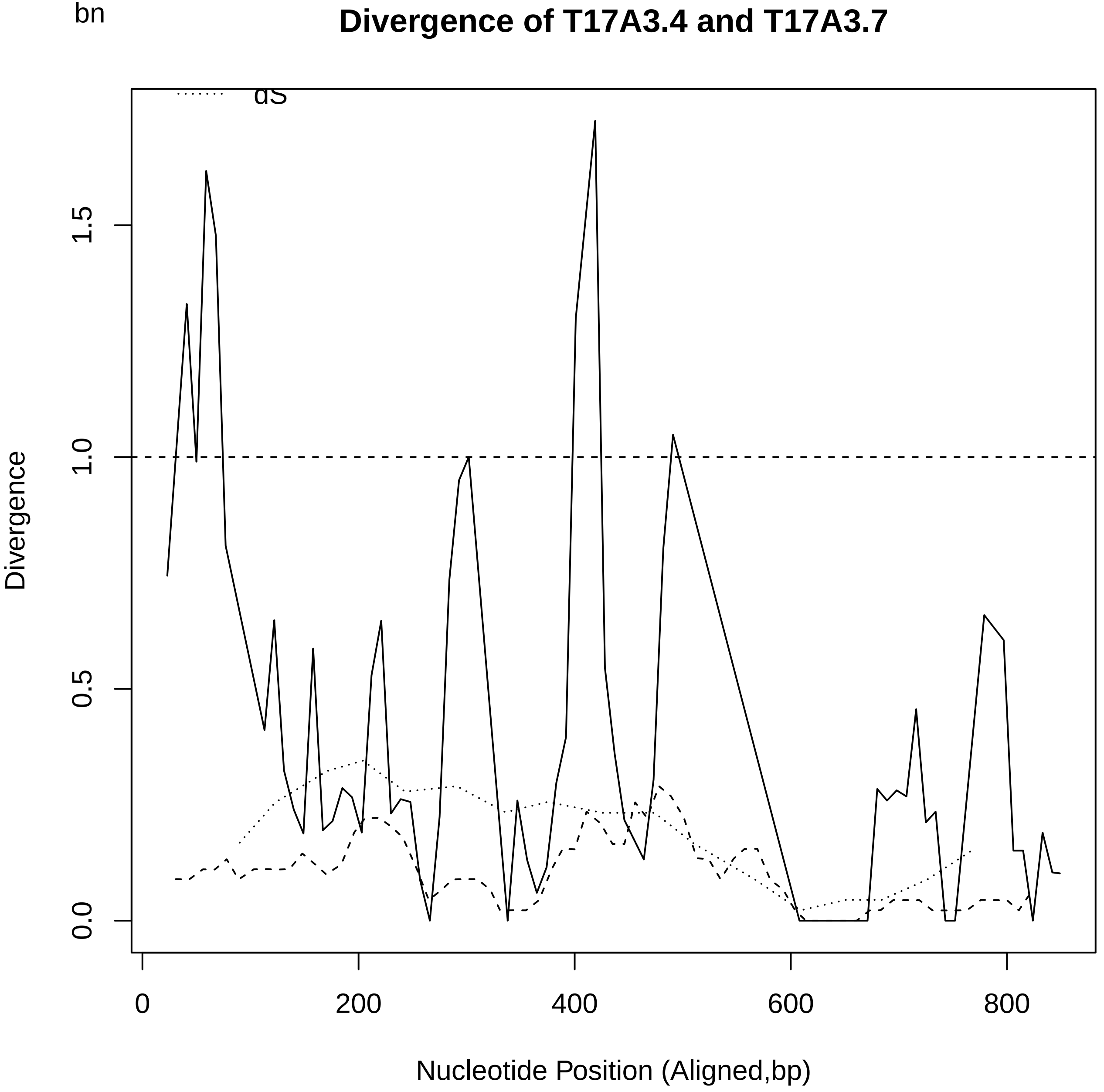
Divergence of T17A3.4 and T17A3.7
dS
Divergence
0.0
0.5
1.0
1.5
0
200
400
600
800
Nucleotide Position (Aligned,bp)

bo

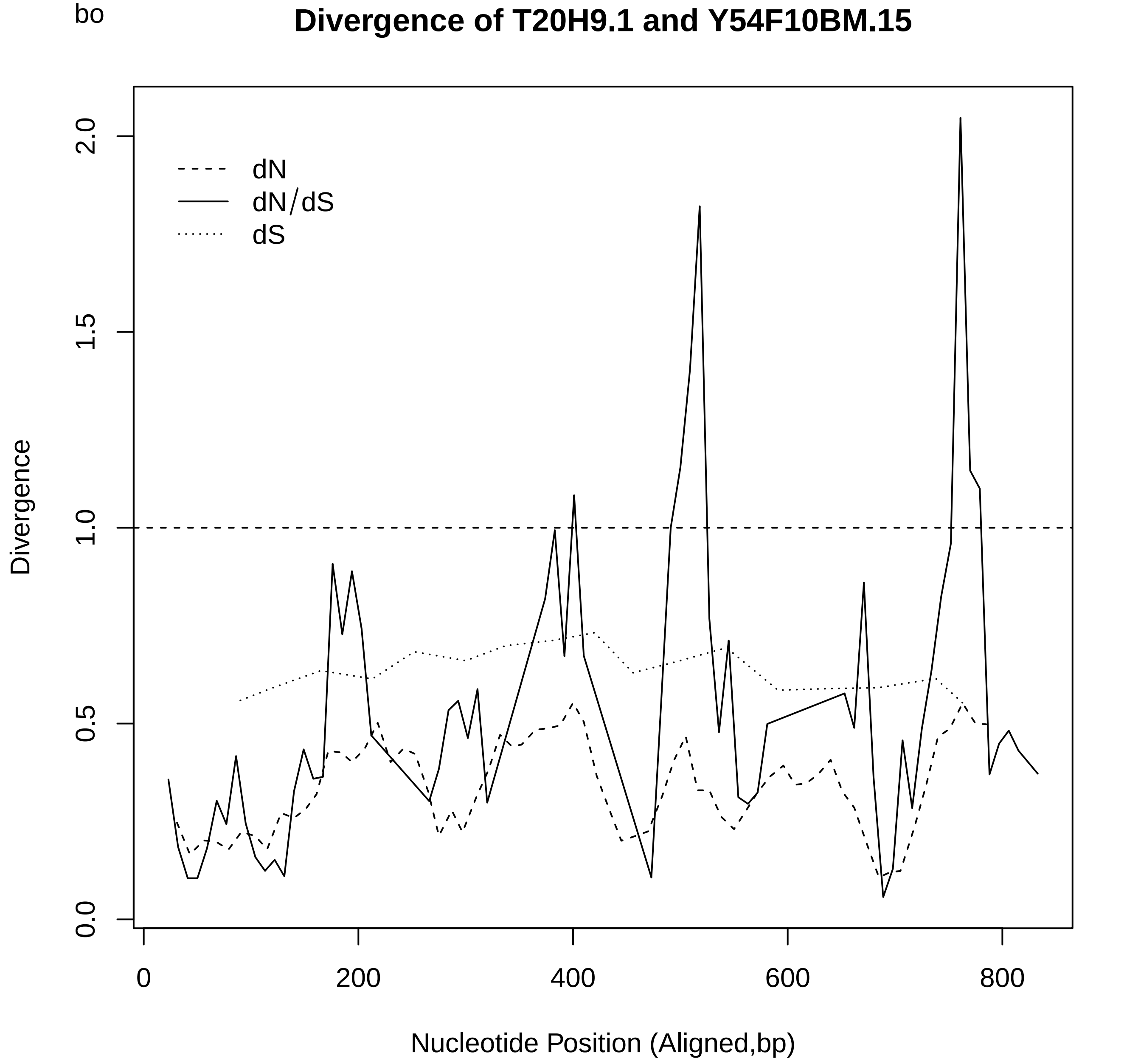

Divergence of T20H9.1 and Y54F10BM.15
dN
dN/dS
dS
Divergence
0.0
0.5
1.0
1.5
2.0
0
200
400
600
800
Nucleotide Position (Aligned,bp)

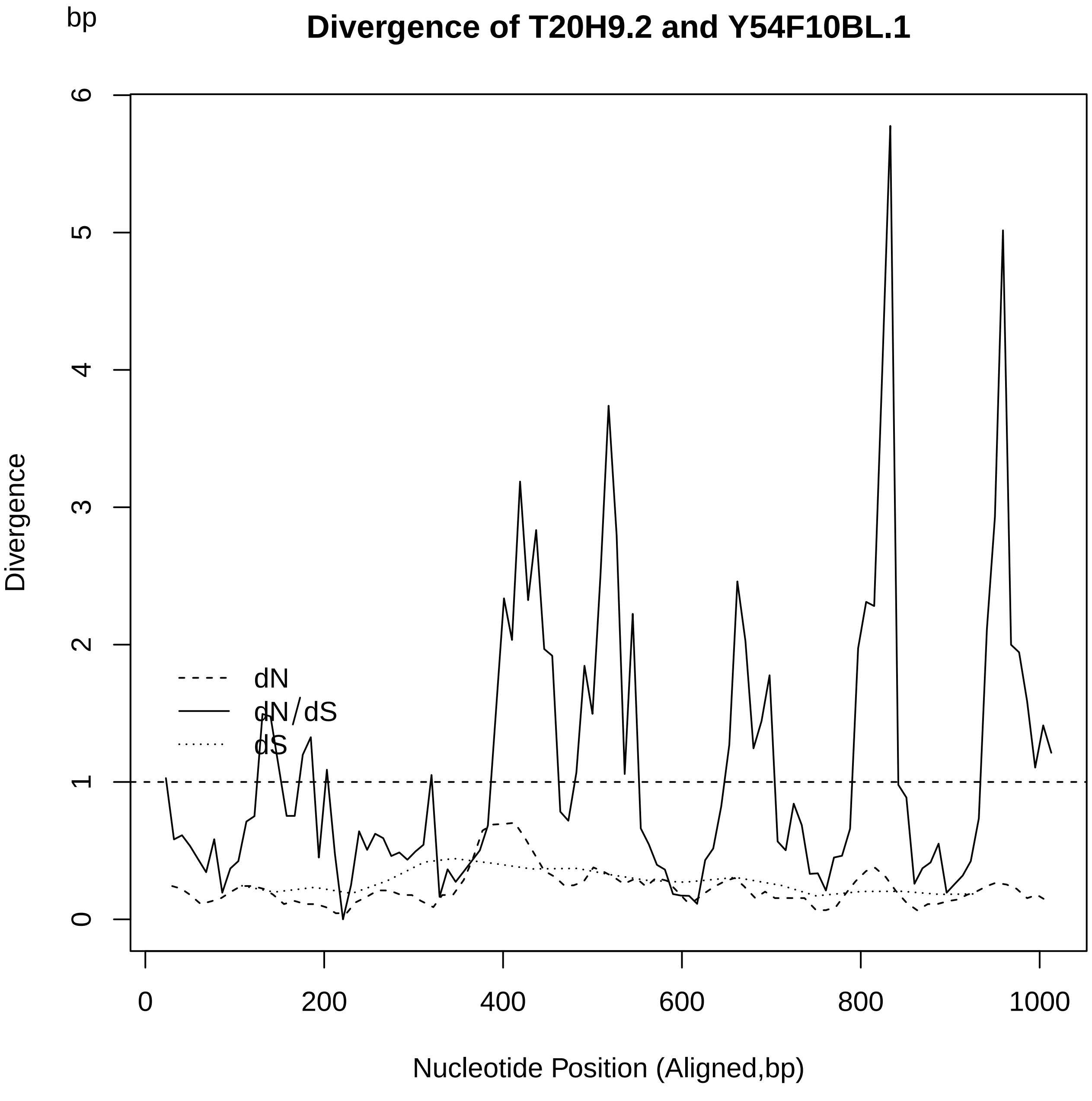
bp
Divergence of T20H9.2 and Y54F10BL.1
Divergence
0
1
2
3
4
5
6
dN
dN/dS
dS
0
200
400
600
800
1000
Nucleotide Position (Aligned,bp)

bq

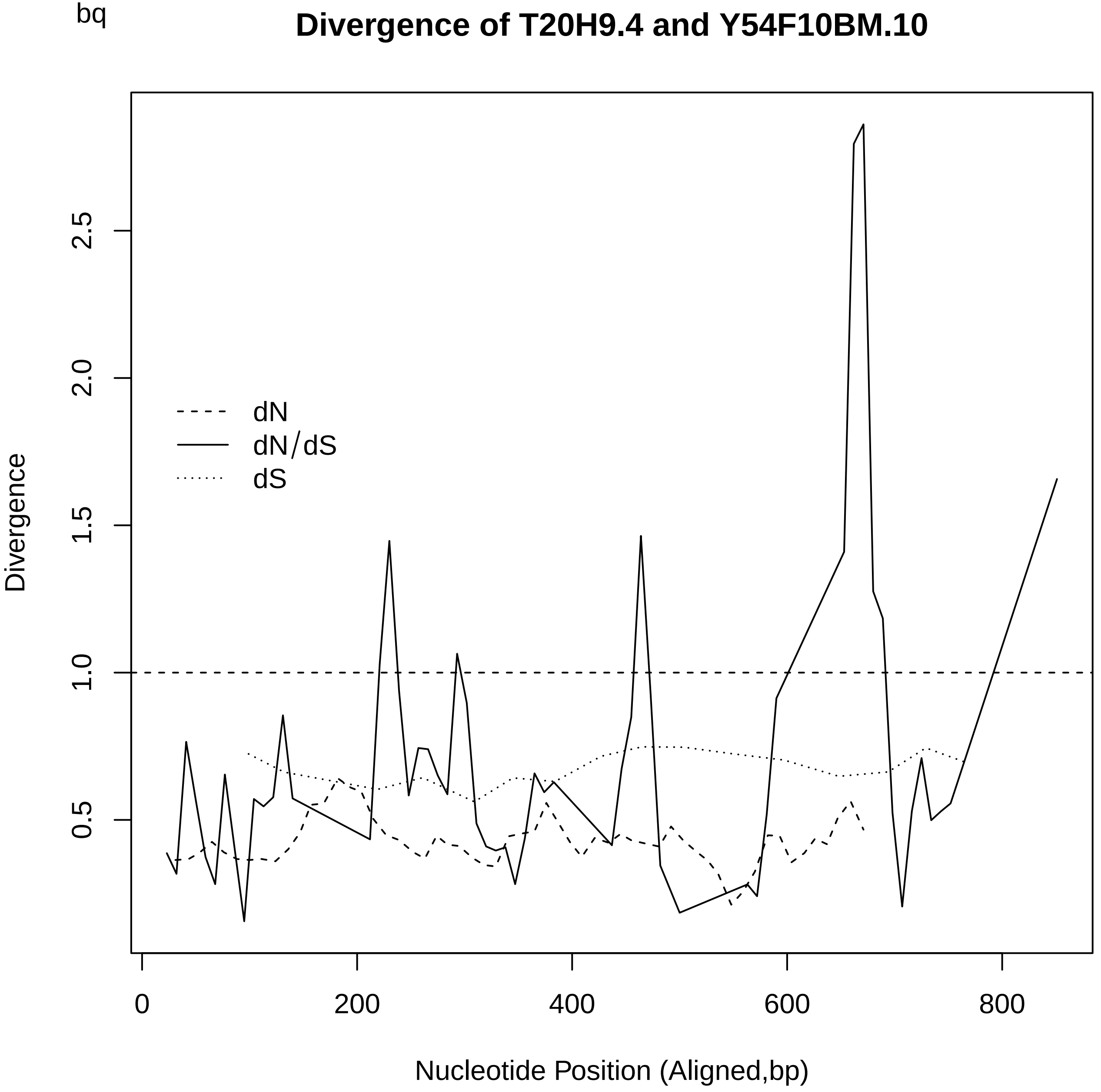
Divergence of T20H9.4 and Y54F10BM.10
dN
dN/dS
dS
Divergence
0.5
1.0
1.5
2.0
2.5
0
200
400
600
800
Nucleotide Position (Aligned,bp)

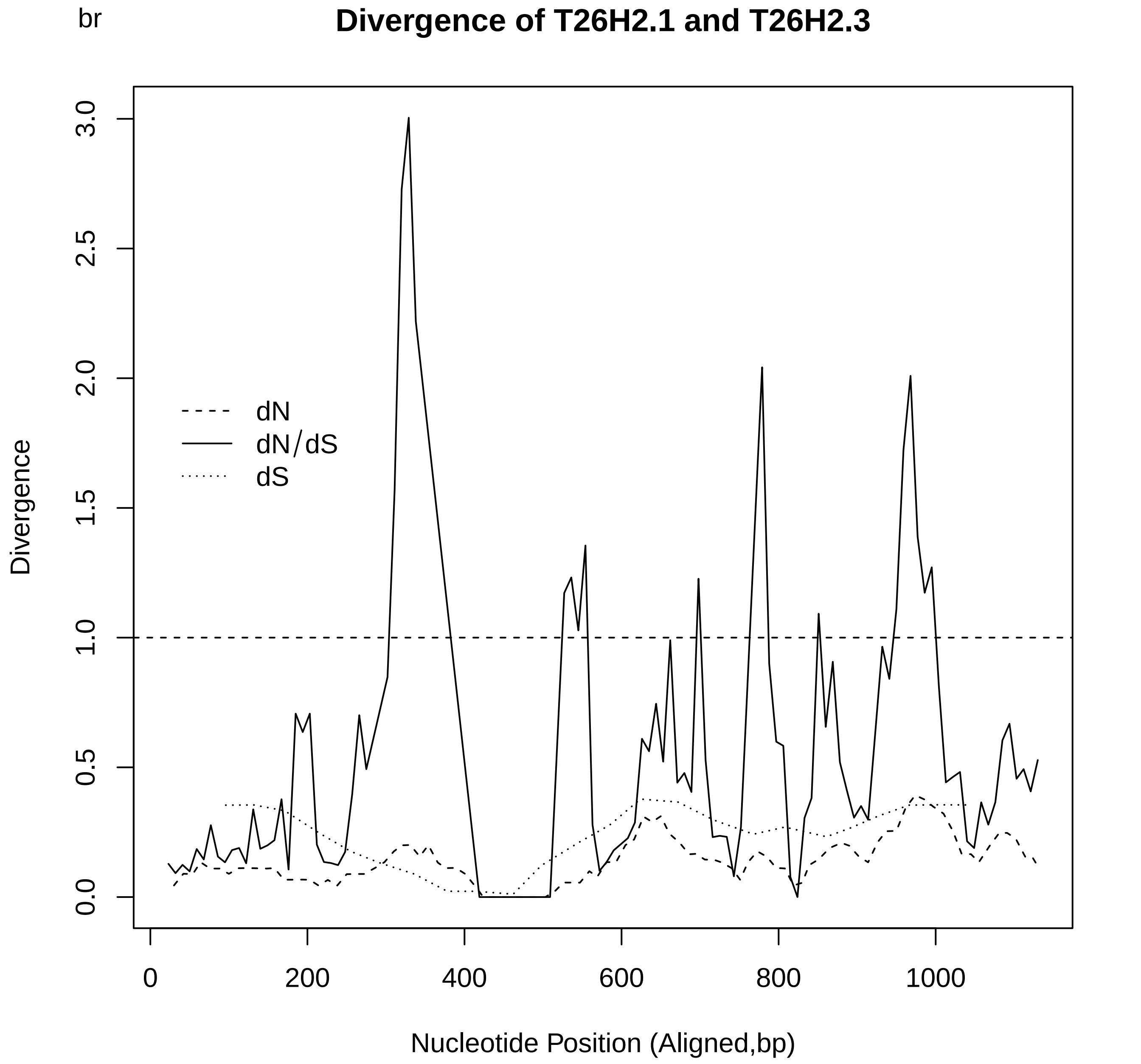
br
Divergence of T26H2.1 and T26H2.3
dN
dN/dS
dS
Divergence
0.0
0.5
1.0
1.5
2.0
2.5
3.0
0
200
400
600
800
1000
Nucleotide Position (Aligned,bp)

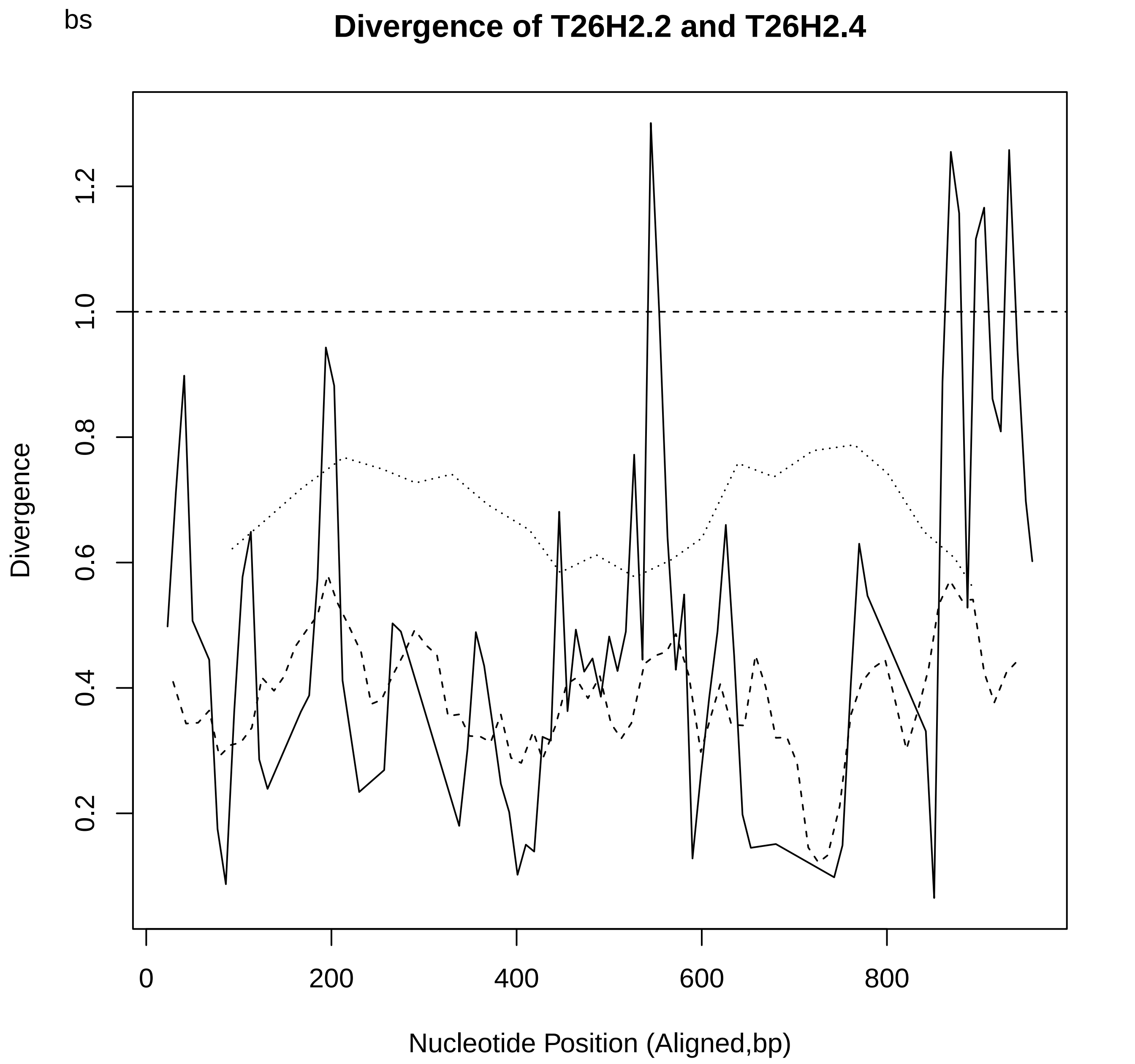

bs
Divergence of T26H2.2 and T26H2.4
Divergence
0.2
0.4
0.6
0.8
1.0
1.2
0
200
400
600
800
Nucleotide Position (Aligned,bp)

bt

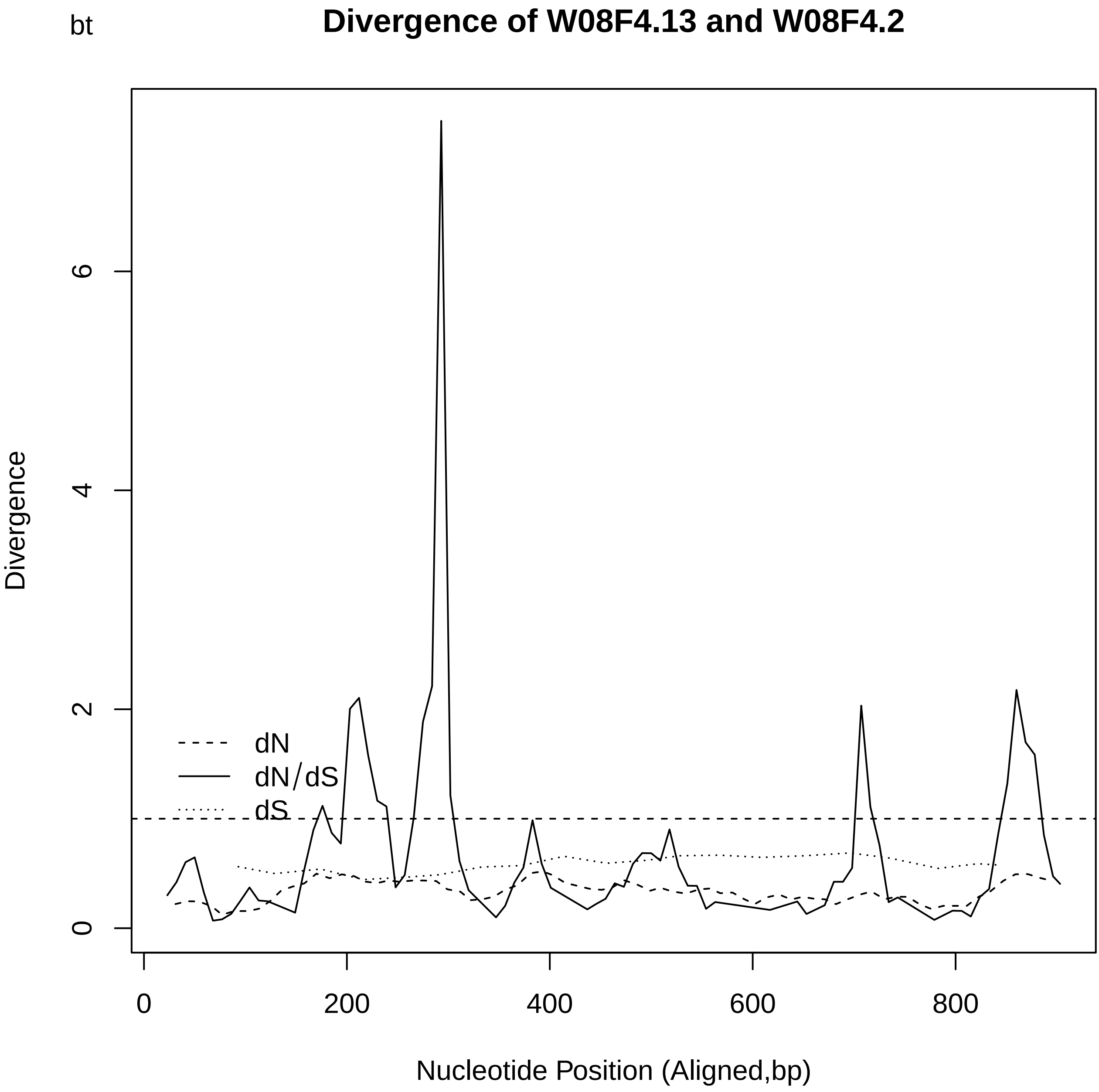
Divergence of W08F4.13 and W08F4.2
Divergence
0
2
4
6
dN
dN/dS
dS
0
200
400
600
800
Nucleotide Position (Aligned,bp)

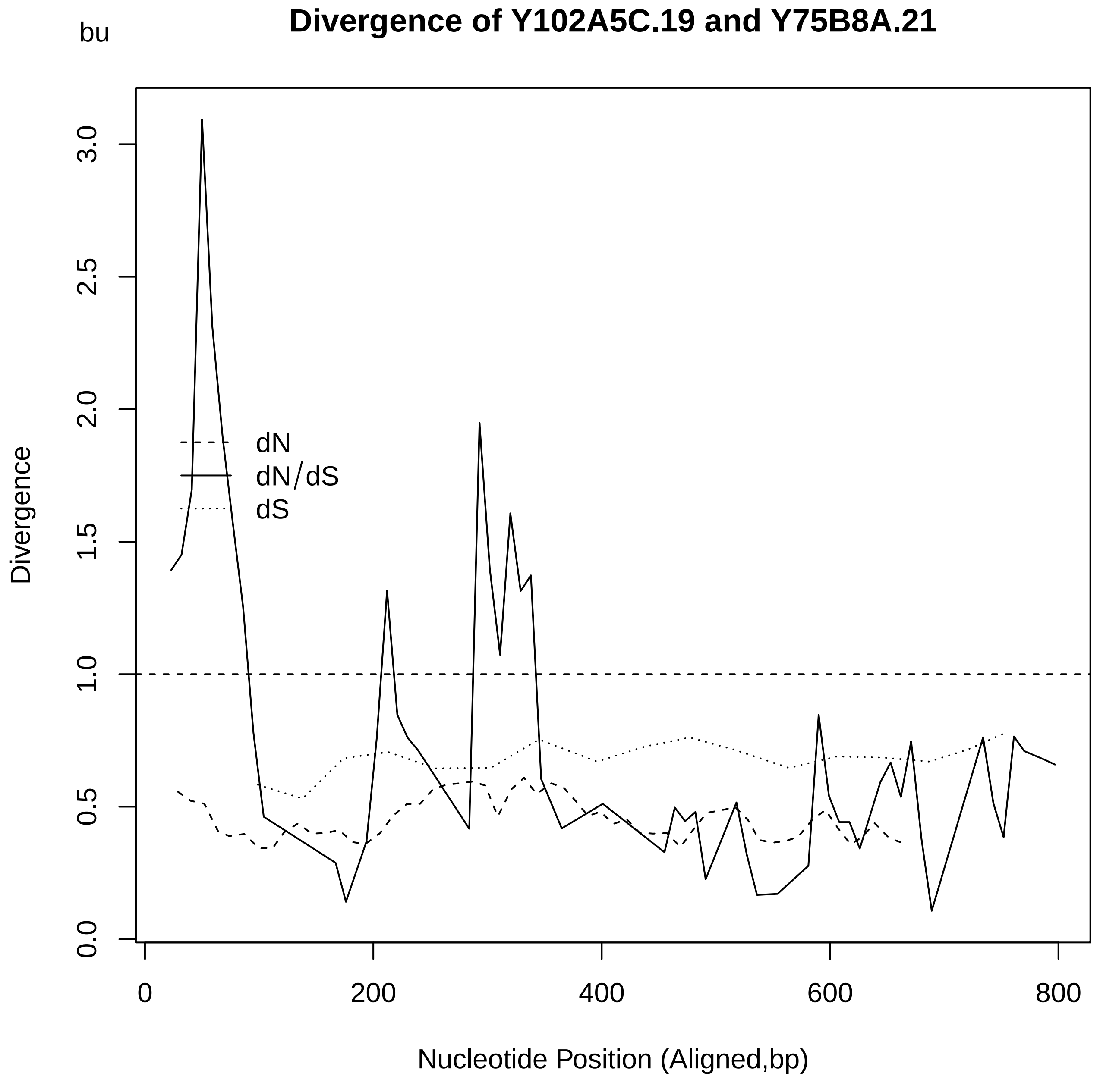
bu
Divergence of Y102A5C.19 and Y75B8A.21
dN
dN/dS
dS
Divergence
0.0
0.5
1.0
1.5
2.0
2.5
3.0
0
200
400
600
800
Nucleotide Position (Aligned,bp)

bv

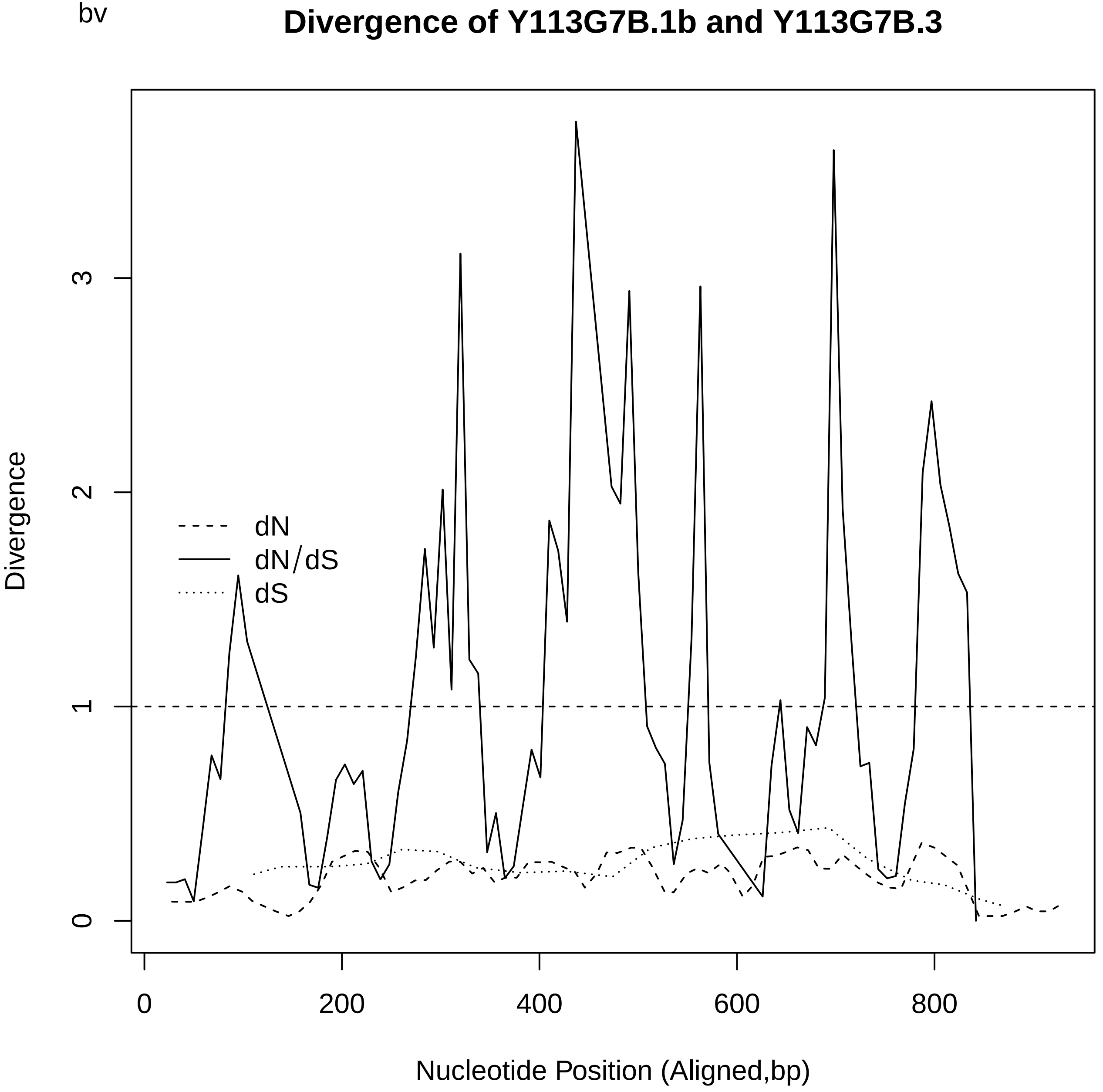

Divergence of Y113G7B.1b and Y113G7B.3
Divergence
Nucleotide Position (Aligned,bp)
dN
dN/dS
dS
0
1
2
3
0
200
400
600
800

bw

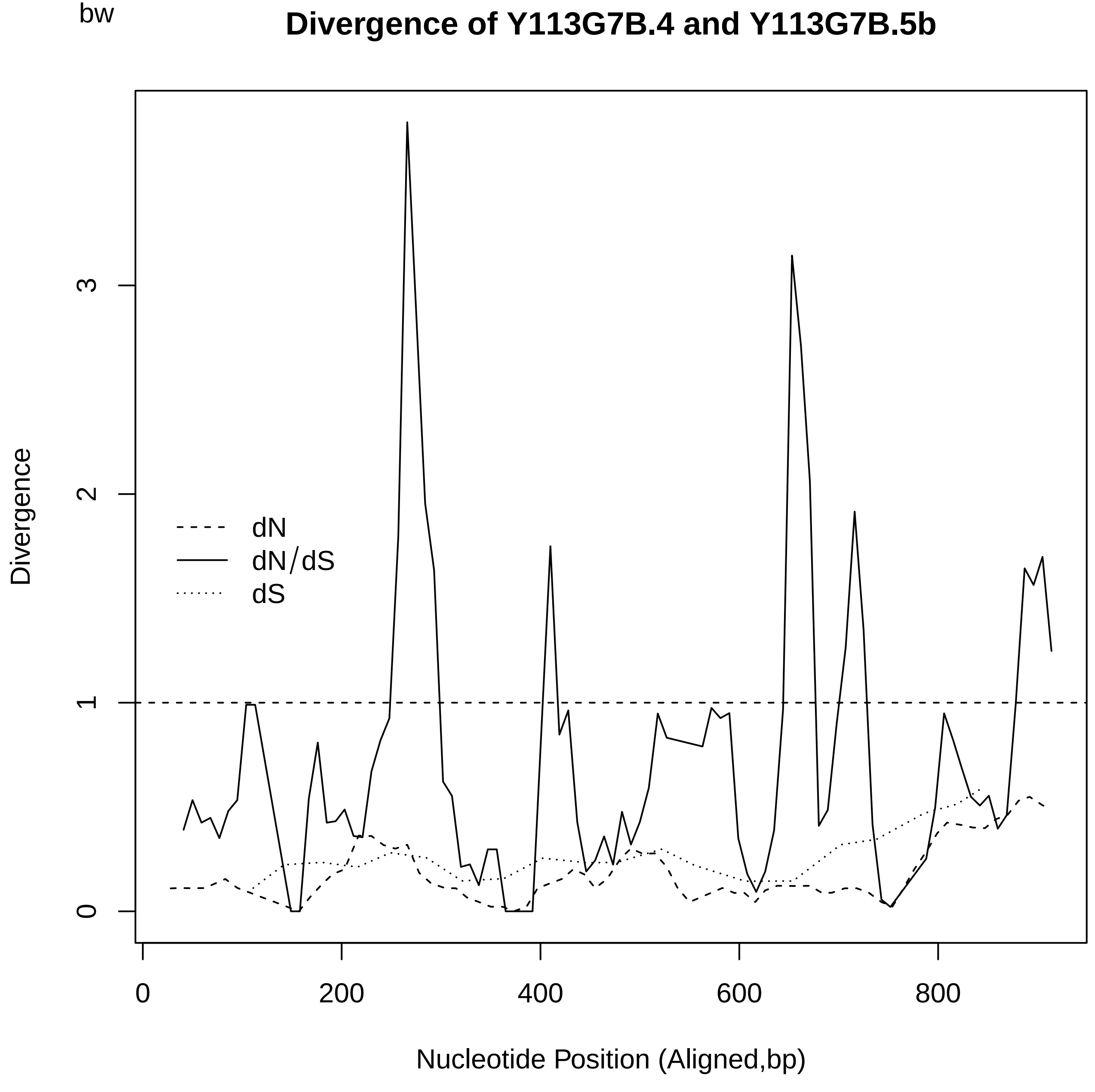

Divergence of Y113G7B.4 and Y113G7B.5b
Divergence
3
2
1
0
dN
dN/dS
dS
0
200
400
600
800
Nucleotide Position (Aligned,bp)

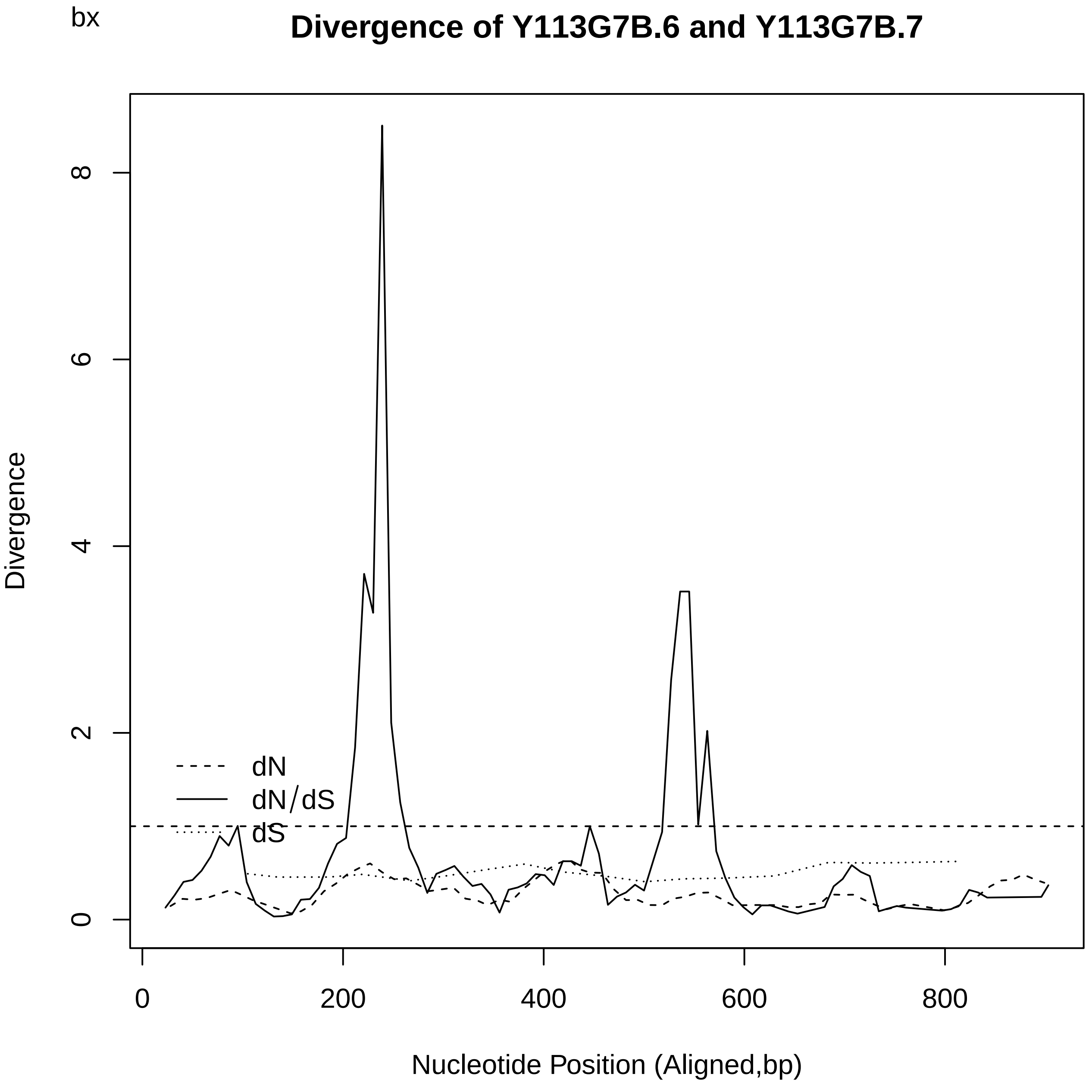
bx
Divergence of Y113G7B.6 and Y113G7B.7
Divergence
0
2
4
6
8
dN
dN/dS
dS
0
200
400
600
800
Nucleotide Position (Aligned,bp)

by

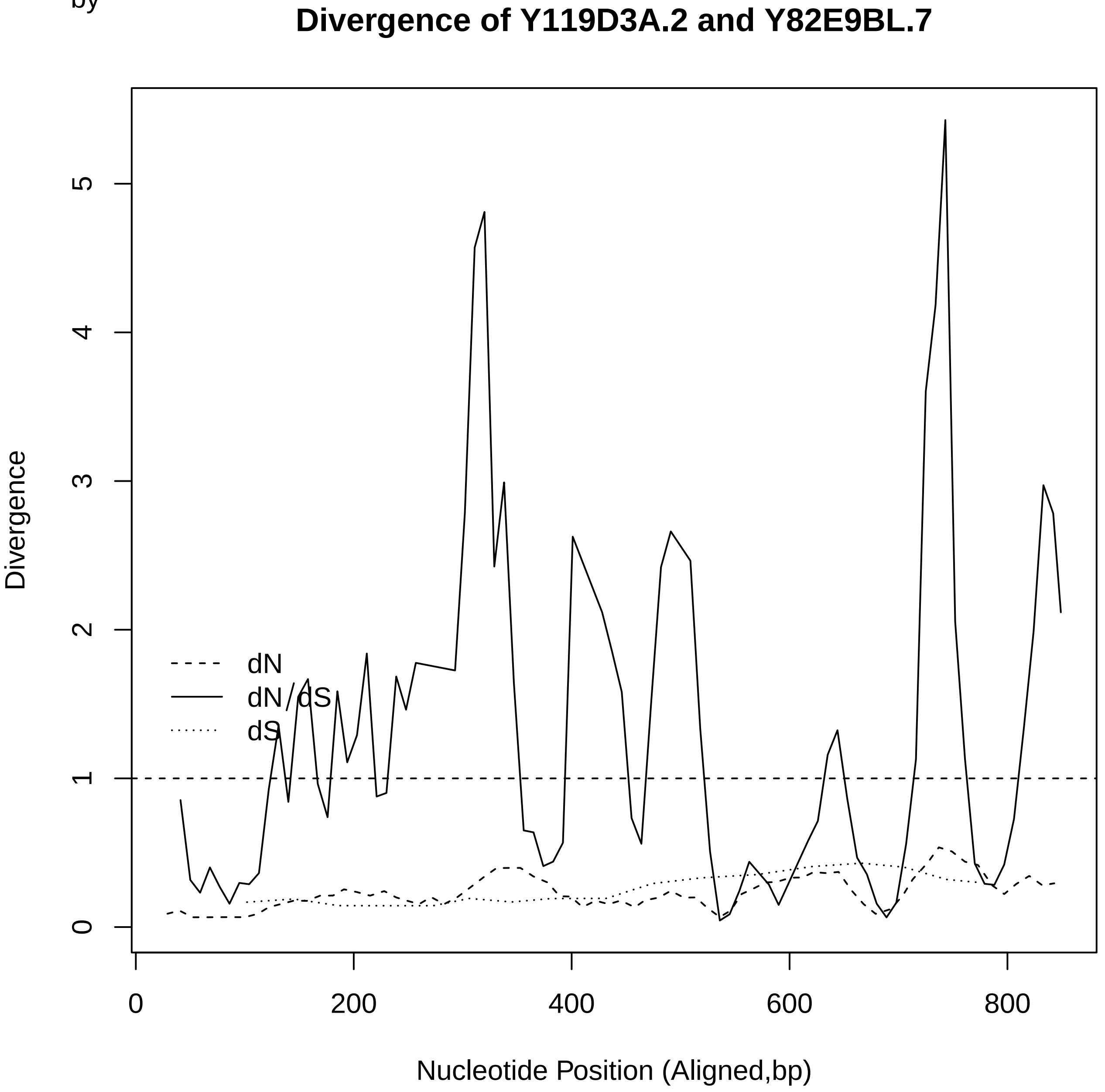

Divergence of Y119D3A.2 and Y82E9BL.7
Divergence
Nucleotide Position (Aligned,bp)
dN
dN/dS
dS
0
1
2
3
4
5
0
200
400
600
800

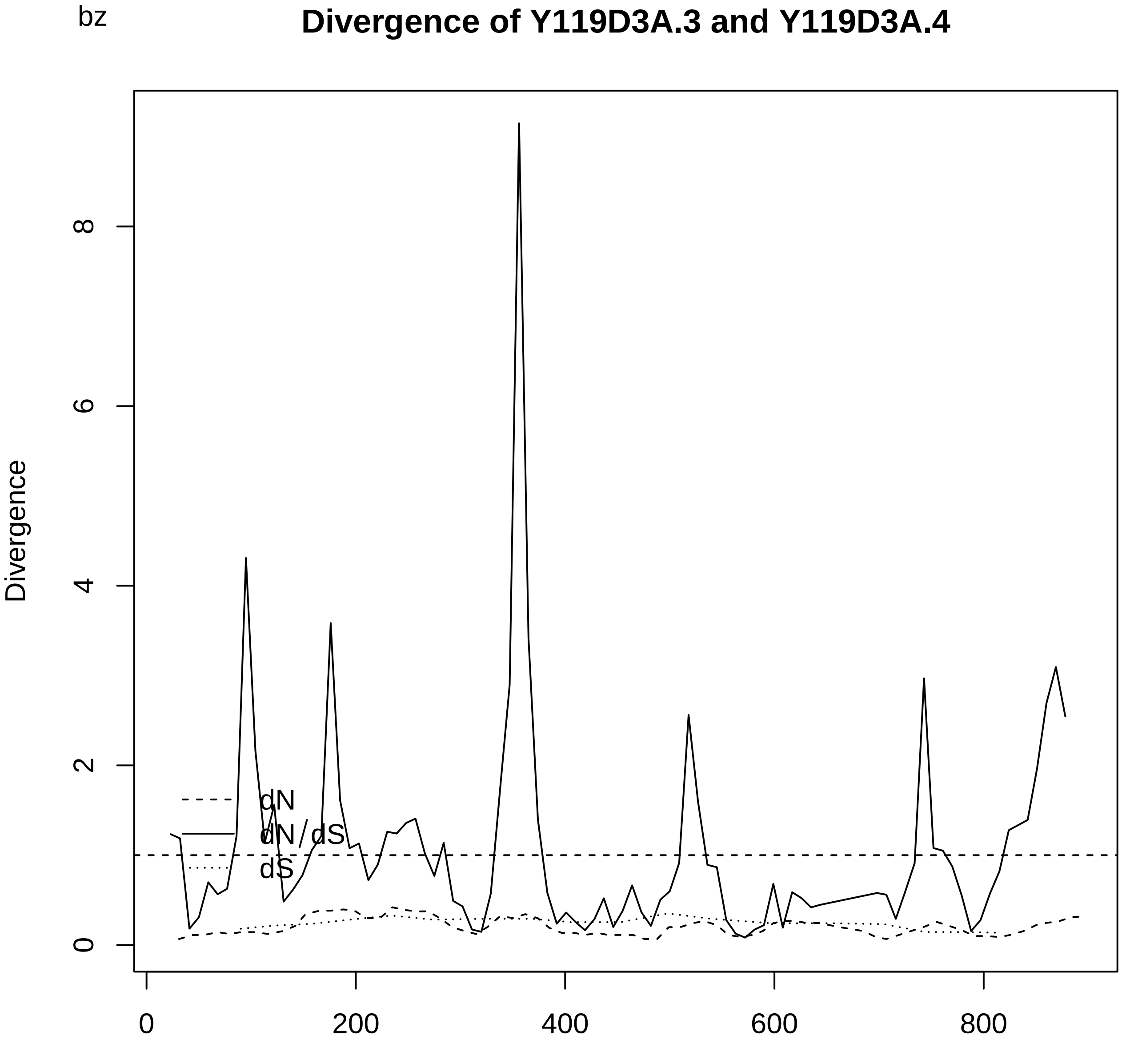
bz
Divergence of Y119D3A.3 and Y119D3A.4
Divergence
0
2
4
6
8
dN
dN/dS
dS
0
200
400
600
800
Nucleotide Position (Aligned,bp)

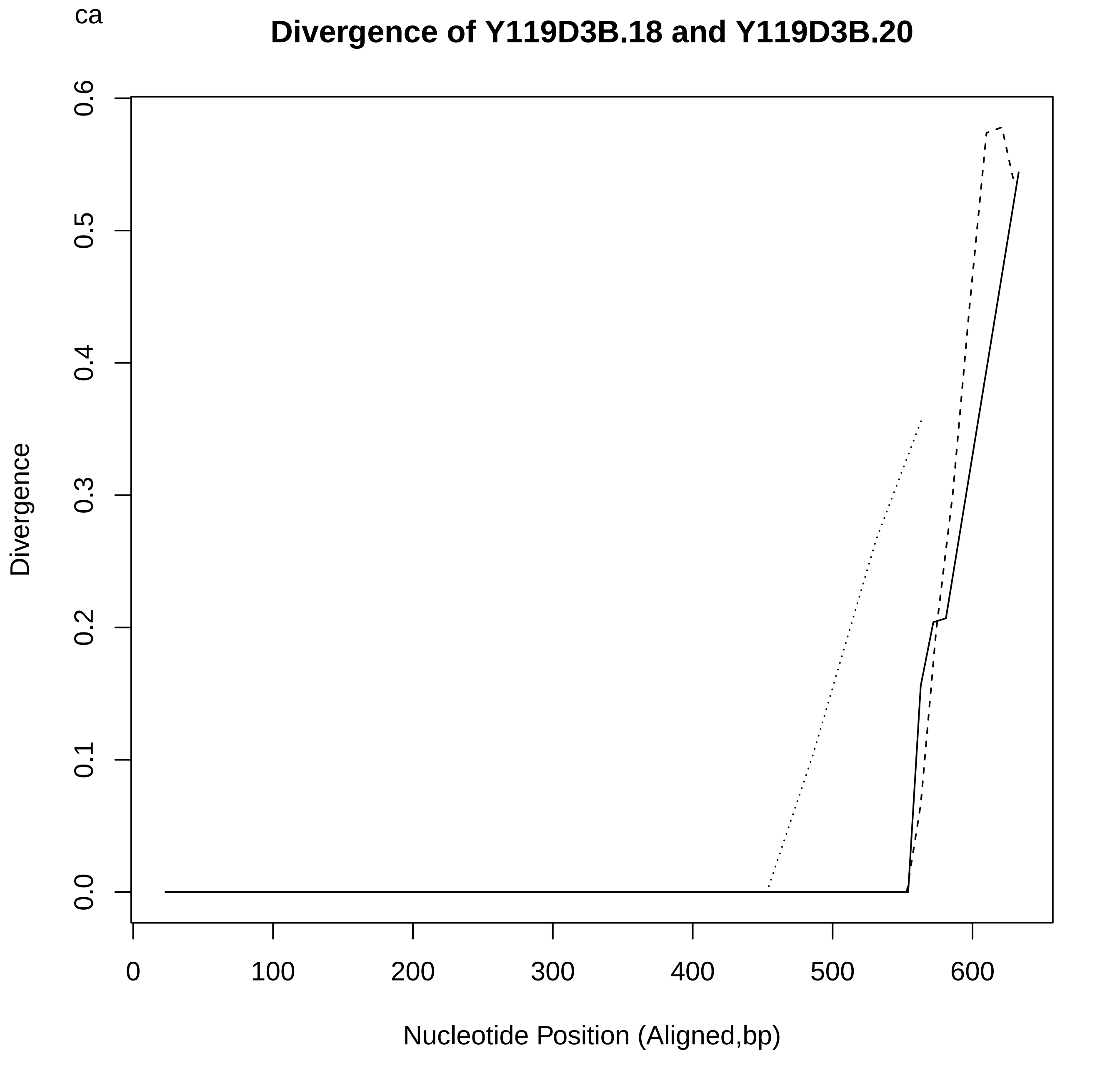
ca
Divergence of Y119D3B.18 and Y119D3B.20
Divergence
0.0
0.1
0.2
0.3
0.4
0.5
0.6
Nucleotide Position (Aligned,bp)
0
100
200
300
400
500
600

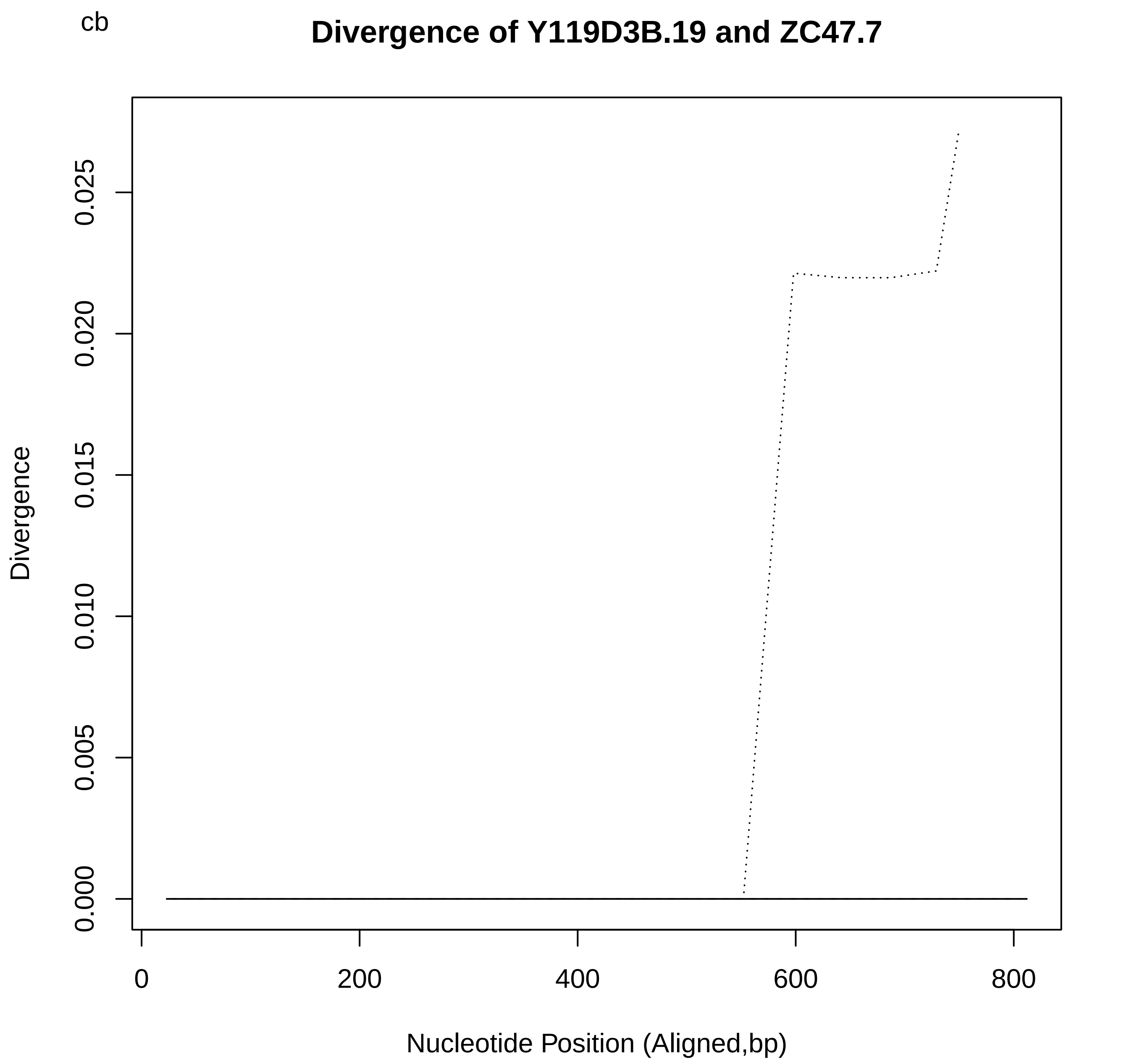
cb
Divergence of Y119D3B.19 and ZC47.7
Divergence
0.000
0.005
0.010
0.015
0.020
0.025
0
200
400
600
800
Nucleotide Position (Aligned,bp)

cc

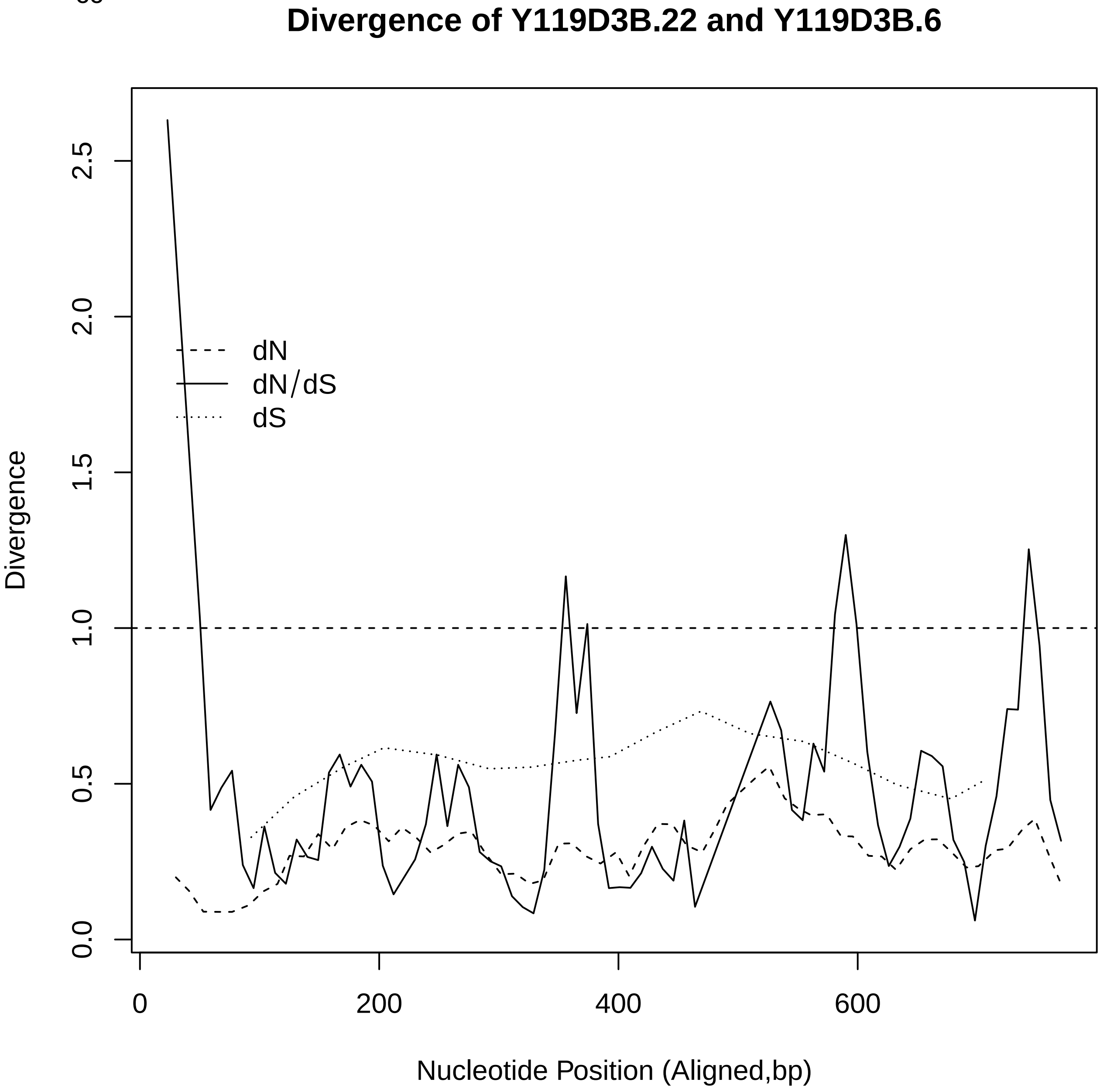
Divergence of Y119D3B.22 and Y119D3B.6
dN
dN/dS
dS
Divergence
0.0
0.5
1.0
1.5
2.0
2.5
0
200
400
600
Nucleotide Position (Aligned,bp)

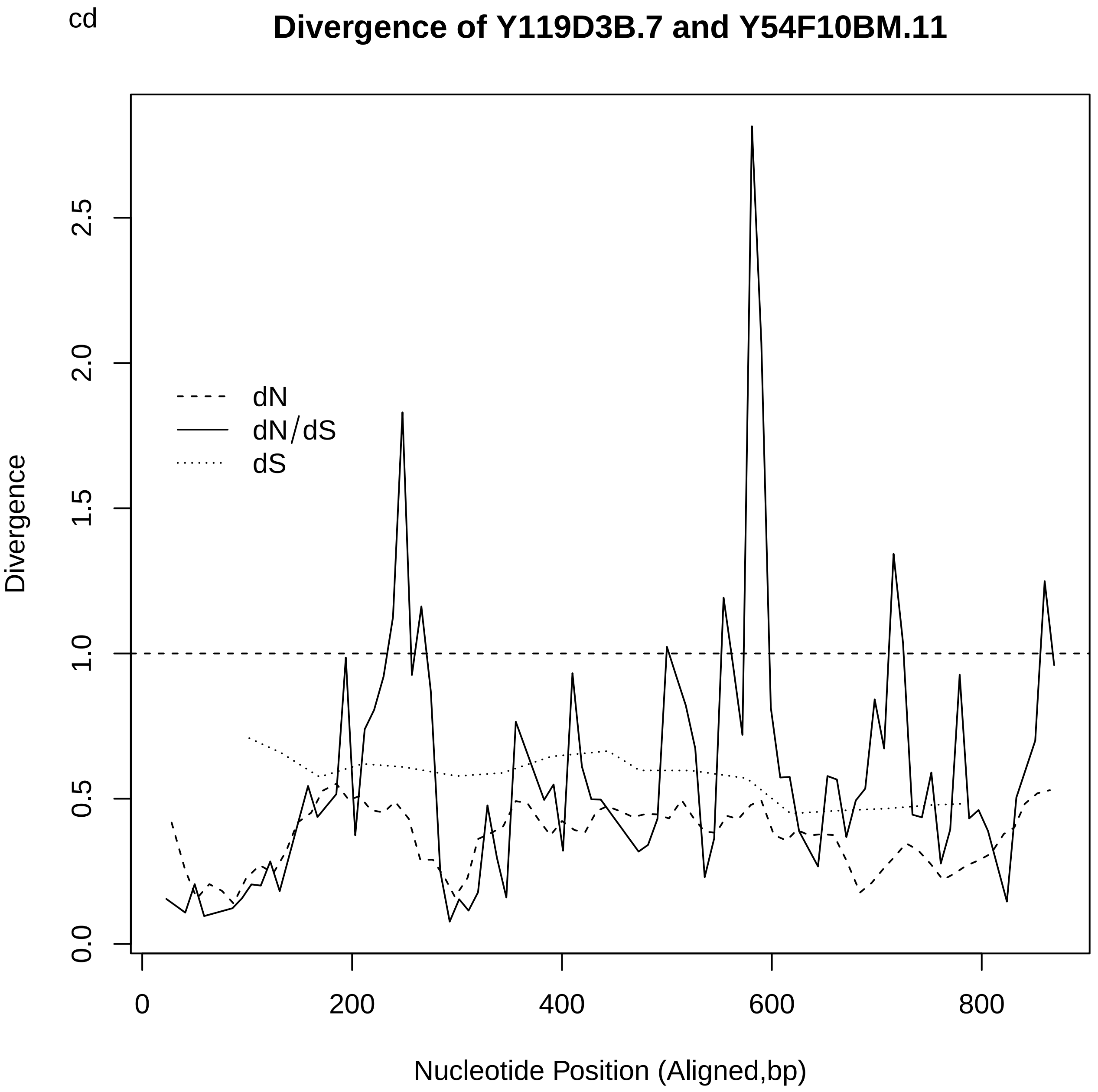
cd
Divergence of Y119D3B.7 and Y54F10BM.11
dN
dN/dS
dS
Divergence
0.0
0.5
1.0
1.5
2.0
2.5
0
200
400
600
800
Nucleotide Position (Aligned,bp)

ce

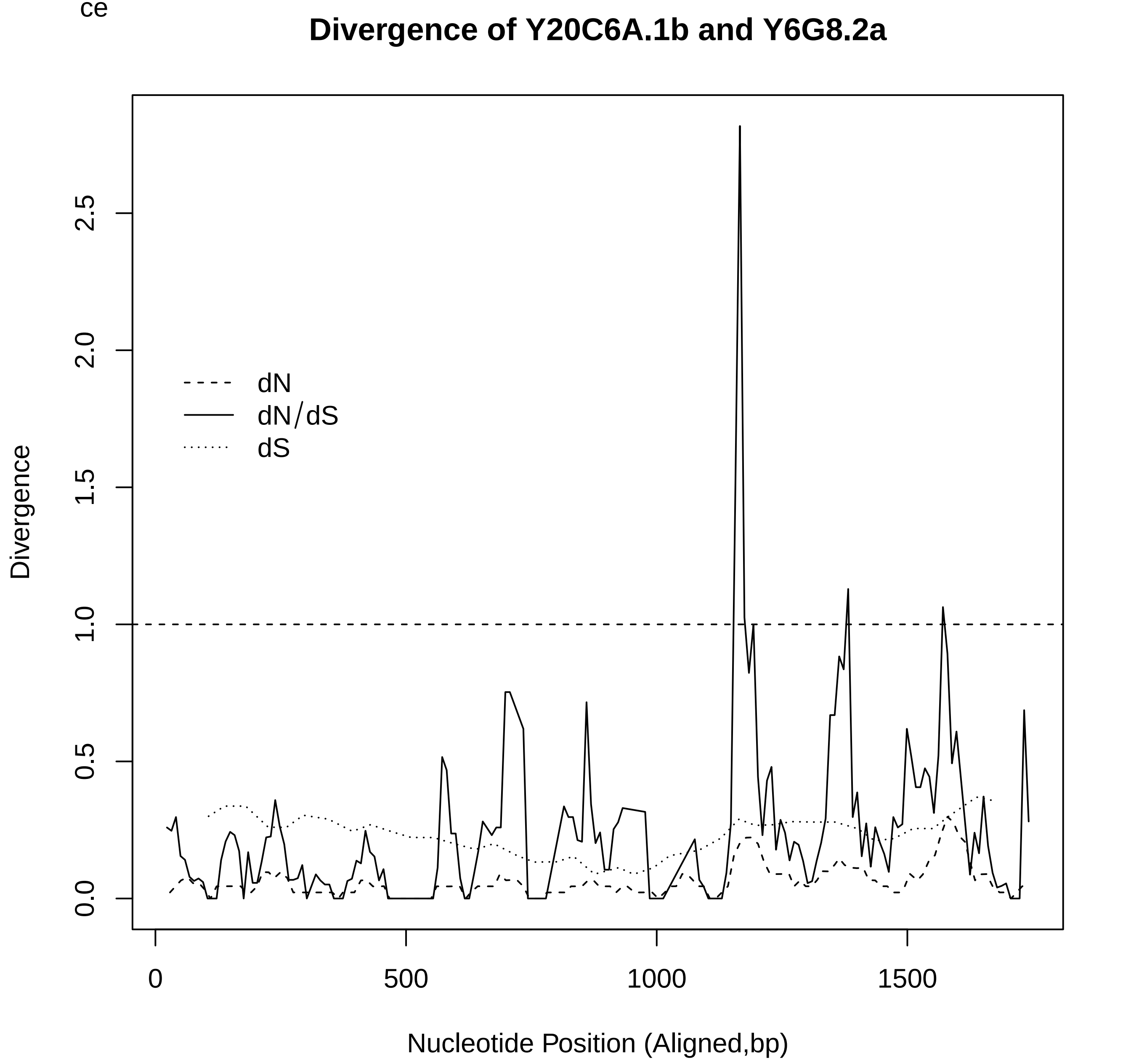

Divergence of Y20C6A.1b and Y6G8.2a
dN
dN/dS
dS
Divergence
0.0
0.5
1.0
1.5
2.0
2.5
0
500
1000
1500
Nucleotide Position (Aligned,bp)

cf

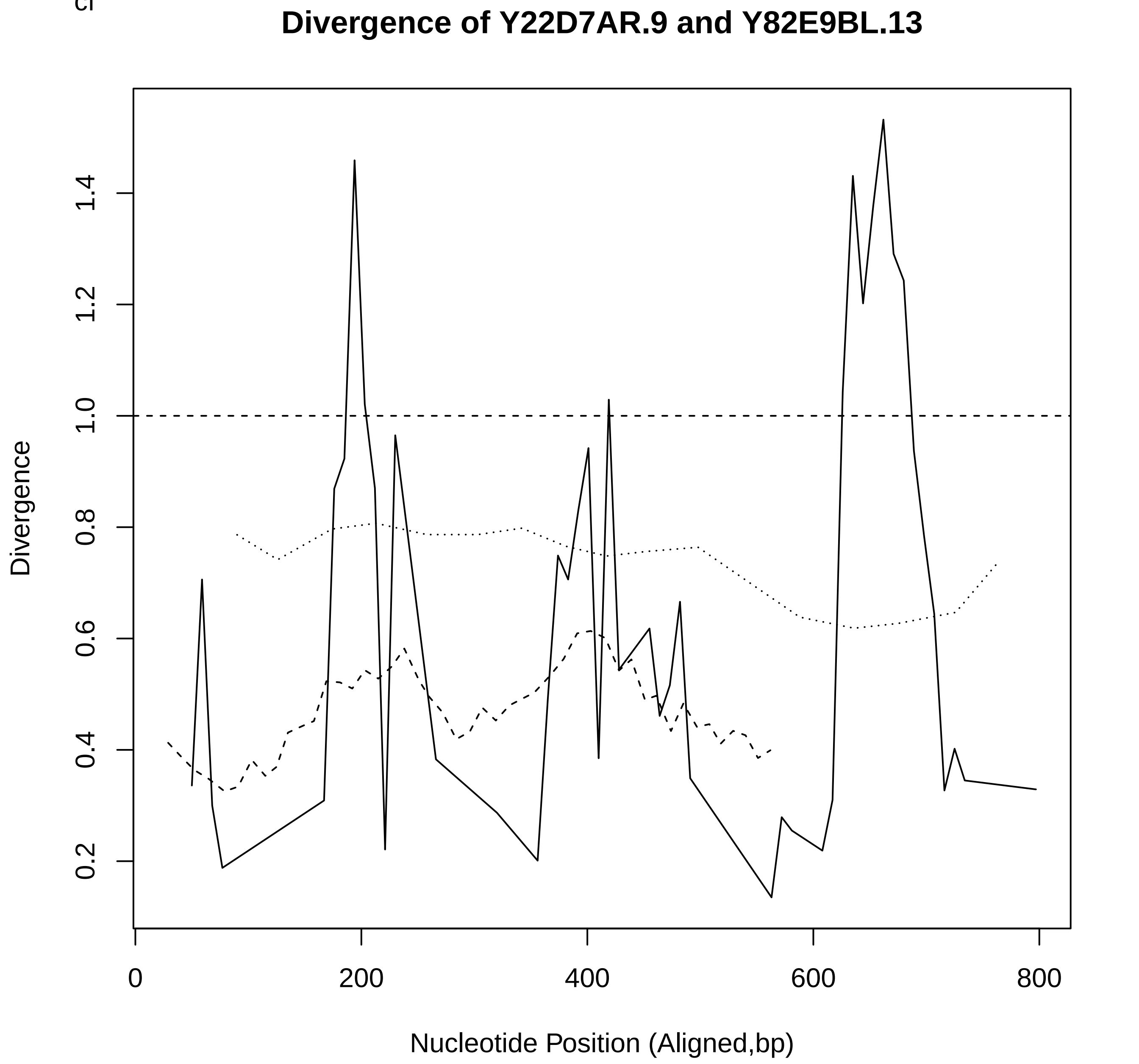

Divergence of Y22D7AR.9 and Y82E9BL.13
Divergence
0.2
0.4
0.6
0.8
1.0
1.2
1.4
0
200
400
600
800
Nucleotide Position (Aligned,bp)

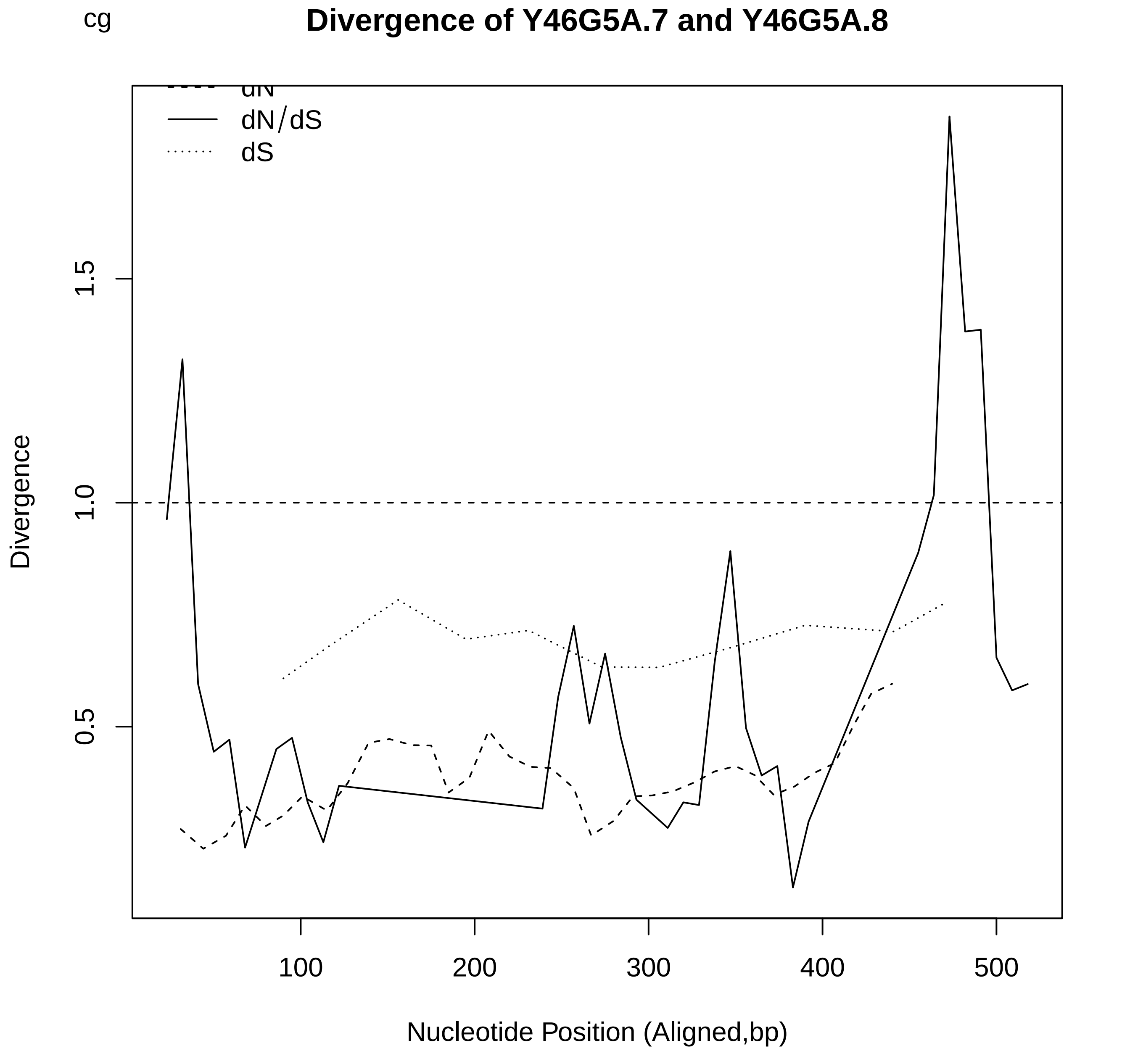
cg
Divergence of Y46G5A.7 and Y46G5A.8
dN
dN/dS
dS
Divergence
0.5
1.0
1.5
100
200
300
400
500
Nucleotide Position (Aligned,bp)

ch

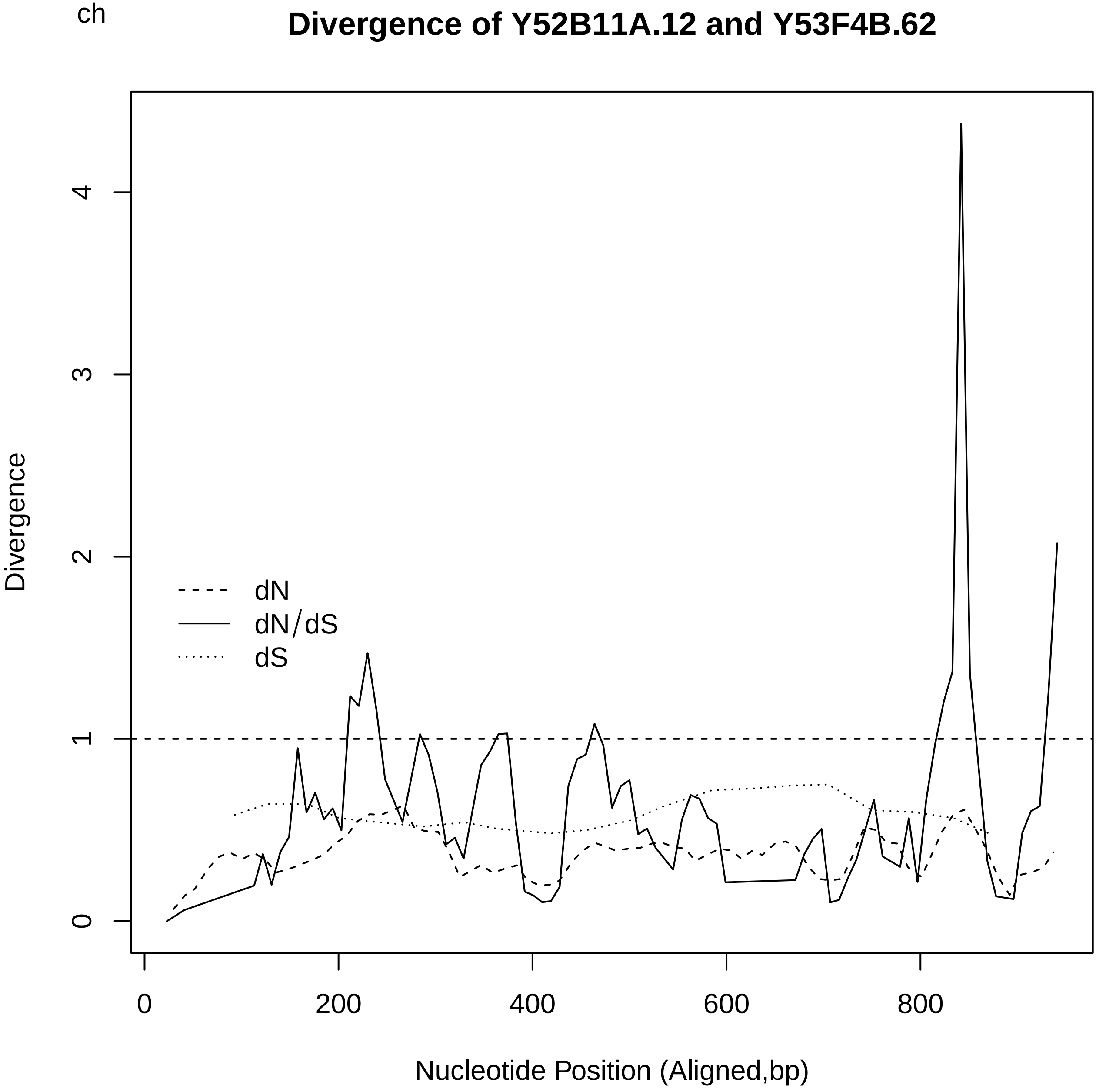

Divergence of Y52B11A.12 and Y53F4B.62
Divergence
0
1
2
3
4
dN
dN/dS
dS
0
200
400
600
800
Nucleotide Position (Aligned,bp)

ci

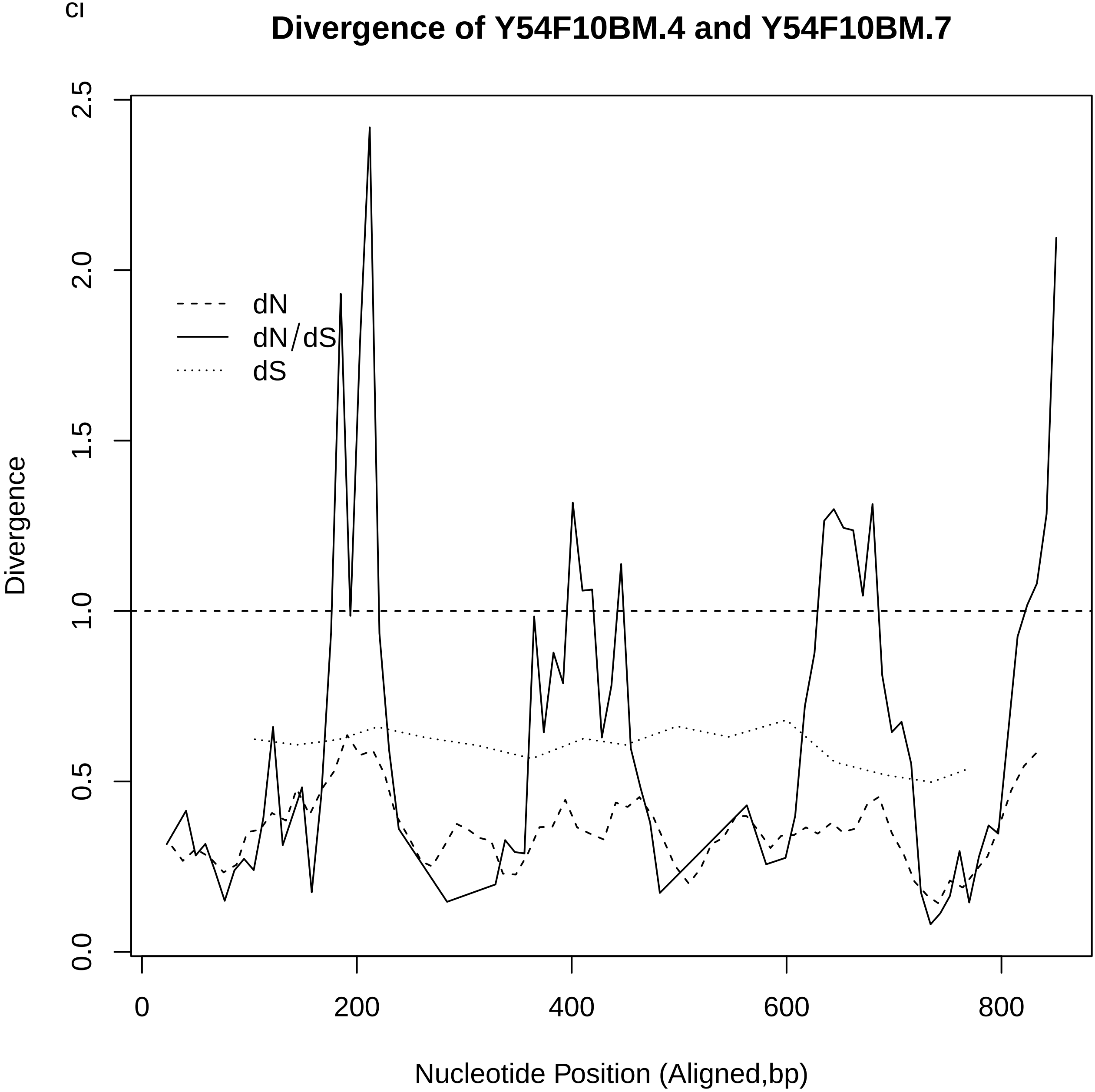
Divergence of Y54F10BM.4 and Y54F10BM.7
dN
dN/dS
dS
Divergence
0.0
0.5
1.0
1.5
2.0
2.5
0
200
400
600
800
Nucleotide Position (Aligned,bp)

cj

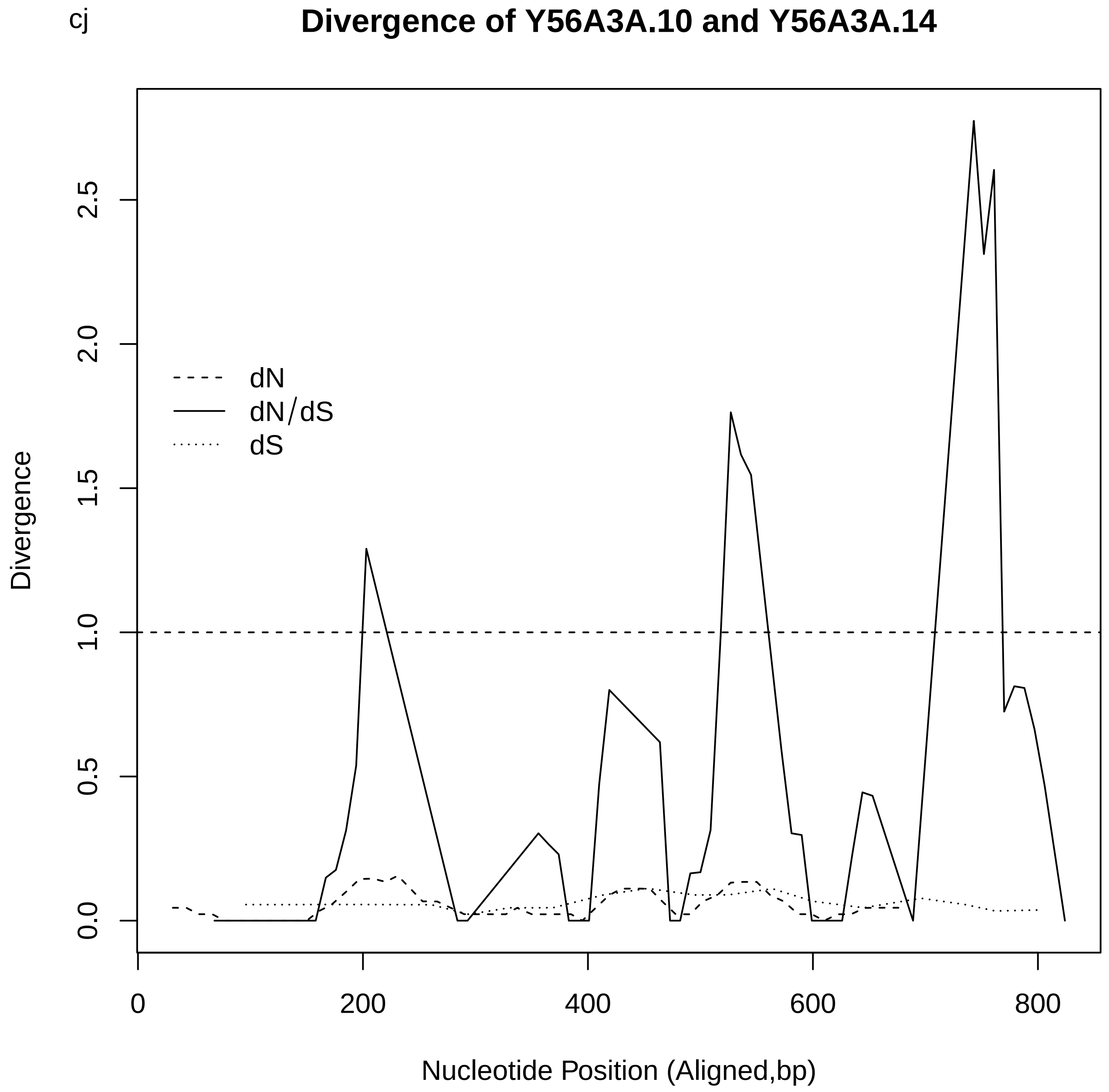

Divergence of Y56A3A.10 and Y56A3A.14
dN
dN/dS
dS
Divergence
0.0
0.5
1.0
1.5
2.0
2.5
0
200
400
600
800
Nucleotide Position (Aligned,bp)

ck

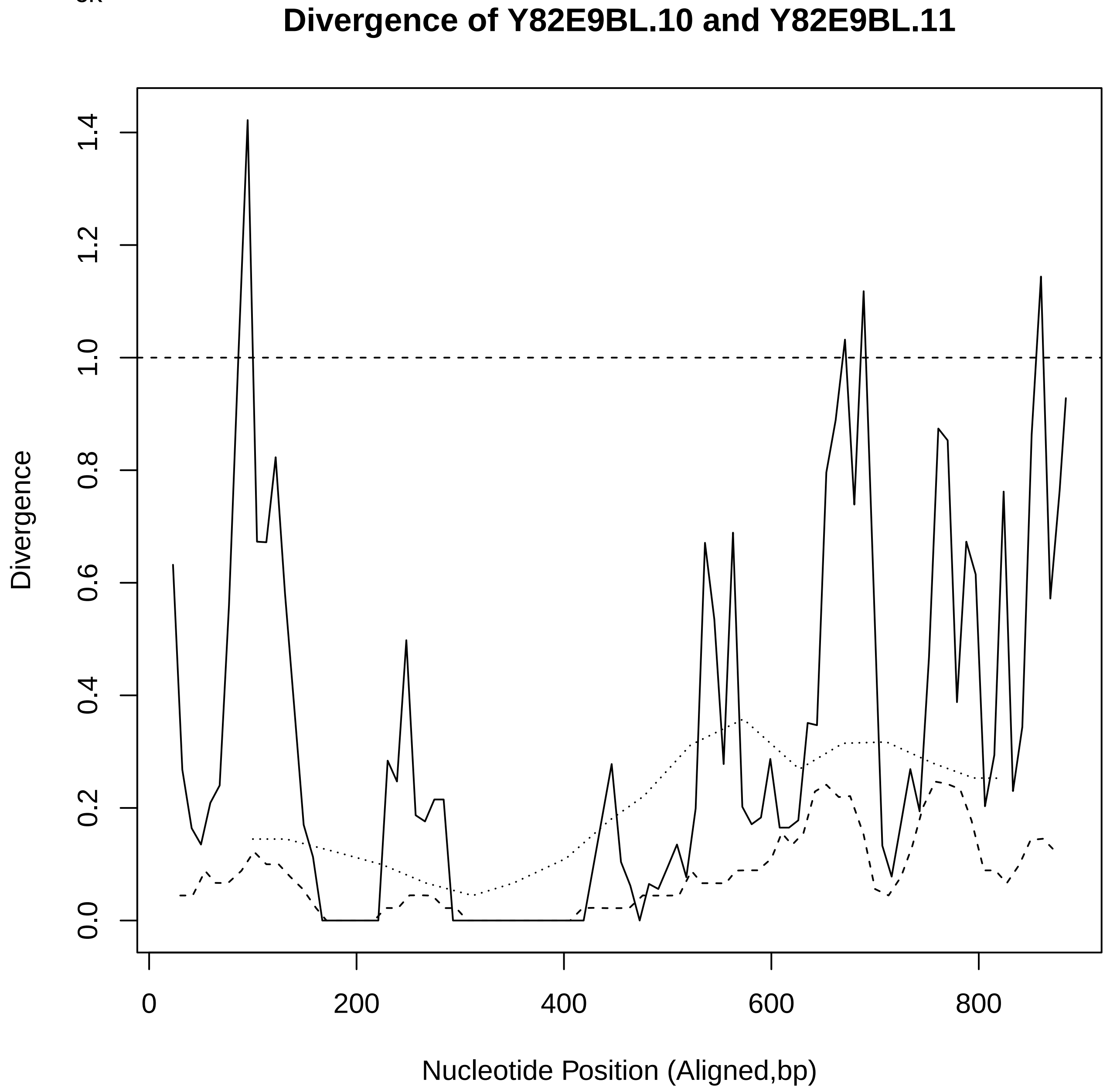

Divergence of Y82E9BL.10 and Y82E9BL.11
Divergence
Nucleotide Position (Aligned,bp)
0.0
0.2
0.4
0.6
0.8
1.0
1.2
1.4
0
200
400
600
800

cl

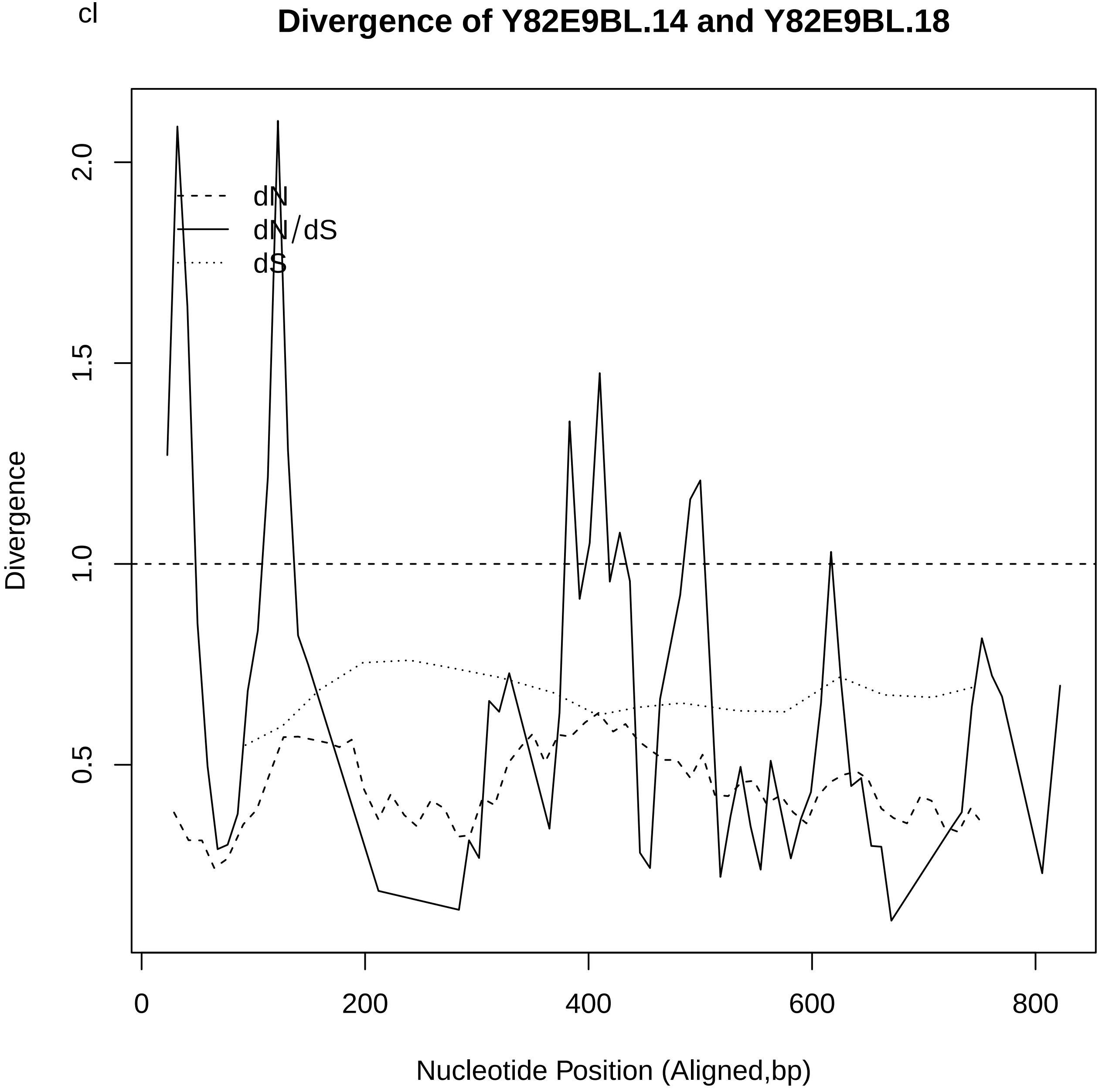

Divergence of Y82E9BL.14 and Y82E9BL.18
dN
dN/dS
dS
Divergence
2.0
1.5
1.0
0.5
0
200
400
600
800
Nucleotide Position (Aligned,bp)

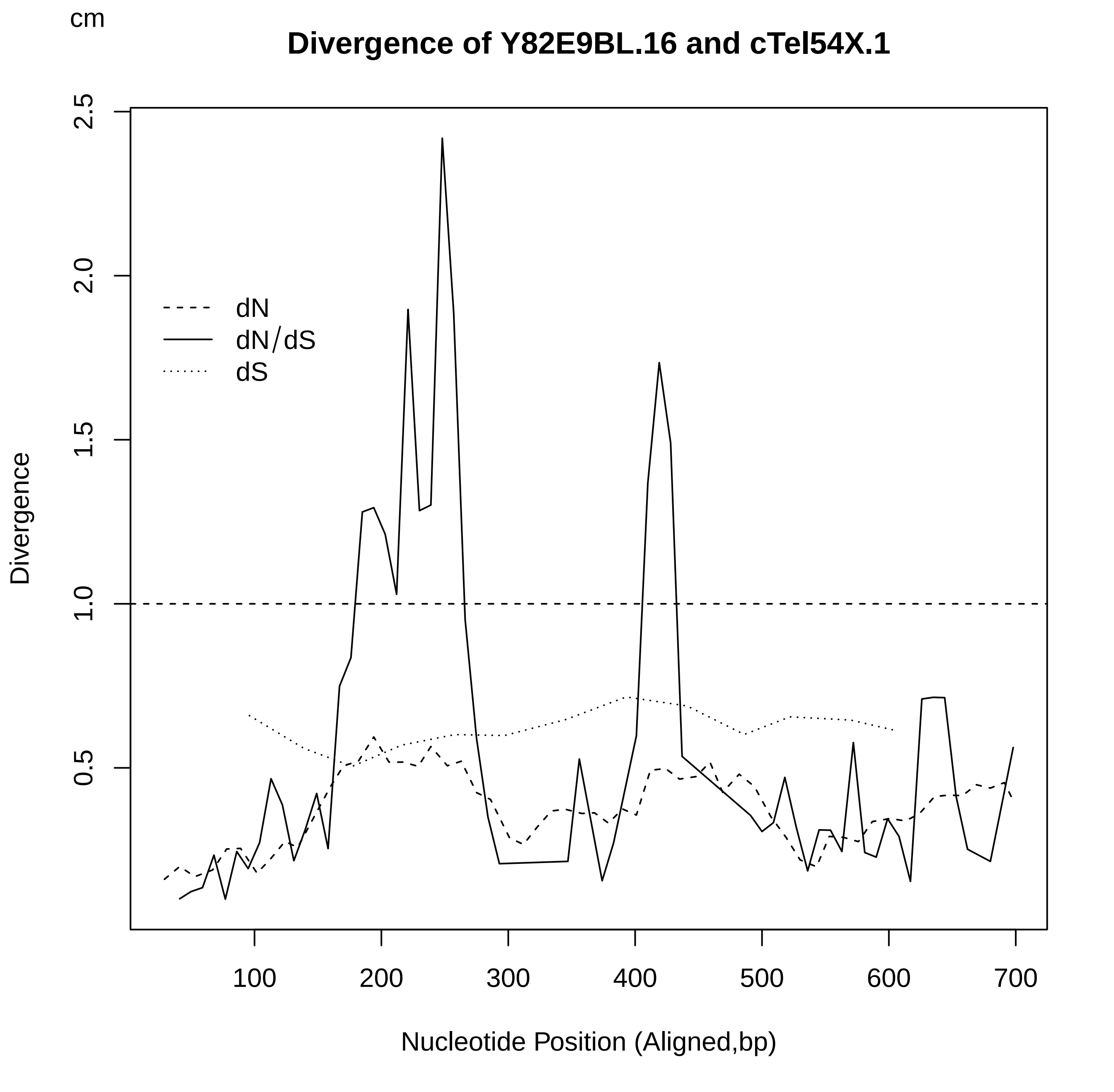
cm
Divergence of Y82E9BL.16 and cTel54X.1
dN
dN/dS
dS
Divergence
2.5
2.0
1.5
1.0
0.5
100
200
300
400
500
600
700
Nucleotide Position (Aligned,bp)

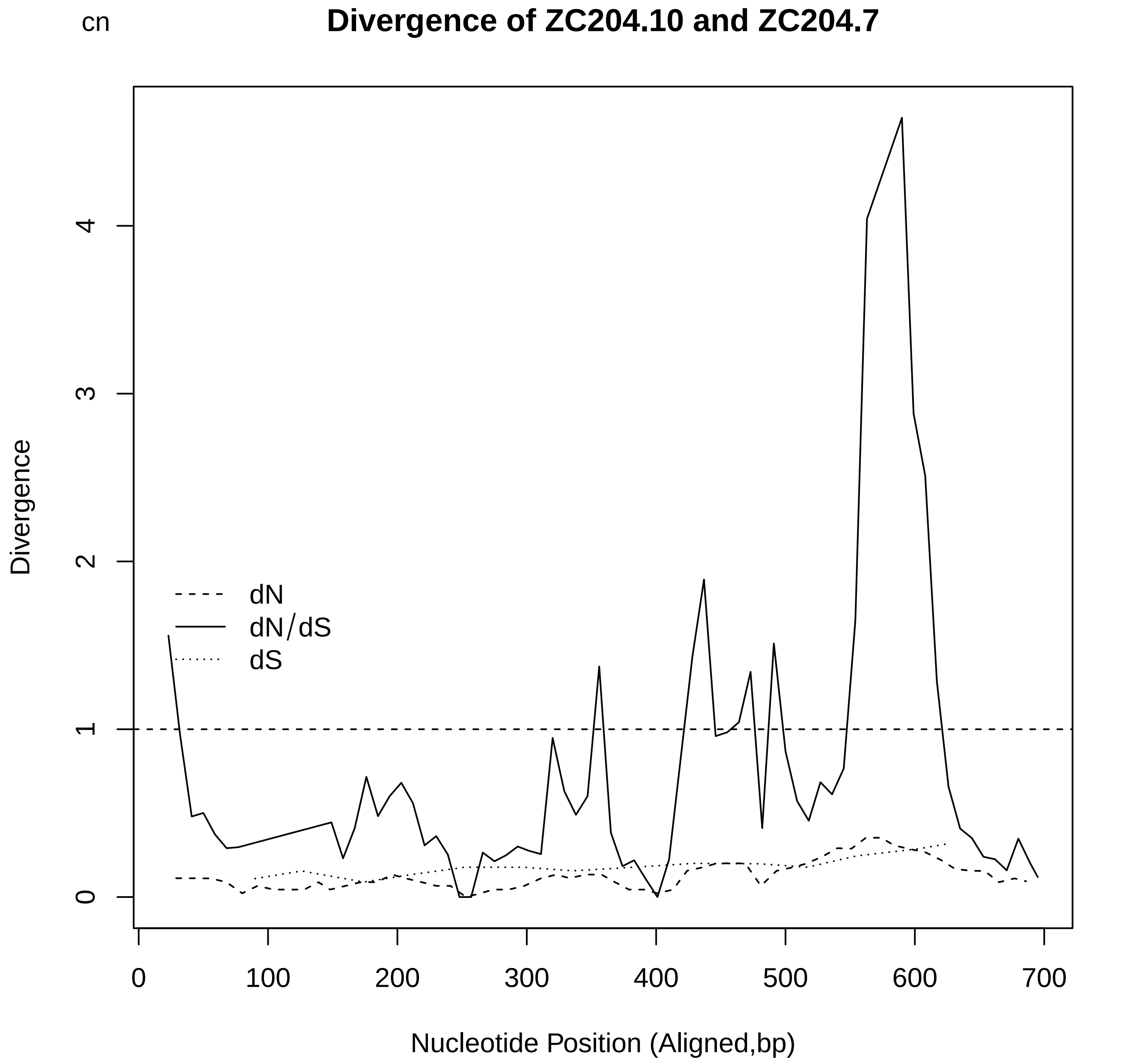
cn
Divergence of ZC204.10 and ZC204.7
Divergence
0
1
2
3
4
dN
dN/dS
dS
0
100
200
300
400
500
600
700
Nucleotide Position (Aligned,bp)

co

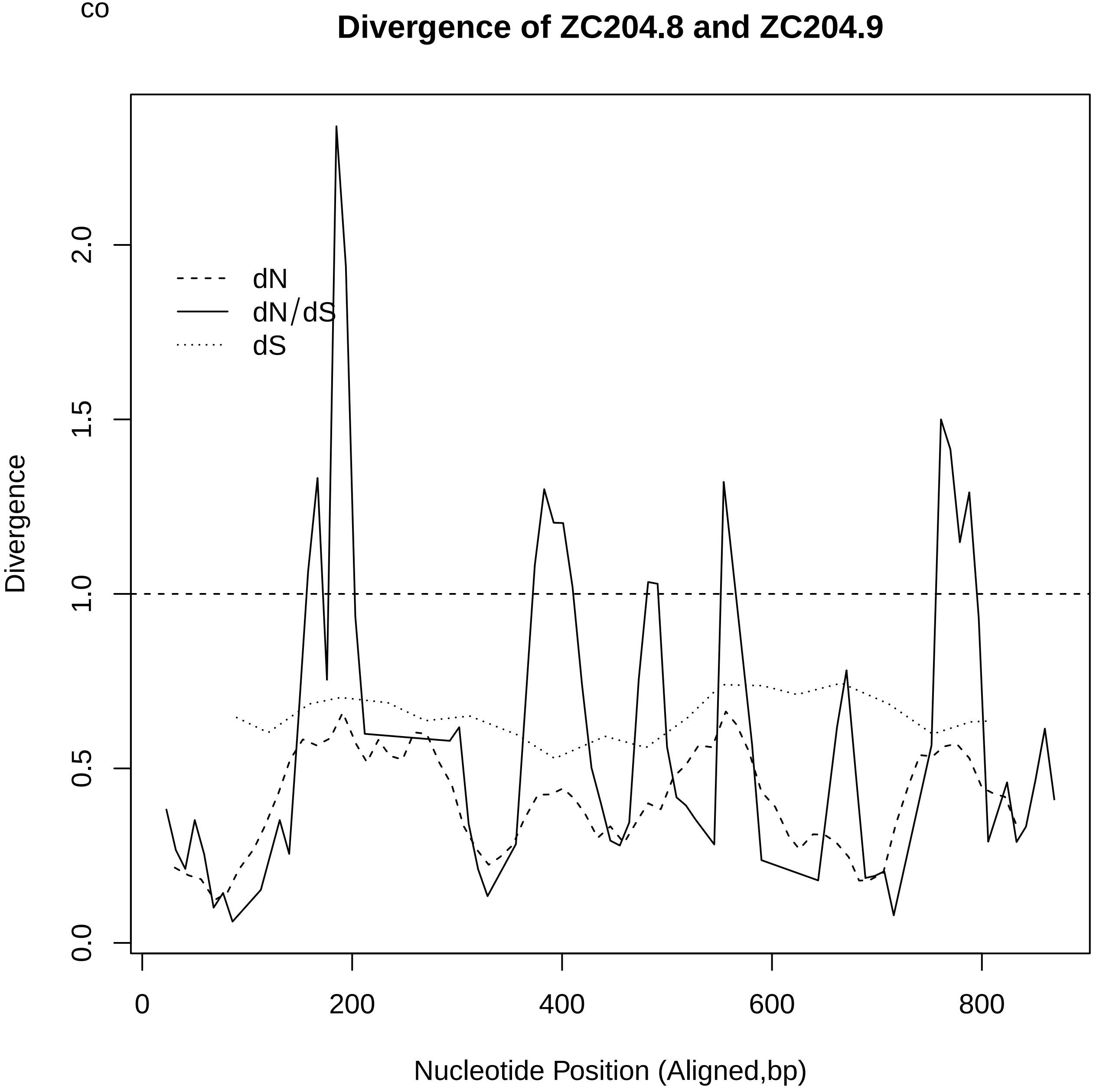
Divergence of ZC204.8 and ZC204.9
dN
dN/dS
dS
Divergence
0.0
0.5
1.0
1.5
2.0
0
200
400
600
800
Nucleotide Position (Aligned,bp)

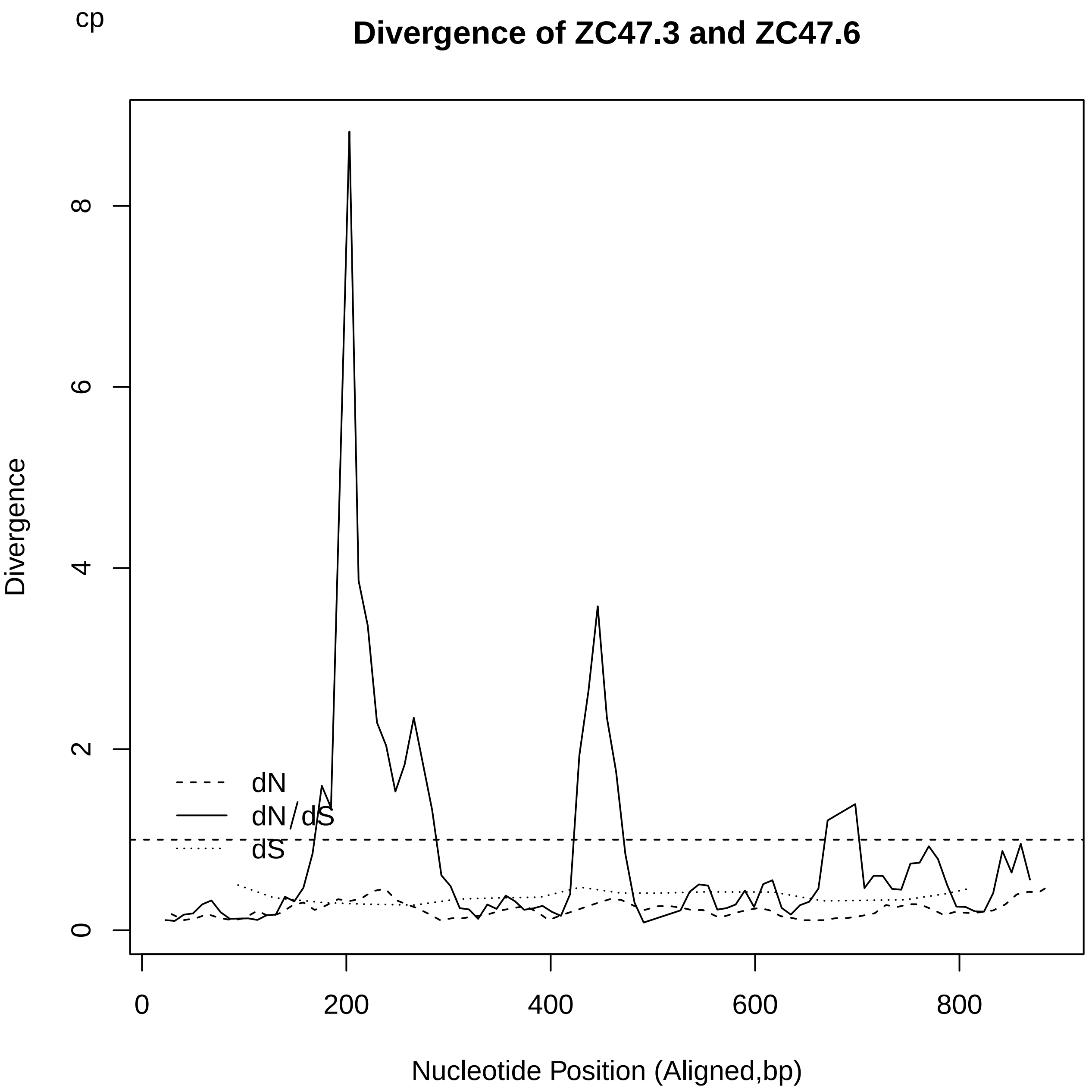
cp
Divergence of ZC47.3 and ZC47.6
Divergence
0
2
4
6
8
dN
dN/dS
dS
0
200
400
600
800
Nucleotide Position (Aligned,bp)

cq

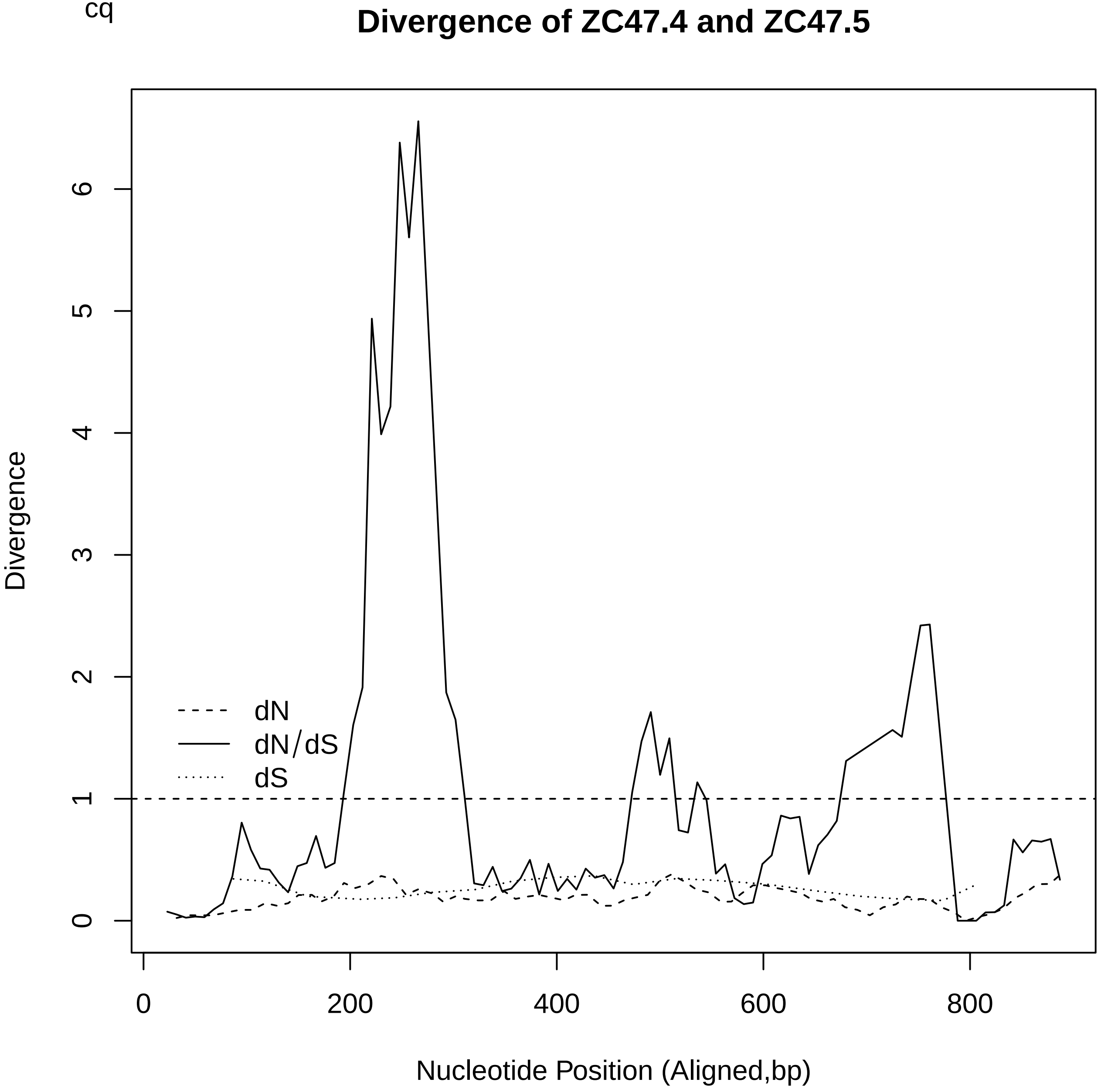
Divergence of ZC47.4 and ZC47.5
Divergence
Nucleotide Position (Aligned,bp)
dN
dN/dS
dS
0
1
2
3
4
5
6
0
200
400
600
800
