# Supplementary material for: Genome-wide characterization, evolution, structure, and expression analysis of the F-box genes in Caenorhabditis
Source: BMC Genomics. 2021 Dec 11;22:889. doi: 10.1186/s12864-021-08189-7 (PMC8665587; doi:10.1186/s12864-021-08189-7)
Supplement: Supplementary file 11 — Additional file 11: Figure S11. Sliding-window plots of dS, dN, and dN/dS in pairwise comparisons of 37 closely related paralogs of F-box genes from C. briggsae. The window size is 45 codons, and the offset between windows is nine codons. The solid line represents plots of dN/dS, the short-dotted line indicates plots of dS, and the long-dotted line indicates plots of dN. [file 12864_2021_8189_MOESM11_ESM.pdf]

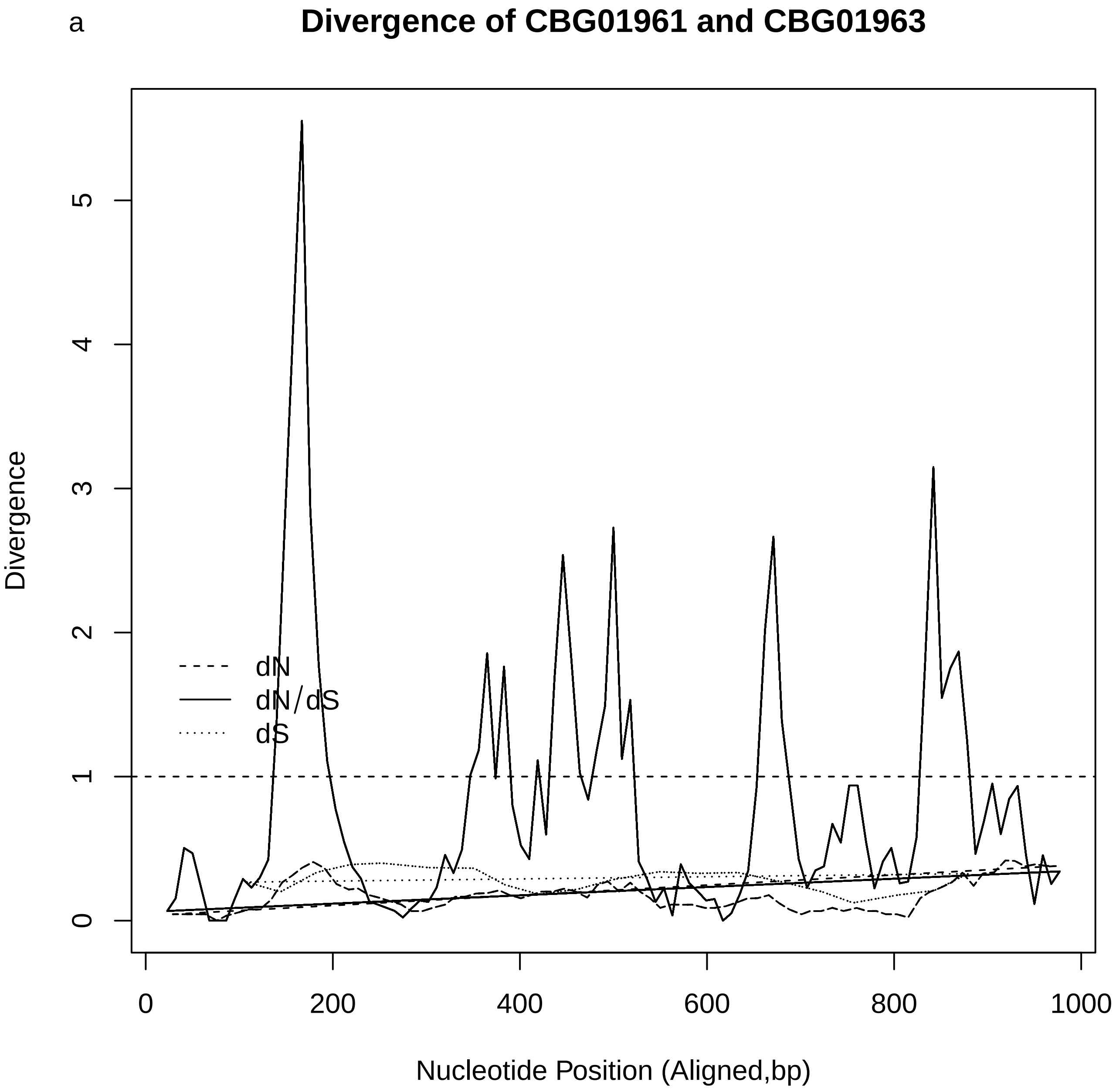
a
Divergence of CBG01961 and CBG01963
Divergence
Nucleotide Position (Aligned,bp)
dN
dN/dS
dS
0
1
2
3
4
5
0
200
400
600
800
1000

b

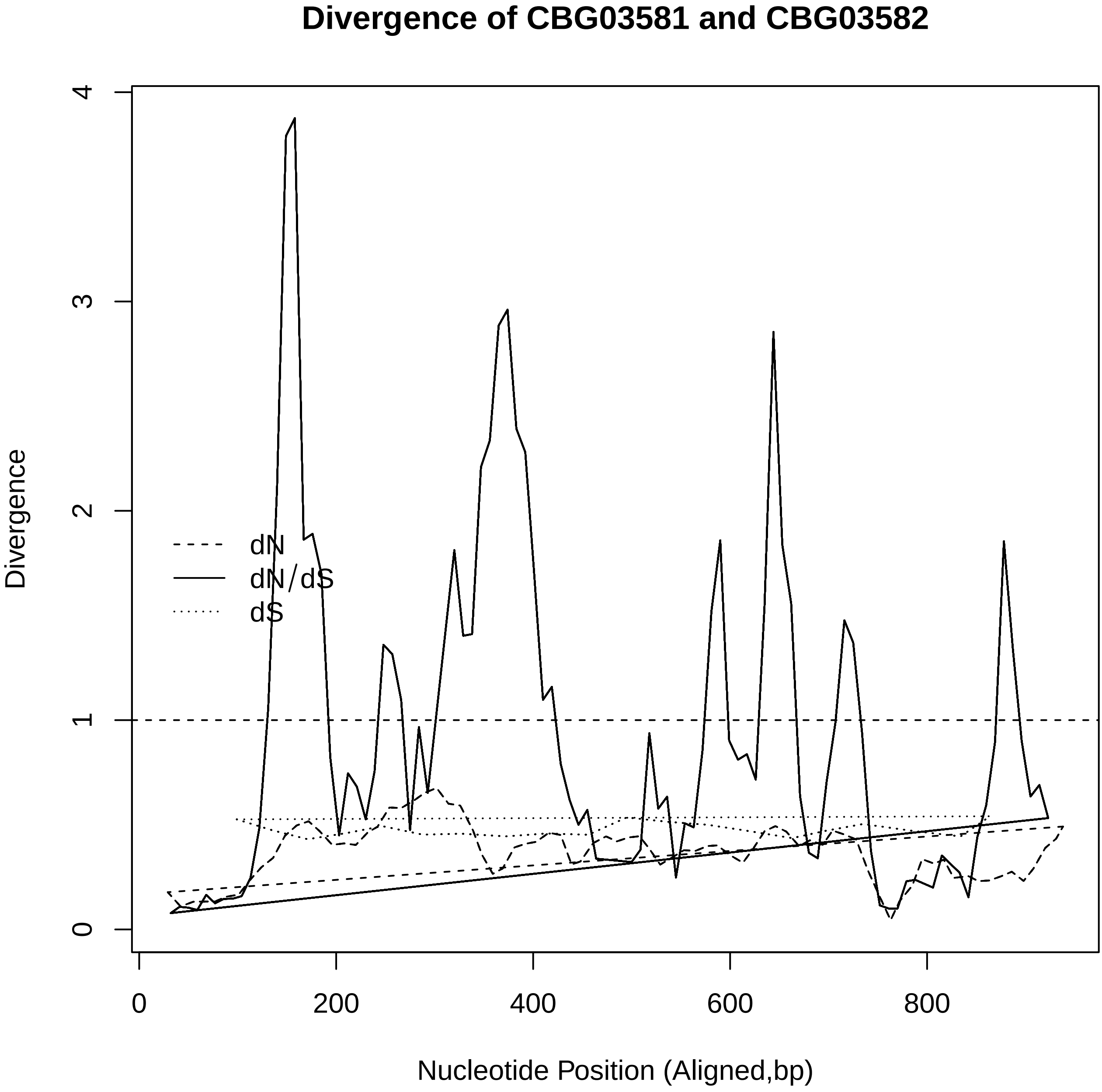
Divergence of CBG03581 and CBG03582
Divergence
4
3
2
1
0
dN
dN/dS
dS
0
200
400
600
800
Nucleotide Position (Aligned,bp)

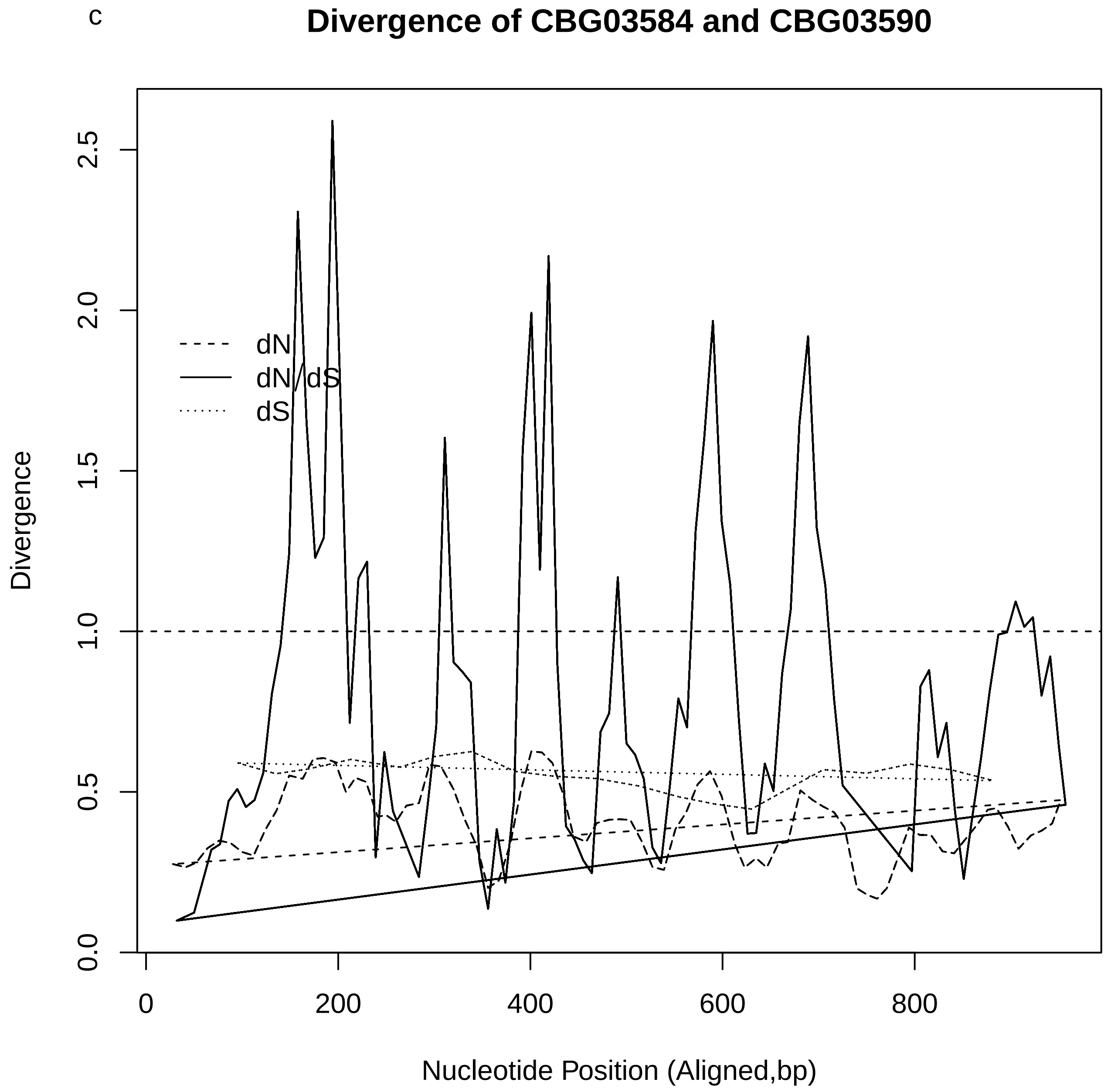
c
Divergence of CBG03584 and CBG03590
Divergence
Nucleotide Position (Aligned,bp)
dN
dN/dS
dS
0.0
0.5
1.0
1.5
2.0
2.5
0
200
400
600
800

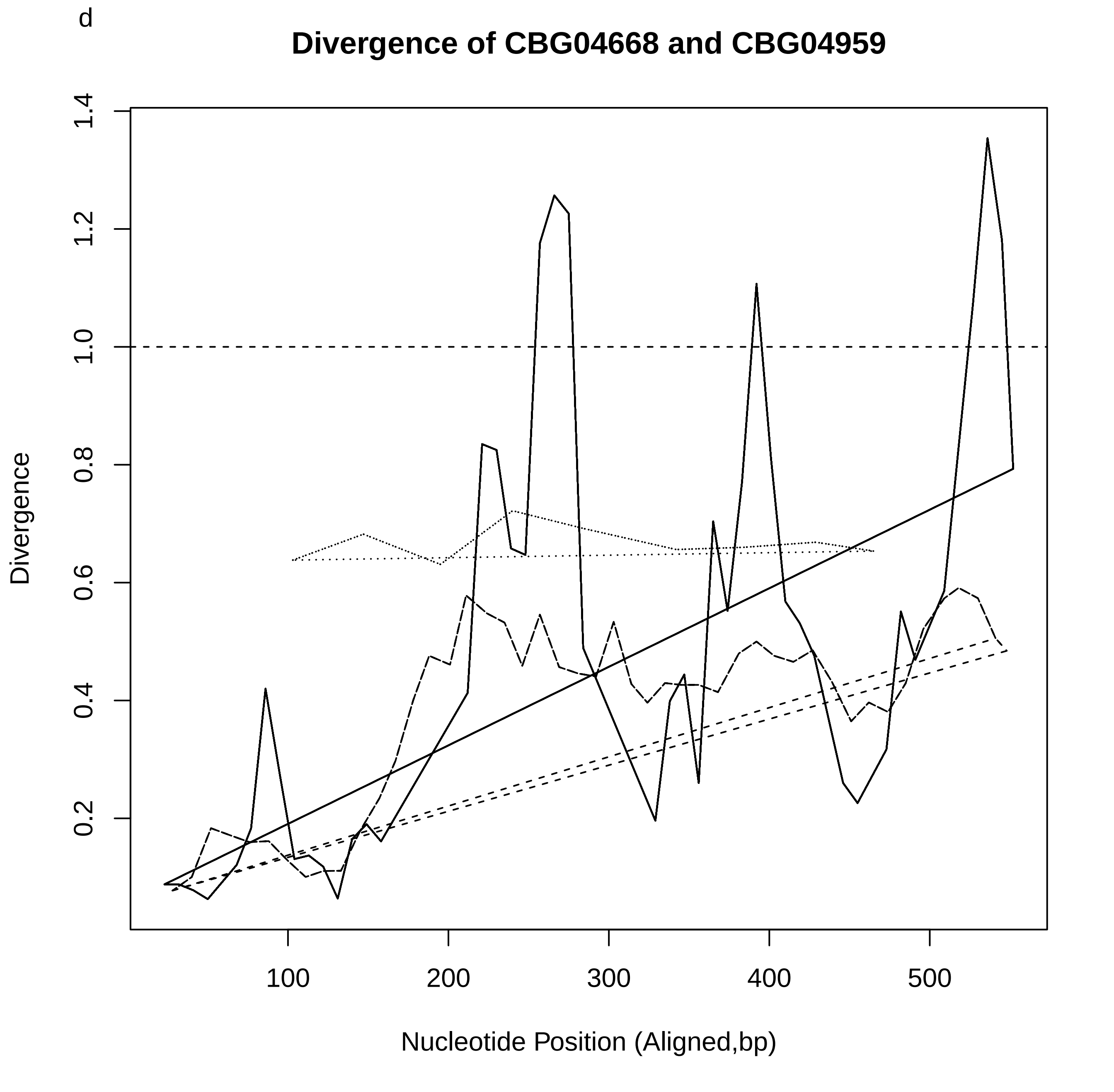
d
Divergence of CBG04668 and CBG04959
Divergence
0.2
0.4
0.6
0.8
1.0
1.2
1.4
100
200
300
400
500
Nucleotide Position (Aligned,bp)

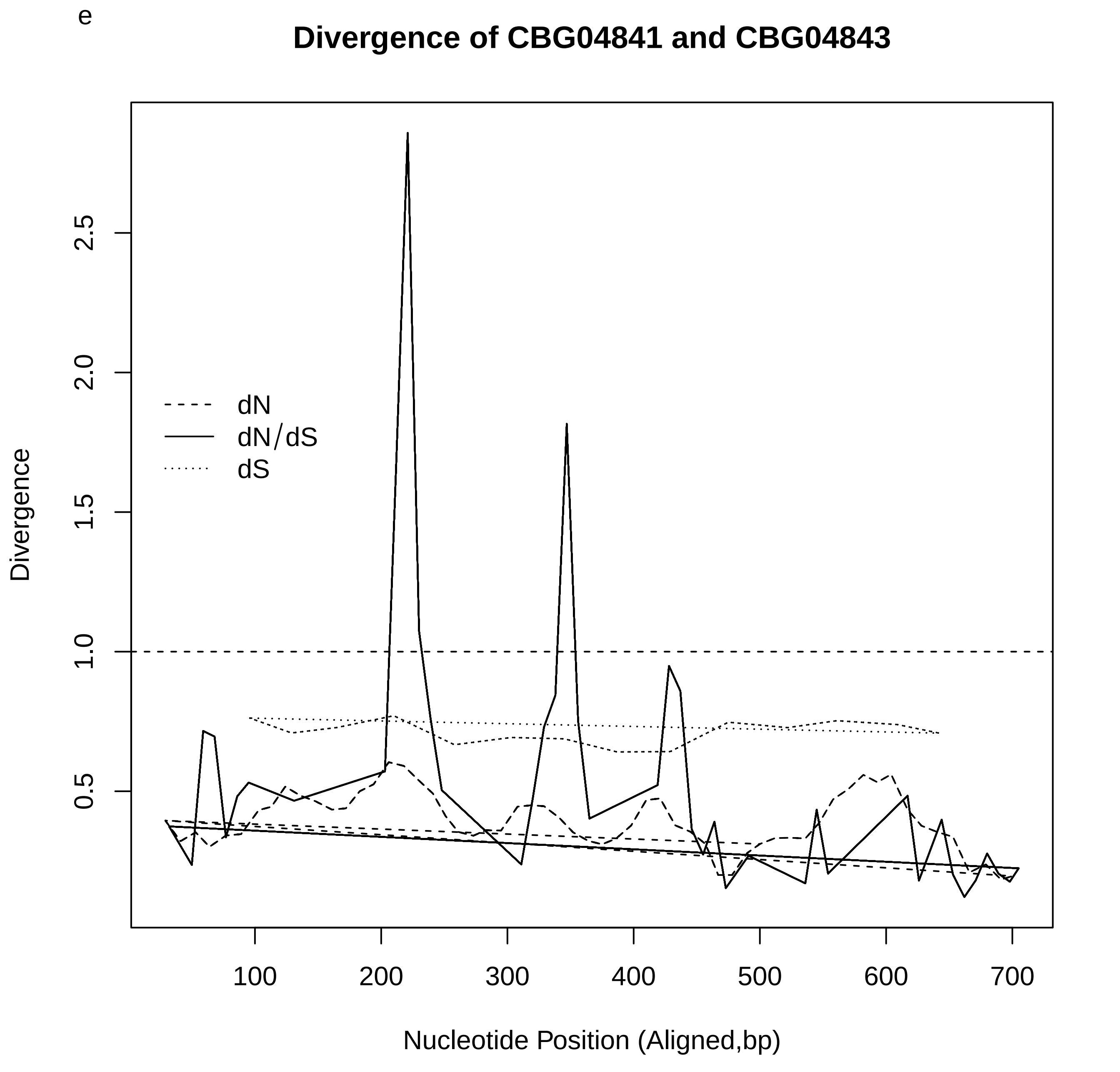
e
Divergence of CBG04841 and CBG04843
Divergence
Nucleotide Position (Aligned,bp)
dN
dN/dS
dS
0.5
1.0
1.5
2.0
2.5
100
200
300
400
500
600
700

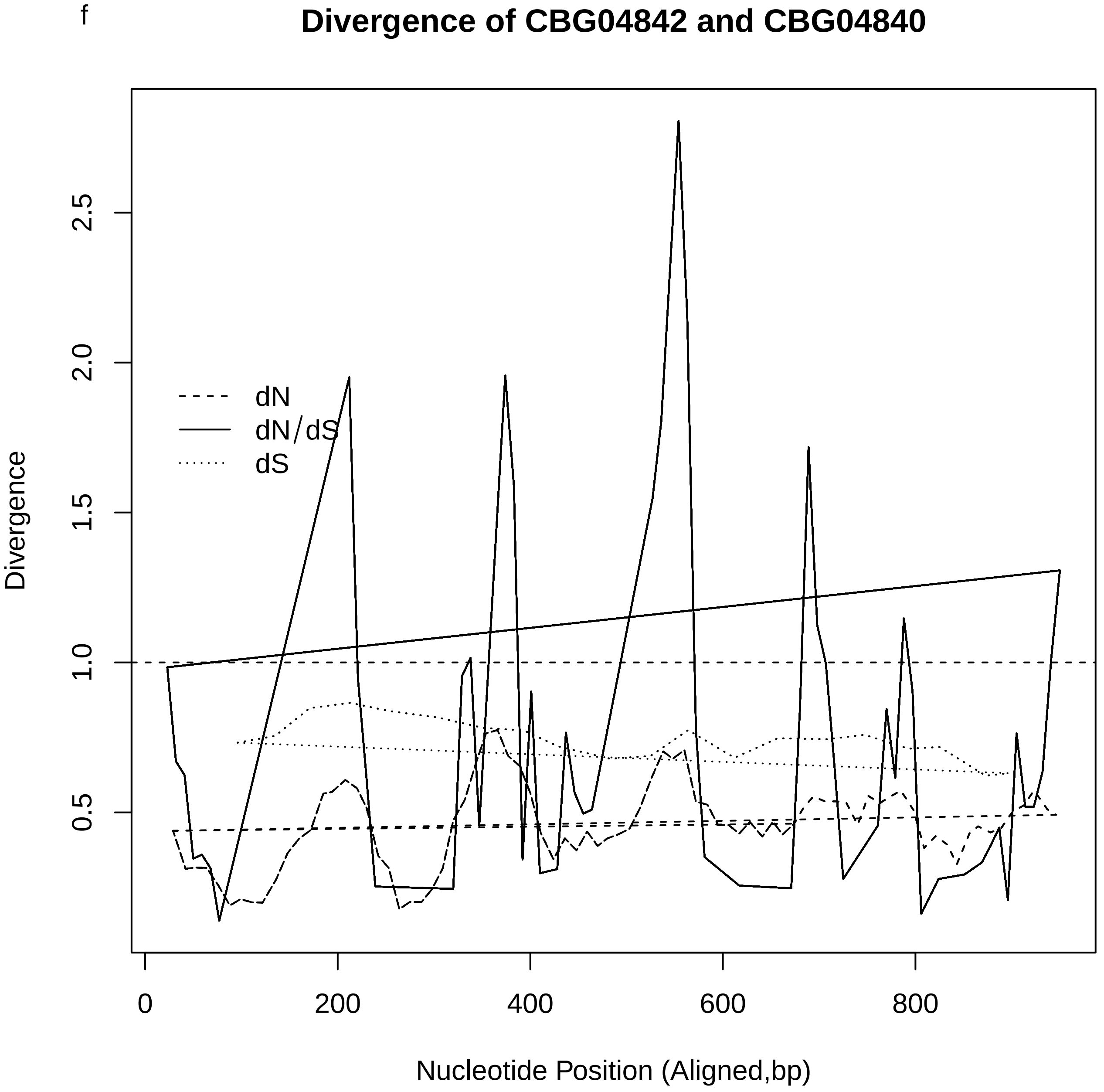
f
Divergence of CBG04842 and CBG04840
dN
dN/dS
dS
Divergence
Nucleotide Position (Aligned,bp)
0
200
400
600
800
0.5
1.0
1.5
2.0
2.5

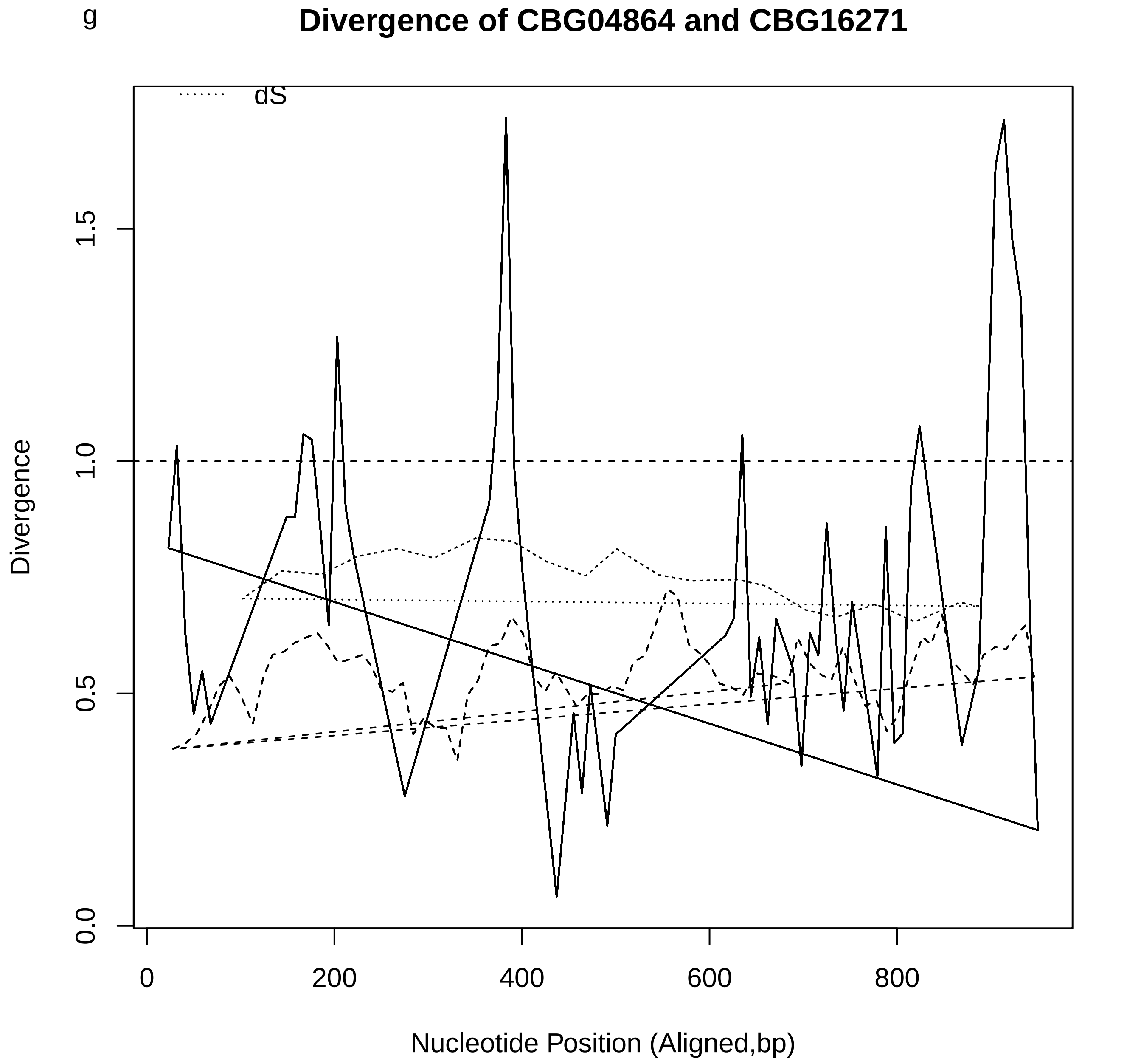

g
Divergence of CBG04864 and CBG16271
dS
Divergence
0.0
0.5
1.0
1.5
0
200
400
600
800
Nucleotide Position (Aligned,bp)

h

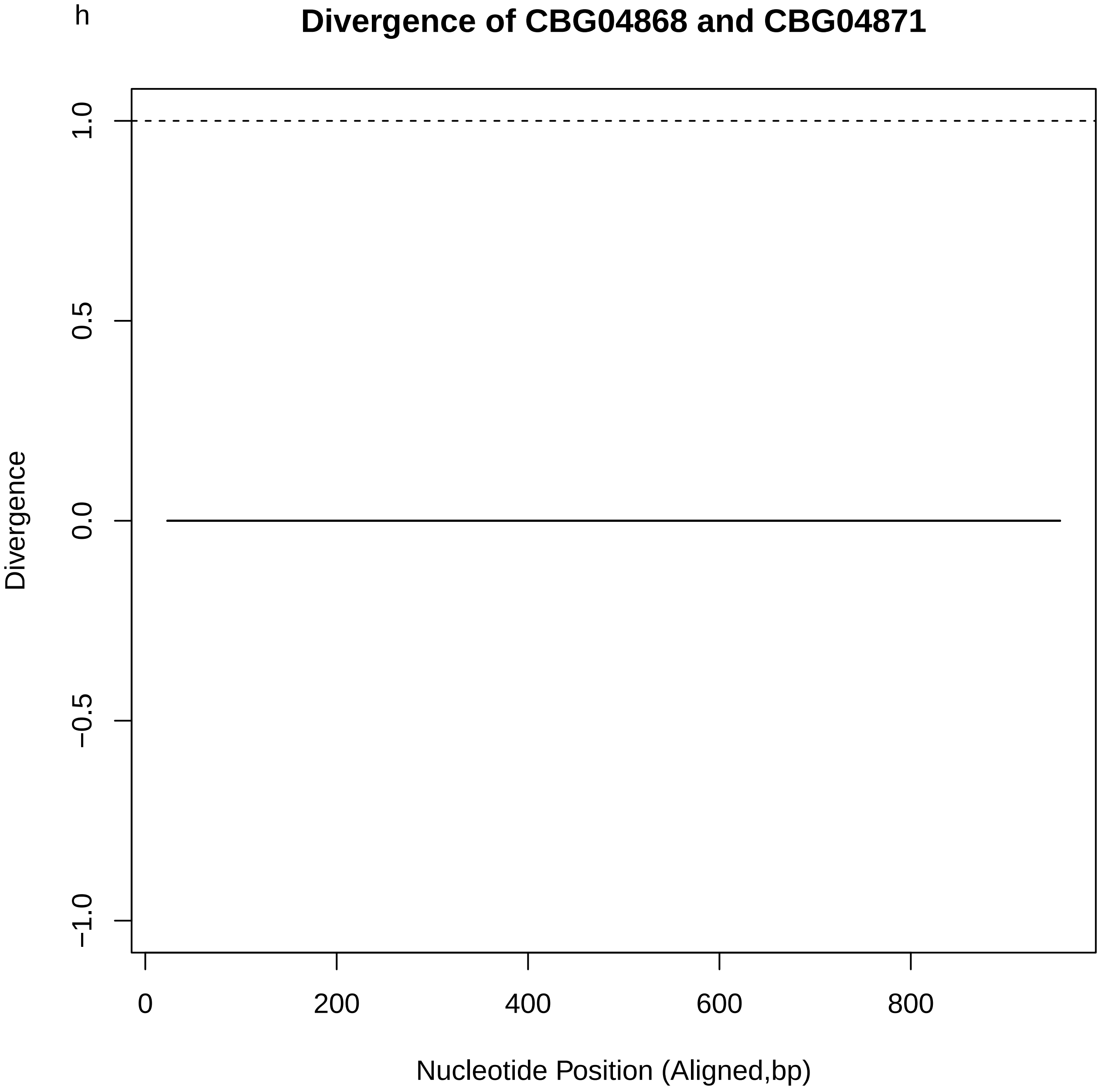
Divergence of CBG04868 and CBG04871
1.0
0.5
0.0
−0.5
−1.0
Divergence
0
200
400
600
800
Nucleotide Position (Aligned,bp)

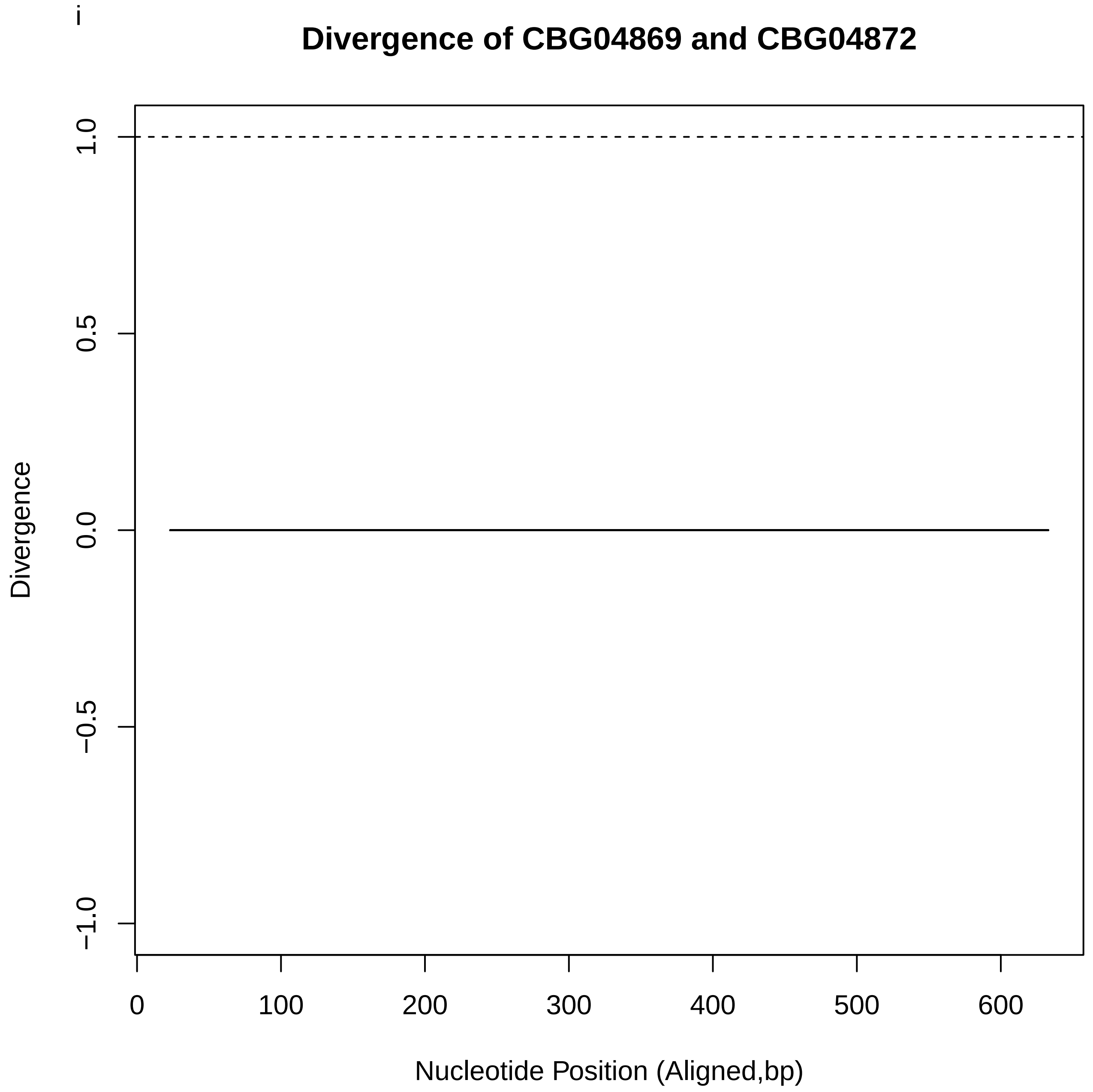
i
Divergence of CBG04869 and CBG04872
Divergence
1.0
0.5
0.0
−0.5
−1.0
0
100
200
300
400
500
600
Nucleotide Position (Aligned,bp)

j

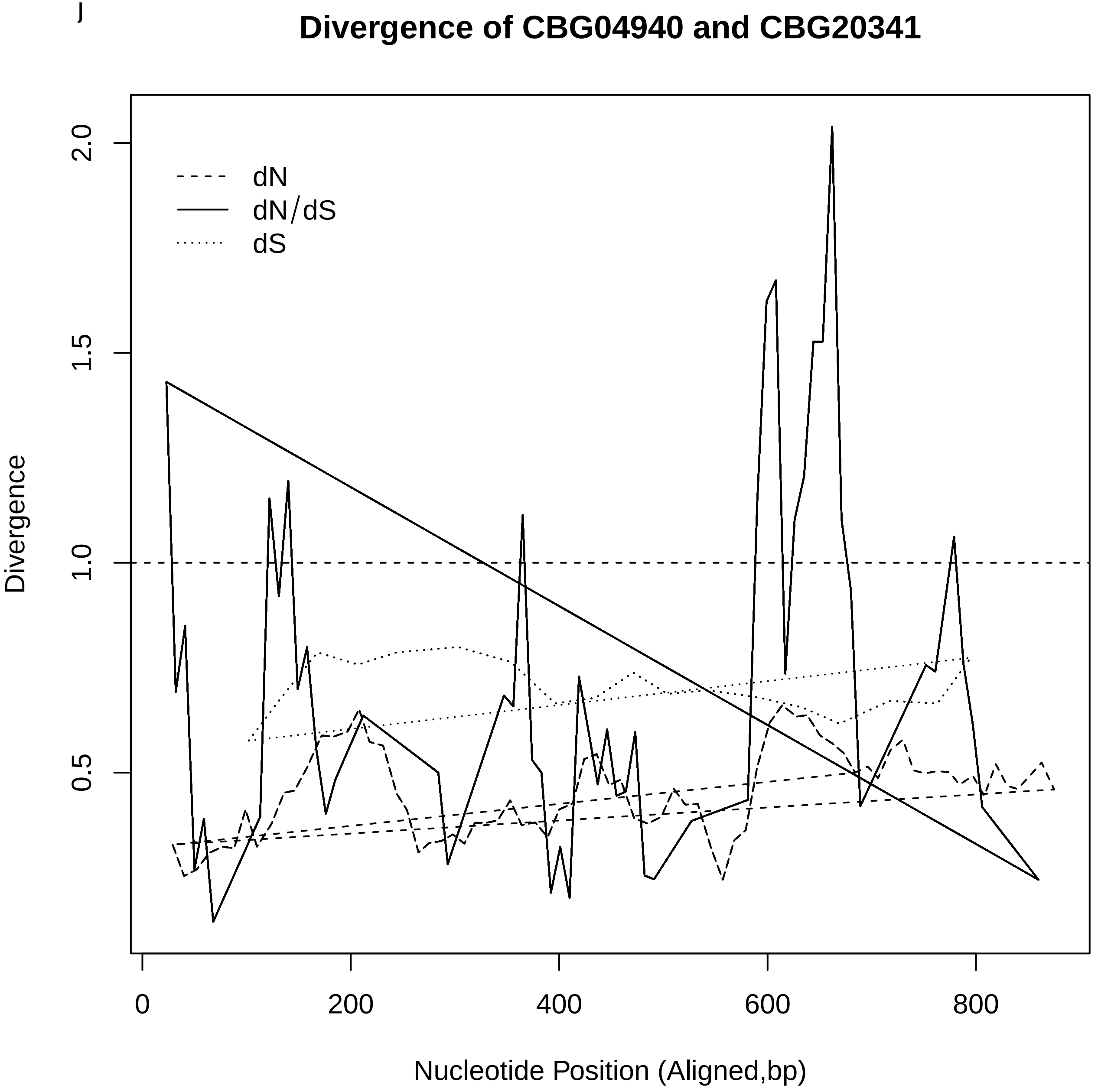
Divergence of CBG04940 and CBG20341
dN
dN/dS
dS
Divergence
2.0
1.5
1.0
0.5
0
200
400
600
800
Nucleotide Position (Aligned,bp)

k

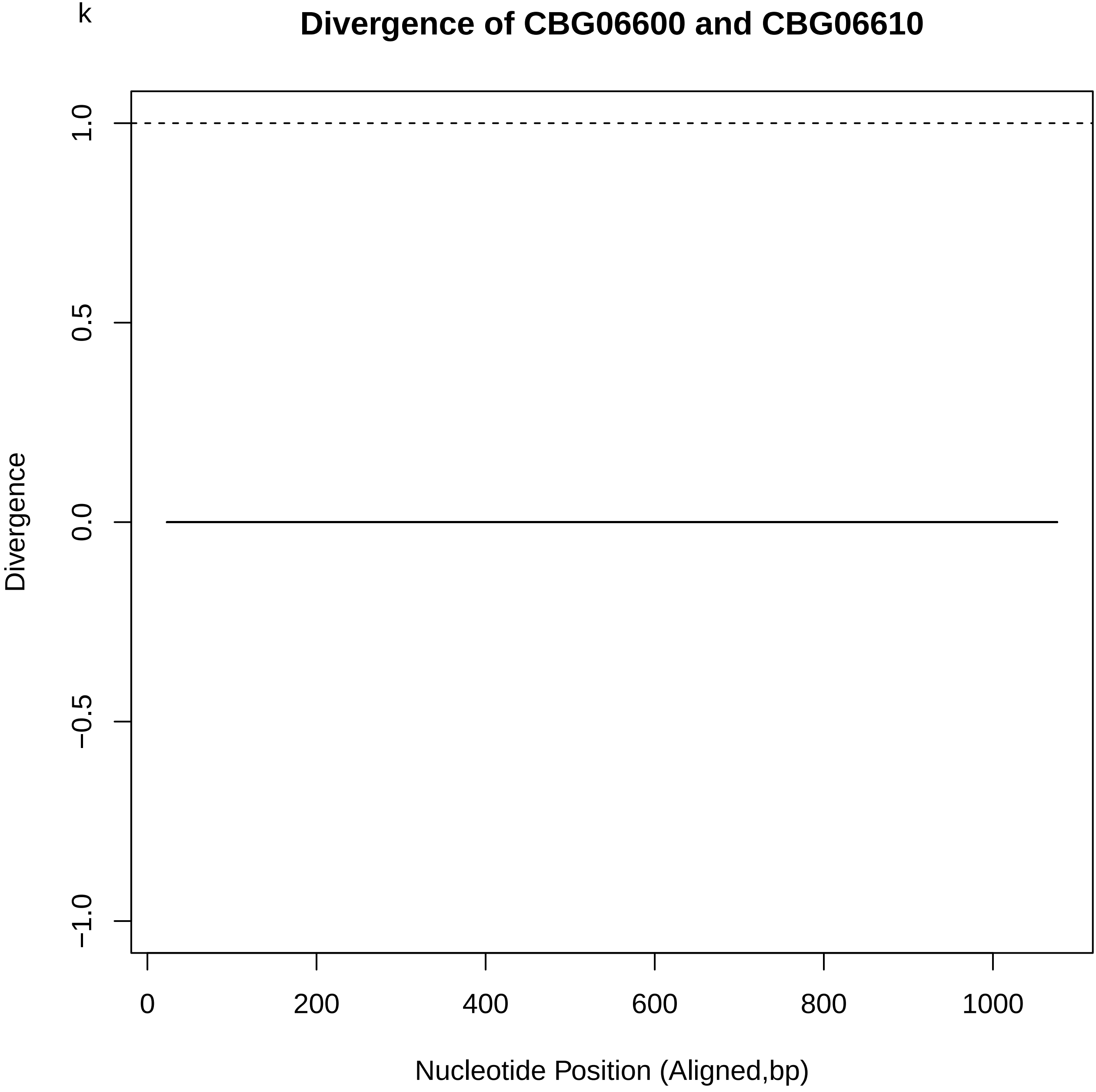

Divergence of CBG06600 and CBG06610
Divergence
1.0
0.5
0.0
−0.5
−1.0
0
200
400
600
800
1000
Nucleotide Position (Aligned,bp)

I

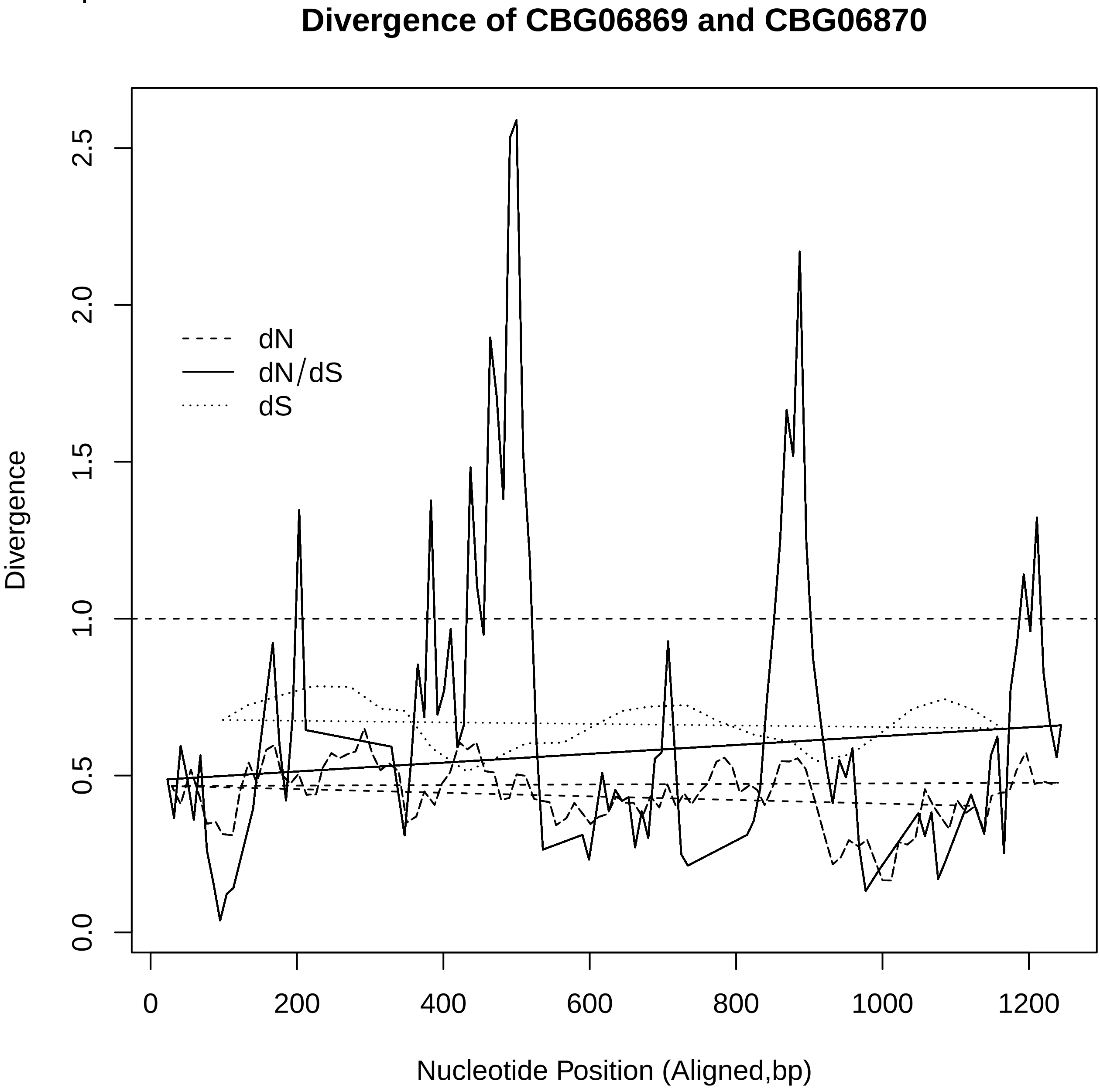
Divergence of CBG06869 and CBG06870
dN
dN/dS
dS
Divergence
Nucleotide Position (Aligned,bp)
0.0
0.5
1.0
1.5
2.0
2.5
0
200
400
600
800
1000
1200

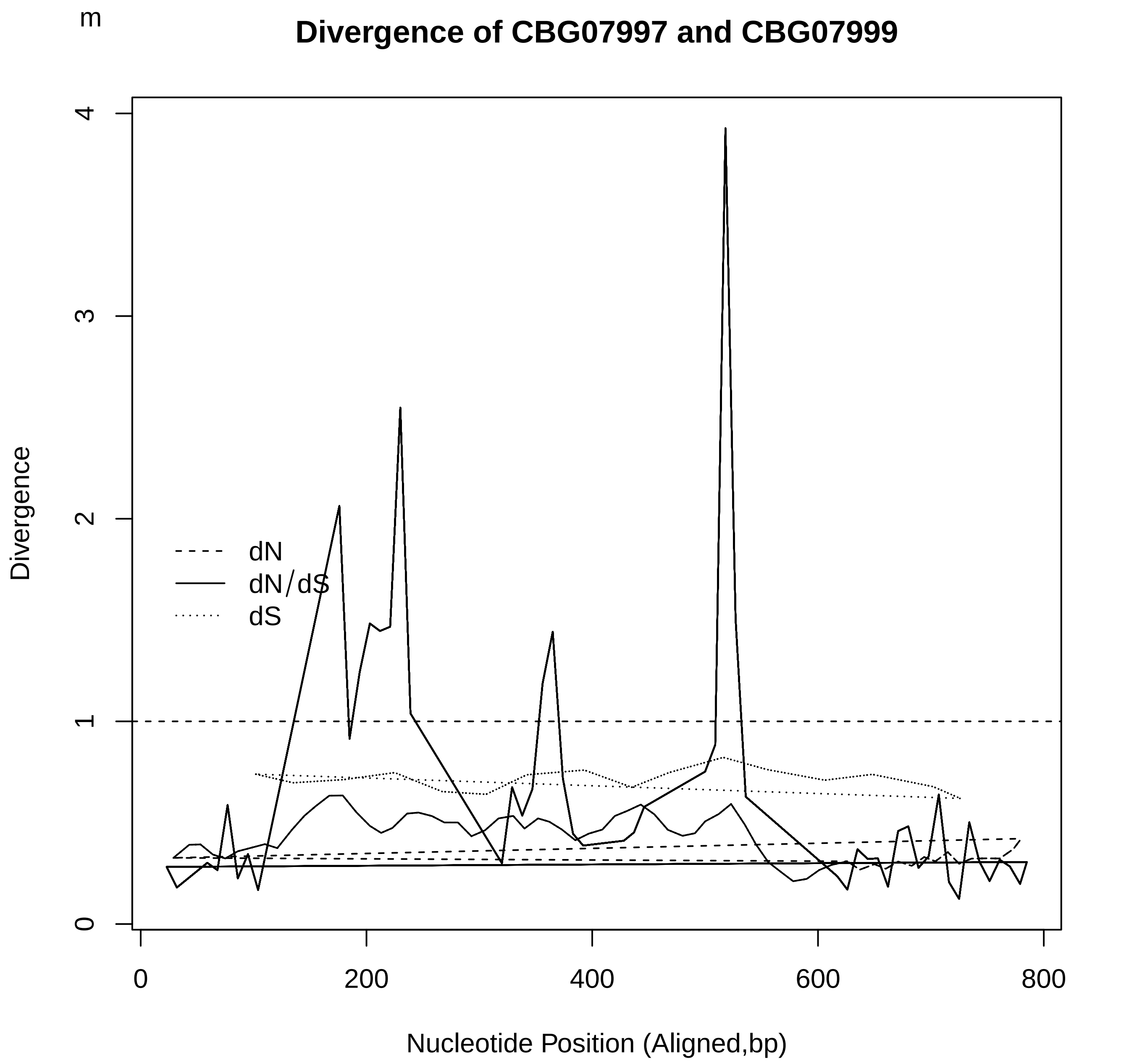
m
Divergence of CBG07997 and CBG07999
Divergence
Nucleotide Position (Aligned,bp)
dN
dN/dS
dS
0
1
2
3
4
0
200
400
600
800

n

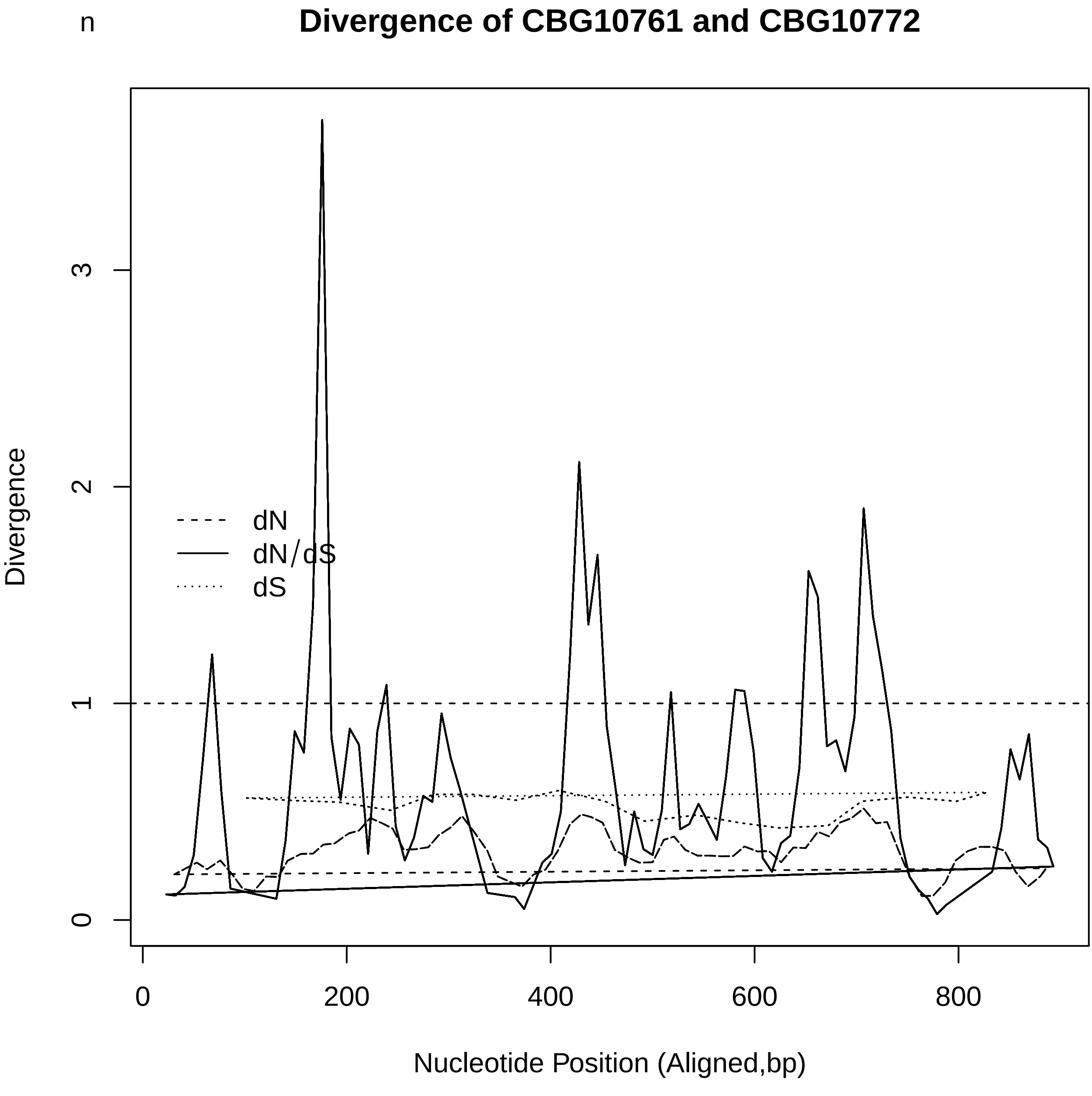

Divergence of CBG10761 and CBG10772
Divergence
0
1
2
3
dN
dN/dS
dS
0
200
400
600
800
Nucleotide Position (Aligned,bp)

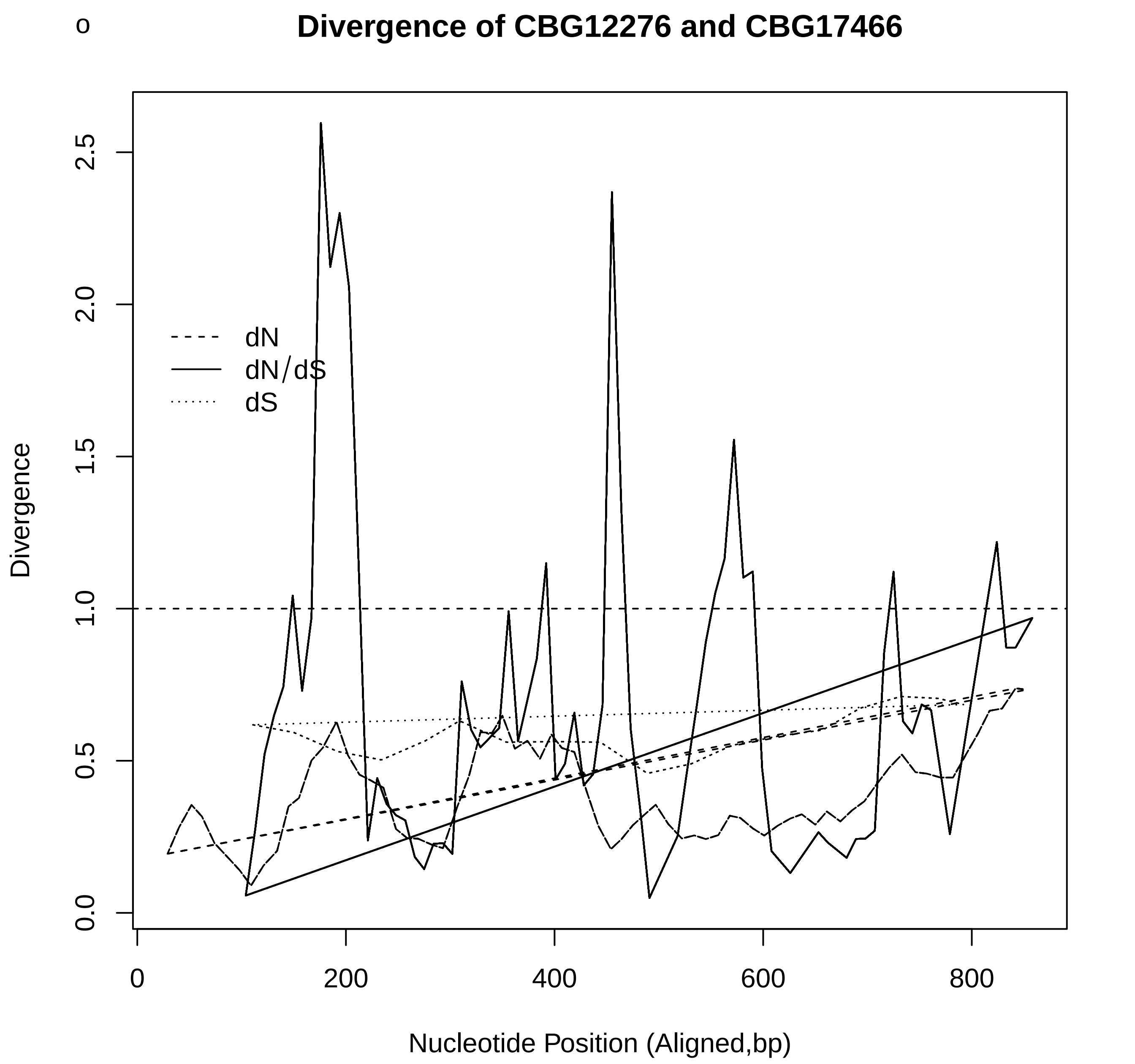

o
Divergence of CBG12276 and CBG17466
dN
dN/dS
dS
Divergence
0.0
0.5
1.0
1.5
2.0
2.5
0
200
400
600
800
Nucleotide Position (Aligned,bp)

p

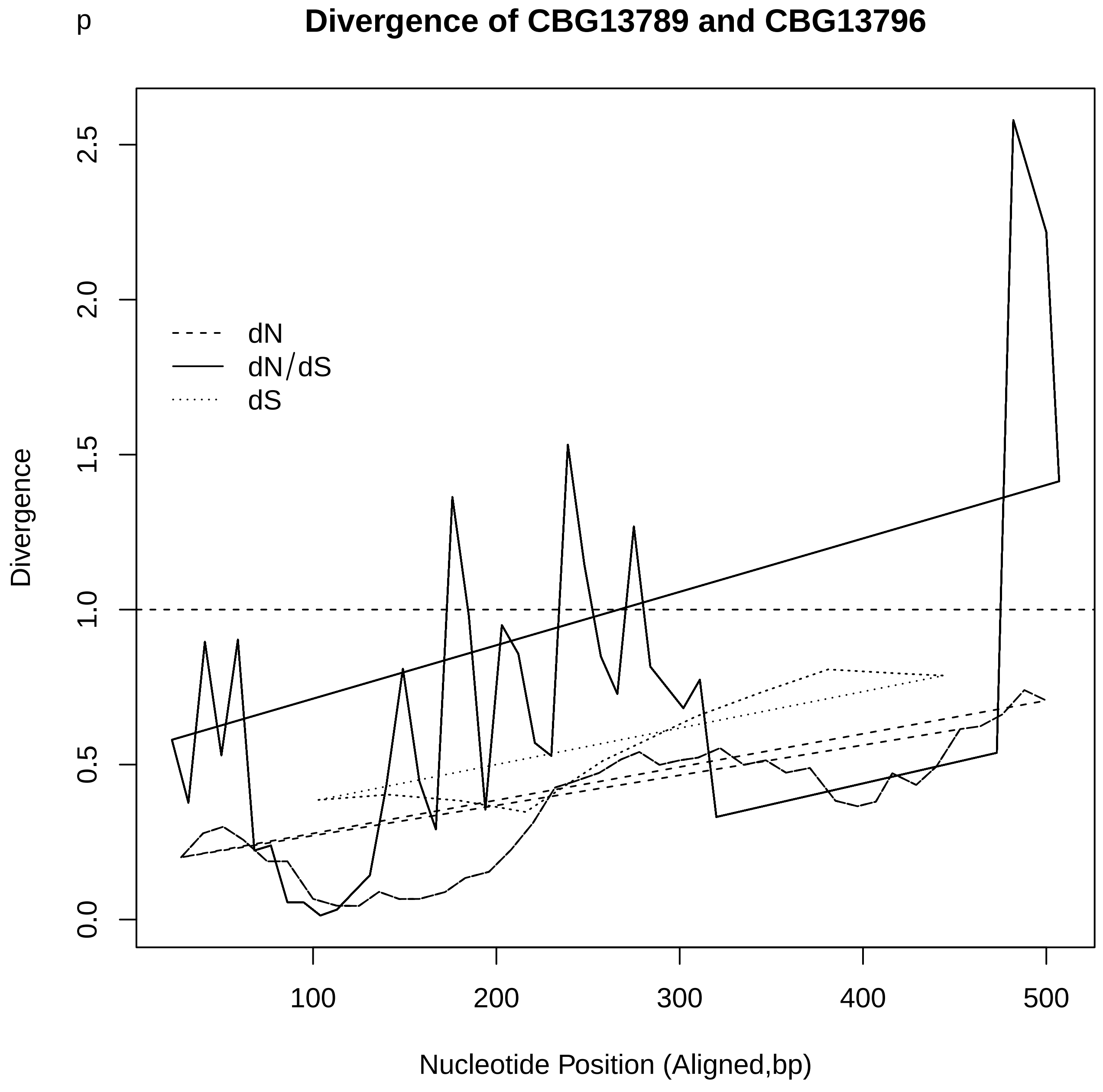
Divergence of CBG13789 and CBG13796
dN
dN/dS
dS
Divergence
0.0
0.5
1.0
1.5
2.0
2.5
100
200
300
400
500
Nucleotide Position (Aligned,bp)

q

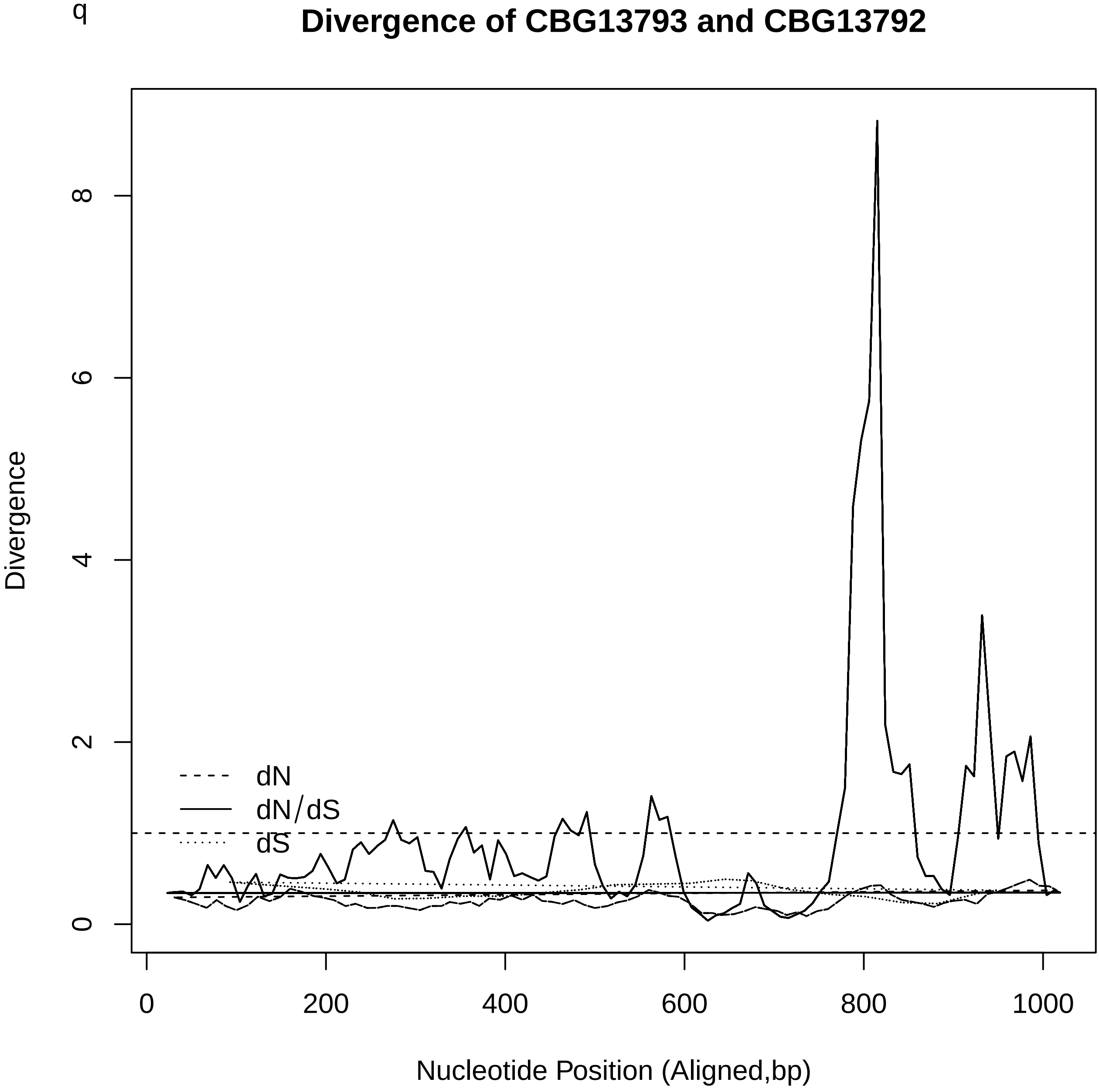
Divergence of CBG13793 and CBG13792
Divergence
Nucleotide Position (Aligned,bp)
dN
dN/dS
dS
0
2
4
6
8
0
200
400
600
800
1000

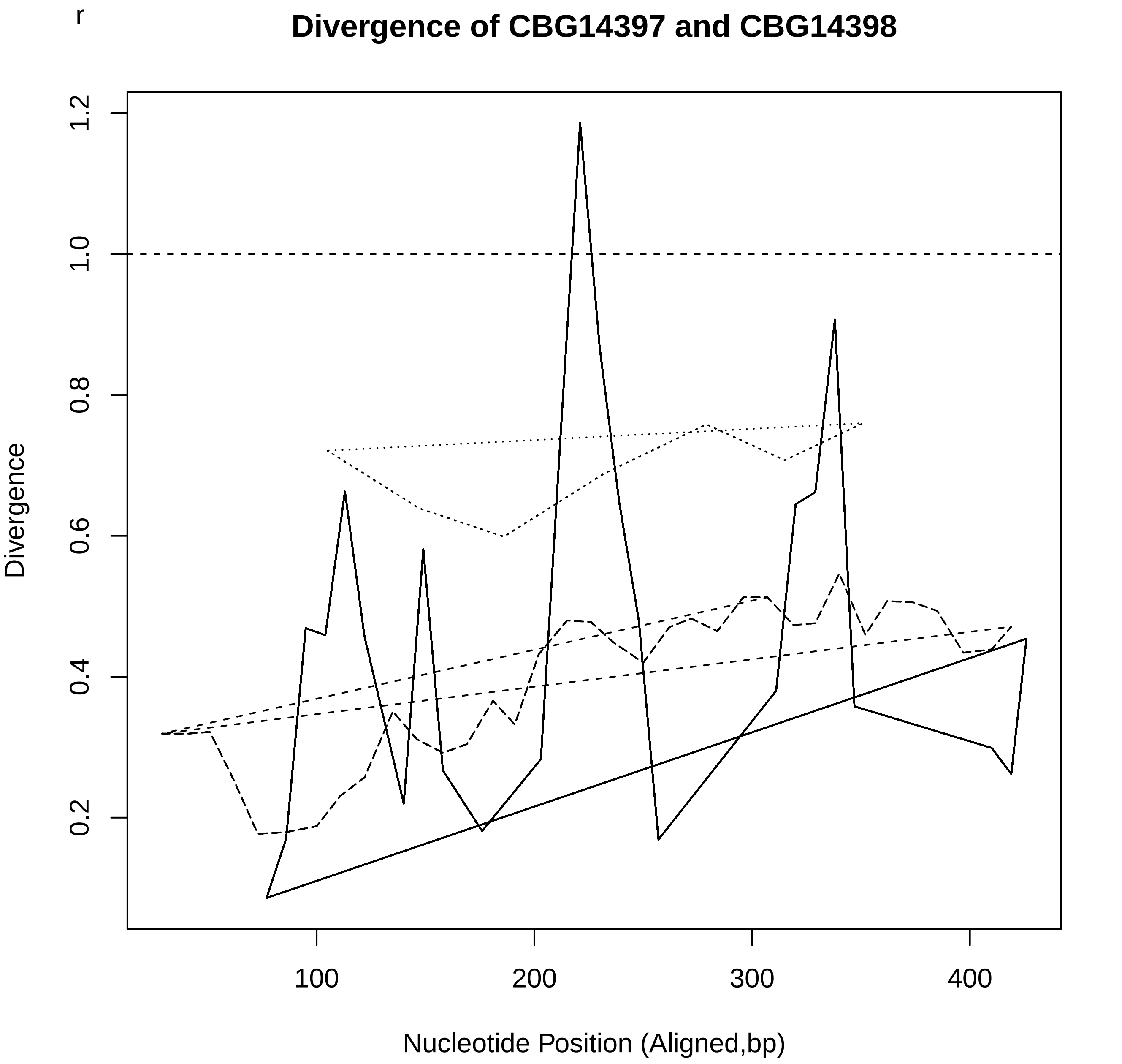
r
Divergence of CBG14397 and CBG14398
Divergence
0.2
0.4
0.6
0.8
1.0
1.2
100
200
300
400
Nucleotide Position (Aligned,bp)

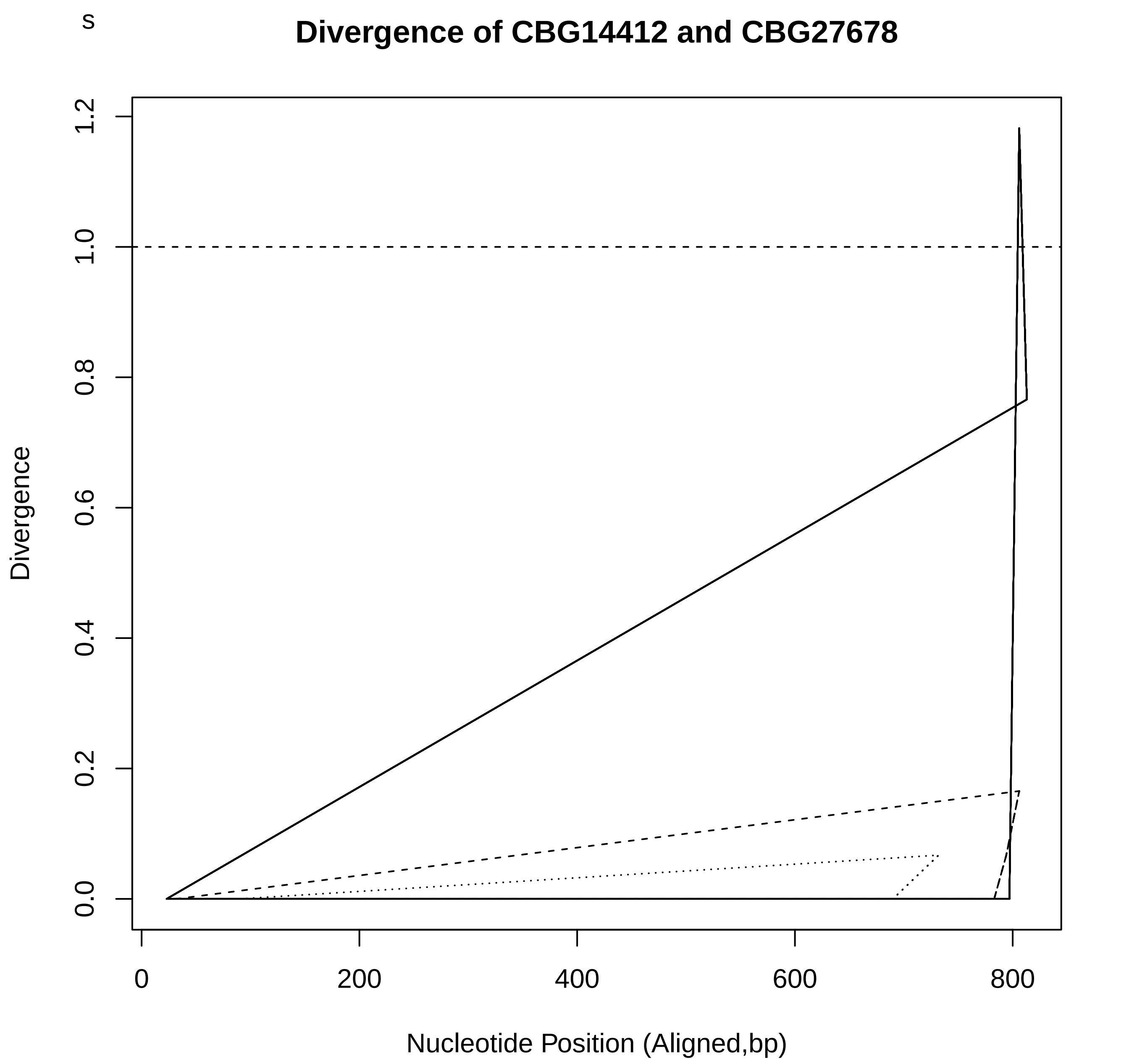
s
Divergence of CBG14412 and CBG27678
Divergence
0.0
0.2
0.4
0.6
0.8
1.0
1.2
0
200
400
600
800
Nucleotide Position (Aligned,bp)

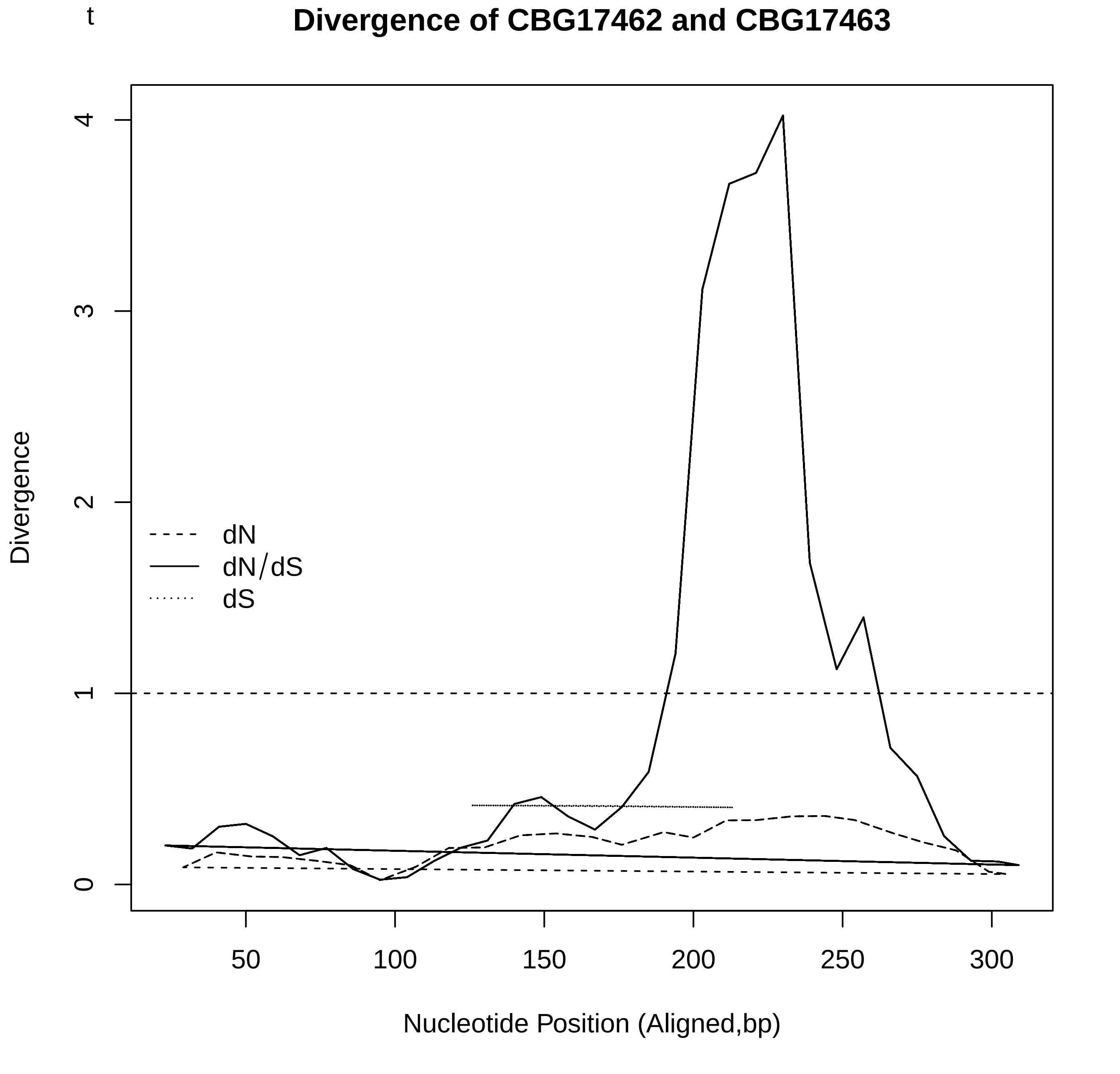
t
Divergence of CBG17462 and CBG17463
Divergence
Nucleotide Position (Aligned,bp)
dN
dN/dS
dS
0
1
2
3
4
50
100
150
200
250
300

u

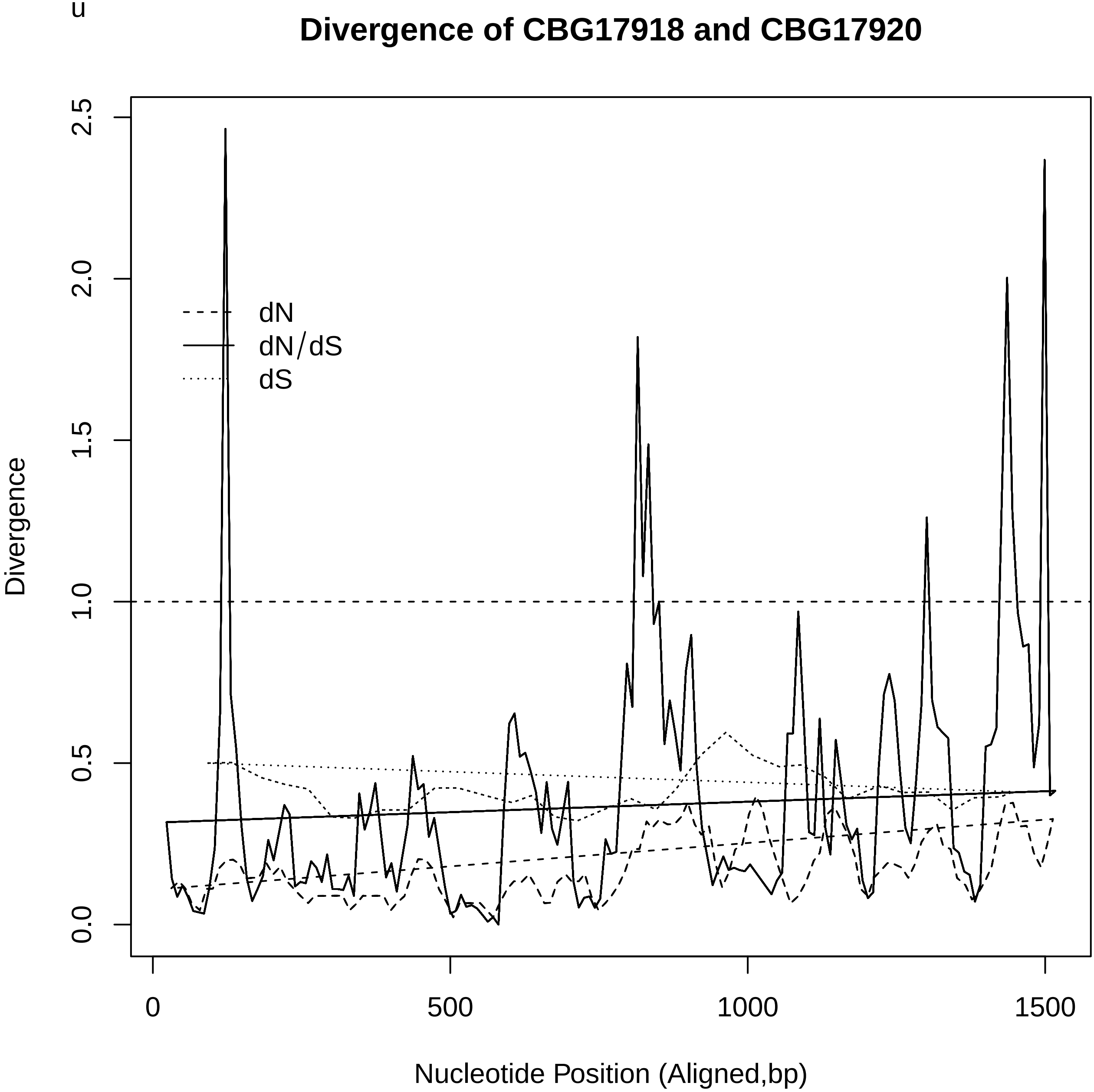
Divergence of CBG17918 and CBG17920
Divergence
0.0
0.5
1.0
1.5
2.0
2.5
dN
dN/dS
dS
0
500
1000
1500
Nucleotide Position (Aligned,bp)

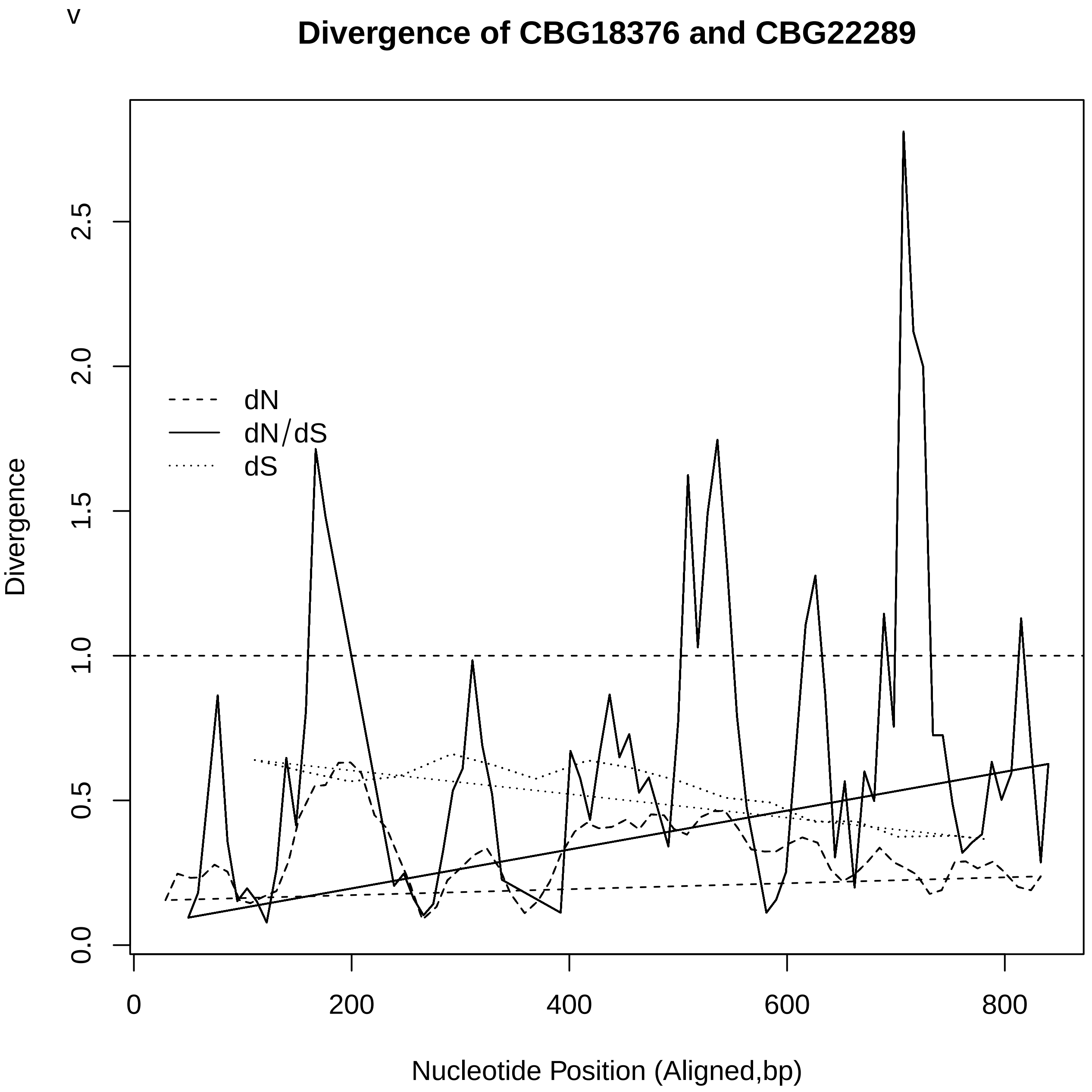
v
Divergence of CBG18376 and CBG22289
Divergence
Nucleotide Position (Aligned,bp)
dN
dN/dS
dS
0.0
0.5
1.0
1.5
2.0
2.5
0
200
400
600
800

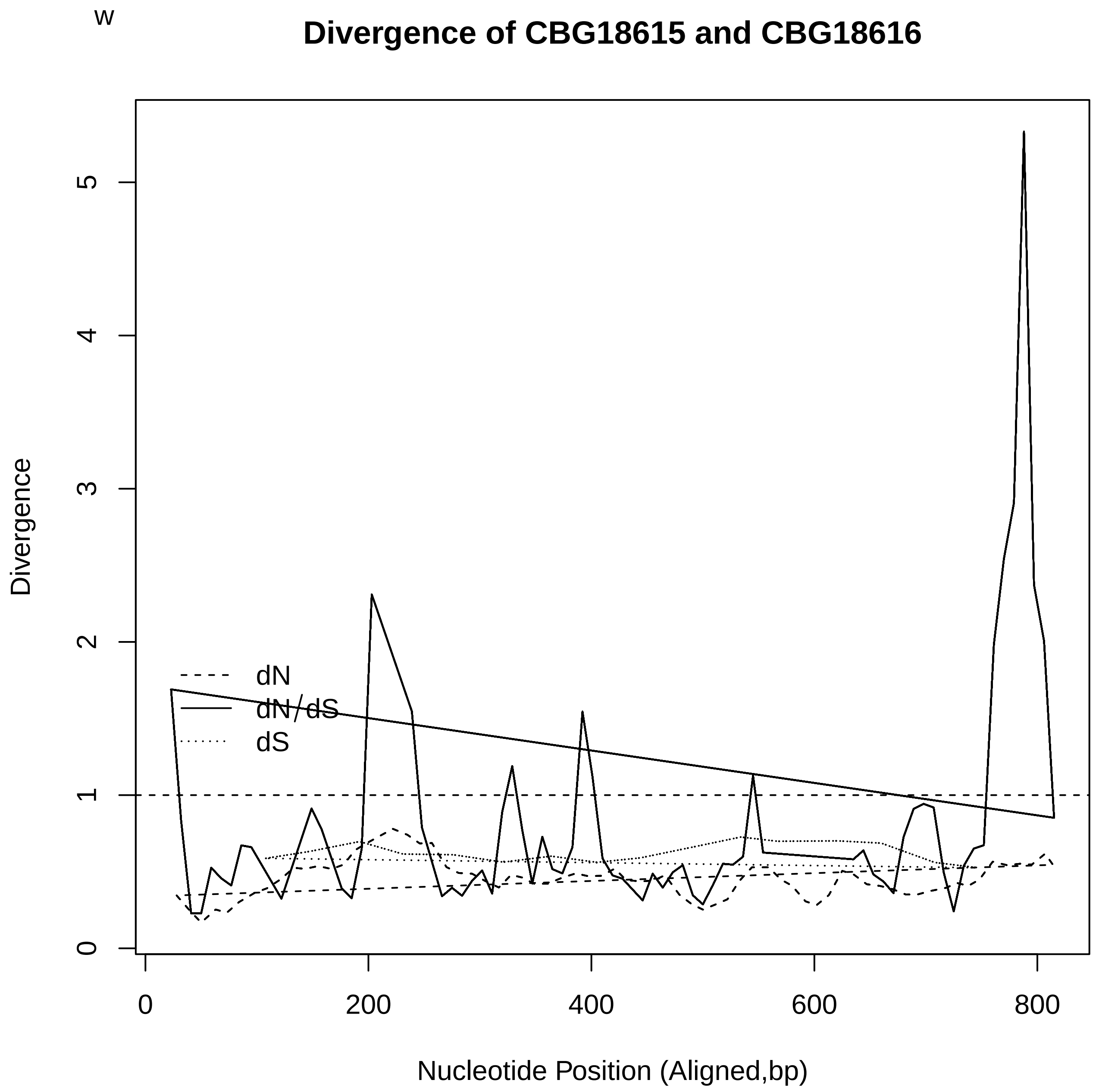

w
Divergence of CBG18615 and CBG18616
Divergence
0
1
2
3
4
5
dN
dN/dS
dS
0
200
400
600
800
Nucleotide Position (Aligned,bp)

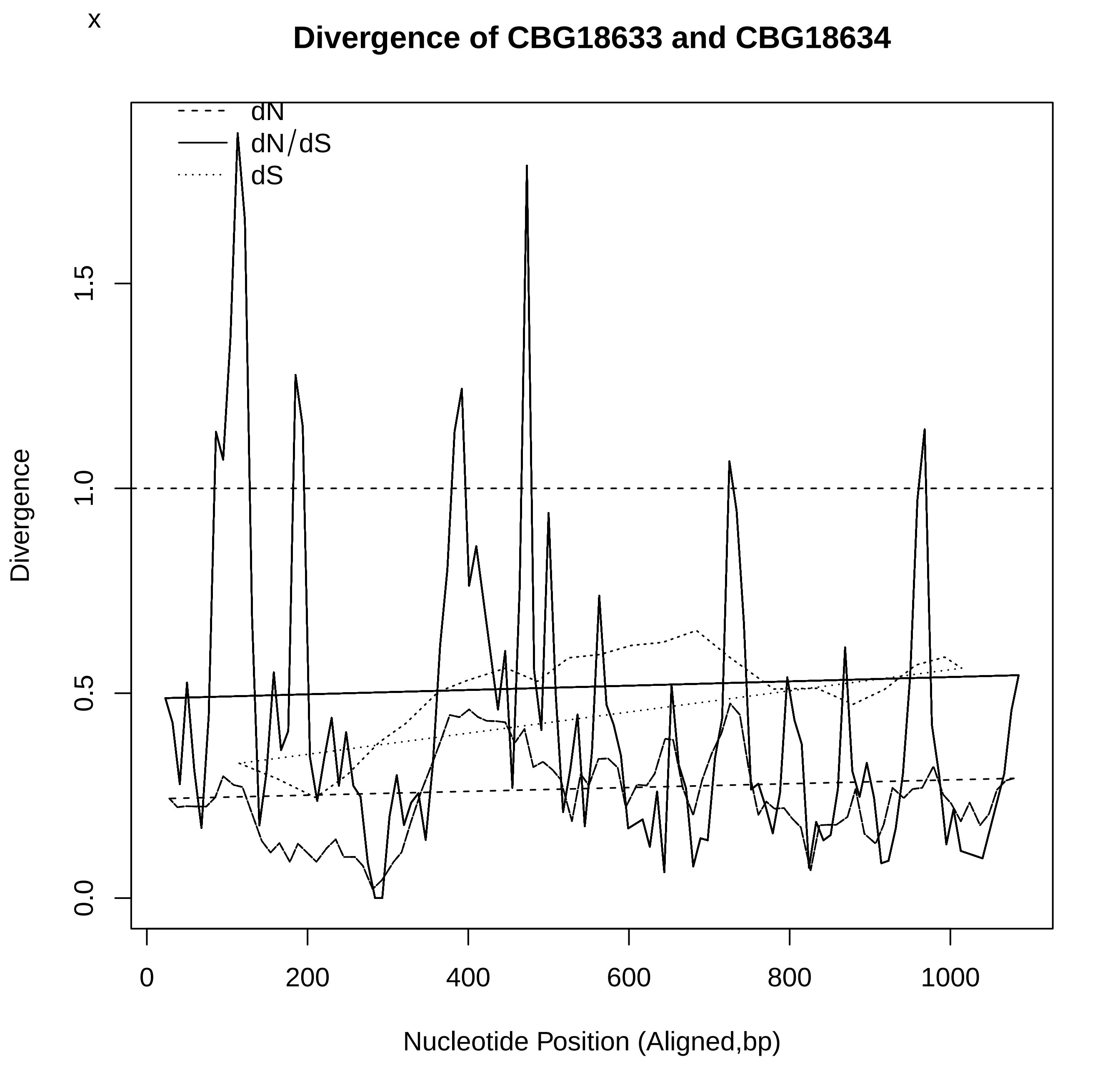
x
Divergence of CBG18633 and CBG18634
dN
dN/dS
dS
Divergence
0.0
0.5
1.0
1.5
0
200
400
600
800
1000
Nucleotide Position (Aligned,bp)

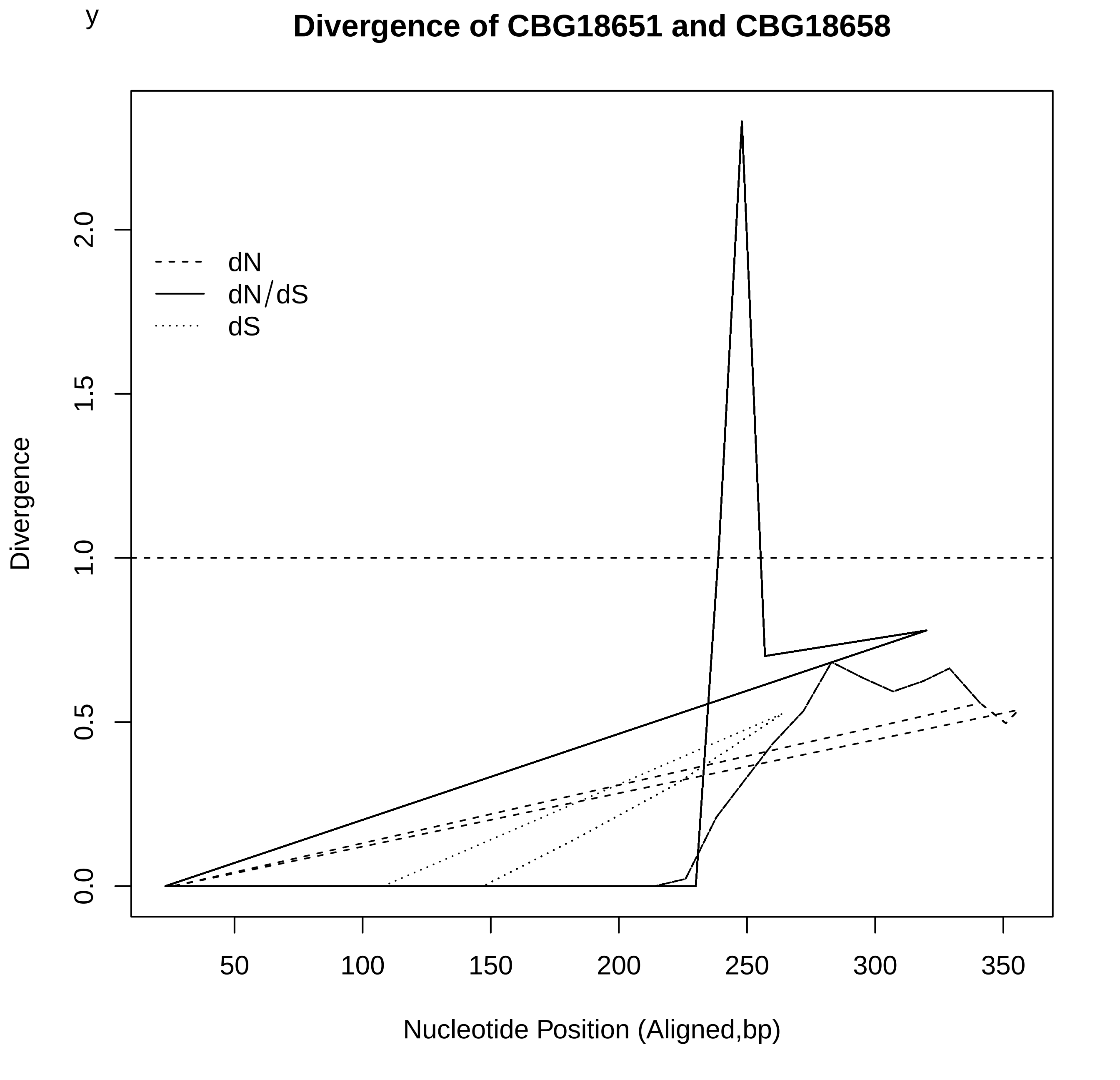
y
Divergence of CBG18651 and CBG18658
Divergence
Nucleotide Position (Aligned,bp)
dN
dN/dS
dS
0.0
0.5
1.0
1.5
2.0
50
100
150
200
250
300
350

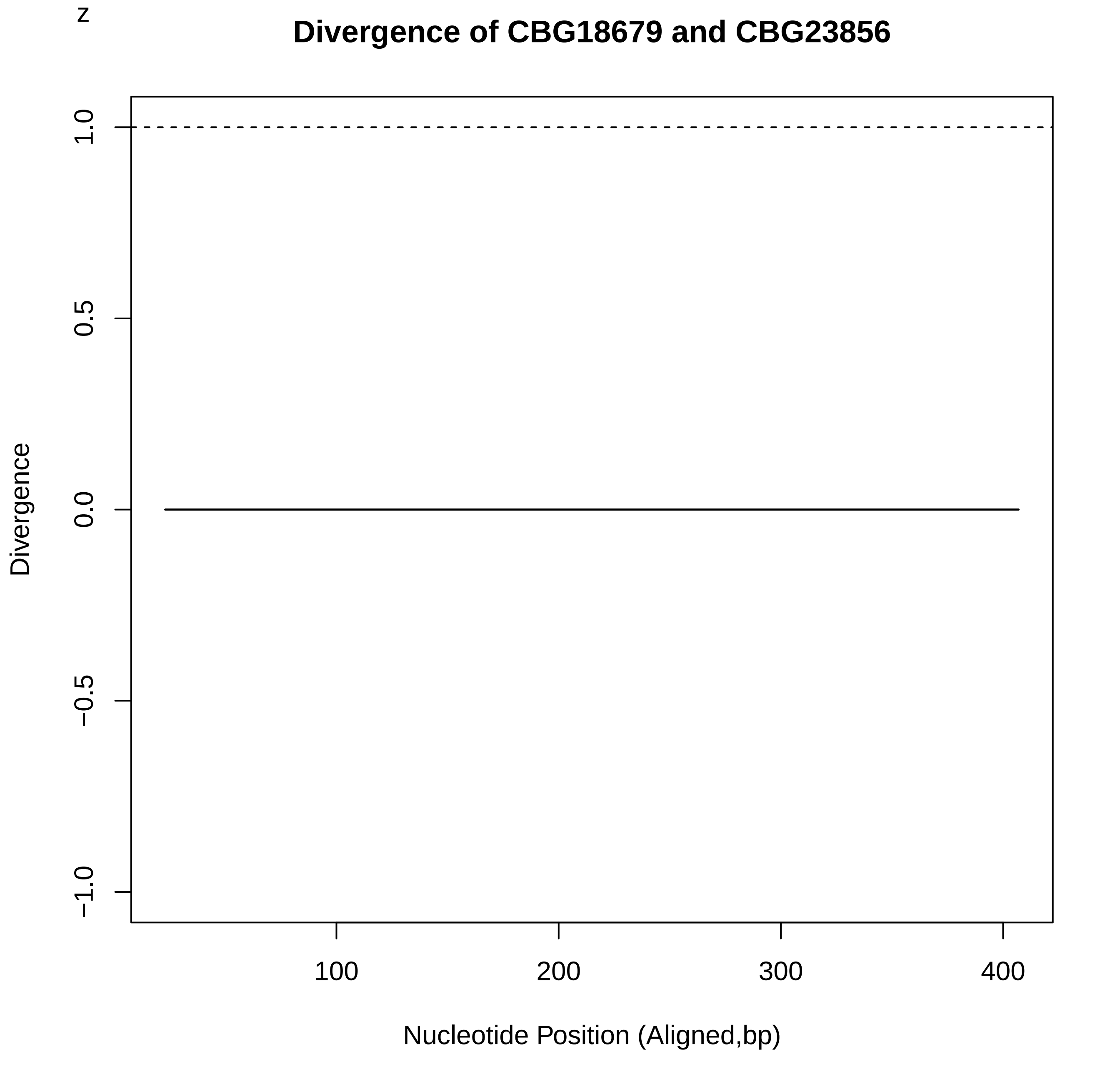

z
Divergence of CBG18679 and CBG23856
Divergence
1.0
0.5
0.0
−0.5
−1.0
100
200
300
400
Nucleotide Position (Aligned,bp)

aa

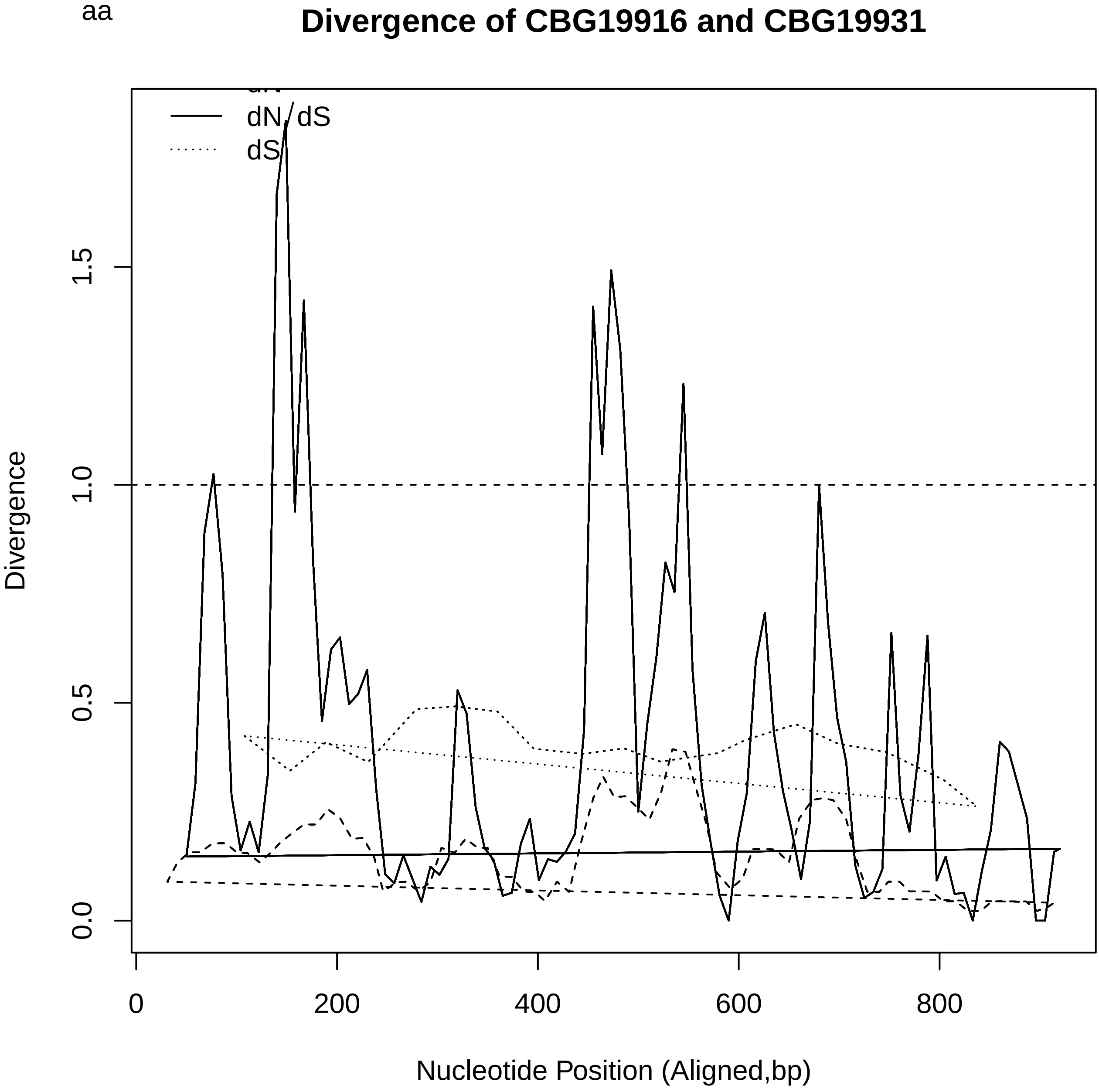

Divergence of CBG19916 and CBG19931
dN
dN/dS
dS
Divergence
0.0
0.5
1.0
1.5
0
200
400
600
800
Nucleotide Position (Aligned,bp)

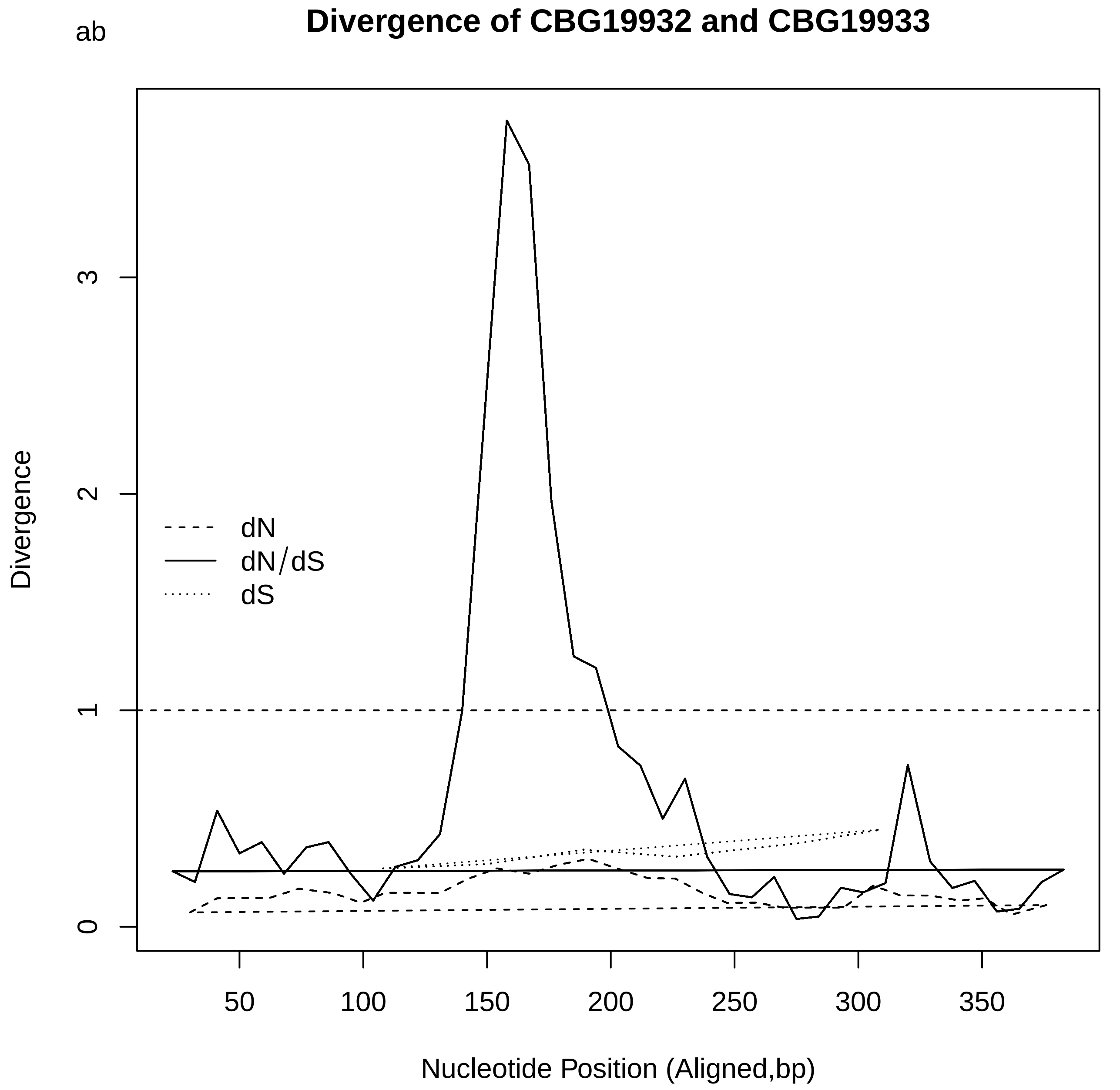

ab
Divergence of CBG19932 and CBG19933
Divergence
0
1
2
3
dN
dN/dS
dS
50
100
150
200
250
300
350
Nucleotide Position (Aligned,bp)

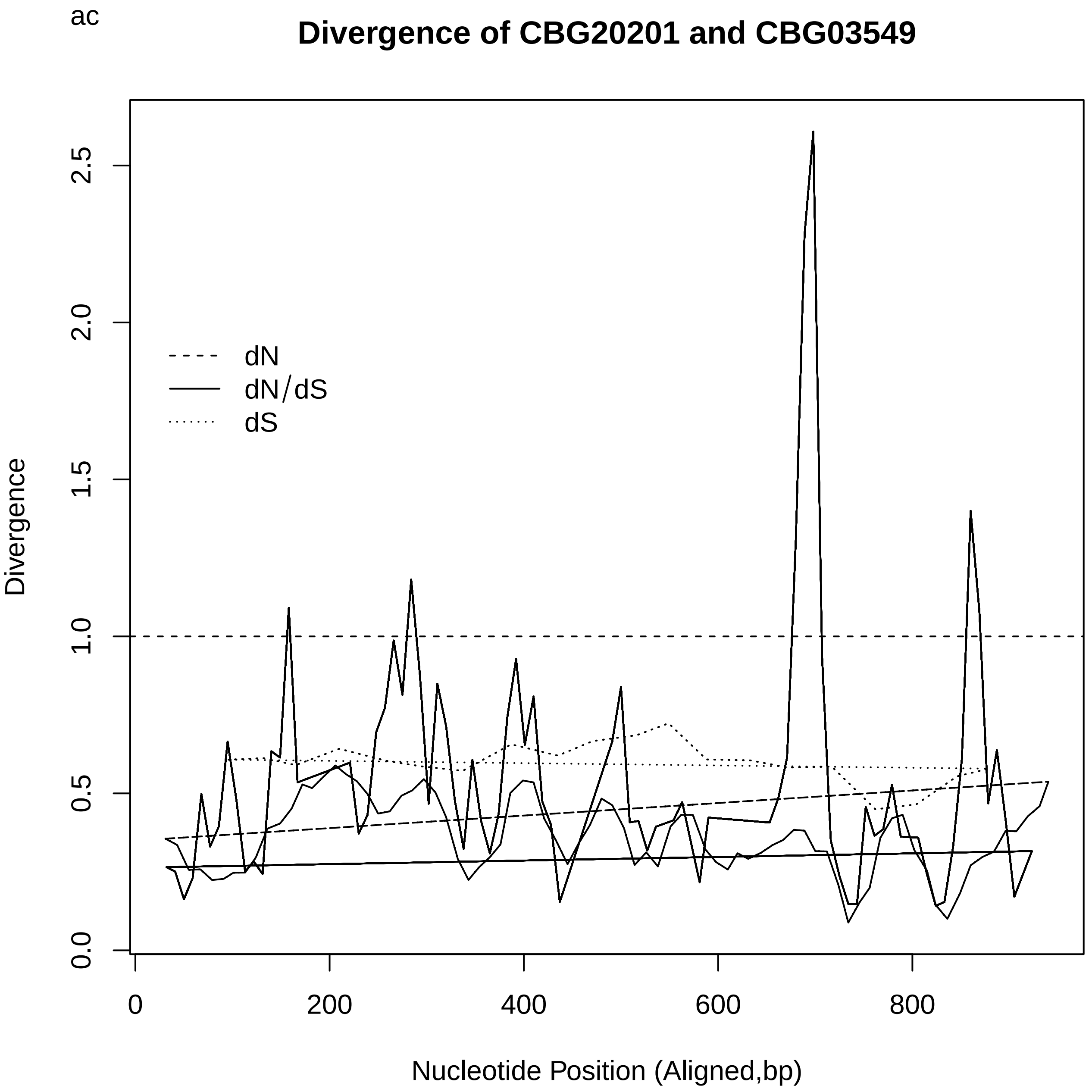
ac
Divergence of CBG20201 and CBG03549
dN
dN/dS
dS
Divergence
Nucleotide Position (Aligned,bp)
0.0
0.5
1.0
1.5
2.0
2.5
0
200
400
600
800

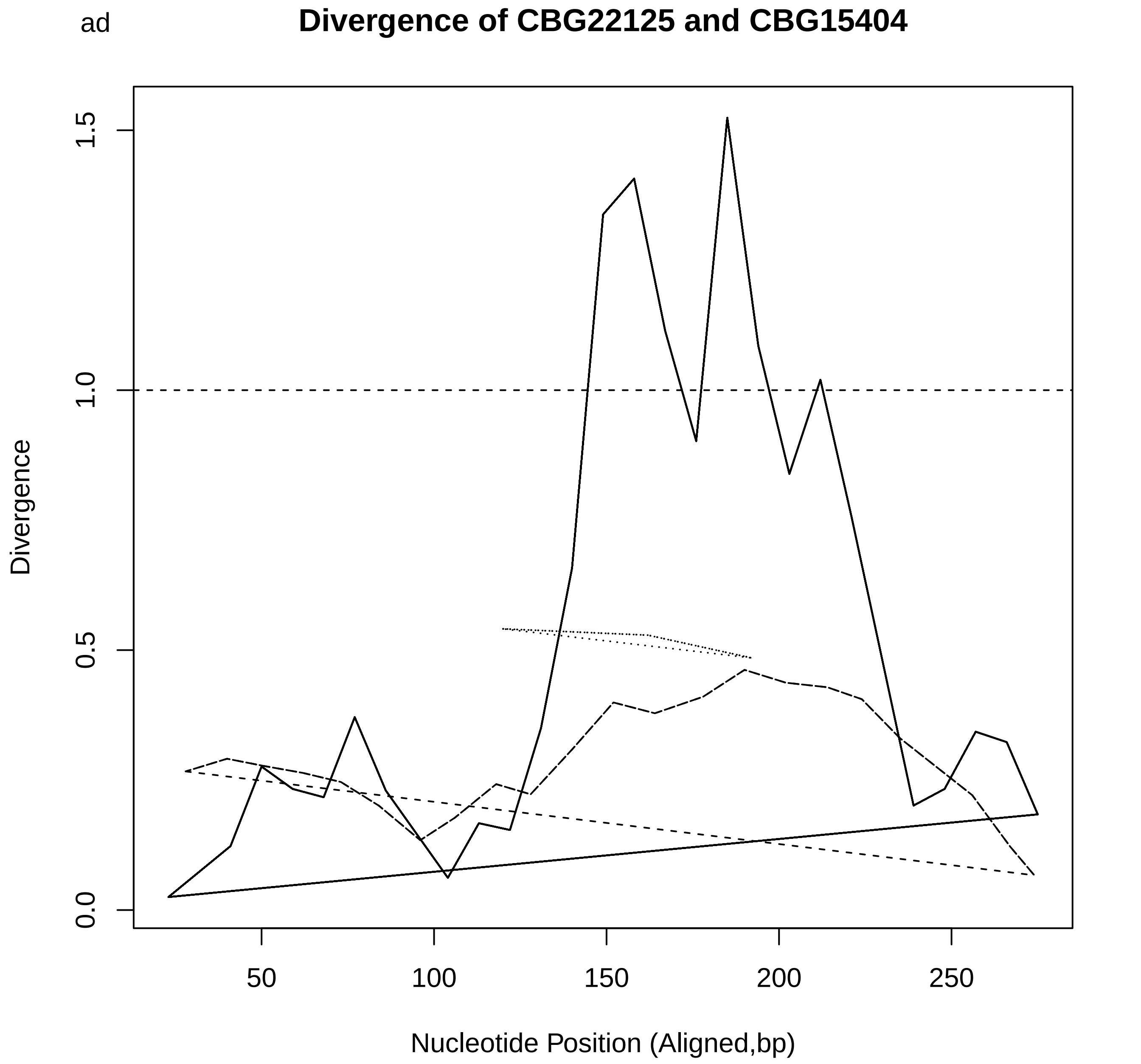
ad
Divergence of CBG22125 and CBG15404
Divergence
0.0
0.5
1.0
1.5
50
100
150
200
250
Nucleotide Position (Aligned,bp)

ae

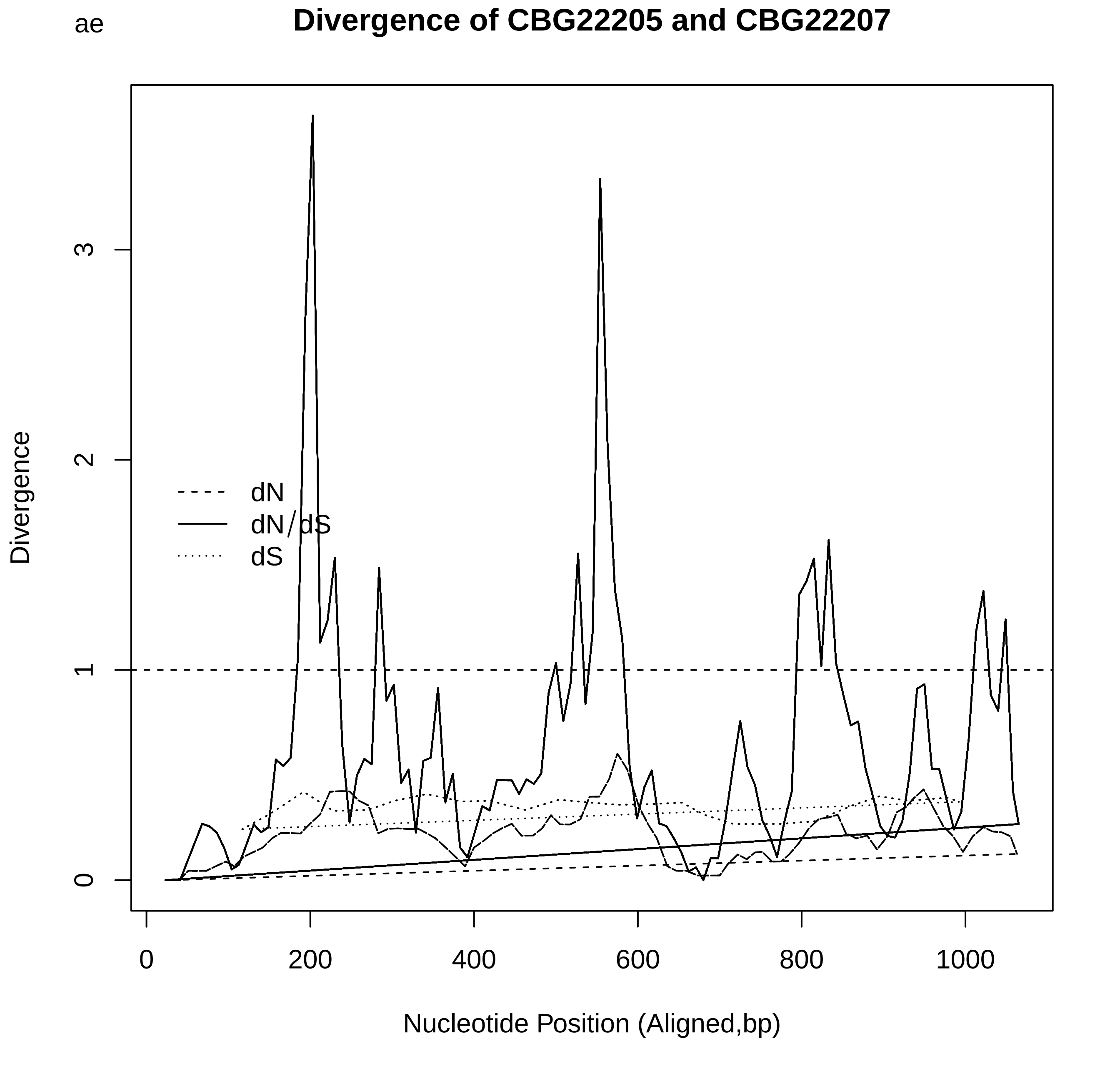

Divergence of CBG22205 and CBG22207
Divergence
0
1
2
3
dN
dN/dS
dS
0
200
400
600
800
1000
Nucleotide Position (Aligned,bp)

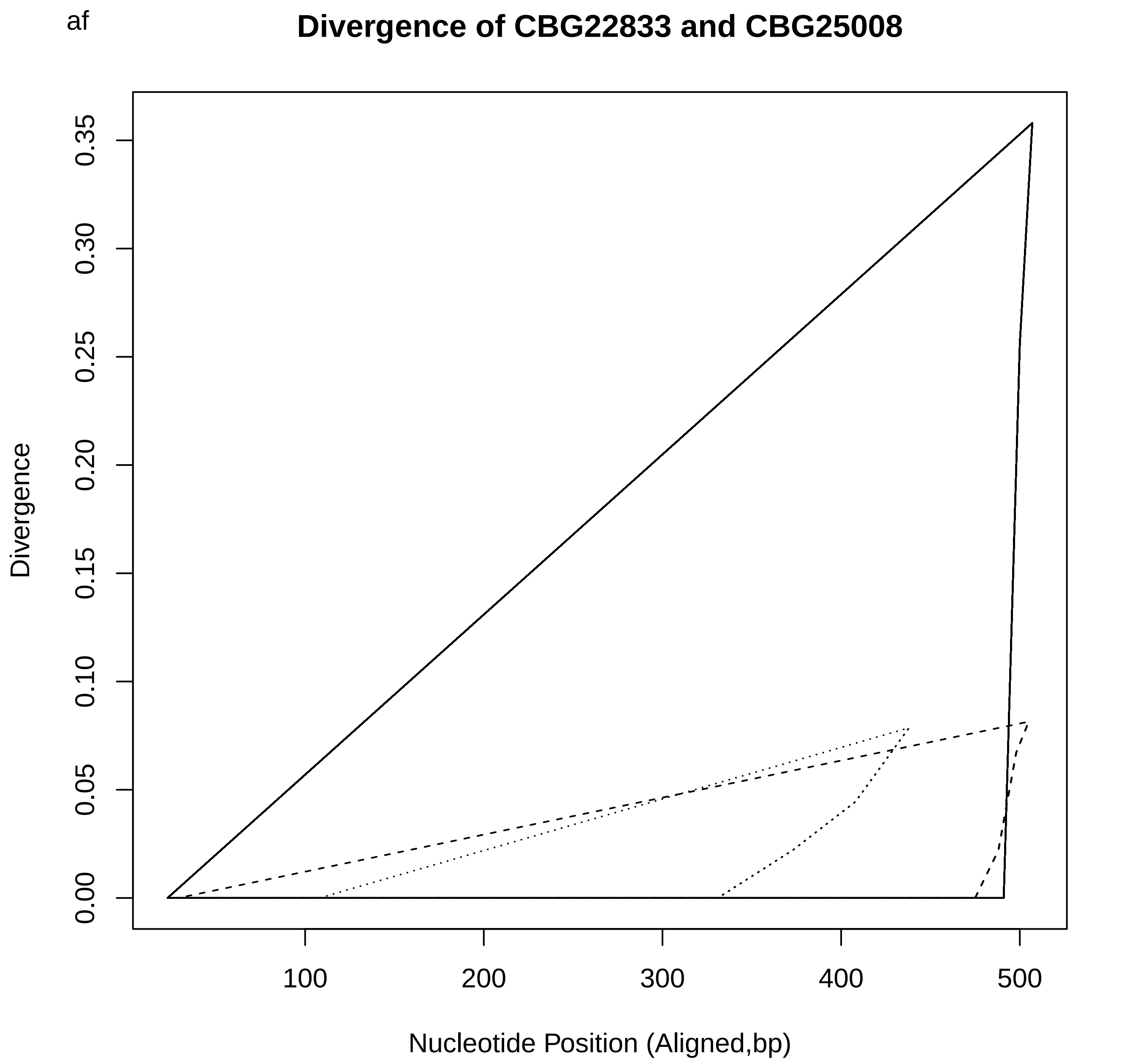
af
Divergence of CBG22833 and CBG25008
Divergence
0.00
0.05
0.10
0.15
0.20
0.25
0.30
0.35
100
200
300
400
500
Nucleotide Position (Aligned,bp)

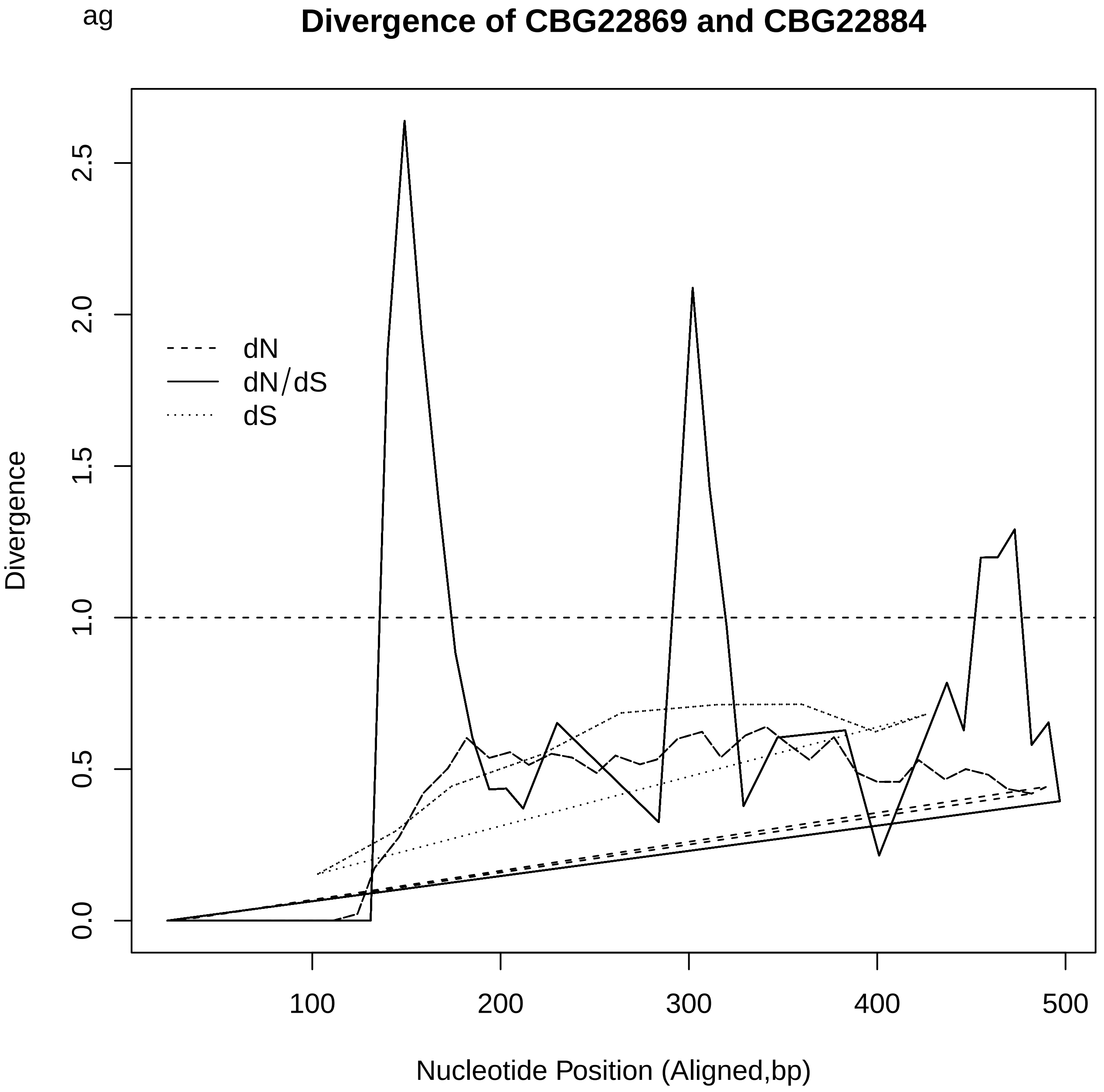
ag
Divergence of CBG22869 and CBG22884
Divergence
Nucleotide Position (Aligned,bp)
dN
dN/dS
dS
0.0
0.5
1.0
1.5
2.0
2.5
100
200
300
400
500

ah

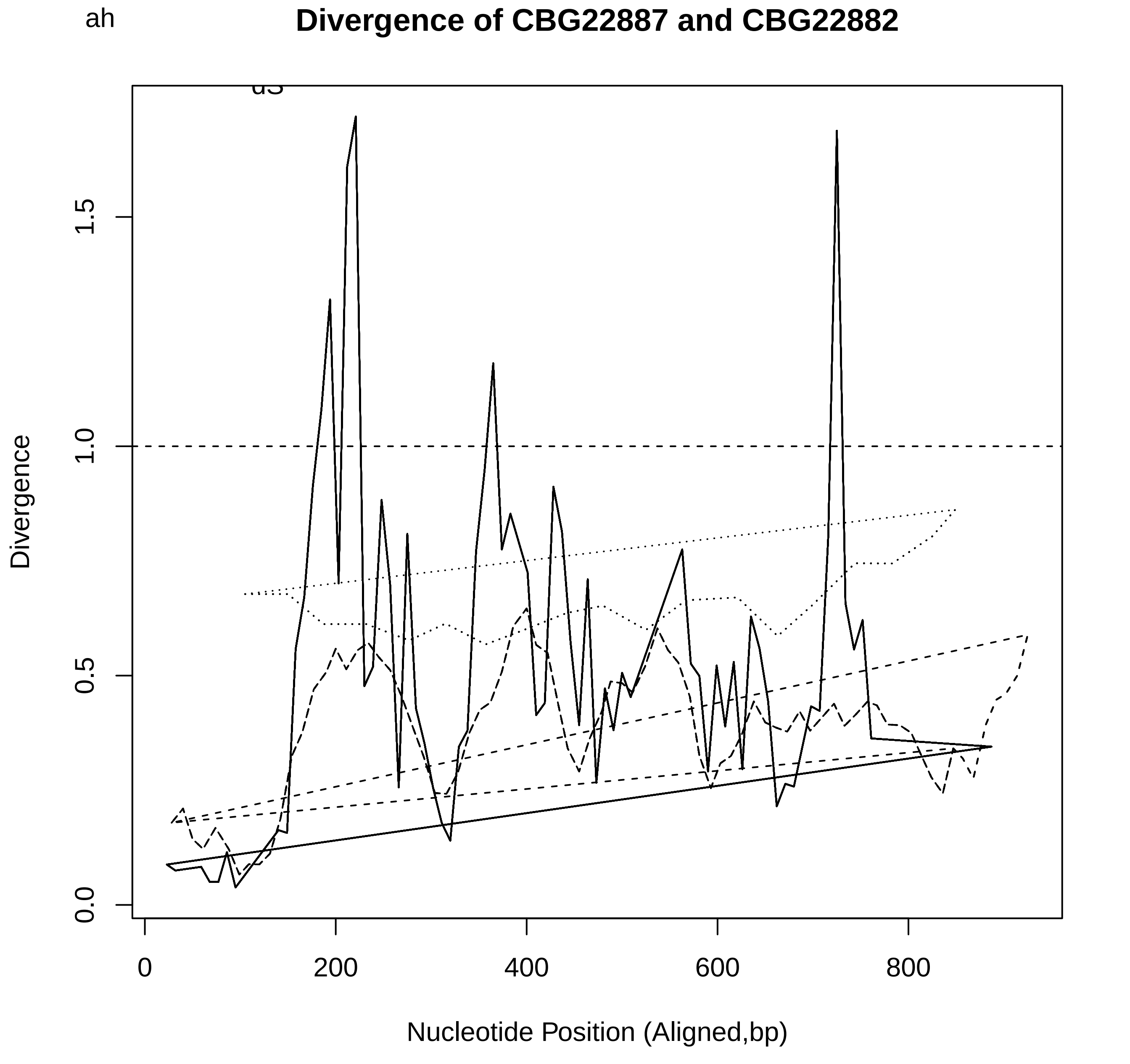

Divergence of CBG22887 and CBG22882
dS
Divergence
0.0
0.5
1.0
1.5
0
200
400
600
800
Nucleotide Position (Aligned,bp)

ai

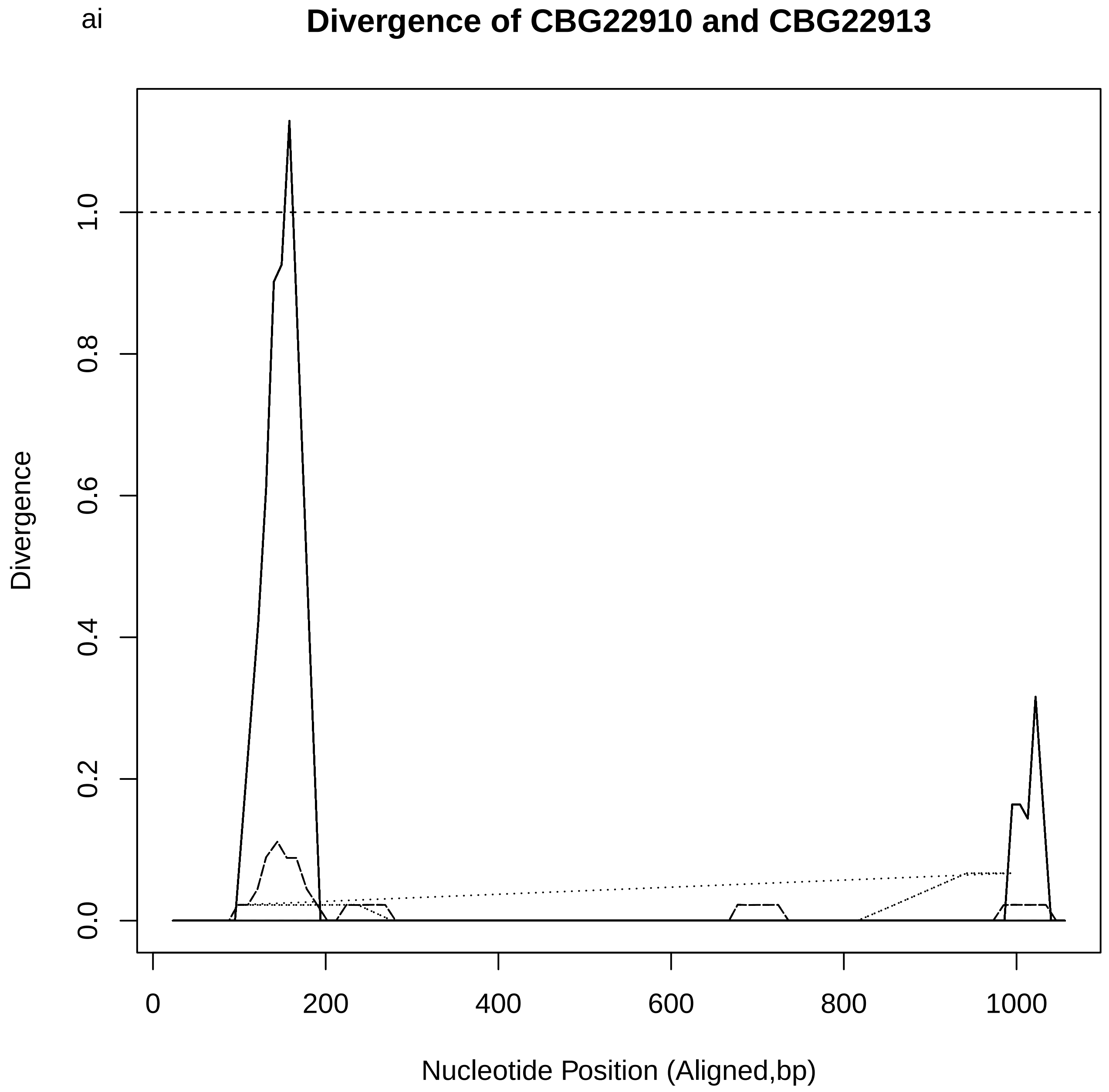

Divergence of CBG22910 and CBG22913
Divergence
0.0
0.2
0.4
0.6
0.8
1.0
0
200
400
600
800
1000
Nucleotide Position (Aligned,bp)

aj

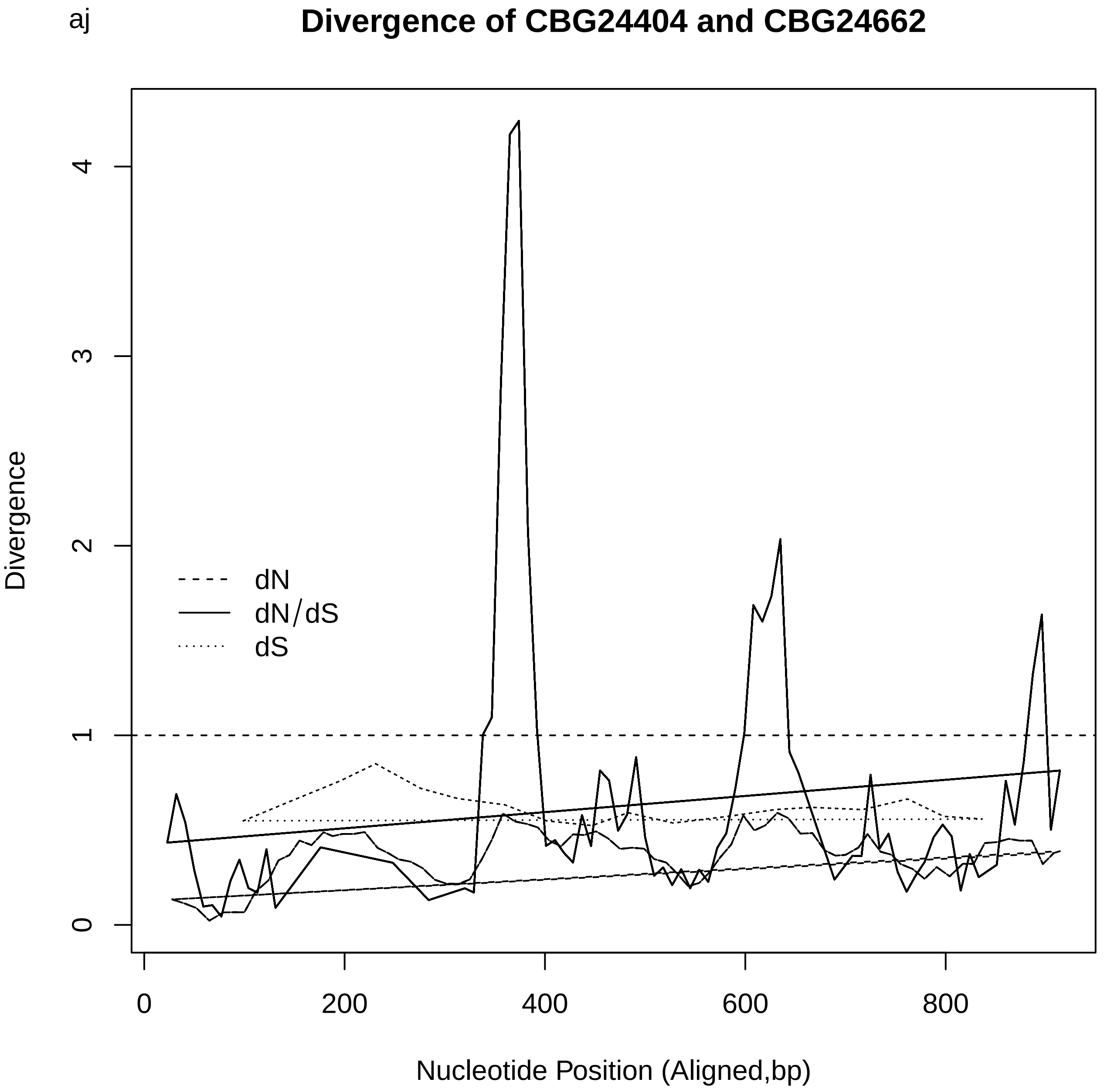

Divergence of CBG24404 and CBG24662
Divergence
0
1
2
3
4
dN
dN/dS
dS
0
200
400
600
800
Nucleotide Position (Aligned,bp)

ak

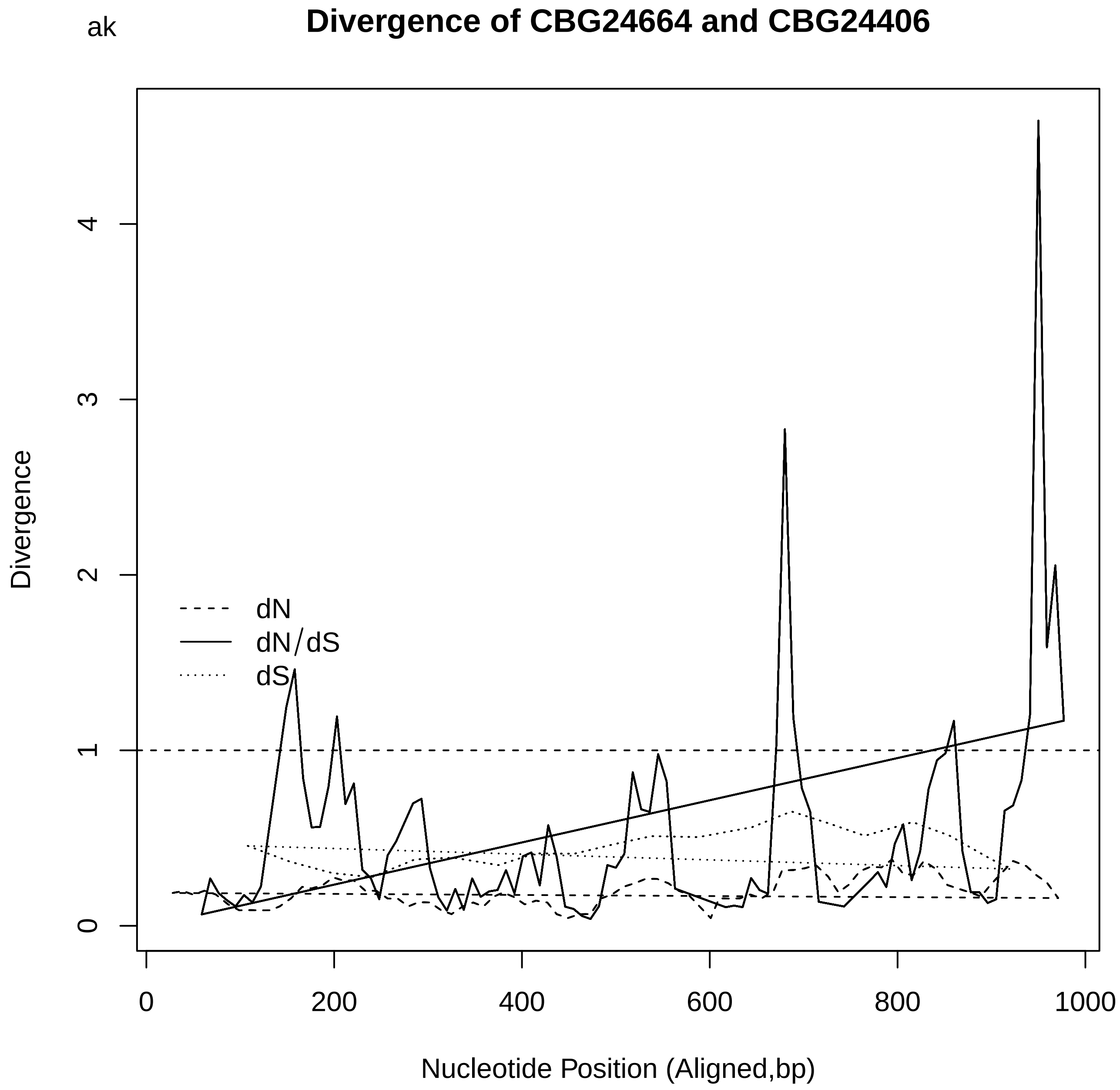

Divergence of CBG24664 and CBG24406
Divergence
Nucleotide Position (Aligned,bp)
dN
dN/dS
dS
0
1
2
3
4
0
200
400
600
800
1000
