# Supplementary material for: Genome-wide characterization, evolution, structure, and expression analysis of the F-box genes in Caenorhabditis
Source: BMC Genomics. 2021 Dec 11;22:889. doi: 10.1186/s12864-021-08189-7 (PMC8665587; doi:10.1186/s12864-021-08189-7)
Supplement: Supplementary file 5 — Additional file 5: Figure S5. The entirely phylogenetic relationships of F-box proteins from C. brenneri, C. briggsae, C. elegans, C. japonica, and C. remanei and P. pacificus being color-coded light sea green, chocolate, purple, pink, lime-green, and orange, respectively. F-box domain sequences were aligned using MUSCLE. The topology was generated by maximum likelihood analysis using RAxML. Statistical support was obtained by 100-bootstrap RAxML replicates, and schematic triangles denote bootstrap values greater than 50. [file 12864_2021_8189_MOESM5_ESM.pdf]

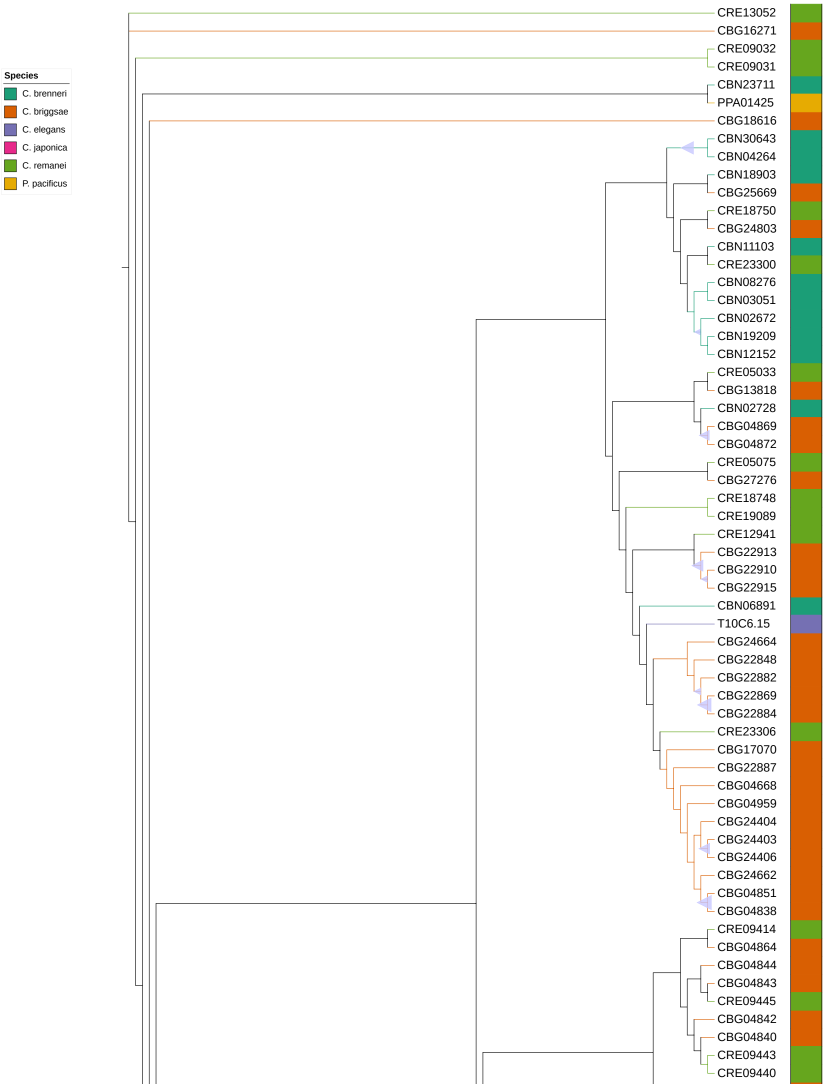
Species
C. brenneri
C. briggsae
C. elegans
C. japonica
C. remanei
P. pacificus
CRE13052
CBG16271
CRE09032
CRE09031
CBN23711
PPA01425
CBG18616
CBN30643
CBN04264
CBN18903
CBG25669
CRE18750
CBG24803
CBN11103
CRE23300
CBN08276
CBN03051
CBN02672
CBN19209
CBN12152
CRE05033
CBG13818
CBN02728
CBG04869
CBG04872
CRE05075
CBG27276
CRE18748
CRE19089
CRE12941
CBG22913
CBG22910
CBG22915
CBN06891
T10C6.15
CBG24664
CBG22848
CBG22882
CBG22869
CBG22884
CRE23306
CBG17070
CBG22887
CBG04668
CBG04959
CBG24404
CBG24403
CBG24406
CBG24662
CBG04851
CBG04838
CRE09414
CBG04864
CBG04844
CBG04843
CRE09445
CBG04842
CBG04840
CRE09443
CRE09440

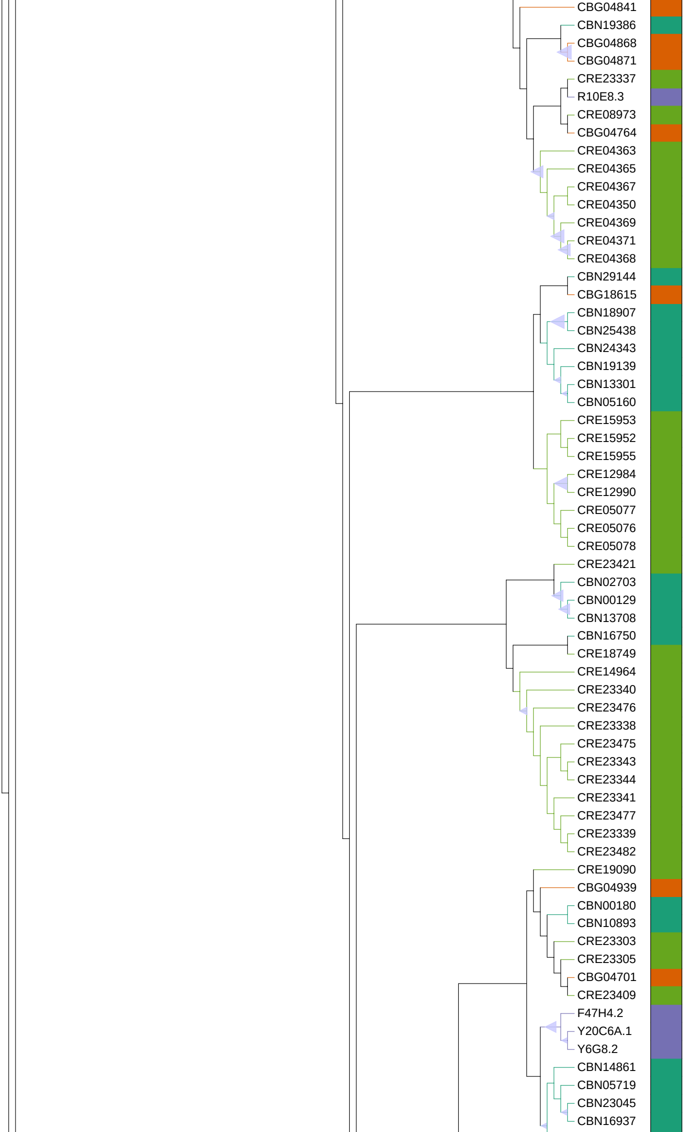
CBG04841
CBN19386
CBG04868
CBG04871
CRE23337
R10E8.3
CRE08973
CBG04764
CRE04363
CRE04365
CRE04367
CRE04350
CRE04369
CRE04371
CRE04368
CBN29144
CBG18615
CBN18907
CBN25438
CBN24343
CBN19139
CBN13301
CBN05160
CRE15953
CRE15952
CRE15955
CRE12984
CRE12990
CRE05077
CRE05076
CRE05078
CRE23421
CBN02703
CBN00129
CBN13708
CBN16750
CRE18749
CRE14964
CRE23340
CRE23476
CRE23338
CRE23475
CRE23343
CRE23344
CRE23341
CRE23477
CRE23339
CRE23482
CRE19090
CBG04939
CBN00180
CBN10893
CRE23303
CRE23305
CBG04701
CRE23409
F47H4.2
Y20C6A.1
Y6G8.2
CBN14861
CBN05719
CBN23045
CBN16937

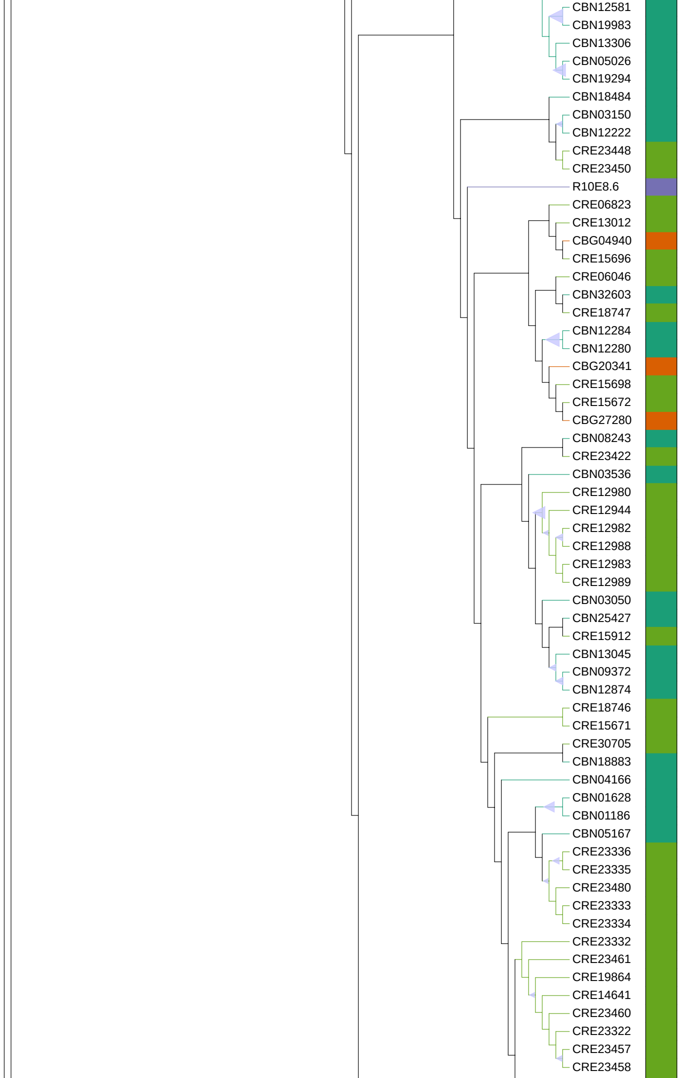
CBN12581
CBN19983
CBN13306
CBN05026
CBN19294
CBN18484
CBN03150
CBN12222
CRE23448
CRE23450
R10E8.6
CRE06823
CRE13012
CBG04940
CRE15696
CRE06046
CBN32603
CRE18747
CBN12284
CBN12280
CBG20341
CRE15698
CRE15672
CBG27280
CBN08243
CRE23422
CBN03536
CRE12980
CRE12944
CRE12982
CRE12988
CRE12983
CRE12989
CBN03050
CBN25427
CRE15912
CBN13045
CBN09372
CBN12874
CRE18746
CRE15671
CRE30705
CBN18883
CBN04166
CBN01628
CBN01186
CBN05167
CRE23336
CRE23335
CRE23480
CRE23333
CRE23334
CRE23332
CRE23461
CRE19864
CRE14641
CRE23460
CRE23322
CRE23457
CRE23458

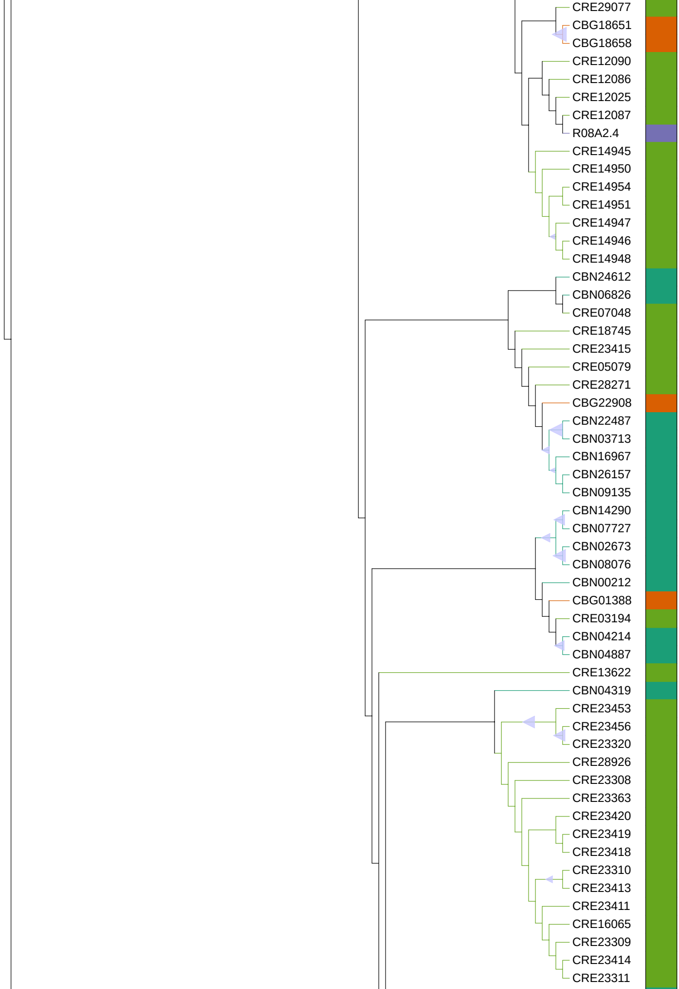
CRE29077
CBG18651
CBG18658
CRE12090
CRE12086
CRE12025
CRE12087
R08A2.4
CRE14945
CRE14950
CRE14954
CRE14951
CRE14947
CRE14946
CRE14948
CBN24612
CBN06826
CRE07048
CRE18745
CRE23415
CRE05079
CRE28271
CBG22908
CBN22487
CBN03713
CBN16967
CBN26157
CBN09135
CBN14290
CBN07727
CBN02673
CBN08076
CBN00212
CBG01388
CRE03194
CBN04214
CBN04887
CRE13622
CBN04319
CRE23453
CRE23456
CRE23320
CRE28926
CRE23308
CRE23363
CRE23420
CRE23419
CRE23418
CRE23310
CRE23413
CRE23411
CRE16065
CRE23309
CRE23414
CRE23311

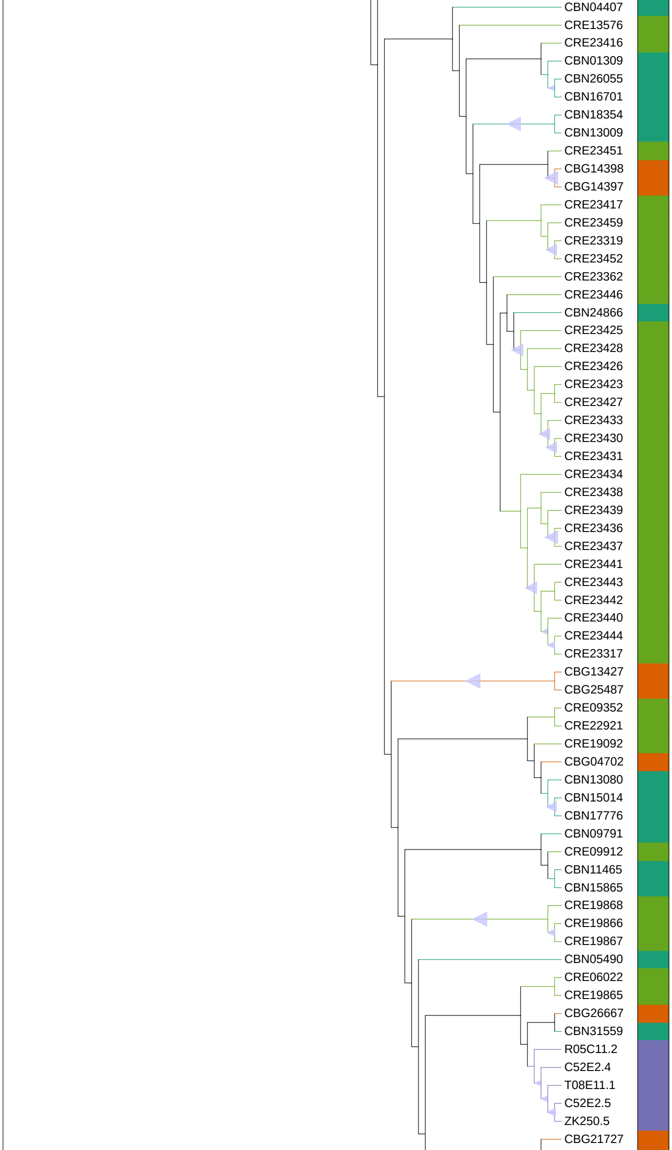
CBN04407
CRE13576
CRE23416
CBN01309
CBN26055
CBN16701
CBN18354
CBN13009
CRE23451
CBG14398
CBG14397
CRE23417
CRE23459
CRE23319
CRE23452
CRE23362
CRE23446
CBN24866
CRE23425
CRE23428
CRE23426
CRE23423
CRE23427
CRE23433
CRE23430
CRE23431
CRE23434
CRE23438
CRE23439
CRE23436
CRE23437
CRE23441
CRE23443
CRE23442
CRE23440
CRE23444
CRE23317
CBG13427
CBG25487
CRE09352
CRE22921
CRE19092
CBG04702
CBN13080
CBN15014
CBN17776
CBN09791
CRE09912
CBN11465
CBN15865
CRE19868
CRE19866
CRE19867
CBN05490
CRE06022
CRE19865
CBG26667
CBN31559
R05C11.2
C52E2.4
T08E11.1
C52E2.5
ZK250.5
CBG21727

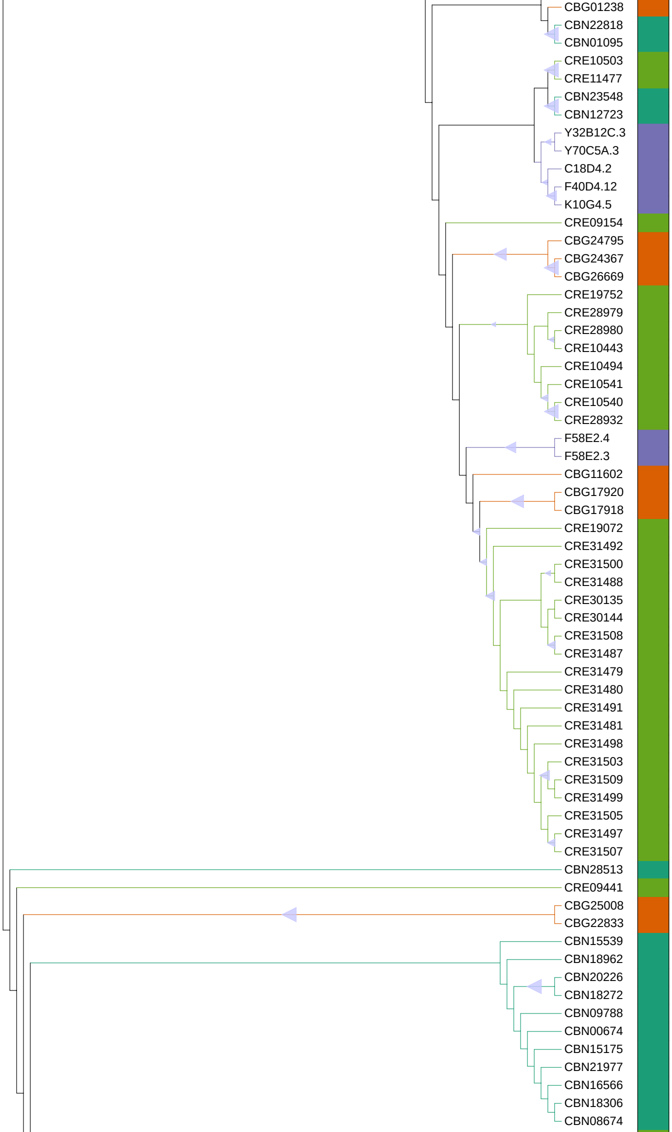
CBG01238
CBN22818
CBN01095
CRE10503
CRE11477
CBN23548
CBN12723
Y32B12C.3
Y70C5A.3
C18D4.2
F40D4.12
K10G4.5
CRE09154
CBG24795
CBG24367
CBG26669
CRE19752
CRE28979
CRE28980
CRE10443
CRE10494
CRE10541
CRE10540
CRE28932
F58E2.4
F58E2.3
CBG11602
CBG17920
CBG17918
CRE19072
CRE31492
CRE31500
CRE31488
CRE30135
CRE30144
CRE31508
CRE31487
CRE31479
CRE31480
CRE31491
CRE31481
CRE31498
CRE31503
CRE31509
CRE31499
CRE31505
CRE31497
CRE31507
CBN28513
CRE09441
CBG25008
CBG22833
CBN15539
CBN18962
CBN20226
CBN18272
CBN09788
CBN00674
CBN15175
CBN21977
CBN16566
CBN18306
CBN08674

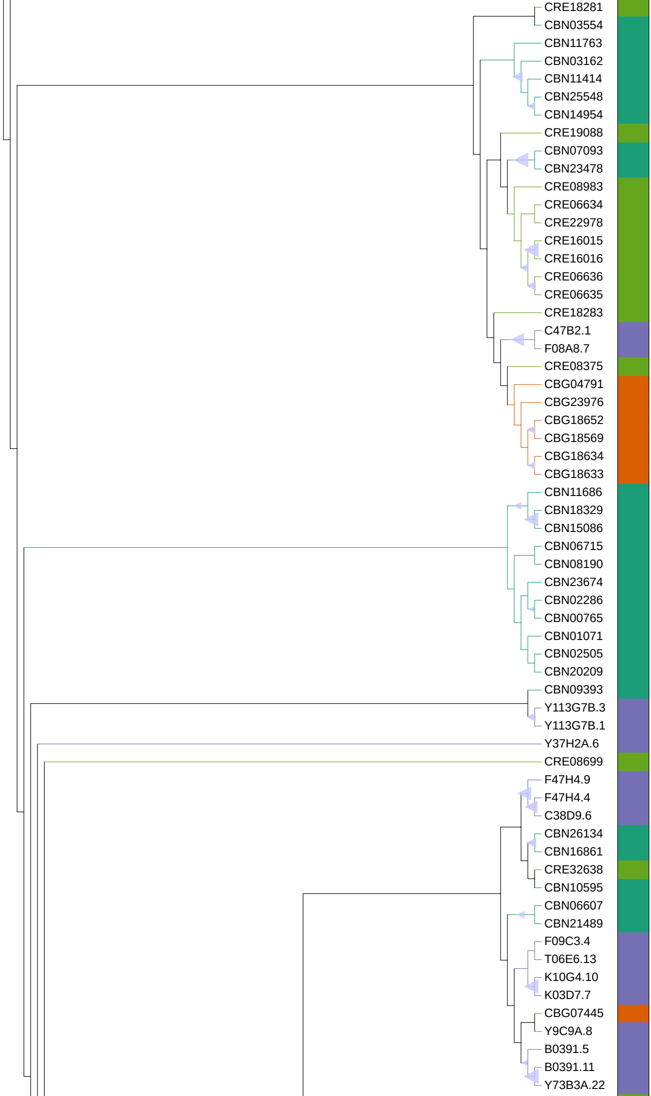
CRE18281
CBN03554
CBN11763
CBN03162
CBN11414
CBN25548
CBN14954
CRE19088
CBN07093
CBN23478
CRE08983
CRE06634
CRE22978
CRE16015
CRE16016
CRE06636
CRE06635
CRE18283
C47B2.1
F08A8.7
CRE08375
CBG04791
CBG23976
CBG18652
CBG18569
CBG18634
CBG18633
CBN11686
CBN18329
CBN15086
CBN06715
CBN08190
CBN23674
CBN02286
CBN00765
CBN01071
CBN02505
CBN20209
CBN09393
Y113G7B.3
Y113G7B.1
Y37H2A.6
CRE08699
F47H4.9
F47H4.4
C38D9.6
CBN26134
CBN16861
CRE32638
CBN10595
CBN06607
CBN21489
F09C3.4
T06E6.13
K10G4.10
K03D7.7
CBG07445
Y9C9A.8
B0391.5
B0391.11
Y73B3A.22

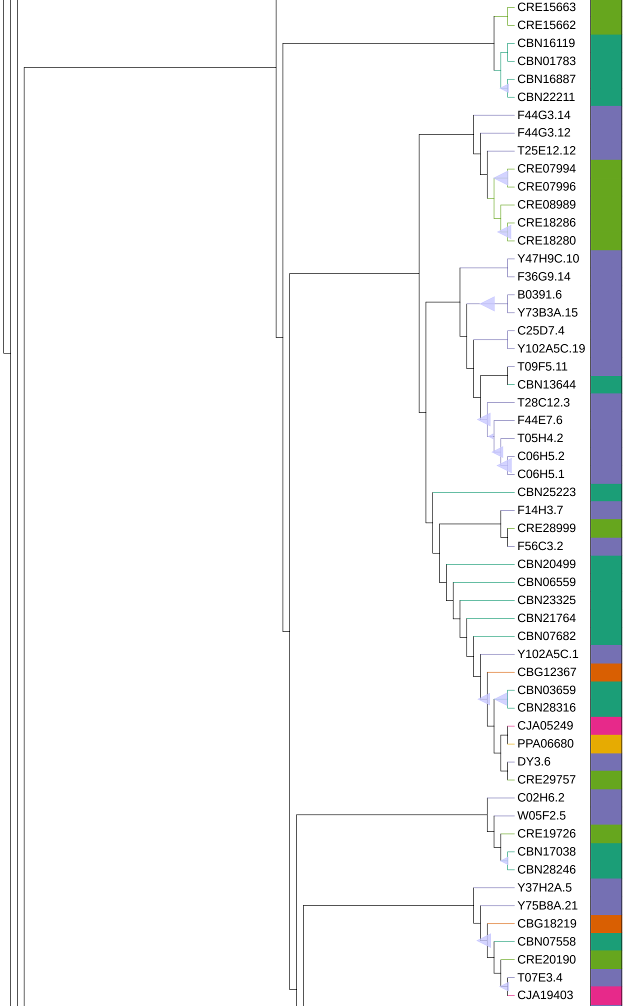
CRE15663
CRE15662
CBN16119
CBN01783
CBN16887
CBN22211
F44G3.14
F44G3.12
T25E12.12
CRE07994
CRE07996
CRE08989
CRE18286
CRE18280
Y47H9C.10
F36G9.14
B0391.6
Y73B3A.15
C25D7.4
Y102A5C.19
T09F5.11
CBN13644
T28C12.3
F44E7.6
T05H4.2
C06H5.2
C06H5.1
CBN25223
F14H3.7
CRE28999
F56C3.2
CBN20499
CBN06559
CBN23325
CBN21764
CBN07682
Y102A5C.1
CBG12367
CBN03659
CBN28316
CJA05249
PPA06680
DY3.6
CRE29757
C02H6.2
W05F2.5
CRE19726
CBN17038
CBN28246
Y37H2A.5
Y75B8A.21
CBG18219
CBN07558
CRE20190
T07E3.4
CJA19403

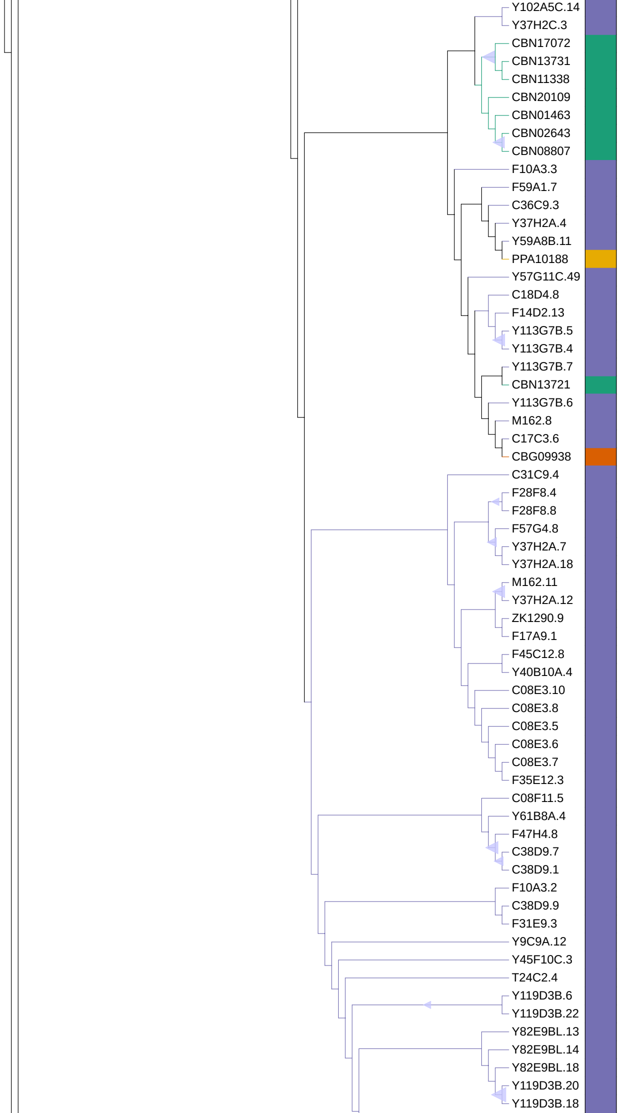
Y102A5C.14
Y37H2C.3
CBN17072
CBN13731
CBN11338
CBN20109
CBN01463
CBN02643
CBN08807
F10A3.3
F59A1.7
C36C9.3
Y37H2A.4
Y59A8B.11
PPA10188
Y57G11C.49
C18D4.8
F14D2.13
Y113G7B.5
Y113G7B.4
Y113G7B.7
CBN13721
Y113G7B.6
M162.8
C17C3.6
CBG09938
C31C9.4
F28F8.4
F28F8.8
F57G4.8
Y37H2A.7
Y37H2A.18
M162.11
Y37H2A.12
ZK1290.9
F17A9.1
F45C12.8
Y40B10A.4
C08E3.10
C08E3.8
C08E3.5
C08E3.6
C08E3.7
F35E12.3
C08F11.5
Y61B8A.4
F47H4.8
C38D9.7
C38D9.1
F10A3.2
C38D9.9
F31E9.3
Y9C9A.12
Y45F10C.3
T24C2.4
Y119D3B.6
Y119D3B.22
Y82E9BL.13
Y82E9BL.14
Y82E9BL.18
Y119D3B.20
Y119D3B.18

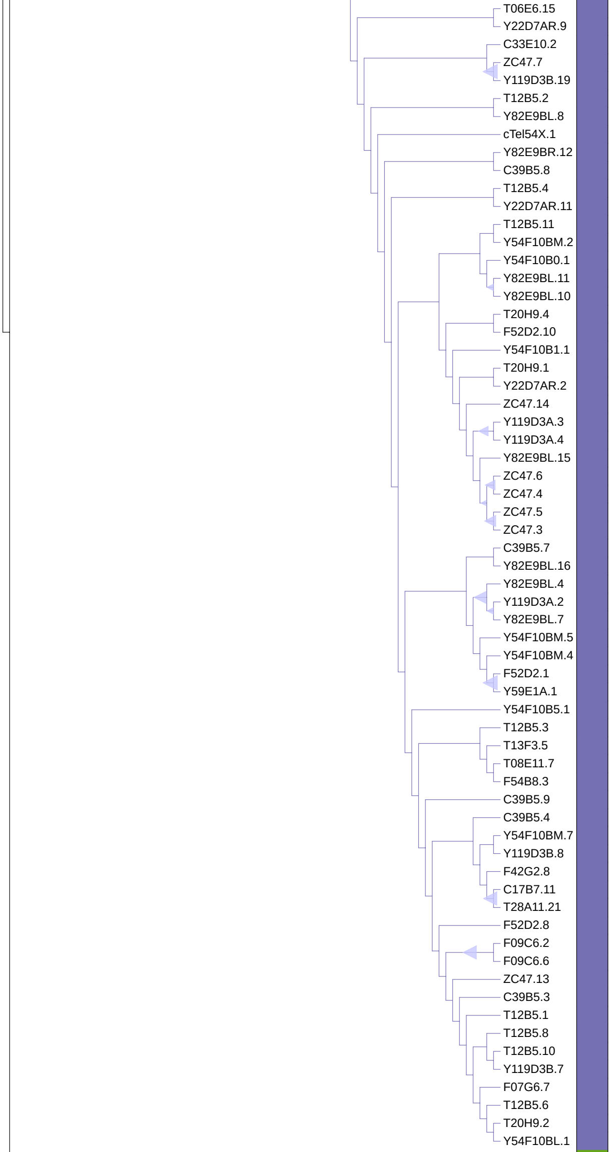
T06E6.15
Y22D7AR.9
C33E10.2
ZC47.7
Y119D3B.19
T12B5.2
Y82E9BL.8
cTel54X.1
Y82E9BR.12
C39B5.8
T12B5.4
Y22D7AR.11
T12B5.11
Y54F10BM.2
Y54F10B0.1
Y82E9BL.11
Y82E9BL.10
T20H9.4
F52D2.10
Y54F10B1.1
T20H9.1
Y22D7AR.2
ZC47.14
Y119D3A.3
Y119D3A.4
Y82E9BL.15
ZC47.6
ZC47.4
ZC47.5
ZC47.3
C39B5.7
Y82E9BL.16
Y82E9BL.4
Y119D3A.2
Y82E9BL.7
Y54F10BM.5
Y54F10BM.4
F52D2.1
Y59E1A.1
Y54F10B5.1
T12B5.3
T13F3.5
T08E11.7
F54B8.3
C39B5.9
C39B5.4
Y54F10BM.7
Y119D3B.8
F42G2.8
C17B7.11
T28A11.21
F52D2.8
F09C6.2
F09C6.6
ZC47.13
C39B5.3
T12B5.1
T12B5.8
T12B5.10
Y119D3B.7
F07G6.7
T12B5.6
T20H9.2
Y54F10BL.1

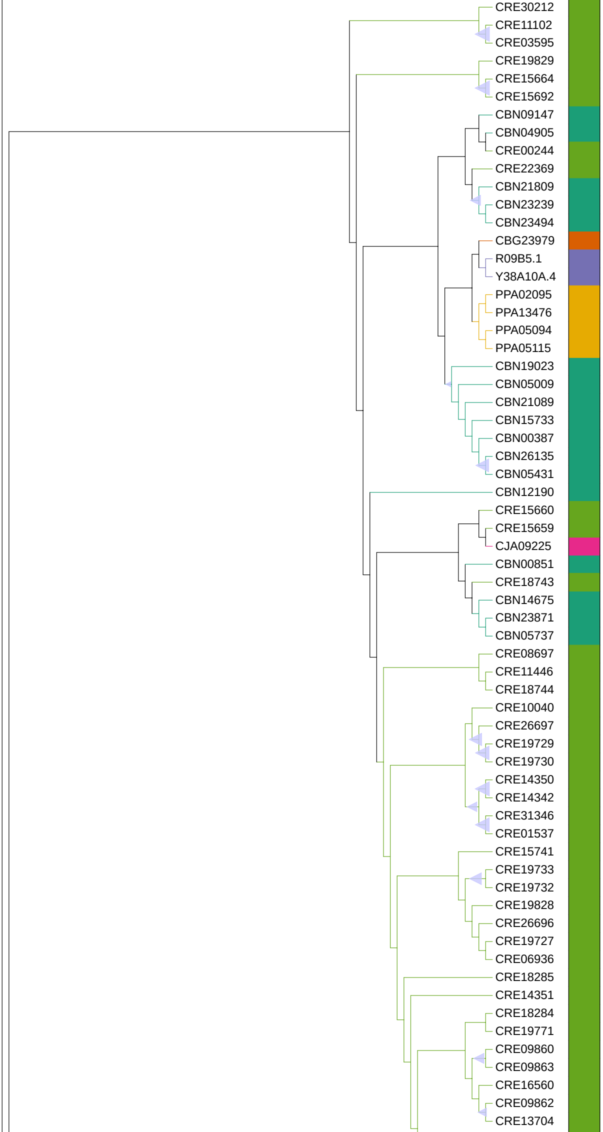
CRE30212
CRE11102
CRE03595
CRE19829
CRE15664
CRE15692
CBN09147
CBN04905
CRE00244
CRE22369
CBN21809
CBN23239
CBN23494
CBG23979
R09B5.1
Y38A10A.4
PPA02095
PPA13476
PPA05094
PPA05115
CBN19023
CBN05009
CBN21089
CBN15733
CBN00387
CBN26135
CBN05431
CBN12190
CRE15660
CRE15659
CJA09225
CBN00851
CRE18743
CBN14675
CBN23871
CBN05737
CRE08697
CRE11446
CRE18744
CRE10040
CRE26697
CRE19729
CRE19730
CRE14350
CRE14342
CRE31346
CRE01537
CRE15741
CRE19733
CRE19732
CRE19828
CRE26696
CRE19727
CRE06936
CRE18285
CRE14351
CRE18284
CRE19771
CRE09860
CRE09863
CRE16560
CRE09862
CRE13704

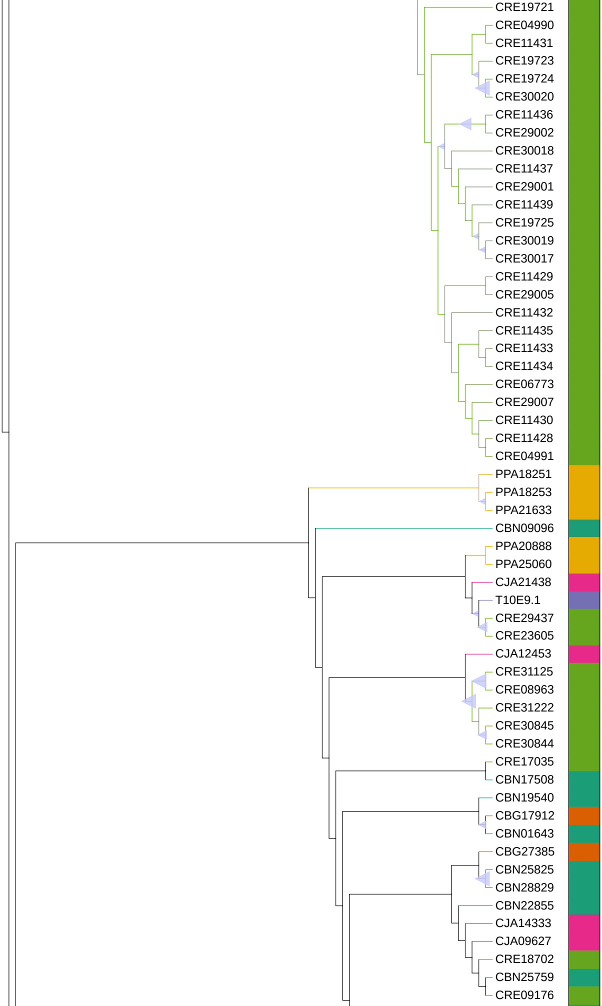
CRE19721
CRE04990
CRE11431
CRE19723
CRE19724
CRE30020
CRE11436
CRE29002
CRE30018
CRE11437
CRE29001
CRE11439
CRE19725
CRE30019
CRE30017
CRE11429
CRE29005
CRE11432
CRE11435
CRE11433
CRE11434
CRE06773
CRE29007
CRE11430
CRE11428
CRE04991
PPA18251
PPA18253
PPA21633
CBN09096
PPA20888
PPA25060
CJA21438
T10E9.1
CRE29437
CRE23605
CJA12453
CRE31125
CRE08963
CRE31222
CRE30845
CRE30844
CRE17035
CBN17508
CBN19540
CBG17912
CBN01643
CBG27385
CBN25825
CBN28829
CBN22855
CJA14333
CJA09627
CRE18702
CBN25759
CRE09176

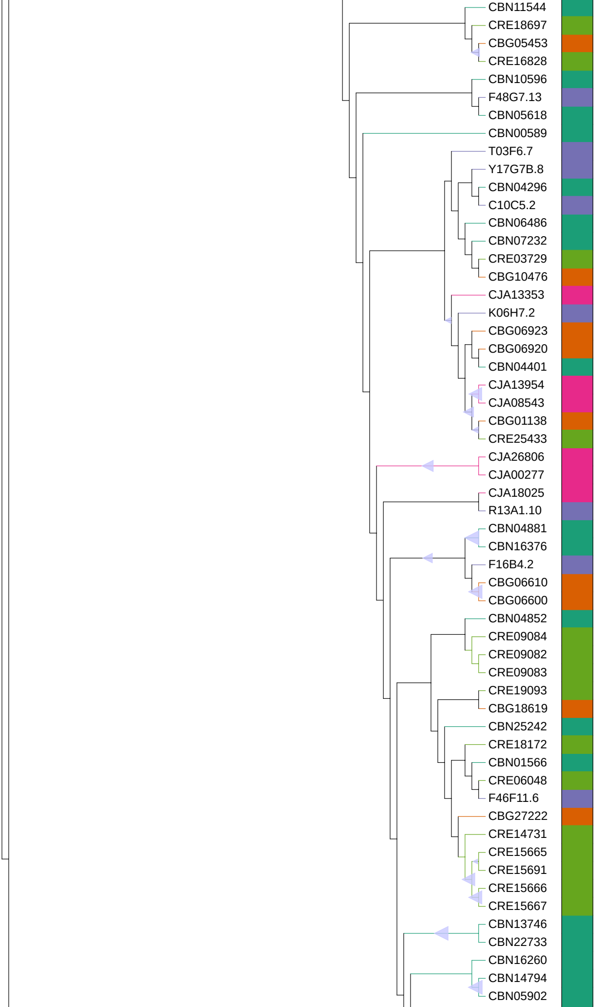
CBN11544
CRE18697
CBG05453
CRE16828
CBN10596
F48G7.13
CBN05618
CBN00589
T03F6.7
Y17G7B.8
CBN04296
C10C5.2
CBN06486
CBN07232
CRE03729
CBG10476
CJA13353
K06H7.2
CBG06923
CBG06920
CBN04401
CJA13954
CJA08543
CBG01138
CRE25433
CJA26806
CJA00277
CJA18025
R13A1.10
CBN04881
CBN16376
F16B4.2
CBG06610
CBG06600
CBN04852
CRE09084
CRE09082
CRE09083
CRE19093
CBG18619
CBN25242
CRE18172
CBN01566
CRE06048
F46F11.6
CBG27222
CRE14731
CRE15665
CRE15691
CRE15666
CRE15667
CBN13746
CBN22733
CBN16260
CBN14794
CBN05902

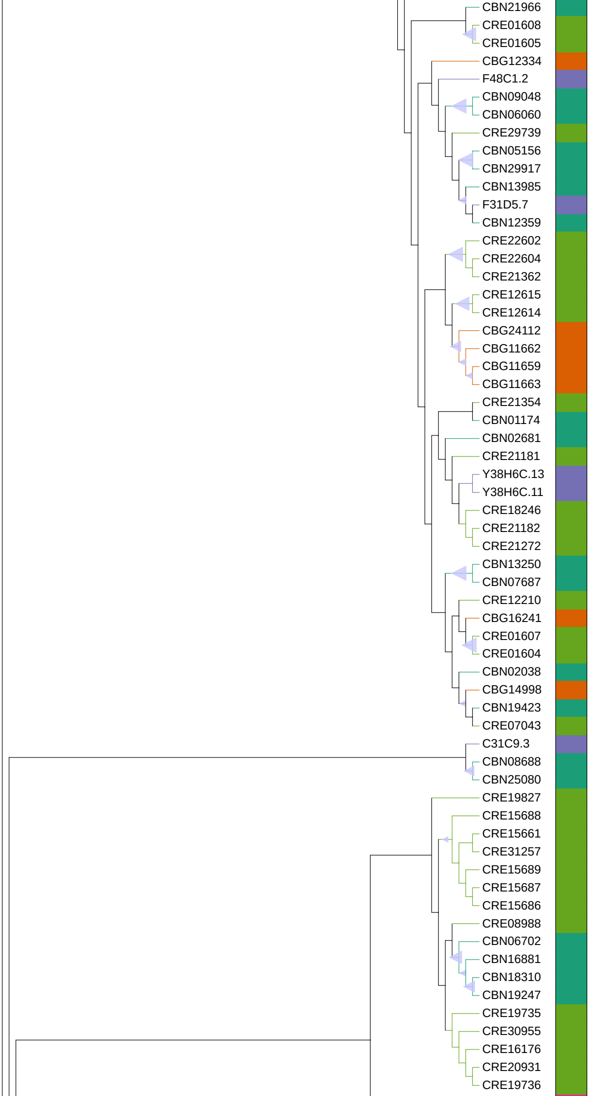
CBN21966
CRE01608
CRE01605
CBG12334
F48C1.2
CBN09048
CBN06060
CRE29739
CBN05156
CBN29917
CBN13985
F31D5.7
CBN12359
CRE22602
CRE22604
CRE21362
CRE12615
CRE12614
CBG24112
CBG11662
CBG11659
CBG11663
CRE21354
CBN01174
CBN02681
CRE21181
Y38H6C.13
Y38H6C.11
CRE18246
CRE21182
CRE21272
CBN13250
CBN07687
CRE12210
CBG16241
CRE01607
CRE01604
CBN02038
CBG14998
CBN19423
CRE07043
C31C9.3
CBN08688
CBN25080
CRE19827
CRE15688
CRE15661
CRE31257
CRE15689
CRE15687
CRE15686
CRE08988
CBN06702
CBN16881
CBN18310
CBN19247
CRE19735
CRE30955
CRE16176
CRE20931
CRE19736

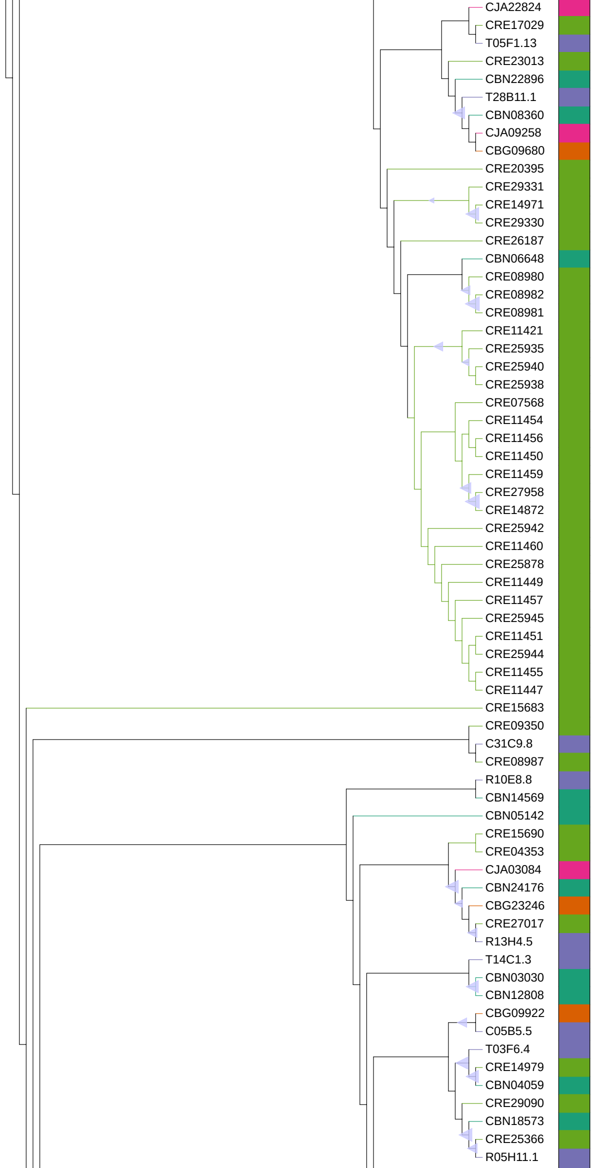
CJA22824
CRE17029
T05F1.13
CRE23013
CBN22896
T28B11.1
CBN08360
CJA09258
CBG09680
CRE20395
CRE29331
CRE14971
CRE29330
CRE26187
CBN06648
CRE08980
CRE08982
CRE08981
CRE11421
CRE25935
CRE25940
CRE25938
CRE07568
CRE11454
CRE11456
CRE11450
CRE11459
CRE27958
CRE14872
CRE25942
CRE11460
CRE25878
CRE11449
CRE11457
CRE25945
CRE11451
CRE25944
CRE11455
CRE11447
CRE15683
CRE09350
C31C9.8
CRE08987
R10E8.8
CBN14569
CBN05142
CRE15690
CRE04353
CJA03084
CBN24176
CBG23246
CRE27017
R13H4.5
T14C1.3
CBN03030
CBN12808
CBG09922
C05B5.5
T03F6.4
CRE14979
CBN04059
CRE29090
CBN18573
CRE25366
R05H11.1

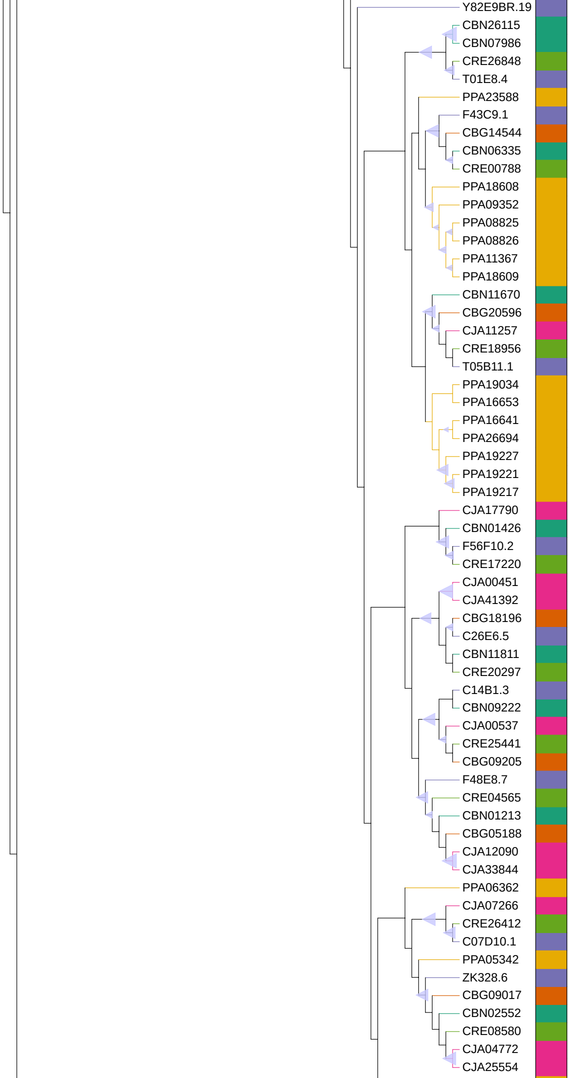
Y82E9BR.19
CBN26115
CBN07986
CRE26848
T01E8.4
PPA23588
F43C9.1
CBG14544
CBN06335
CRE00788
PPA18608
PPA09352
PPA08825
PPA08826
PPA11367
PPA18609
CBN11670
CBG20596
CJA11257
CRE18956
T05B11.1
PPA19034
PPA16653
PPA16641
PPA26694
PPA19227
PPA19221
PPA19217
CJA17790
CBN01426
F56F10.2
CRE17220
CJA00451
CJA41392
CBG18196
C26E6.5
CBN11811
CRE20297
C14B1.3
CBN09222
CJA00537
CRE25441
CBG09205
F48E8.7
CRE04565
CBN01213
CBG05188
CJA12090
CJA33844
PPA06362
CJA07266
CRE26412
C07D10.1
PPA05342
ZK328.6
CBG09017
CBN02552
CRE08580
CJA04772
CJA25554

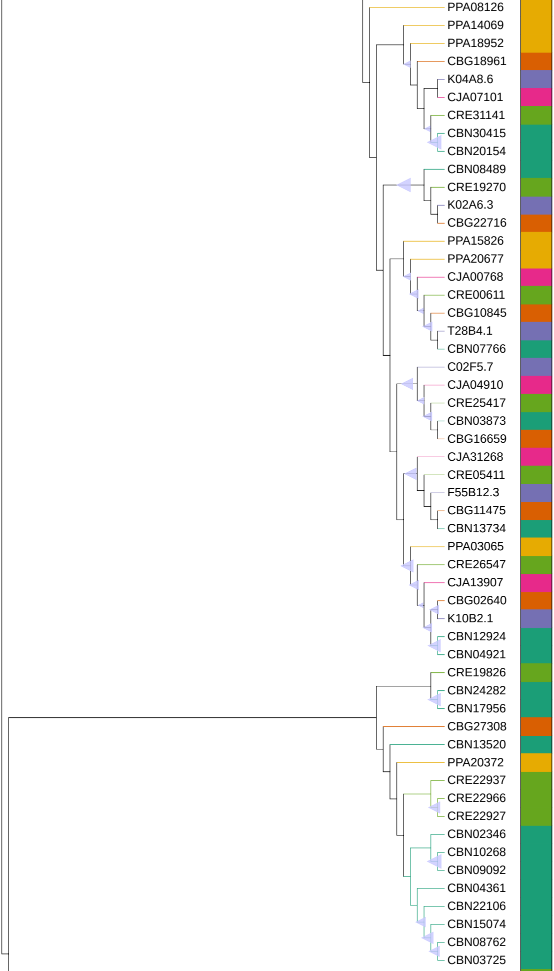
PPA08126
PPA14069
PPA18952
CBG18961
K04A8.6
CJA07101
CRE31141
CBN30415
CBN20154
CBN08489
CRE19270
K02A6.3
CBG22716
PPA15826
PPA20677
CJA00768
CRE00611
CBG10845
T28B4.1
CBN07766
C02F5.7
CJA04910
CRE25417
CBN03873
CBG16659
CJA31268
CRE05411
F55B12.3
CBG11475
CBN13734
PPA03065
CRE26547
CJA13907
CBG02640
K10B2.1
CBN12924
CBN04921
CRE19826
CBN24282
CBN17956
CBG27308
CBN13520
PPA20372
CRE22937
CRE22966
CRE22927
CBN02346
CBN10268
CBN09092
CBN04361
CBN22106
CBN15074
CBN08762
CBN03725

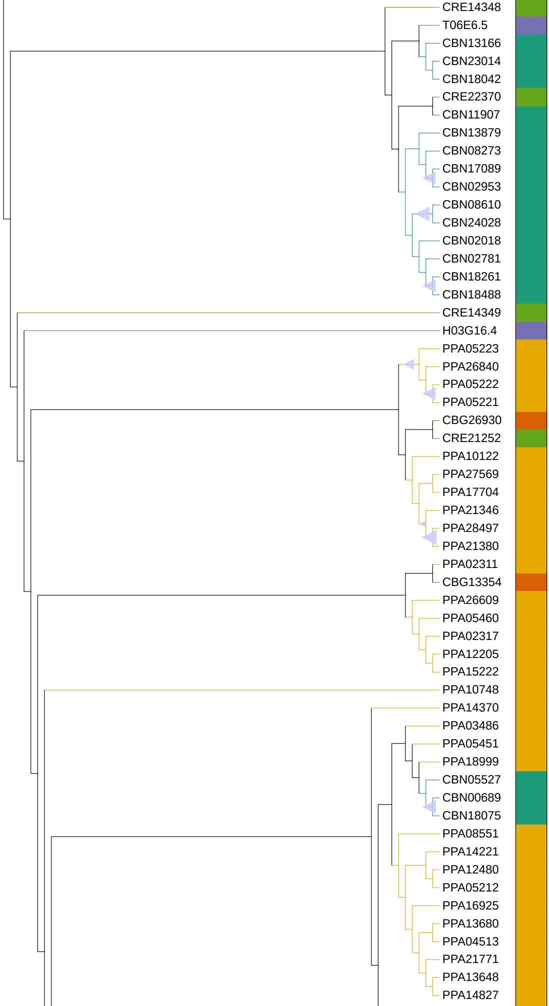
CRE14348
T06E6.5
CBN13166
CBN23014
CBN18042
CRE22370
CBN11907
CBN13879
CBN08273
CBN17089
CBN02953
CBN08610
CBN24028
CBN02018
CBN02781
CBN18261
CBN18488
CRE14349
H03G16.4
PPA05223
PPA26840
PPA05222
PPA05221
CBG26930
CRE21252
PPA10122
PPA27569
PPA17704
PPA21346
PPA28497
PPA21380
PPA02311
CBG13354
PPA26609
PPA05460
PPA02317
PPA12205
PPA15222
PPA10748
PPA14370
PPA03486
PPA05451
PPA18999
CBN05527
CBN00689
CBN18075
PPA08551
PPA14221
PPA12480
PPA05212
PPA16925
PPA13680
PPA04513
PPA21771
PPA13648
PPA14827

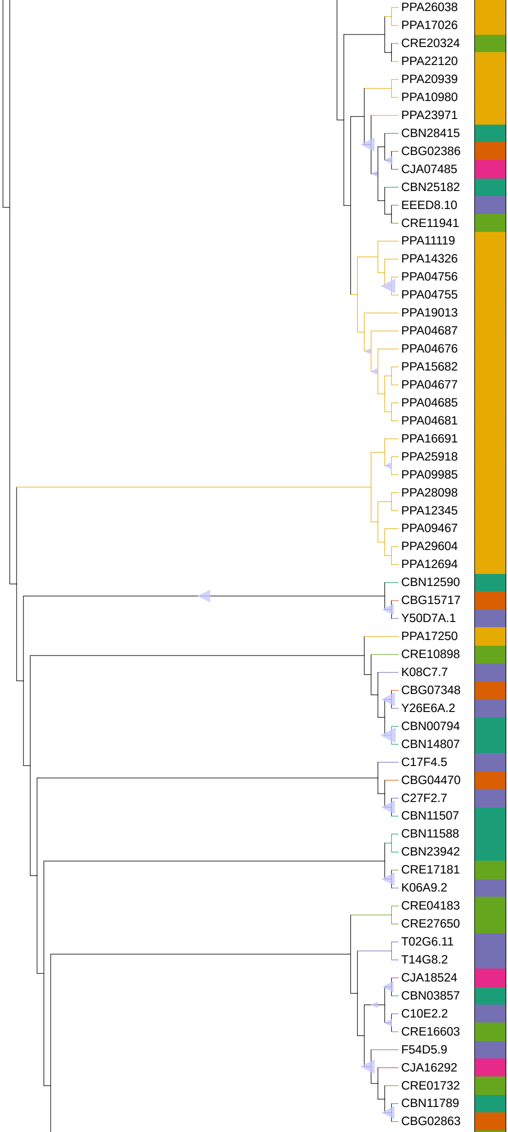
PPA26038
PPA17026
CRE20324
PPA22120
PPA20939
PPA10980
PPA23971
CBN28415
CBG02386
CJA07485
CBN25182
EEED8.10
CRE11941
PPA11119
PPA14326
PPA04756
PPA04755
PPA19013
PPA04687
PPA04676
PPA15682
PPA04677
PPA04685
PPA04681
PPA16691
PPA25918
PPA09985
PPA28098
PPA12345
PPA09467
PPA29604
PPA12694
CBN12590
CBG15717
Y50D7A.1
PPA17250
CRE10898
K08C7.7
CBG07348
Y26E6A.2
CBN00794
CBN14807
C17F4.5
CBG04470
C27F2.7
CBN11507
CBN11588
CBN23942
CRE17181
K06A9.2
CRE04183
CRE27650
T02G6.11
T14G8.2
CJA18524
CBN03857
C10E2.2
CRE16603
F54D5.9
CJA16292
CRE01732
CBN11789
CBG02863

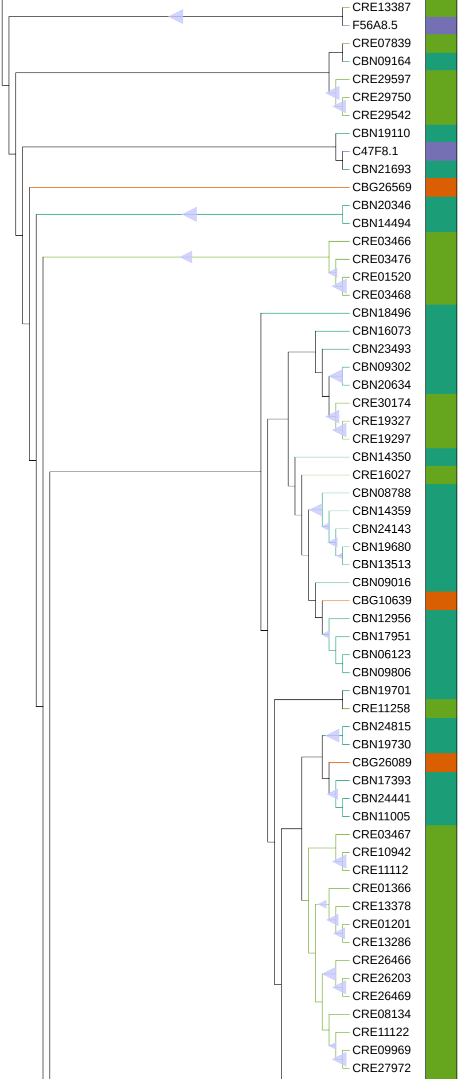
CRE13387
F56A8.5
CRE07839
CBN09164
CRE29597
CRE29750
CRE29542
CBN19110
C47F8.1
CBN21693
CBG26569
CBN20346
CBN14494
CRE03466
CRE03476
CRE01520
CRE03468
CBN18496
CBN16073
CBN23493
CBN09302
CBN20634
CRE30174
CRE19327
CRE19297
CBN14350
CRE16027
CBN08788
CBN14359
CBN24143
CBN19680
CBN13513
CBN09016
CBG10639
CBN12956
CBN17951
CBN06123
CBN09806
CBN19701
CRE11258
CBN24815
CBN19730
CBG26089
CBN17393
CBN24441
CBN11005
CRE03467
CRE10942
CRE11112
CRE01366
CRE13378
CRE01201
CRE13286
CRE26466
CRE26203
CRE26469
CRE08134
CRE11122
CRE09969
CRE27972

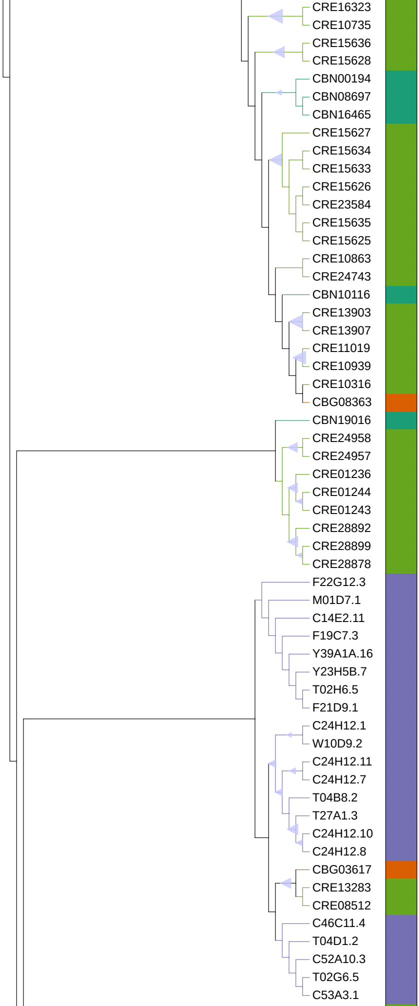
CRE16323
CRE10735
CRE15636
CRE15628
CBN00194
CBN08697
CBN16465
CRE15627
CRE15634
CRE15633
CRE15626
CRE23584
CRE15635
CRE15625
CRE10863
CRE24743
CBN10116
CRE13903
CRE13907
CRE11019
CRE10939
CRE10316
CBG08363
CBN19016
CRE24958
CRE24957
CRE01236
CRE01244
CRE01243
CRE28892
CRE28899
CRE28878
F22G12.3
M01D7.1
C14E2.11
F19C7.3
Y39A1A.16
Y23H5B.7
T02H6.5
F21D9.1
C24H12.1
W10D9.2
C24H12.11
C24H12.7
T04B8.2
T27A1.3
C24H12.10
C24H12.8
CBG03617
CRE13283
CRE08512
C46C11.4
T04D1.2
C52A10.3
T02G6.5
C53A3.1

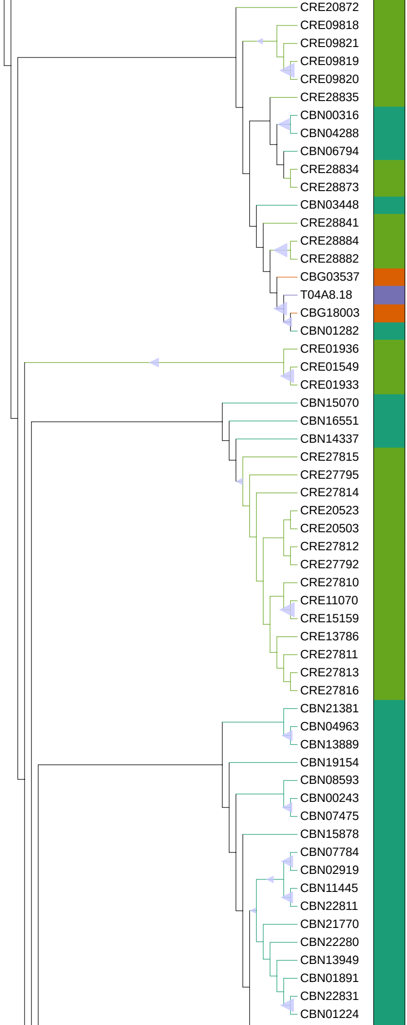
CRE20872
CRE09818
CRE09821
CRE09819
CRE09820
CRE28835
CBN00316
CBN04288
CBN06794
CRE28834
CRE28873
CBN03448
CRE28841
CRE28884
CRE28882
CBG03537
T04A8.18
CBG18003
CBN01282
CRE01936
CRE01549
CRE01933
CBN15070
CBN16551
CBN14337
CRE27815
CRE27795
CRE27814
CRE20523
CRE20503
CRE27812
CRE27792
CRE27810
CRE11070
CRE15159
CRE13786
CRE27811
CRE27813
CRE27816
CBN21381
CBN04963
CBN13889
CBN19154
CBN08593
CBN00243
CBN07475
CBN15878
CBN07784
CBN02919
CBN11445
CBN22811
CBN21770
CBN22280
CBN13949
CBN01891
CBN22831
CBN01224

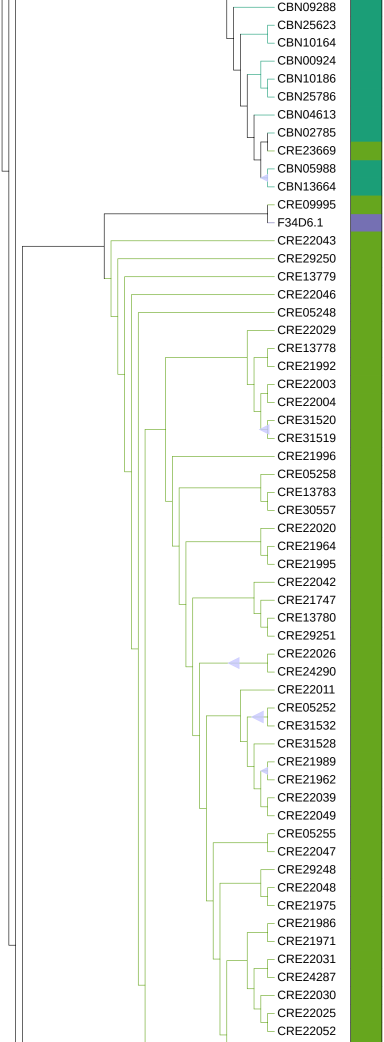
CBN09288
CBN25623
CBN10164
CBN00924
CBN10186
CBN25786
CBN04613
CBN02785
CRE23669
CBN05988
CBN13664
CRE09995
F34D6.1
CRE22043
CRE29250
CRE13779
CRE22046
CRE05248
CRE22029
CRE13778
CRE21992
CRE22003
CRE22004
CRE31520
CRE31519
CRE21996
CRE05258
CRE13783
CRE30557
CRE22020
CRE21964
CRE21995
CRE22042
CRE21747
CRE13780
CRE29251
CRE22026
CRE24290
CRE22011
CRE05252
CRE31532
CRE31528
CRE21989
CRE21962
CRE22039
CRE22049
CRE05255
CRE22047
CRE29248
CRE22048
CRE21975
CRE21986
CRE21971
CRE22031
CRE24287
CRE22030
CRE22025
CRE22052

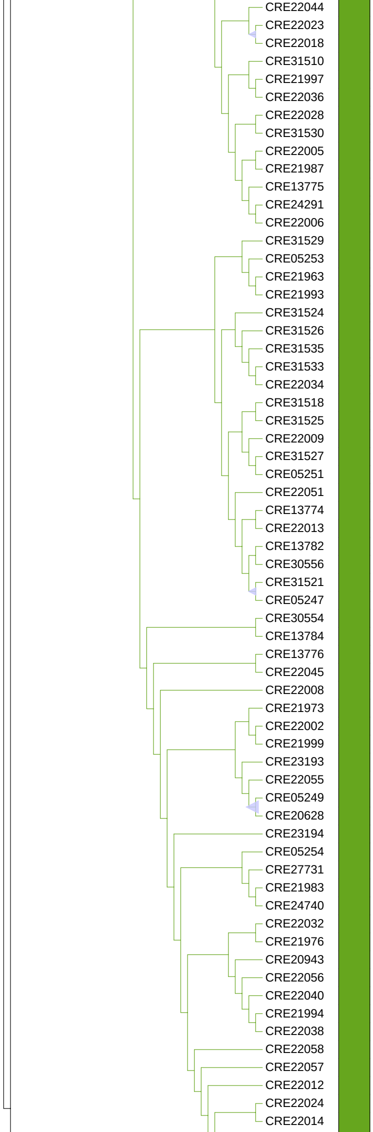
CRE22044
CRE22023
CRE22018
CRE31510
CRE21997
CRE22036
CRE22028
CRE31530
CRE22005
CRE21987
CRE13775
CRE24291
CRE22006
CRE31529
CRE05253
CRE21963
CRE21993
CRE31524
CRE31526
CRE31535
CRE31533
CRE22034
CRE31518
CRE31525
CRE22009
CRE31527
CRE05251
CRE22051
CRE13774
CRE22013
CRE13782
CRE30556
CRE31521
CRE05247
CRE30554
CRE13784
CRE13776
CRE22045
CRE22008
CRE21973
CRE22002
CRE21999
CRE23193
CRE22055
CRE05249
CRE20628
CRE23194
CRE05254
CRE27731
CRE21983
CRE24740
CRE22032
CRE21976
CRE20943
CRE22056
CRE22040
CRE21994
CRE22038
CRE22058
CRE22057
CRE22012
CRE22024
CRE22014

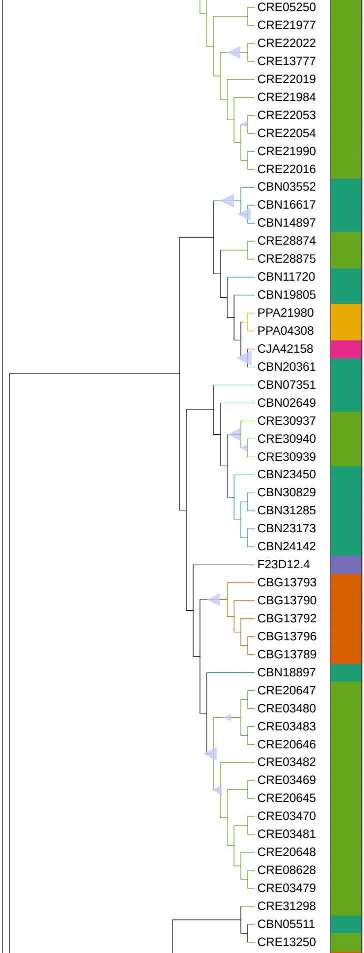
CRE05250
CRE21977
CRE22022
CRE13777
CRE22019
CRE21984
CRE22053
CRE22054
CRE21990
CRE22016
CBN03552
CBN16617
CBN14897
CRE28874
CRE28875
CBN11720
CBN19805
PPA21980
PPA04308
CJA42158
CBN20361
CBN07351
CBN02649
CRE30937
CRE30940
CRE30939
CBN23450
CBN30829
CBN31285
CBN23173
CBN24142
F23D12.4
CBG13793
CBG13790
CBG13792
CBG13796
CBG13789
CBN18897
CRE20647
CRE03480
CRE03483
CRE20646
CRE03482
CRE03469
CRE20645
CRE03470
CRE03481
CRE20648
CRE08628
CRE03479
CRE31298
CBN05511
CRE13250

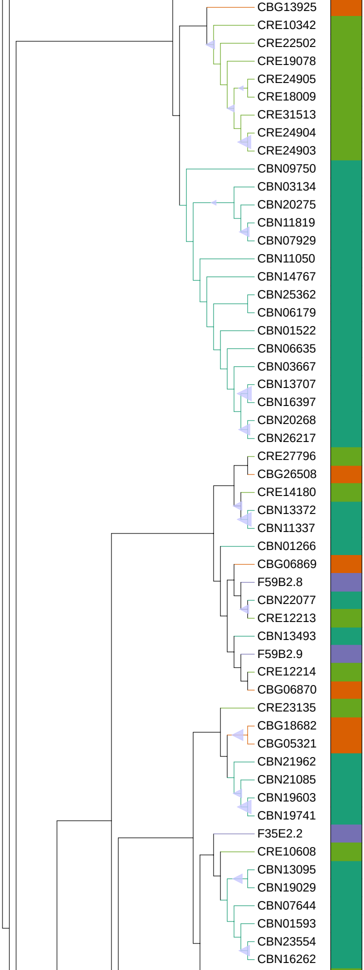
CBG13925
CRE10342
CRE22502
CRE19078
CRE24905
CRE18009
CRE31513
CRE24904
CRE24903
CBN09750
CBN03134
CBN20275
CBN11819
CBN07929
CBN11050
CBN14767
CBN25362
CBN06179
CBN01522
CBN06635
CBN03667
CBN13707
CBN16397
CBN20268
CBN26217
CRE27796
CBG26508
CRE14180
CBN13372
CBN11337
CBN01266
CBG06869
F59B2.8
CBN22077
CRE12213
CBN13493
F59B2.9
CRE12214
CBG06870
CRE23135
CBG18682
CBG05321
CBN21962
CBN21085
CBN19603
CBN19741
F35E2.2
CRE10608
CBN13095
CBN19029
CBN07644
CBN01593
CBN23554
CBN16262

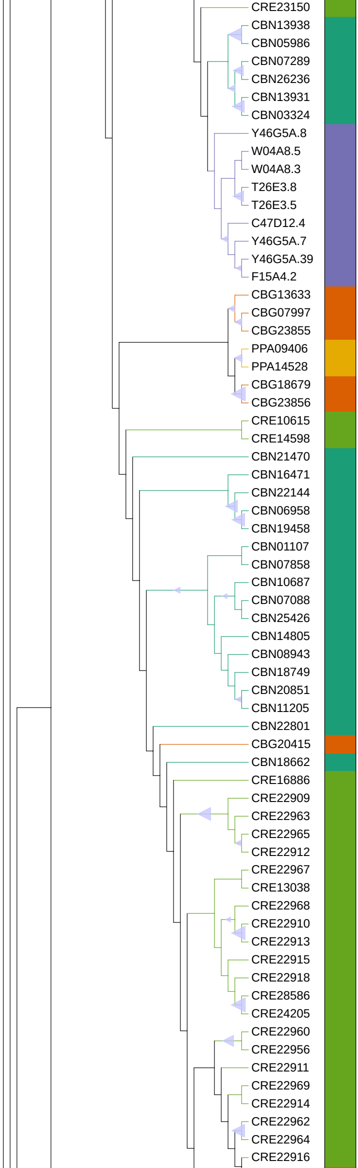
CRE23150
CBN13938
CBN05986
CBN07289
CBN26236
CBN13931
CBN03324
Y46G5A.8
W04A8.5
W04A8.3
T26E3.8
T26E3.5
C47D12.4
Y46G5A.7
Y46G5A.39
F15A4.2
CBG13633
CBG07997
CBG23855
PPA09406
PPA14528
CBG18679
CBG23856
CRE10615
CRE14598
CBN21470
CBN16471
CBN22144
CBN06958
CBN19458
CBN01107
CBN07858
CBN10687
CBN07088
CBN25426
CBN14805
CBN08943
CBN18749
CBN20851
CBN11205
CBN22801
CBG20415
CBN18662
CRE16886
CRE22909
CRE22963
CRE22965
CRE22912
CRE22967
CRE13038
CRE22968
CRE22910
CRE22913
CRE22915
CRE22918
CRE28586
CRE24205
CRE22960
CRE22956
CRE22911
CRE22969
CRE22914
CRE22962
CRE22964
CRE22916

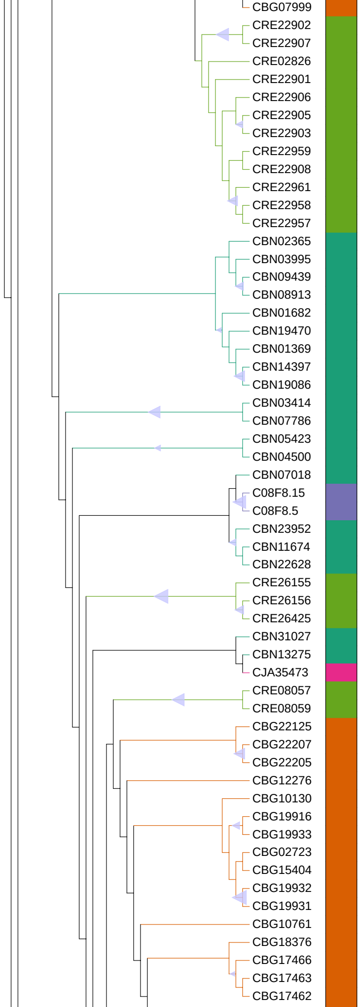
CBG07999
CRE22902
CRE22907
CRE02826
CRE22901
CRE22906
CRE22905
CRE22903
CRE22959
CRE22908
CRE22961
CRE22958
CRE22957
CBN02365
CBN03995
CBN09439
CBN08913
CBN01682
CBN19470
CBN01369
CBN14397
CBN19086
CBN03414
CBN07786
CBN05423
CBN04500
CBN07018
C08F8.15
C08F8.5
CBN23952
CBN11674
CBN22628
CRE26155
CRE26156
CRE26425
CBN31027
CBN13275
CJA35473
CRE08057
CRE08059
CBG22125
CBG22207
CBG22205
CBG12276
CBG10130
CBG19916
CBG19933
CBG02723
CBG15404
CBG19932
CBG19931
CBG10761
CBG18376
CBG17466
CBG17463
CBG17462

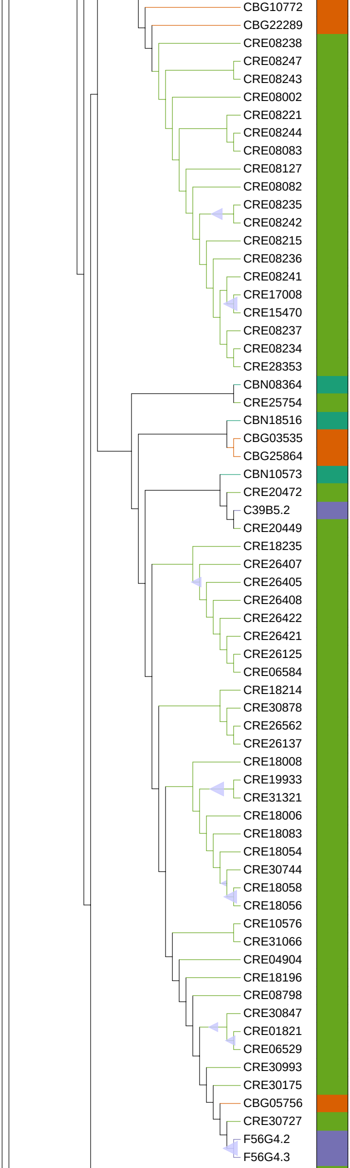
CBG10772
CBG22289
CRE08238
CRE08247
CRE08243
CRE08002
CRE08221
CRE08244
CRE08083
CRE08127
CRE08082
CRE08235
CRE08242
CRE08215
CRE08236
CRE08241
CRE17008
CRE15470
CRE08237
CRE08234
CRE28353
CBN08364
CRE25754
CBN18516
CBG03535
CBG25864
CBN10573
CRE20472
C39B5.2
CRE20449
CRE18235
CRE26407
CRE26405
CRE26408
CRE26422
CRE26421
CRE26125
CRE06584
CRE18214
CRE30878
CRE26562
CRE26137
CRE18008
CRE19933
CRE31321
CRE18006
CRE18083
CRE18054
CRE30744
CRE18058
CRE18056
CRE10576
CRE31066
CRE04904
CRE18196
CRE08798
CRE30847
CRE01821
CRE06529
CRE30993
CRE30175
CBG05756
CRE30727
F56G4.2
F56G4.3

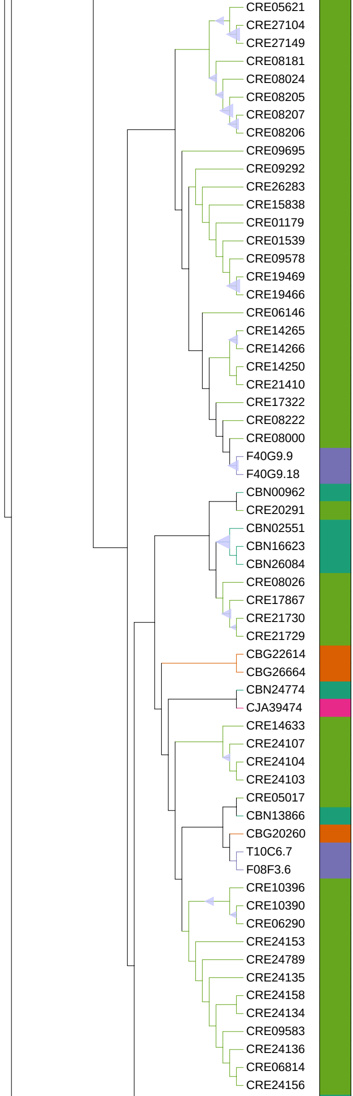
CRE05621
CRE27104
CRE27149
CRE08181
CRE08024
CRE08205
CRE08207
CRE08206
CRE09695
CRE09292
CRE26283
CRE15838
CRE01179
CRE01539
CRE09578
CRE19469
CRE19466
CRE06146
CRE14265
CRE14266
CRE14250
CRE21410
CRE17322
CRE08222
CRE08000
F40G9.9
F40G9.18
CBN00962
CRE20291
CBN02551
CBN16623
CBN26084
CRE08026
CRE17867
CRE21730
CRE21729
CBG22614
CBG26664
CBN24774
CJA39474
CRE14633
CRE24107
CRE24104
CRE24103
CRE05017
CBN13866
CBG20260
T10C6.7
F08F3.6
CRE10396
CRE10390
CRE06290
CRE24153
CRE24789
CRE24135
CRE24158
CRE24134
CRE09583
CRE24136
CRE06814
CRE24156

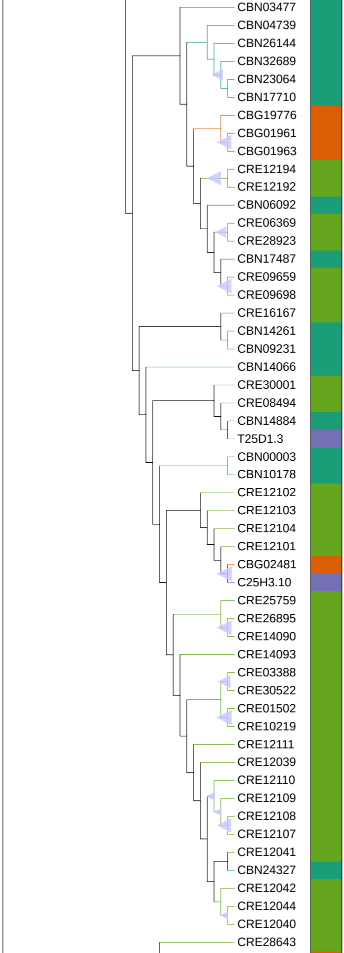
CBN03477
CBN04739
CBN26144
CBN32689
CBN23064
CBN17710
CBG19776
CBG01961
CBG01963
CRE12194
CRE12192
CBN06092
CRE06369
CRE28923
CBN17487
CRE09659
CRE09698
CRE16167
CBN14261
CBN09231
CBN14066
CRE30001
CRE08494
CBN14884
T25D1.3
CBN00003
CBN10178
CRE12102
CRE12103
CRE12104
CRE12101
CBG02481
C25H3.10
CRE25759
CRE26895
CRE14090
CRE14093
CRE03388
CRE30522
CRE01502
CRE10219
CRE12111
CRE12039
CRE12110
CRE12109
CRE12108
CRE12107
CRE12041
CBN24327
CRE12042
CRE12044
CRE12040
CRE28643

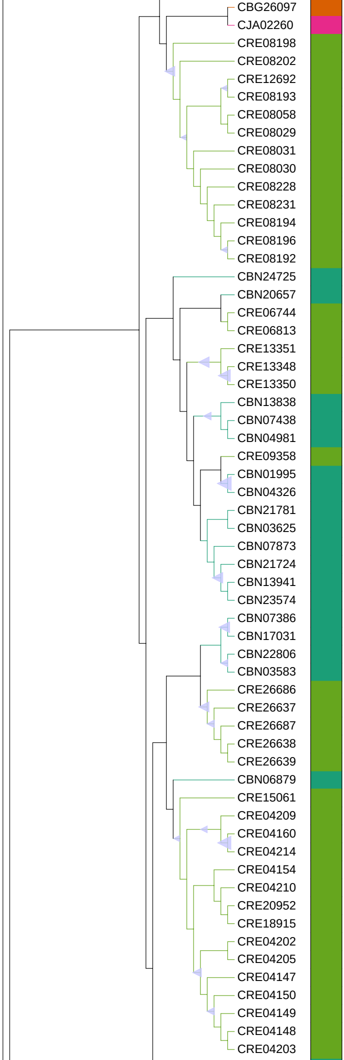
CBG26097
CJA02260
CRE08198
CRE08202
CRE12692
CRE08193
CRE08058
CRE08029
CRE08031
CRE08030
CRE08228
CRE08231
CRE08194
CRE08196
CRE08192
CBN24725
CBN20657
CRE06744
CRE06813
CRE13351
CRE13348
CRE13350
CBN13838
CBN07438
CBN04981
CRE09358
CBN01995
CBN04326
CBN21781
CBN03625
CBN07873
CBN21724
CBN13941
CBN23574
CBN07386
CBN17031
CBN22806
CBN03583
CRE26686
CRE26637
CRE26687
CRE26638
CRE26639
CBN06879
CRE15061
CRE04209
CRE04160
CRE04214
CRE04154
CRE04210
CRE20952
CRE18915
CRE04202
CRE04205
CRE04147
CRE04150
CRE04149
CRE04148
CRE04203

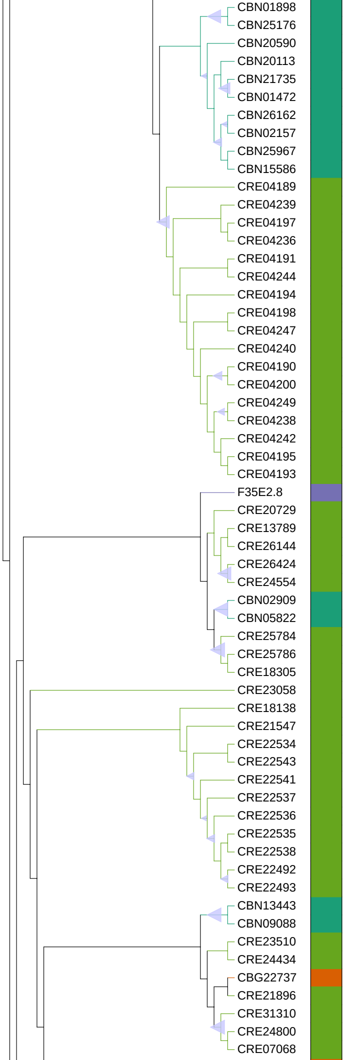
CBN01898
CBN25176
CBN20590
CBN20113
CBN21735
CBN01472
CBN26162
CBN02157
CBN25967
CBN15586
CRE04189
CRE04239
CRE04197
CRE04236
CRE04191
CRE04244
CRE04194
CRE04198
CRE04247
CRE04240
CRE04190
CRE04200
CRE04249
CRE04238
CRE04242
CRE04195
CRE04193
F35E2.8
CRE20729
CRE13789
CRE26144
CRE26424
CRE24554
CBN02909
CBN05822
CRE25784
CRE25786
CRE18305
CRE23058
CRE18138
CRE21547
CRE22534
CRE22543
CRE22541
CRE22537
CRE22536
CRE22535
CRE22538
CRE22492
CRE22493
CBN13443
CBN09088
CRE23510
CRE24434
CBG22737
CRE21896
CRE31310
CRE24800
CRE07068

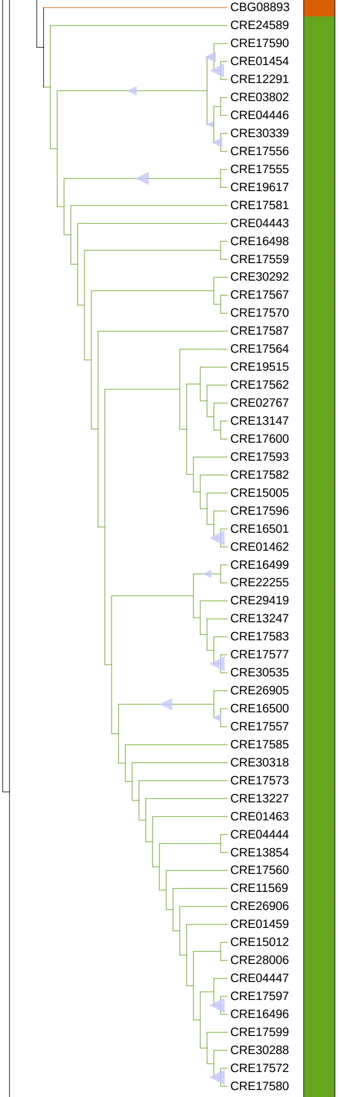
CBG08893
CRE24589
CRE17590
CRE01454
CRE12291
CRE03802
CRE04446
CRE30339
CRE17556
CRE17555
CRE19617
CRE17581
CRE04443
CRE16498
CRE17559
CRE30292
CRE17567
CRE17570
CRE17587
CRE17564
CRE19515
CRE17562
CRE02767
CRE13147
CRE17600
CRE17593
CRE17582
CRE15005
CRE17596
CRE16501
CRE01462
CRE16499
CRE22255
CRE29419
CRE13247
CRE17583
CRE17577
CRE30535
CRE26905
CRE16500
CRE17557
CRE17585
CRE30318
CRE17573
CRE13227
CRE01463
CRE04444
CRE13854
CRE17560
CRE11569
CRE26906
CRE01459
CRE15012
CRE28006
CRE04447
CRE17597
CRE16496
CRE17599
CRE30288
CRE17572
CRE17580

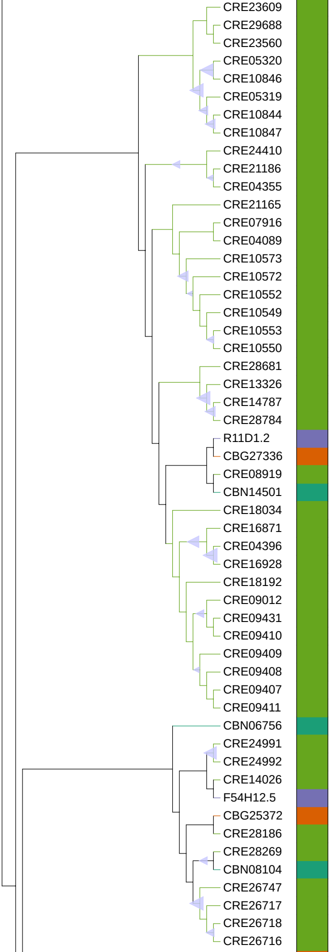
CRE23609
CRE29688
CRE23560
CRE05320
CRE10846
CRE05319
CRE10844
CRE10847
CRE24410
CRE21186
CRE04355
CRE21165
CRE07916
CRE04089
CRE10573
CRE10572
CRE10552
CRE10549
CRE10553
CRE10550
CRE28681
CRE13326
CRE14787
CRE28784
R11D1.2
CBG27336
CRE08919
CBN14501
CRE18034
CRE16871
CRE04396
CRE16928
CRE18192
CRE09012
CRE09431
CRE09410
CRE09409
CRE09408
CRE09407
CRE09411
CBN06756
CRE24991
CRE24992
CRE14026
F54H12.5
CBG25372
CRE28186
CRE28269
CBN08104
CRE26747
CRE26717
CRE26718
CRE26716

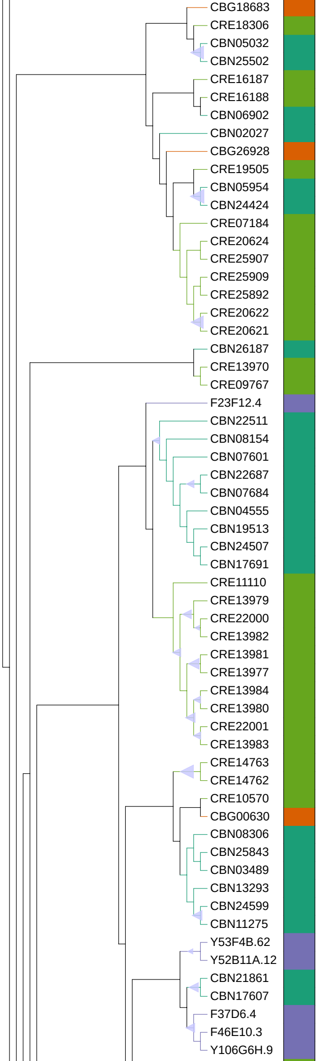

CBG18683
CRE18306
CBN05032
CBN25502
CRE16187
CRE16188
CBN06902
CBN02027
CBG26928
CRE19505
CBN05954
CBN24424
CRE07184
CRE20624
CRE25907
CRE25909
CRE25892
CRE20622
CRE20621
CBN26187
CRE13970
CRE09767
F23F12.4
CBN22511
CBN08154
CBN07601
CBN22687
CBN07684
CBN04555
CBN19513
CBN24507
CBN17691
CRE11110
CRE13979
CRE22000
CRE13982
CRE13981
CRE13977
CRE13984
CRE13980
CRE22001
CRE13983
CRE14763
CRE14762
CRE10570
CBG00630
CBN08306
CBN25843
CBN03489
CBN13293
CBN24599
CBN11275
Y53F4B.62
Y52B11A.12
CBN21861
CBN17607
F37D6.4
F46E10.3
Y106G6H.9

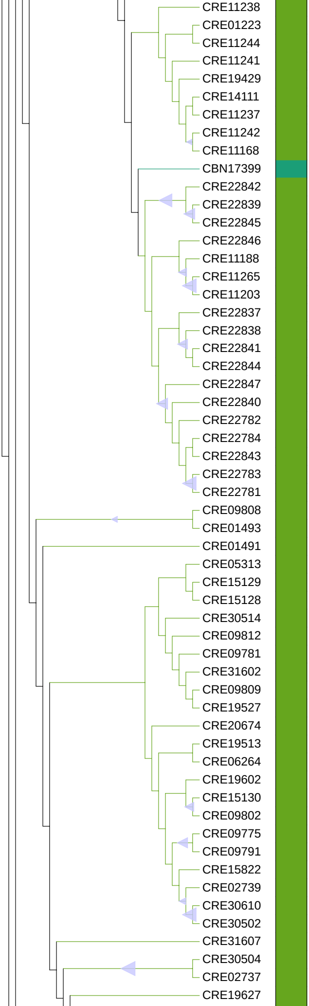
CRE11238
CRE01223
CRE11244
CRE11241
CRE19429
CRE14111
CRE11237
CRE11242
CRE11168
CBN17399
CRE22842
CRE22839
CRE22845
CRE22846
CRE11188
CRE11265
CRE11203
CRE22837
CRE22838
CRE22841
CRE22844
CRE22847
CRE22840
CRE22782
CRE22784
CRE22843
CRE22783
CRE22781
CRE09808
CRE01493
CRE01491
CRE05313
CRE15129
CRE15128
CRE30514
CRE09812
CRE09781
CRE31602
CRE09809
CRE19527
CRE20674
CRE19513
CRE06264
CRE19602
CRE15130
CRE09802
CRE09775
CRE09791
CRE15822
CRE02739
CRE30610
CRE30502
CRE31607
CRE30504
CRE02737
CRE19627

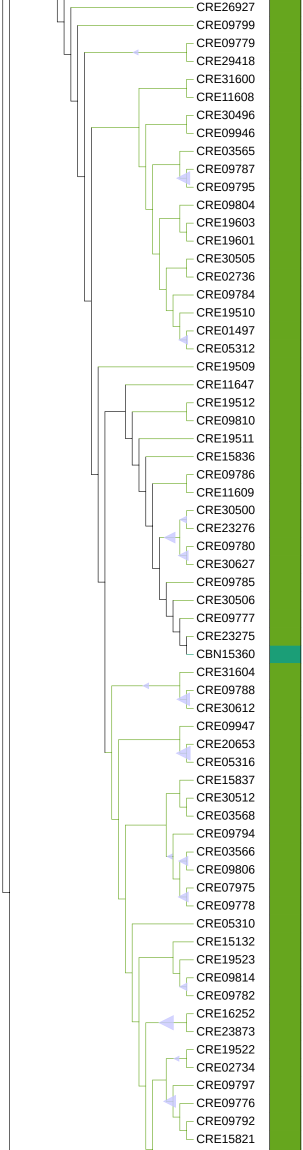
CRE26927
CRE09799
CRE09779
CRE29418
CRE31600
CRE11608
CRE30496
CRE09946
CRE03565
CRE09787
CRE09795
CRE09804
CRE19603
CRE19601
CRE30505
CRE02736
CRE09784
CRE19510
CRE01497
CRE05312
CRE19509
CRE11647
CRE19512
CRE09810
CRE19511
CRE15836
CRE09786
CRE11609
CRE30500
CRE23276
CRE09780
CRE30627
CRE09785
CRE30506
CRE09777
CRE23275
CBN15360
CRE31604
CRE09788
CRE30612
CRE09947
CRE20653
CRE05316
CRE15837
CRE30512
CRE03568
CRE09794
CRE03566
CRE09806
CRE07975
CRE09778
CRE05310
CRE15132
CRE19523
CRE09814
CRE09782
CRE16252
CRE23873
CRE19522
CRE02734
CRE09797
CRE09776
CRE09792
CRE15821

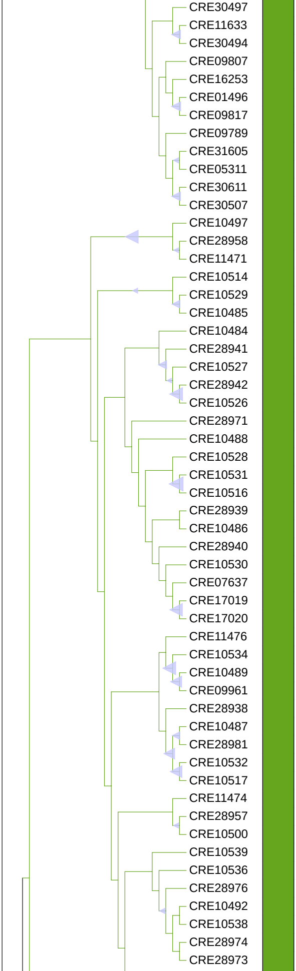
CRE30497
CRE11633
CRE30494
CRE09807
CRE16253
CRE01496
CRE09817
CRE09789
CRE31605
CRE05311
CRE30611
CRE30507
CRE10497
CRE28958
CRE11471
CRE10514
CRE10529
CRE10485
CRE10484
CRE28941
CRE10527
CRE28942
CRE10526
CRE28971
CRE10488
CRE10528
CRE10531
CRE10516
CRE28939
CRE10486
CRE28940
CRE10530
CRE07637
CRE17019
CRE17020
CRE11476
CRE10534
CRE10489
CRE09961
CRE28938
CRE10487
CRE28981
CRE10532
CRE10517
CRE11474
CRE28957
CRE10500
CRE10539
CRE10536
CRE28976
CRE10492
CRE10538
CRE28974
CRE28973

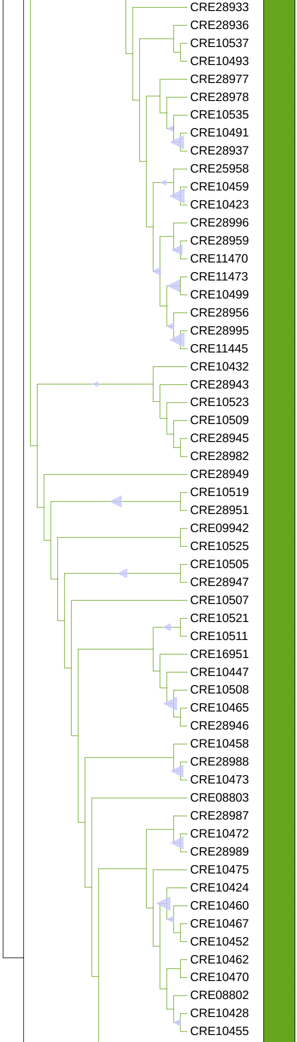
CRE28933
CRE28936
CRE10537
CRE10493
CRE28977
CRE28978
CRE10535
CRE10491
CRE28937
CRE25958
CRE10459
CRE10423
CRE28996
CRE28959
CRE11470
CRE11473
CRE10499
CRE28956
CRE28995
CRE11445
CRE10432
CRE28943
CRE10523
CRE10509
CRE28945
CRE28982
CRE28949
CRE10519
CRE28951
CRE09942
CRE10525
CRE10505
CRE28947
CRE10507
CRE10521
CRE10511
CRE16951
CRE10447
CRE10508
CRE10465
CRE28946
CRE10458
CRE28988
CRE10473
CRE08803
CRE28987
CRE10472
CRE28989
CRE10475
CRE10424
CRE10460
CRE10467
CRE10452
CRE10462
CRE10470
CRE08802
CRE10428
CRE10455

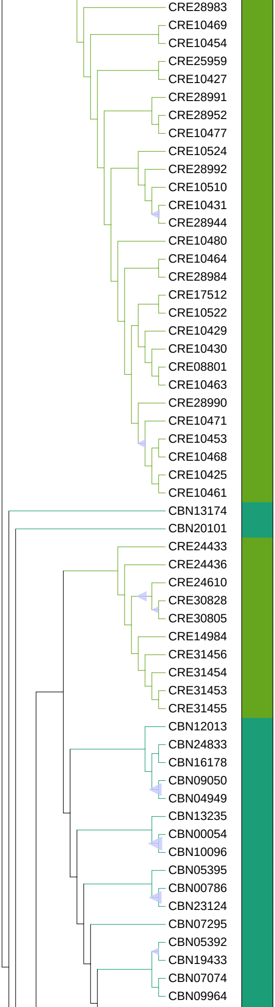
CRE28983
CRE10469
CRE10454
CRE25959
CRE10427
CRE28991
CRE28952
CRE10477
CRE10524
CRE28992
CRE10510
CRE10431
CRE28944
CRE10480
CRE10464
CRE28984
CRE17512
CRE10522
CRE10429
CRE10430
CRE08801
CRE10463
CRE28990
CRE10471
CRE10453
CRE10468
CRE10425
CRE10461
CBN13174
CBN20101
CRE24433
CRE24436
CRE24610
CRE30828
CRE30805
CRE14984
CRE31456
CRE31454
CRE31453
CRE31455
CBN12013
CBN24833
CBN16178
CBN09050
CBN04949
CBN13235
CBN00054
CBN10096
CBN05395
CBN00786
CBN23124
CBN07295
CBN05392
CBN19433
CBN07074
CBN09964

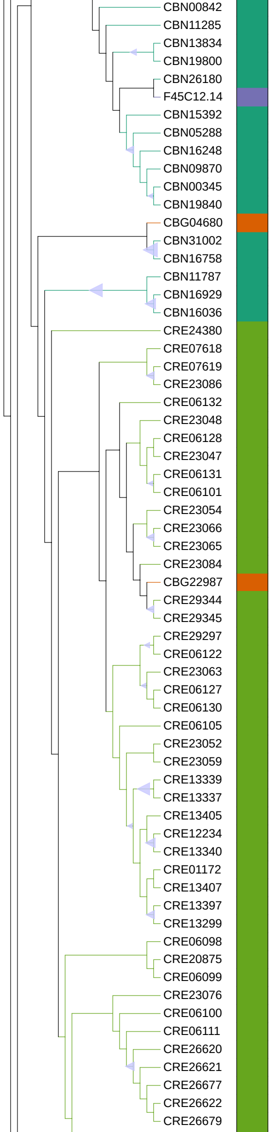
CBN00842
CBN11285
CBN13834
CBN19800
CBN26180
F45C12.14
CBN15392
CBN05288
CBN16248
CBN09870
CBN00345
CBN19840
CBG04680
CBN31002
CBN16758
CBN11787
CBN16929
CBN16036
CRE24380
CRE07618
CRE07619
CRE23086
CRE06132
CRE23048
CRE06128
CRE23047
CRE06131
CRE06101
CRE23054
CRE23066
CRE23065
CRE23084
CBG22987
CRE29344
CRE29345
CRE29297
CRE06122
CRE23063
CRE06127
CRE06130
CRE06105
CRE23052
CRE23059
CRE13339
CRE13337
CRE13405
CRE12234
CRE13340
CRE01172
CRE13407
CRE13397
CRE13299
CRE06098
CRE20875
CRE06099
CRE23076
CRE06100
CRE06111
CRE26620
CRE26621
CRE26677
CRE26622
CRE26679

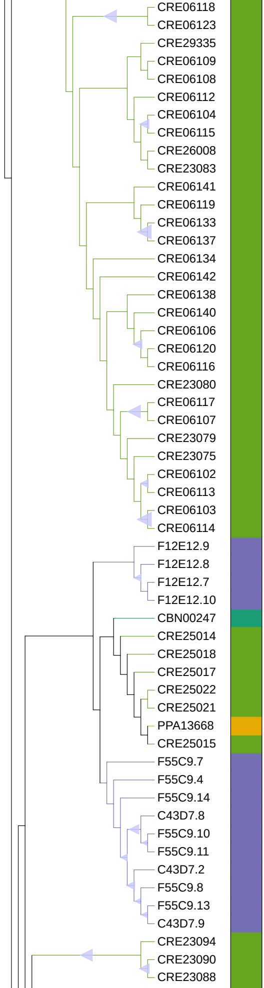
CRE06118
CRE06123
CRE29335
CRE06109
CRE06108
CRE06112
CRE06104
CRE06115
CRE26008
CRE23083
CRE06141
CRE06119
CRE06133
CRE06137
CRE06134
CRE06142
CRE06138
CRE06140
CRE06106
CRE06120
CRE06116
CRE23080
CRE06117
CRE06107
CRE23079
CRE23075
CRE06102
CRE06113
CRE06103
CRE06114
F12E12.9
F12E12.8
F12E12.7
F12E12.10
CBN00247
CRE25014
CRE25018
CRE25017
CRE25022
CRE25021
PPA13668
CRE25015
F55C9.7
F55C9.4
F55C9.14
C43D7.8
F55C9.10
F55C9.11
C43D7.2
F55C9.8
F55C9.13
C43D7.9
CRE23094
CRE23090
CRE23088

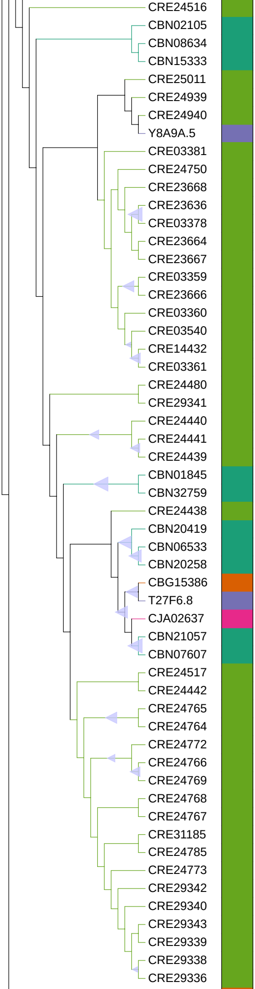
CRE24516
CBN02105
CBN08634
CBN15333
CRE25011
CRE24939
CRE24940
Y8A9A.5
CRE03381
CRE24750
CRE23668
CRE23636
CRE03378
CRE23664
CRE23667
CRE03359
CRE23666
CRE03360
CRE03540
CRE14432
CRE03361
CRE24480
CRE29341
CRE24440
CRE24441
CRE24439
CBN01845
CBN32759
CRE24438
CBN20419
CBN06533
CBN20258
CBG15386
T27F6.8
CJA02637
CBN21057
CBN07607
CRE24517
CRE24442
CRE24765
CRE24764
CRE24772
CRE24766
CRE24769
CRE24768
CRE24767
CRE31185
CRE24785
CRE24773
CRE29342
CRE29340
CRE29343
CRE29339
CRE29338
CRE29336

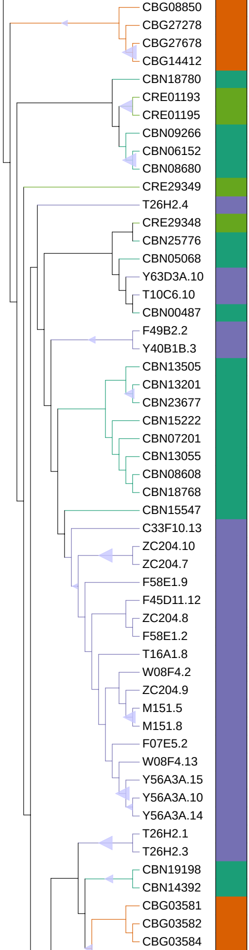
CBG08850
CBG27278
CBG27678
CBG14412
CBN18780
CRE01193
CRE01195
CBN09266
CBN06152
CBN08680
CRE29349
T26H2.4
CRE29348
CBN25776
CBN05068
Y63D3A.10
T10C6.10
CBN00487
F49B2.2
Y40B1B.3
CBN13505
CBN13201
CBN23677
CBN15222
CBN07201
CBN13055
CBN08608
CBN18768
CBN15547
C33F10.13
ZC204.10
ZC204.7
F58E1.9
F45D11.12
ZC204.8
F58E1.2
T16A1.8
W08F4.2
ZC204.9
M151.5
M151.8
F07E5.2
W08F4.13
Y56A3A.15
Y56A3A.10
Y56A3A.14
T26H2.1
T26H2.3
CBN19198
CBN14392
CBG03581
CBG03582
CBG03584

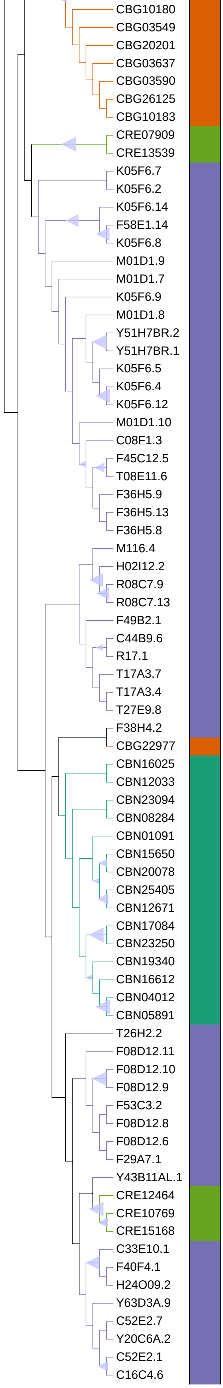
CBG10180
CBG03549
CBG20201
CBG03637
CBG03590
CBG26125
CBG10183
CRE07909
CRE13539
K05F6.7
K05F6.2
K05F6.14
F58E1.14
K05F6.8
M01D1.9
M01D1.7
K05F6.9
M01D1.8
Y51H7BR.2
Y51H7BR.1
K05F6.5
K05F6.4
K05F6.12
M01D1.10
C08F1.3
F45C12.5
T08E11.6
F36H5.9
F36H5.13
F36H5.8
M116.4
H02I12.2
R08C7.9
R08C7.13
F49B2.1
C44B9.6
R17.1
T17A3.7
T17A3.4
T27E9.8
F38H4.2
CBG22977
CBN16025
CBN12033
CBN23094
CBN08284
CBN01091
CBN15650
CBN20078
CBN25405
CBN12671
CBN17084
CBN23250
CBN19340
CBN16612
CBN04012
CBN05891
T26H2.2
F08D12.11
F08D12.10
F08D12.9
F53C3.2
F08D12.8
F08D12.6
F29A7.1
Y43B11AL.1
CRE12464
CRE10769
CRE15168
C33E10.1
F40F4.1
H24O09.2
Y63D3A.9
C52E2.7
Y20C6A.2
C52E2.1
C16C4.6
